# Vicinal Diaryl-Substituted Isoxazole and Pyrazole Derivatives with *In Vitro* Growth Inhibitory and *In Vivo* Antitumor Activity

**DOI:** 10.1021/acsomega.2c03405

**Published:** 2022-10-03

**Authors:** Sümeyye Turanlı, Esra Nalbat, Deniz Lengerli, Kübra İbiş, Sezen Güntekin Ergün, Ece Akhan Güzelcan, Mesut Muyan, Rengul Cetin-Atalay, Burcu Çalışkan, Erden Banoglu

**Affiliations:** † Department of Pharmaceutical Chemistry, Faculty of Pharmacy, Gazi University, Yenimahalle, Ankara 06560, Turkey; ‡ Cancer Systems Biology Laboratory, Graduate School of Informatics, Middle East Technical University, Ankara 06800, Turkey; § Department of Biological Sciences, Middle East Technical University, Ankara 06800, Turkey

## Abstract

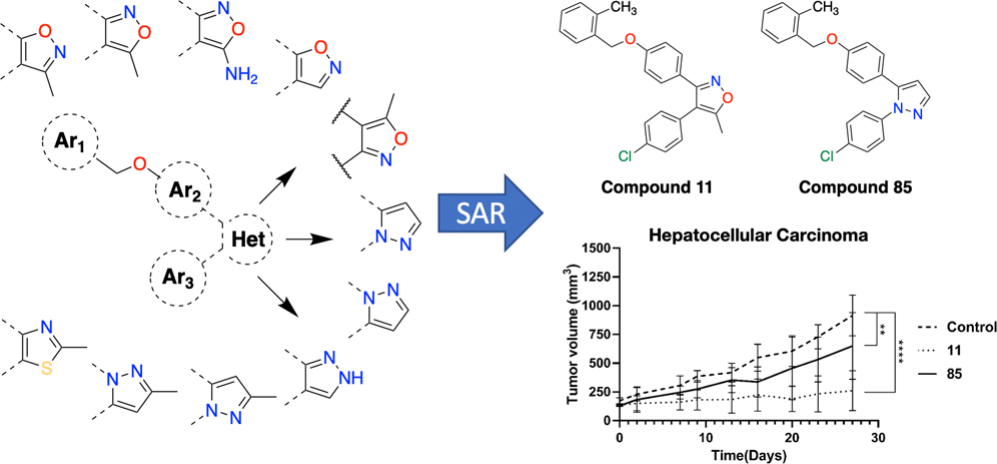

The vicinal diaryl heterocyclic framework has been widely used for the development of compounds with significant bioactivities. In this study, a series of diaryl heterocycles were designed and synthesized based on an in-house diaryl isoxazole derivative (**9**), and most of the newly synthesized derivatives demonstrated moderate to good antiproliferative activities against a panel of hepatocellular carcinoma and breast cancer cells, exemplified with the diaryl isoxazole **11** and the diaryl pyrazole **85** with IC_50_ values in the range of 0.7–9.5 μM. Treatments with both **11** and **85** induced apoptosis in these tumor cells, and they displayed antitumor activity *in vivo* in the Mahlavu hepatocellular carcinoma and the MDA-MB-231 breast cancer xenograft models, indicating that these compounds could be considered as leads for further development of antitumor agents. Important structural features of this compound class for the antitumor activity have also been proposed, which warrant further exploration to guide the design of new and more potent diaryl heterocycles.

## 1. Introduction

**Figure 1:**
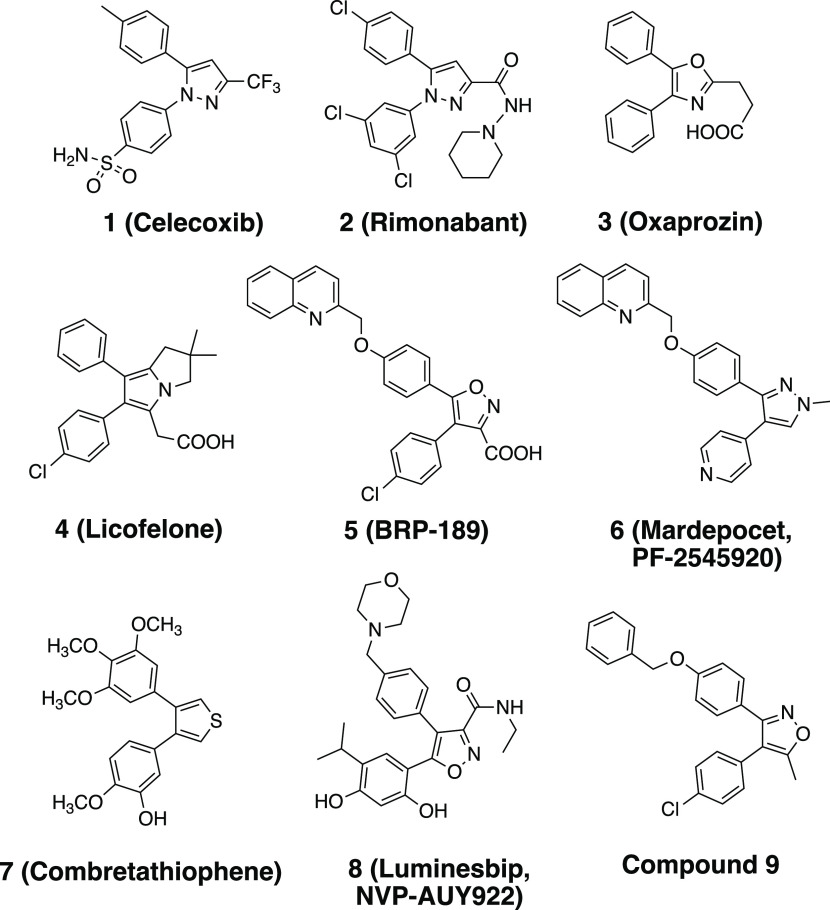
Examples of clinical drugs and experimental compounds with therapeutic potential having the vicinal diaryl heterocyclic motif.

Vicinal diaryl-substituted heterocycles can be considered privileged scaffolds as they form the core structure of a number of compounds with diverse biological properties such as anticancer, antiviral, and anti-inflammatory activities.^1^ Therefore, the use of this scaffold incorporating different central heterocycles could be pivotal in the drug development practice since they exist in the structures of various clinical therapeutics or compounds with therapeutic potential, which establishes their value as chemical tools in the field of medicinal chemistry (Figure 1). For example, the first COX-2 selective nonsteroidal anti-inflammatory drug celecoxib (**1**) was a member of vicinal diaryl pyrazole derivatives,^2^ which stimulated the development of latter COX-2 inhibitors belonging to the vicinal diaryl heterocyclic class such as rofecoxib and valdecoxib.^3^ Another vicinal diaryl pyrazole derivative developed as an antiobesity drug was rimonabant (**2**),^4^ which continues to be an inspiration for further development of cannabinoid CB1 receptor antagonists, i.e., surinabant.^5^ Oxaprozin (**3**) and licofelone (**4**) are anti-inflammatory drugs for the treatment of osteo- and rheumatoid arthritis, also belonging to a vicinal diaryl oxazole and pyrrolizidine class, respectively.^6,7^

Furthermore, a considerable number of vicinal diaryl heterocyclic motifs spread throughout the literature with various biological activities.^1^ For example, a 4,5-diaryl isoxazole derivative BRP-187 (**5**) was reported to be a 5-lipoxygenase-activating protein (FLAP) inhibitor with potent anti-inflammatory activity.^8,9^ The 3,4-diarylpyrazole derivative mardepodect (**6**, PF-2545920) was discovered as a phosphodiesterase (PDE) 10A inhibitor,^10^ which progressed through Phase-II clinical trials for Schizophrenia treatment, and was used as a model compound leading to a considerable number of follow-up PDE10A inhibitors having vicinal diaryl heterocyclic framework.^11^ In addition, a good number of vicinal diaryl-substituted heterocycles were studied as anticancer therapeutics in which the vicinal diaryl scaffold was shown to be a simple pharmacophore for tubulin polymerization inhibitors mimicking the features of combretastatin A4 with potent anticancer activity as exemplified with combretatiophene (**7**).^12^ In addition, 4,5-diarylisoxazole luminespib (**8**, NVP-AUY922) was developed as an Hsp90 inhibitor for the treatment of several cancer types and reached Phase-II clinical trials.^13^

Breast cancer (BC) is the most commonly occurring cancer in women and the most common cancer overall, while hepatocellular carcinoma (HCC) is the sixth most common and the second most lethal cancer.^14^ A large number of treatment options including localized and/or systematic therapies are available for both cancer types. Despite the initial effectiveness in controlling tumor growth and prolonging patient survival, nearly all current treatments result in resistance to therapies. This necessitates the development of novel approaches as well as effective chemotherapeutic agents for cancer treatments. We have long been working with the vicinal diaryl heterocyclic skeleton toward novel anti-inflammatory, antiplatelet, and anticancer agents.^8,9,15−20^ In this context, inspired by the therapeutic potential of the vicinal diaryl motif around a central heterocycle, our compiled library of compounds belonging to this promising class was screened for their potential cytotoxic activity against an HCC cell line. Compound **9** with 3,4-diaryl isoxazole motif with synthetic accessibility for compound library generation was identified, although the cytotoxicity against HCC cells was negligible (IC_50_ ≥ 20 μM) (Figure 1, Table 1). However, encouraged by the anticancer potential of vicinal diaryl heterocyclic scaffold and the synthetic feasibility of compound **9** skeleton, a focused library of compounds around compound **9** was prepared by decorating the vicinal diaryl rings and by incorporating various heterocyclic rings as the central core, which resulted in new analogues with improved cytotoxicity against both HCC and BC cancer cell lines. Here, we report the biological assessment of a new set of compounds that we have developed against cell lines derived from BC and HCC, which would be valuable for the understanding of the main features of these new vicinal diaryl heterocyclic analogues as anticancer therapeutic leads.

**Table 1:**
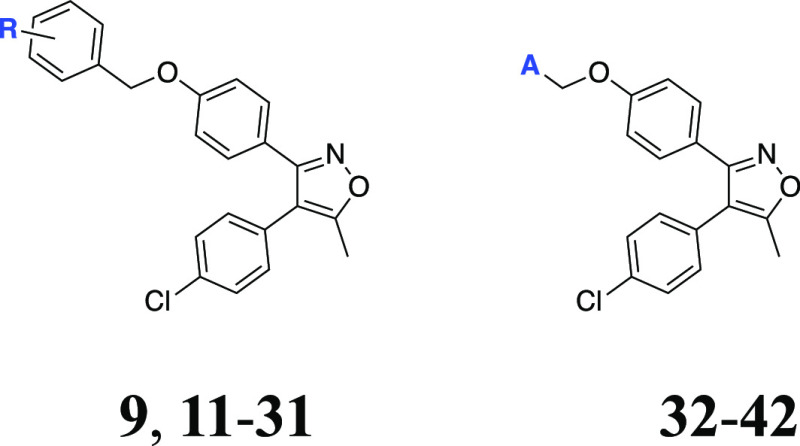

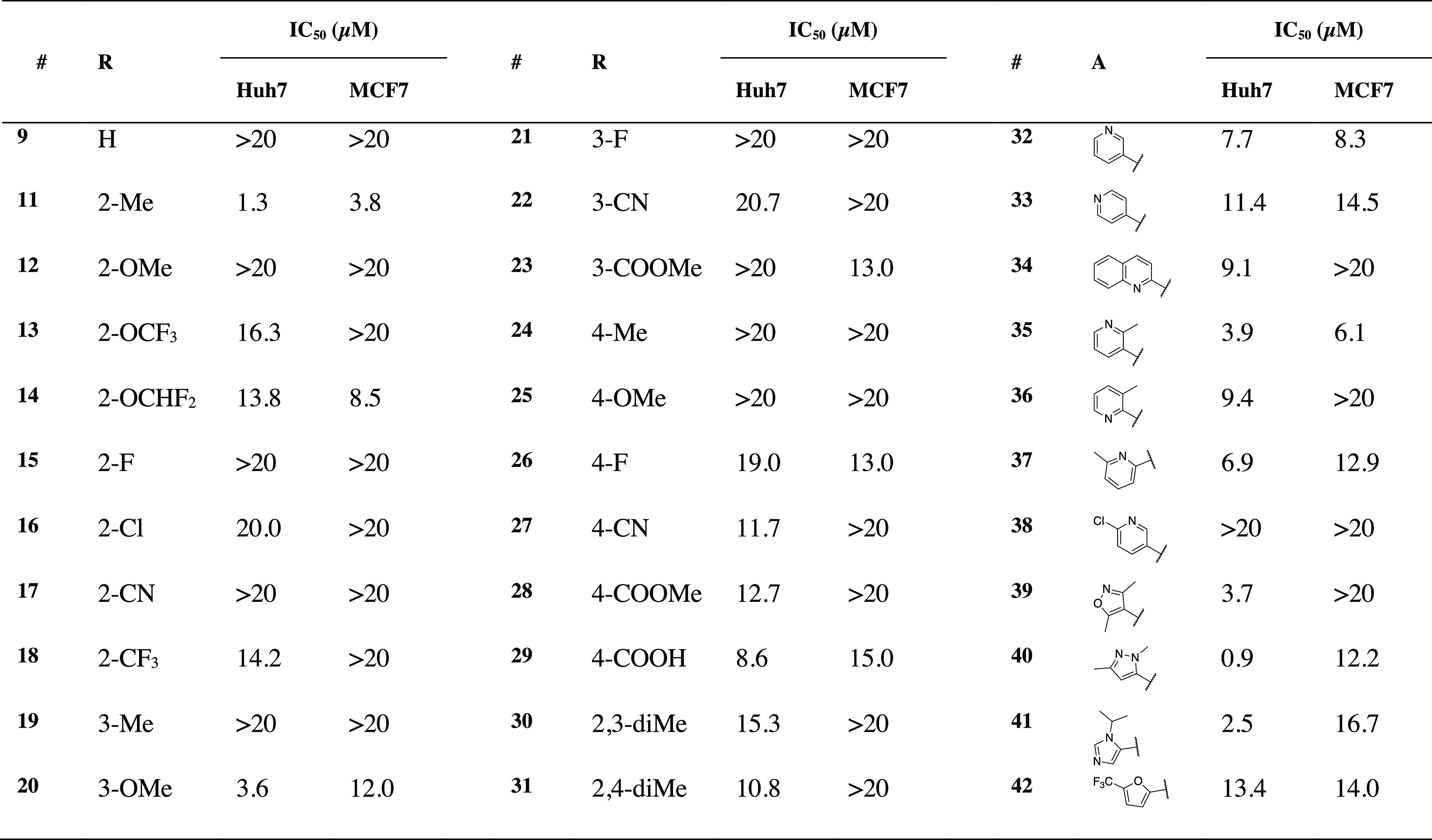
*In Vitro* Cell Growth Inhibitory Activity against Hepatocellular Carcinoma and Breast Cancer Cell Lines^a^

a IC_50_ values were determined at least with five different concentrations of the compounds from the cell growth inhibition percentages.

## Results and Discussion

### Chemistry

**Scheme 1:**
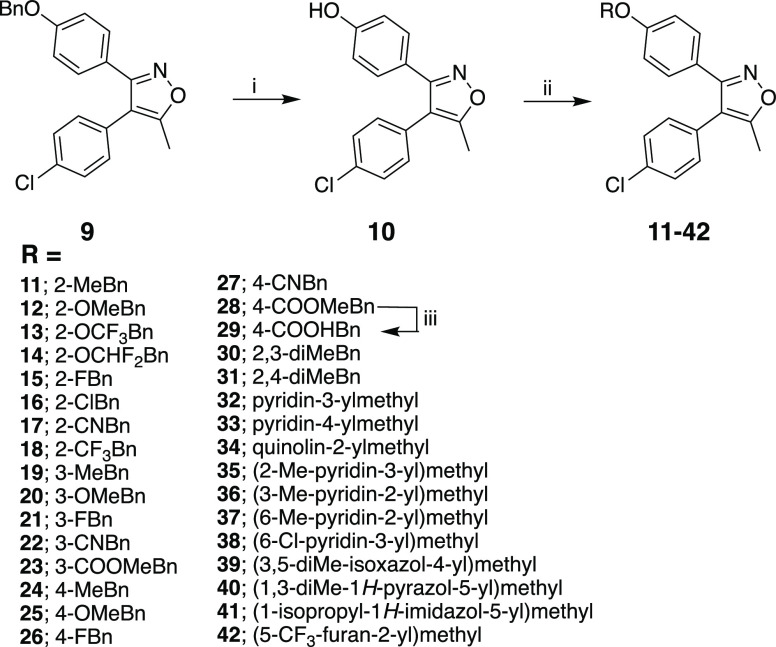
Reaction Conditions and Reagents: (i) Pd/C (10% w/w), HCl, EtOH, rt; (ii) Aryl/Alkyl Halide Derivative, K_2_CO_3_, MeCN, Δ; (iii) LiOH·H_2_O, THF:water, Δ

**Scheme 2:**
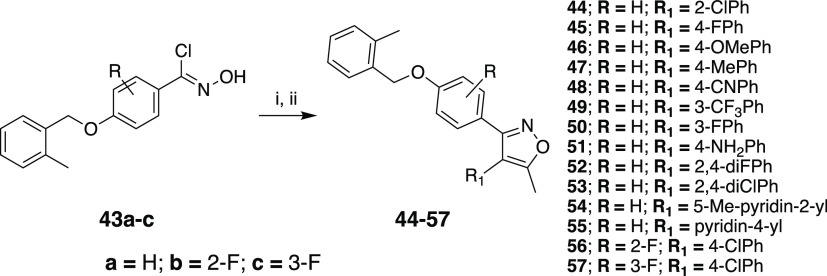
Reaction Conditions and Reagents: (i) TEA, Diethyl Ether, 0 °C; (ii) Phenyl/pyridyl Acetone Derivative, NaH, THF, 0 °C

**Scheme 3:**
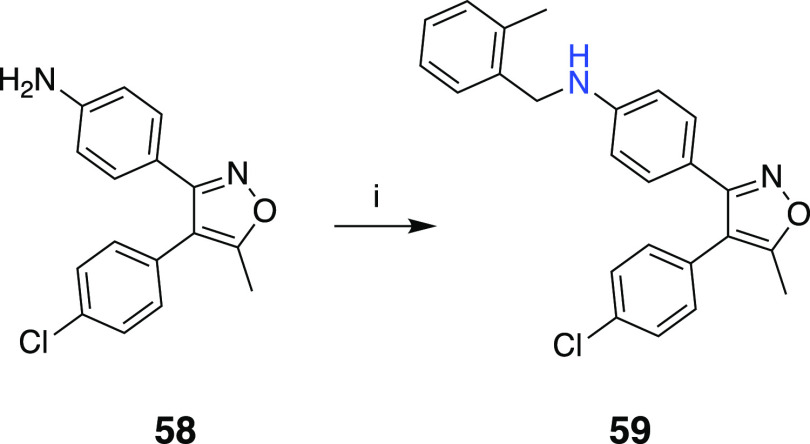
Reaction Conditions and Reagents: (i) 2-Methylbenzyl Bromide, DIEA, DMF, 65 °C

Based on the developable potential of compound **9**, we developed additional analogues to establish structure–activity relationships (SAR). New 3,4-diaryl-5-methylisoxazole derivatives **11**-**42** were synthesized according to the literature,^21^ and synthesis procedures and schemes of the relevant intermediates are given in the Supporting Information. In brief, hydrogenation of **9** to remove the benzyl group produced the key intermediate **10**, which was subsequently used to generate desired final compounds **11**–**42** through alkylation of the phenolic hydroxyl (Scheme 1). The similar procedures in Scheme 1 were successfully applied to the synthesis of **44–57** with modifications at the middle phenyl and **59** with amine linker, following the reaction conditions in Schemes 2 and 3. The synthesis of 4,5-diaryl-3-methylisoxazole analogue **60** (Table 3) was achieved as we previously reported.^8^

**Scheme 4:**
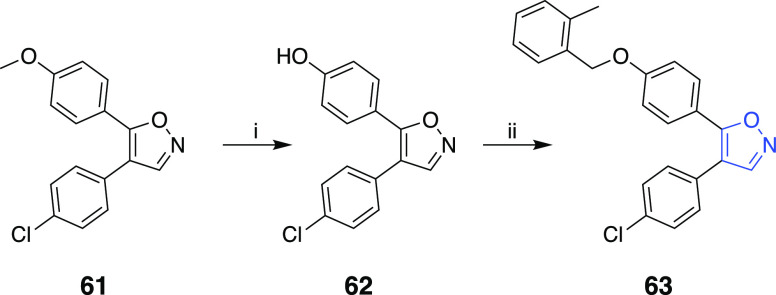
Reaction Conditions and Reagents: (i) BBr_3_, DCM, 0 °C; (ii) 2-Methylbenzyl Bromide, K_2_CO_3_, MeCN, MWI, 120 °C, 20 min

The preparation of 4,5-diarylisoxazole derivative **63** was achieved starting with readily available starting materials as illustrated in Scheme 4. Compound **61** was obtained according to the corresponding procedures.^10,22^ The obtained 4,5-diarylisoxazole framework **62** was subsequently used to produce the desired **63** through first the hydrolysis of the methoxy and then the alkylation of the phenolic hydroxyl group (Scheme 4).

**Scheme 5:**
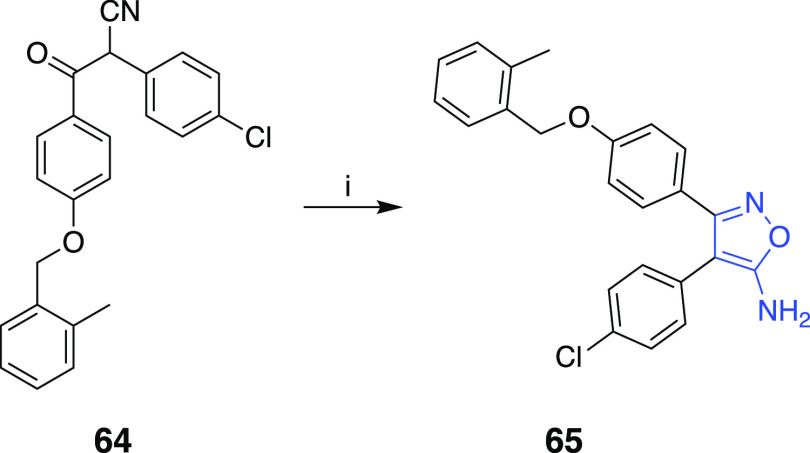
Reaction Conditions and Reagents: (i) (4-Chlorophenyl)acetonitrile, LHMDS, THF, −70 °C; (vi) NH_2_OH·HCl, EtOH, Δ

**Scheme 6:**
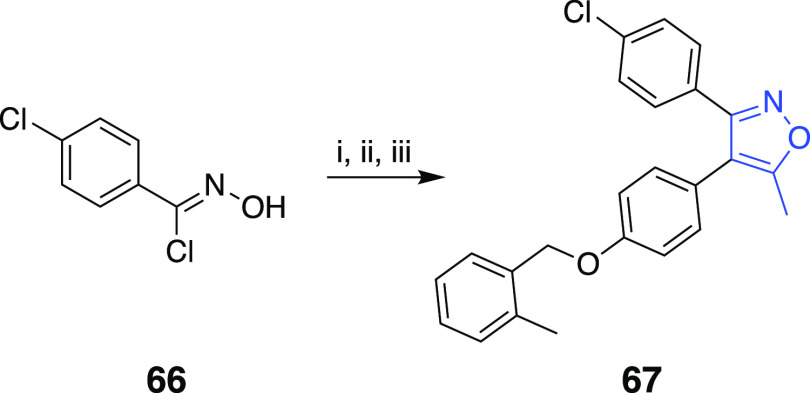
Reaction Conditions and Reagents: (i) TEA, Diethyl Ether, 0 °C; (ii) 4-((2-Methylbenzyl)oxy)phenylacetone, NaH, THF, 0 °C; (iii) Na_2_CO_3_, MeOH, H_2_O, Δ

The 3,4-diaryl-5-aminoisoxazole congener **65** was synthesized as shown in Scheme 5. The 3,4-diaryl-5-aminoisoxazole **65** was synthesized by cyclization of the β-cyanoketone **64**, which was obtained according to the literature procedure.^23,24^ The synthesis of 3,4-diaryl-5-methylisoxazole **67** where the 2-methylbenzyloxy arm was relocated on the 4-aryl ring was analogous to the similar chemistry that has been described in Scheme 1 (Scheme 6).

**Scheme 7:**
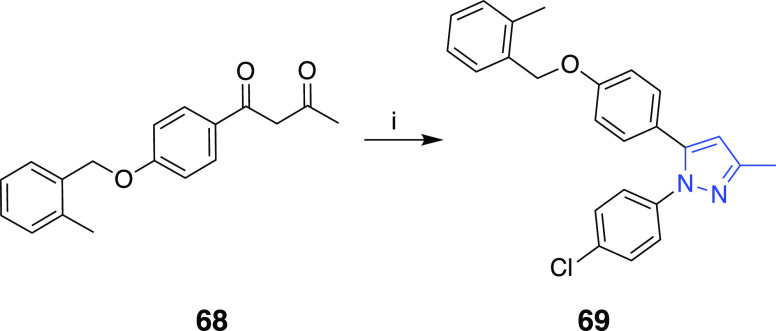
Reaction Conditions and Reagents: (i) 4-Chlorophenylhydrazine·HCl, TEA, MeOH, Δ

The preparation of the 1,5-diaryl-3-methylpyrazole derivative **69** carrying the 2-methylbenzyloxy arm on 5-aryl group is outlined in Scheme 7. The synthesis of the β-diketone **68** was achieved by Claisen–Schmidt condensation.^25^ The regioselective synthesis of the 1,5-diaryl-3-methylpyrazole **69** was then conveniently achieved by condensation of the diketone **68** with the hydrochloride salt of the 4-chlorophenylhydrazine in methanol/triethylamine.^15^

**Scheme 8:**
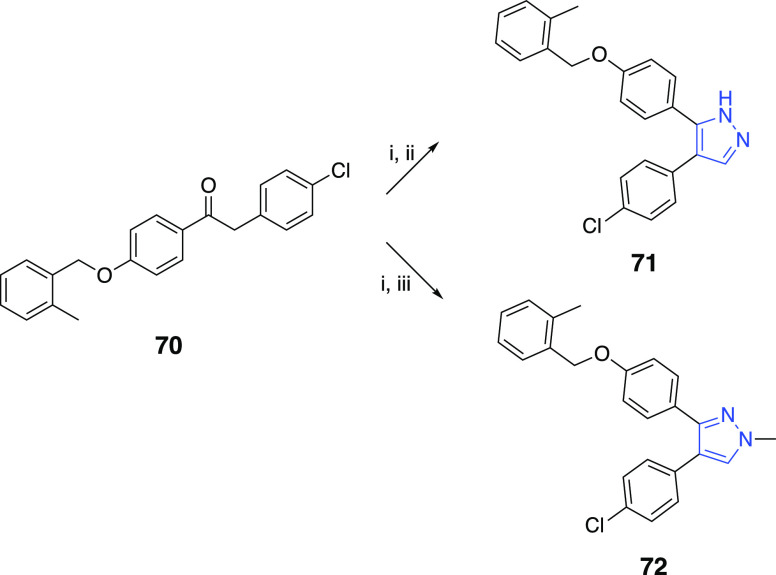
Reaction Conditions and Reagents: (i) DMFDMA, DMF; (ii) NH_2_·H_2_O, MeOH, Δ; (iii) Methylhydrazine, MeOH, Δ

**Scheme 9:**
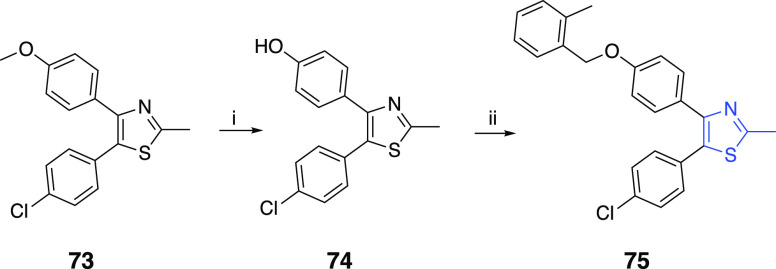
Reaction Conditions and Reagents: (i) BBr_3_, DCM, 0 °C; (ii) 2-Methylbenzyl Bromide, K_2_CO_3_, MeCN, Δ

The central pyrazole series continued with the synthesis of 4,5-diaryl-1*H*-pyrazole **71** and 3,4-diaryl-1-methylpyrazole **72**. Briefly, the in situ formed enaminone intermediate, obtained by the treatment of **70** with DMFDMA,^10^ was cyclized by hydrazine or methylhydrazine to produce the desired pyrazole compounds **71** and **72**, respectively (Scheme 8). For the synthesis of the vicinal diaryl 2-methylthiazole **75**, hydrolysis of **73** to remove the methyl group furnished the intermediate **74**, which was utilized to generate the desired 4,5-diarylthiazole compound **75** through the benzylation of the phenolic hydroxyl (Scheme 9).

**Scheme 10:**
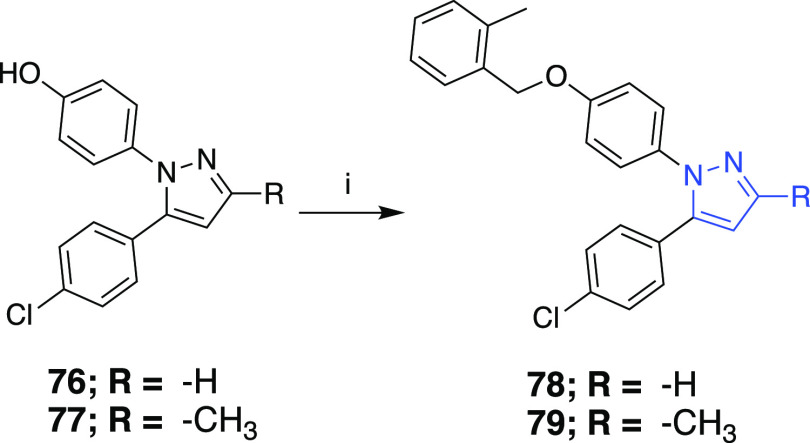
Reaction Conditions and Reagents: (i) 2-Methylbenzyl Bromide, K_2_CO_3_, MeCN, Δ

1,5-Diarylpyrazole scaffolds **78** and **79** were produced according to previously developed procedures (Scheme 10).^26^

**Scheme 11:**
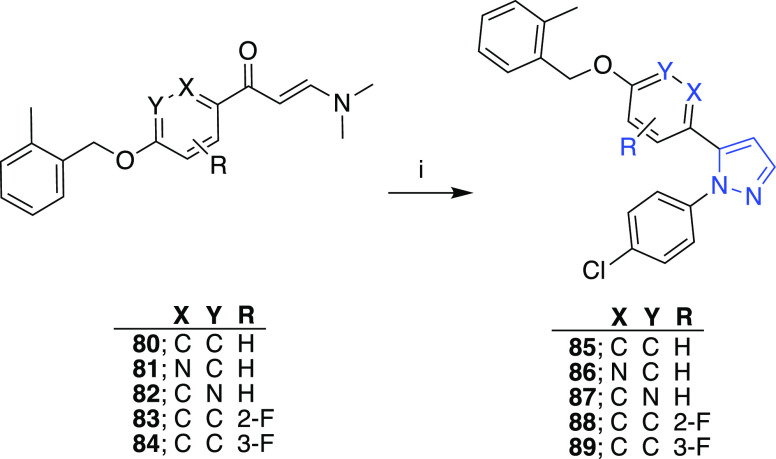
Reaction Conditions and Reagents: (i) 4-Chlorophenylhydrazine·HCl, EtOH, Δ

**Scheme 12:**
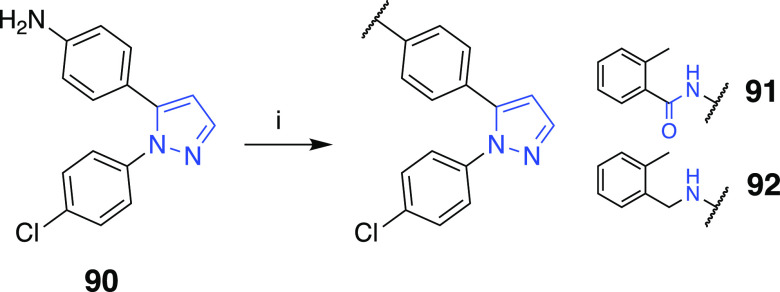
Reaction Conditions and Reagents: (i) 2-Methylbenzoylchloride, DIEA, DCM, rt for Compound **91**; 2-Methylbenzyl Bromide, DIEA, DMF, 65 °C for Compound **92**

1,5-Diarylpyrazole analogues **85**–**89** bearing the benzyloxy fragment on the 5-aryl as opposed to that of **78** were obtained through the cyclization of enaminones **80**–**84** with the hydrochloride salt of the 4-chlorophenylhydrazine in ethanol (Scheme 11). In addition, analogous compounds where the ether bridge in **85** exchanged with amide **91** or amine **92** were accomplished starting from **90** and following the standard reaction steps outlined in Scheme 12.

### Bioactivity Studies and SAR

To deduce SARs, several specific areas were focused on compound **9**. Hence, it was aimed to explore (i) the influence of differently positioned substituents on the benzyloxy arm and the replacement of the phenyl ring of the benzyl group by heteroaryl moieties, (ii) the incorporation of different substituents at the isoxazole-4-phenyl group, (iii) the modification of the middle phenyl, and (iv) the replacement of central isoxazole with isosteric heterocycles. Collectively, a total of seventy vicinal diaryl heterocyclic compounds were synthesized to screen their inhibitory effects on cancer cell proliferation against Huh7 and MCF7 cells by introducing different chemical functionalities with the aforementioned modifications (Tables 1–4). Initially, the substitution pattern at the benzyl functionality was scrutinized by comparison of the differently substituted analogues (**11**–**42**) as illustrated in Table 1. Methylation of the aromatic ring of the benzyl at 2-position (**11**) caused a sudden increase in the potency (IC_50_ = 1.3 μM for Huh7 and 3.8 μM for MCF7) versus compound **9** (IC_50_ ≥ 20 μM for both Huh7 and MCF7) with unsubstituted benzyl arm. However, relocation of the methyl group to 3- or 4-position (**19** and **24**) led to a significant activity loss (IC_50_ ≥ 20 μM). Substitutions at 2-position other than methyl, i.e., electron-donating (**12**-**14**) and electron-withdrawing groups (**15**–**18**), were undesirable resulting in an activity decrease against both cell lines (IC_50_ values of 8.5 to >20 μM). Seemingly, voluminous substituents than methyl at 2-position were not well tolerated and impaired the efficiency of the compounds. Benzyl analogues differently substituted at 3- (**19**-**23**) or 4-position (**24**–**29**) were not chased further as they demonstrated a significant loss of inhibitory activity (IC_50_ values of 12 to >20 μM) for both cell lines except for **20** with 3-methoxy, which showed comparable activity to **11** for the Huh7 cell line (IC_50_ = 1.3 vs 3.6 μM). Compounds with dimethyl substitution pattern, i.e., 2,3-diMe (**30**) or 2,4-diMe (**31**), suggested that additional methyl group other than 2-methyl was not well tolerated on the benzyl moiety.

Next, it was explored the impact of installing a heteroaromatic group in place of the phenyl ring of the benzyl functionality (**32**–**42**). When the 2-methylphenyl was replaced by 3-pyridinyl (**32**), 4-pyridinyl (**33**), or a voluminous 2-quinonyl (**34**), these compounds showed decreased cytotoxic activity, while compound **35** with the 3-pyridine ring having a methyl at a topologically equivalent position as in **11** retained the potency with IC_50_ values of 3.9 μM for Huh7 and 6.1 μM for MCF7. A similar analogue **36** with a 2-pyridine ring having a methyl at the same position partially restored the potency against Huh7 (IC_50_ = 9.4 μM), while the activity loss toward MCF7 was more pronounced (IC_50_ ≥ 20 μM). Of interest, several five-membered heteroaromatic counterparts with alkyl substitutions such as 3,5-dimethylisoxazole (**39**), 1,3-dimethylpyrazole (**40**), and 1-isopropylimidazole (**41**) were well tolerated for their inhibition potential against Huh7 with IC_50_ values of 3.7, 0.9, and 2.5 μM, respectively, while these three compounds appeared to be less effective against MCF7 cells (IC_50_ values of 12 to >20 μM). From this point of view, the 2-methylbenzyl group remains the best choice for the 3,4-diaryl-3-methylisoxazole core, i.e., compound **11**, for cytotoxic potency albeit several five-membered heteroaryl moieties exemplified with compounds **39**–**41** are also conceivable. Apparently, a consistent SAR for cytotoxic activity at this part was not accessible since small structural differences on the benzyl arm were not well tolerated.

Then, it was investigated if the 4-chloro substituent on the phenyl ring at C(4)-isoxazole was replaceable with different atoms or groups, while keeping the 2-methylbenzyl unit due to its good impact on the activity (compounds **44**–**55** in Table 2). Moving the 4-chloro in **11** to 2-position (**44**) or replacing it with a smaller fluoro (**45**) or larger methoxy (**46**) group resulted in a decreased potency against both cell lines (IC_50_ = 9.9 to >20 μM). The amino replacement of 4-chloro (**51**) further reduced the potency for both cell types, similar to **45** and **46**. On the other hand, substitution of the 4-chloro with a methyl group (**47**), which is about the same size as a chlorine atom, was pertinent and regained the bioactivity in both cell lines (IC_50_ = 1.8 and 4.7 μM, respectively), whereas with a linear and less voluminous nitrile group in this position (**48**), the efficiency again dropped, especially against MCF7 cells (IC_50_ ≥ 20 μM). Introducing a nitrogen atom to the 4-methylphenyl ring in **47** also caused an activity loss against MCF7 as exemplified with **54**. Finally, compounds with substitutions at 3-position (**49**-**50**) or with 2,4-dihalogen substitutions (**52**–**53**) as well as with 4-pyridyl (**55**) were also significantly less effective indicating that the phenyl ring of C4-isoxazole may require substitution at 4-position with a similar steric size such as methyl or chlorine (Table 2). The next goal was to explore the substituent effect on the middle phenyl group, and this was briefly examined by fluoro substitution at 2- (**56**) and 3- (**57**) positions (Table 2). Although the 2-F substituted **56** preserved the potency against Huh7 and MCF7 cells with IC_50_ of 2.25 and 9.53 μM, respectively, 3-F substitution in the compound **57** hampered the potency against both cell lines, implying that 2-substitution on the middle phenyl ring may be allowable for retaining or even improving the activity. Lastly, our efforts at the benzyloxyphenyl component included an isosteric exchange of the ether bridge with an amino linker (**59**), which was found tolerable for the Huh7 potency (IC_50_ = 4 μM) with a concomitant loss of the activity against the MCF7 cell line (IC_50_ ≥ 20 μM) (Table 2).

**Table 2:**
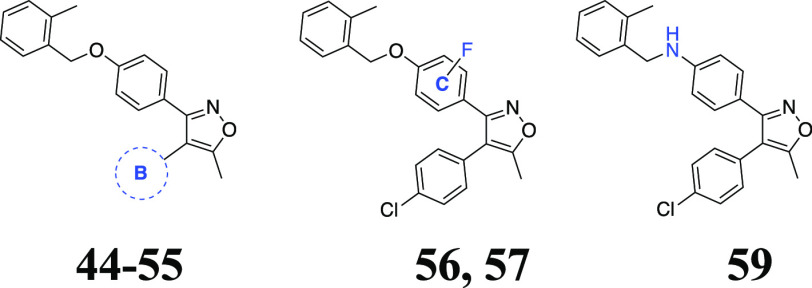

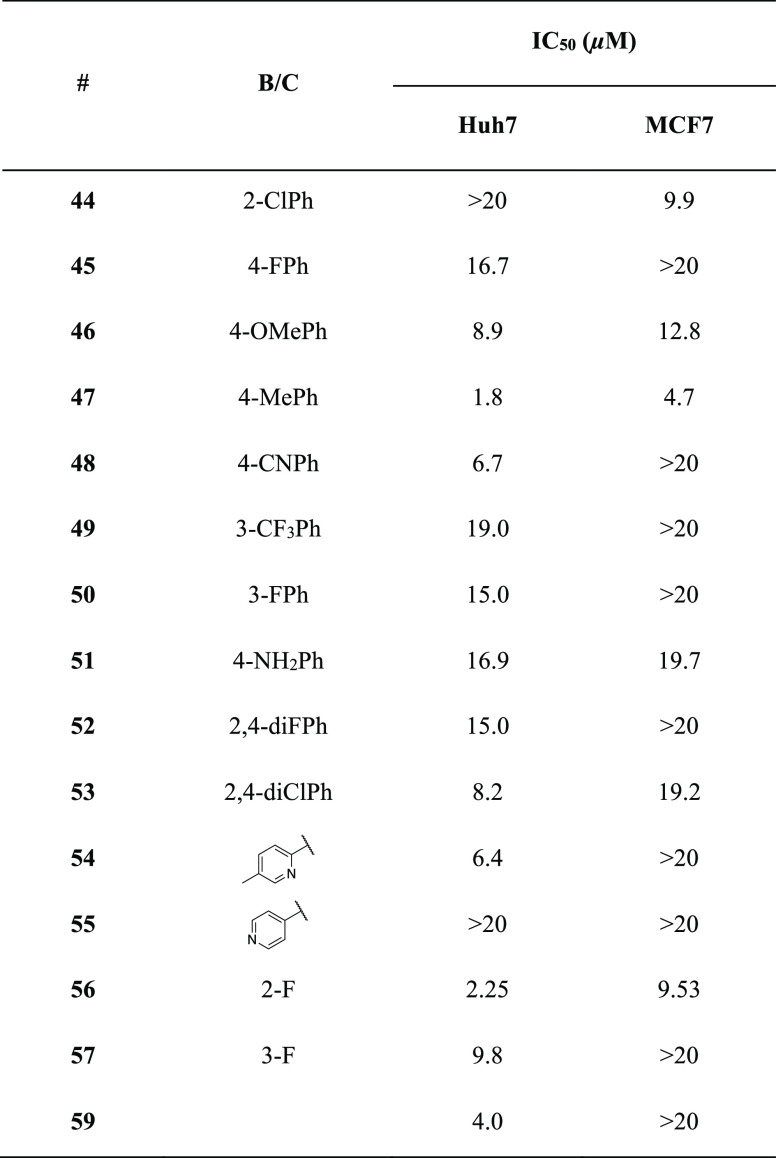
*In Vitro* Cell Growth Inhibitory Activity against Hepatocellular Carcinoma and Breast Cancer Cell Lines^a^

a IC_50_ values were determined at least with five different concentrations of the compounds from the cell growth inhibition percentages.

After the biological confidence was rationalized with 3,4-diarylisoxazole derivatives with respect to C3-benzyloxyphenyl and C4-phenyl pendants, the next area of interest was to explore the central isoxazole with a series of common five-membered heterocycles to broaden the SAR investigation. To this end, eleven heterocyclic congeners with different heteroatom counts and orders were synthesized to probe the effect on the cytotoxic activity against HCC (Huh7 and Mahlavu) and BC (MCF7) cells and to search for the optimal central core for further SAR studies (Table 3). The position exchange of nitrogen and oxygen atoms in **11** to produce **60** resulted in a significant drop of activity against MCF7 cells (IC_50_ ≈ 19 μM), while the potency toward Huh7 cells was still preserved (IC_50_ = 3.8 μM). Furthermore, removal of 3-methyl of isoxazole in **60** to afford **63** appeared to diminish the potency against Mahlavu and MCF7 cells (IC_50_ ≥ 20 μM) but improved the cytotoxic activity for Huh7 (IC_50_ = 1.5 μM). Based on this result, the methyl in **11** was replaced with a polar amino group to further explore the hydrophobic effect and procured **65** with comparable potency to parent **11** against all cell lines with IC_50_ = 2.0-3.9 μM. Interestingly, switching the sites of C3-benzyloxyphenyl unit and C4-phenyl in **11** to afford **67** appeared to diminish the cytotoxic activity for all three cell lines (IC_50_ ≥ 20 μM). Meanwhile, other combinations of heteroatoms such as a group of pyrazoles as well as a thiazole core were also evaluated for their potential to replace the isoxazole core in **11**. While thiazole **75** and pyrazole **78** did not produce the desired activity enhancement, other pyrazole derivatives with different orders of nitrogens and methyl substitutions (**69**, **71**, **72**, **79**, and **85**) maintained adequate cytotoxicity against all cell lines with IC_50_ values in the range of 0.7 to 14.4 μM. Gratifyingly, the reversal of benzyloxyphenyl and 4-chlorophenyl components in **78** to produce **85** displayed a potency boost for Huh7 and MCF7 with IC_50_ values of <1 μM, while still maintaining a decent activity against Mahlavu cells (IC_50_ = 3.7 μM) compared with the parent **11**. Based on the promising potential of **85** as an anticancer agent, the SAR around the middle phenyl and the ether linker was briefly examined, while preserving the 2-methylphenyl and 4-chlorophenyl units, which had been found optimal for the potency (Table 4). The introduction of a nitrogen atom into the middle phenyl in **85** resulted in isosteric pyridine mimics^27^**86** and **87** in which the 2-pyridyl regioisomer (**86**) exhibited higher cytotoxicity toward Huh7 and Mahlavu cells (IC_50_ = 2.0 and 7.1 μM, respectively), although this was accompanied by a reduction in the potency against MCF7 cells (IC_50_ ≥ 20 μM). The bioisosterism between the azine C-N and the aryl C-F bond in compounds **88** and **89** was also examined.^28^ The activity results indicated an efficient bioisosterism of the C-F bond with the pyridine N atom in this context because the replacement of the pyridine N atom with the C-F moiety led to improved potency in **88** (IC_50_ = 1.6–6.4 μM). This was also in good correlation with its isoxazole congener **56** with the 2-F substituted middle phenyl group (Table 2). Lastly, the role of ether linker was studied in **91** and **92**. As seen, while the amide linker was not tolerated for any cell line, the amine replacement of the ether oxygen (**92**) was tolerable for Huh7 and Mahlavu cells (IC_50_ = 3.6 and 4.5 μM, respectively) with a diminished activity against MCF7 cells (Table 4), again in good agreement with its isoxazole counterpart **59** (Table 2). According to the SAR results, compound **11** with 3,4-diarylisoxazole and compound **85** with 1,5-diarylpyrazole frameworks were selected for further analysis, and unambiguous structural elucidation of **11** and **85** using the single-crystal X-ray diffraction method was accomplished showing the accurate rearrangement of aromatic rings and atoms in 3D-shape (Figure S1).^29,30^

**Table 3:**
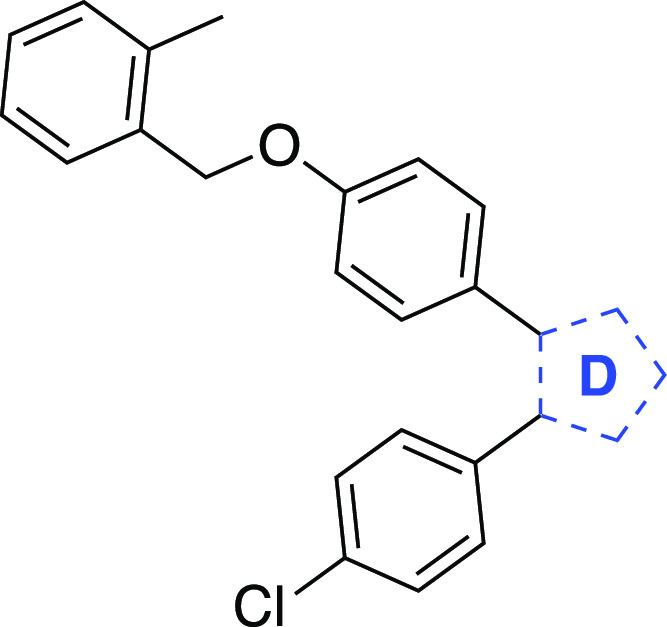

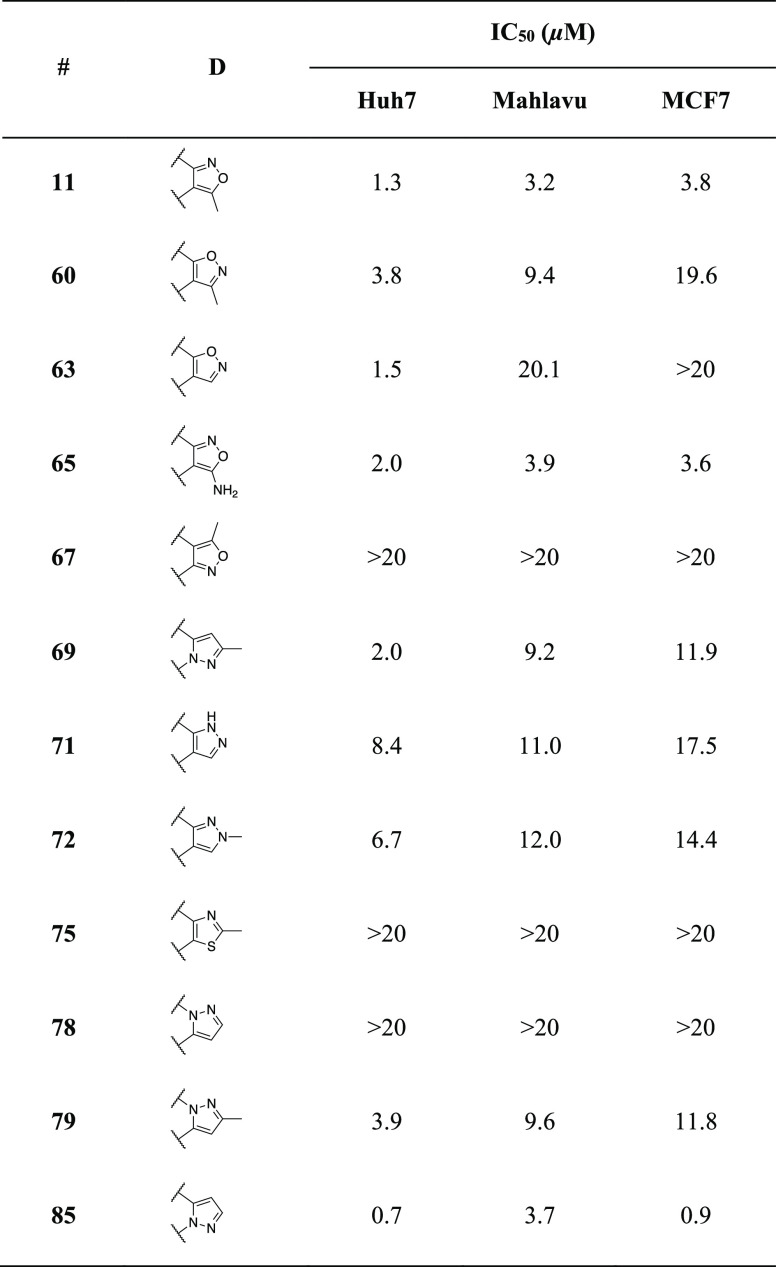
*In Vitro* Cell Growth Inhibitory Activity of Compounds with Distinct Central Heterocycles against Hepatocellular Carcinoma and Breast Cancer Cell Lines^a^

**Table 4:**
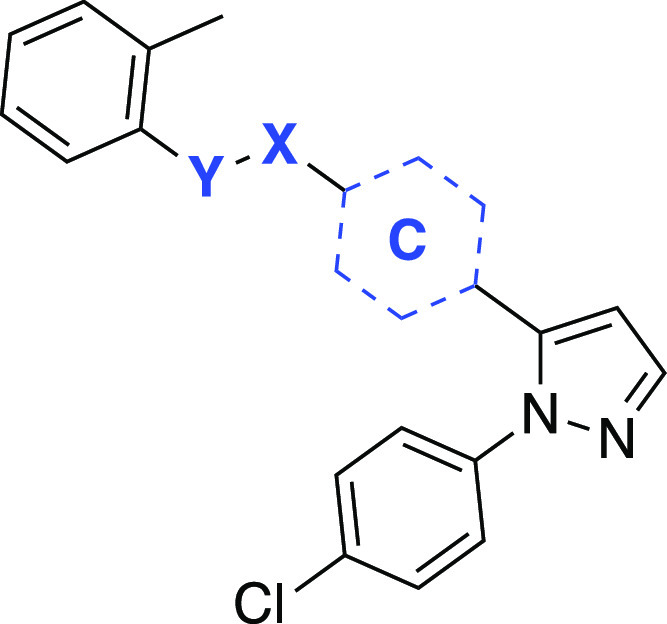

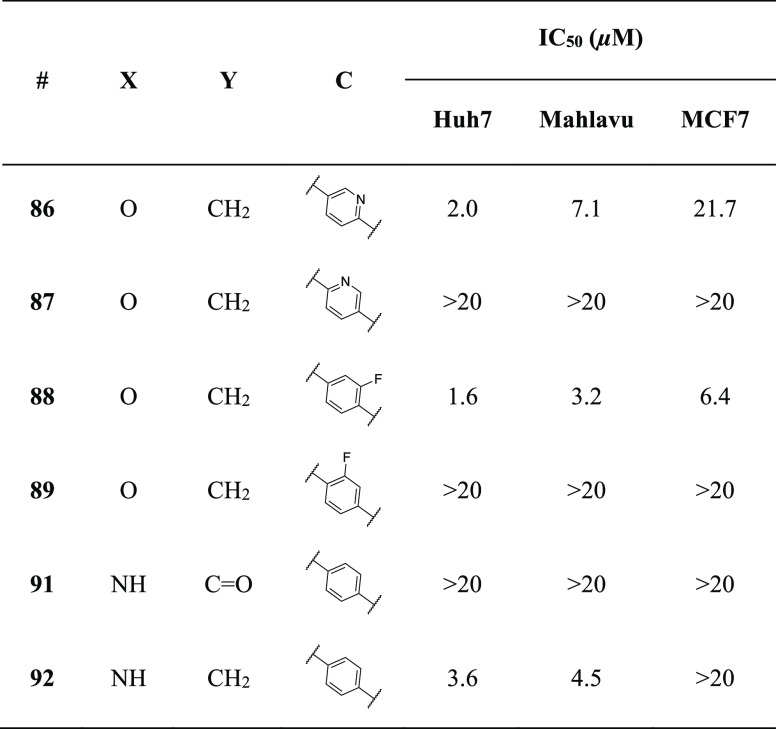
*In Vitro* Growth Cell Inhibitory Activity of 85 Analogues against Hepatocellular Carcinoma and Breast Cancer Cell Lines^a^

Next, judged by the cellular activity of **11** and **85**, both compounds in addition to Huh7, Mahlavu, and MCF7 cell lines were further screened against a panel of the hepatocellular carcinoma (HepG2, SNU475, Hep3B, FOCUS, Hep40, and PLC-PRF-5) and breast cancer (MDA-MB-231, MDA-MB-468, SKBR3, and ZR-75) cell lines along with the non-tumorigenic immortalized breast epithelial cells MCF10A (Table 5). The results demonstrated that both compounds are endowed with potent antiproliferative activity against all cancer cells with IC_50_ values in the range of 1.3–9.5 μM for **11** and 0.77–7.8 μM for **85** for hepatocellular carcinoma and breast cancer cell lines, while found less toxic to the MCF10A immortalized normal breast epithelial cells (Table 5).

**Table 5:** *In Vitro* Growth Inhibitory Activity of 11 and 85 Analogues against Hepatocellular Carcinoma and Breast Cancer Cell Line Panel^a^

		IC_50_ (μM)												
	hepatocellular carcinoma								breast					
#	Huh7	HepG2	SNU475	Hep3B	FOCUS	Hep40	PLC-PRF-5	Mahlavu	MCF7	MDA-MB231	MDA-MB468	SKBR3	ZR75	MCF10A
11	1.3	2.1	1.7	3.0	2.1	8.6	9.5	3.2	3.4	2.0	2.8	3.5	7.6	12.1
85	0.7	1.4	1.5	7.9	2.4	5.2	6.5	3.7	0.9	0.9	1.0	1.8	5.5	7.6

a IC_50_ values were determined at least with five different concentrations of the compounds from the cell growth inhibition percentages.

Moreover, *in vitro* single-dose anticancer screening of **11** and **85** at 10 μM was performed utilizing the stable panel of 60 cell lines comprising 9 different cancer types at the National Cancer Institute (NCI) under the Developmental Therapeutics Program (Table S1).^31^ Compounds **11** and **85** demonstrated variations in sensitivity and selectivity against individual cell lines in the panel, as illustrated in Table S1. Collectively, both compounds exhibited similar antiproliferative activity against 22 cancer cells with growth inhibition (GI) values in the range of 45–100% at 10 μM. The NCI panel results revealed that both compounds displayed a good preference for leukemia such as CCRF-CEM, HL-60, K-562, MOLT-4, and SR, and colon cancer including HCT-116, HCT-15, HT-29, KM-12, and SW-620 cell lines, in addition to hepatocellular carcinoma and breast cancer cells of this work.

Based on the encouraging potency of **85** in cellular assays against hepatocellular carcinoma and breast cancer as well as in the NCI-60 panel, we decided to evaluate *in vitro* ADME and pharmacokinetic (PK) properties in mice (Figure S2). Metabolic stability of **85** in human microsomes was moderate (55% remaining after 45 min incubation). The compound is highly lipophilic with logD_7.4_ of 6.64, sparingly soluble at the physiological pH, and highly plasma protein-bound (99.9%). The capacity of **85** to inhibit human CYP isoforms 2C9, 2D6, and 3A4 was insignificant (7.3, 22.2, and 0% at 10 μM, respectively) implying a safe window for clinical drug interactions.

The PK parameters after intravenous (iv) administration of **85** constitute a moderate volume of distribution, low total plasma clearance, and a moderate iv half-life of 3.85 h resulting in mice with low oral bioavailability (Table 6). Although PK parameters are nonoptimal, this may be counterbalanced by the potent *in vitro* antiproliferative activity spectrum of **85** and is not considered limiting for further progression of **85** to *in vivo* antitumor efficacy studies.

**Table 6:** PK Properties of Compound 85^a^

Mouse IV PK, 1 mpk					Mouse PO PK, 10 mpk			
*t*_1/2_	*V*_d_	*C*_o_	Cl_p_	AUC	*C*_max_	AUC	*t*_1/2_	F%
3.85	13.2	393	2.33	436	19.3	192	6.64	5

a *t*_1/2_ = h, *V*_d_ = (L/kg), *C*_o_, *C*_max_ = (ng/mL), Clp = (L/h/kg), AUC = (h*ng/mL).

### Real-Time Cellular Response of Cells with Compound **11** and **85** Treatment

**Figure 2:**
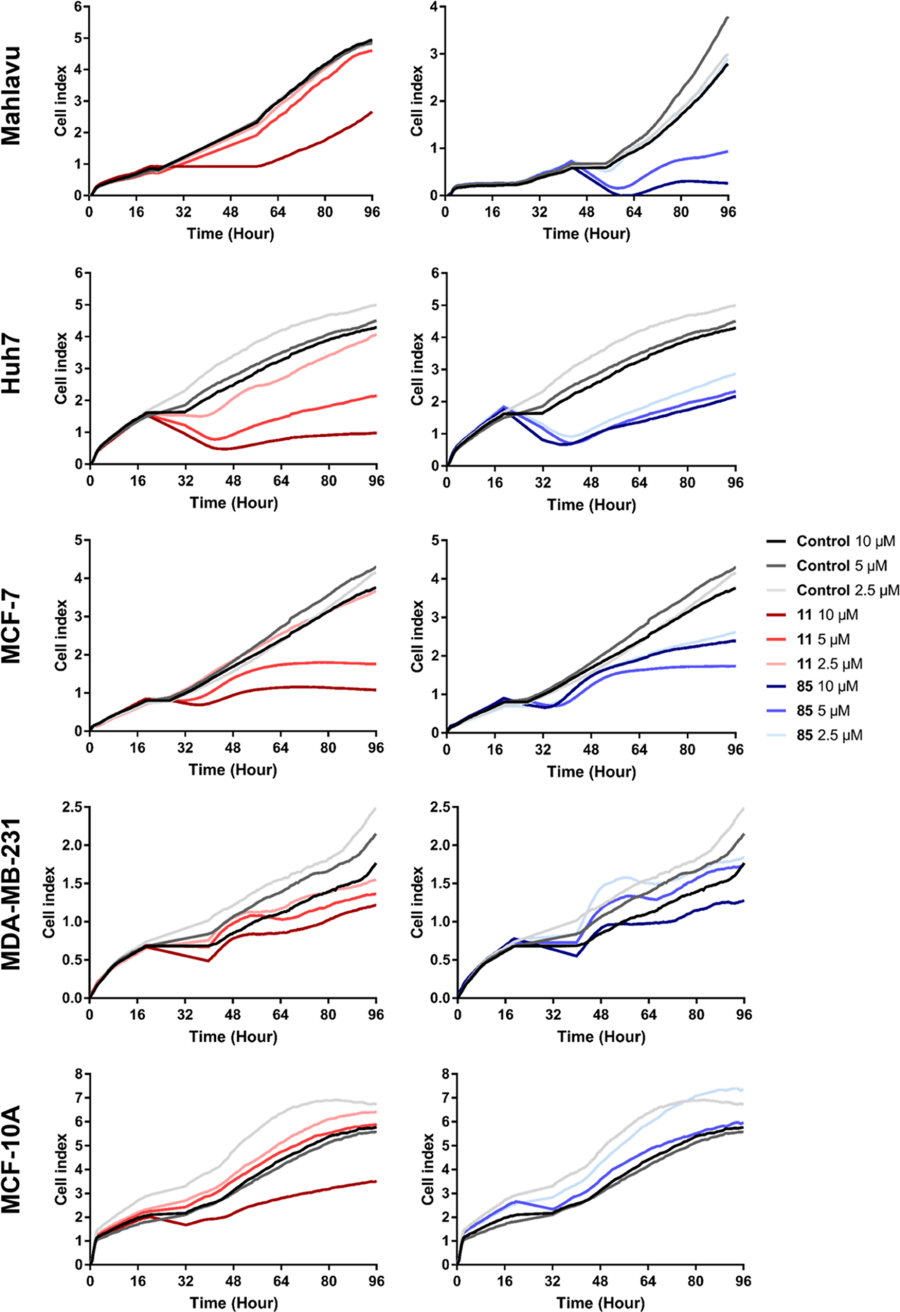
Real-time cell growth response of Mahlavu, Huh7, MCF-7, and MDA-MB-231 cancer cells and MCF10A immortalized normal breast epithelial cells treated with compounds **11** and **85** and control (DMSO) for 96 h. The experiment was done in triplicate, and results were normalized to controls of 10-5-2.5 μM concentrations **11** and **85**.

Time- and dose-dependent effects of compounds **11** and **85** on Mahlavu, Huh7, MDA-MB-231, and MCF-7 were evaluated with the use of real-time cell electronic sensing by monitoring dynamic cell proliferation. Both **11** and **85** caused inhibition in the growth of both breast and hepatocellular carcinoma cell lines compared to the control (DMSO) group, whereas there was no difference in the growth of MCF10A immortalized normal breast epithelial cells upon treatment. This assay further confirmed that both compounds displayed time and dose-dependent growth inhibitory effects. Cytotoxic effects of **11** and **85** on cell lines could be observed after 24 h of compound treatment and reached their highest values after 72 h (Figure 2).

### Characterization of Cell Death Mechanism Induced by **11** and **85**

**Figure 3:**
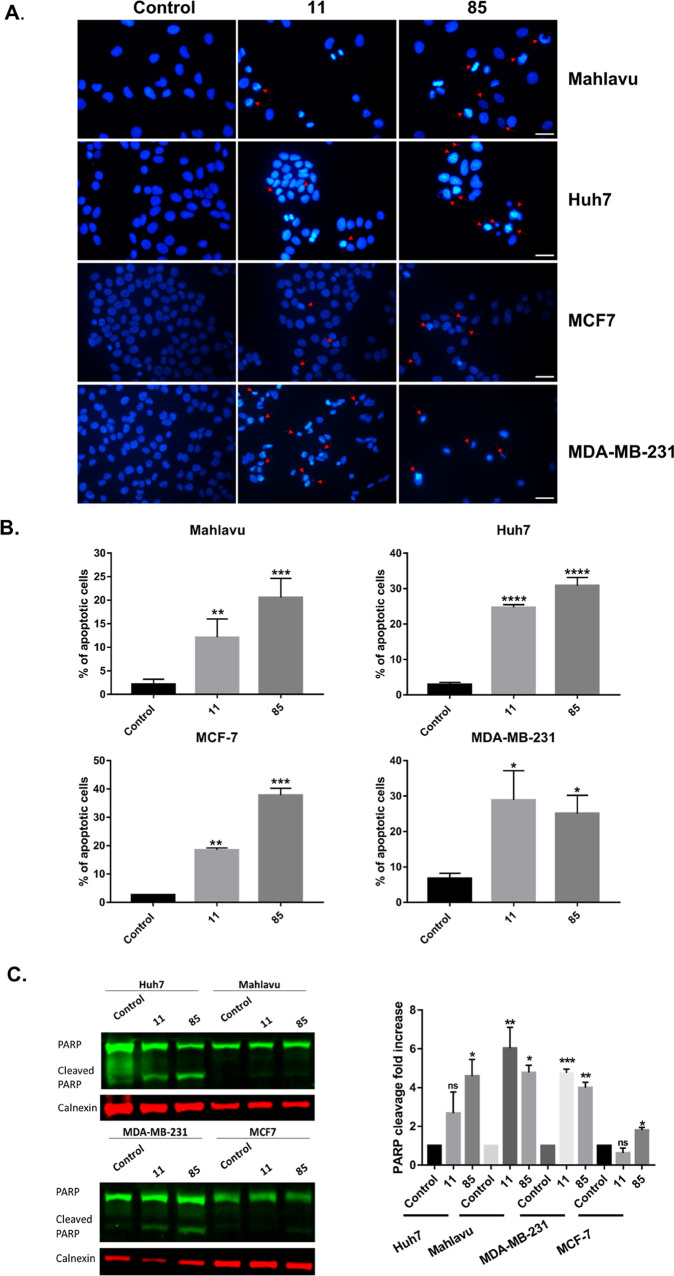
Compounds **11** and **85** induced cell death. (A) Cells were treated with compounds **11** and **85** using their IC_100_ concentrations or DMSO as a negative control for 48 h. Nuclear morphology was revealed by Hoechst staining under fluorescent microscopy. Apoptotic bodies were detectable in **11**- and **85**-treated cells after 48 h. Arrows show apoptotic cells. Scale bar: 20 μM. (B) HCC cells and breast cancer cells were treated with 5 μM compounds **11** and **85** and DMSO as control and were analyzed after 48 h. (C) PARP in Huh7, Mahlavu, MDA-MB-231, and MCF-7 cells treated with **11** and **85** for 48h. Calnexin was used for equal loading control. (D) Bar graph represents relative band intensities of Cleaved PARP/Total PARP, which were normalized with their calnexin loading controls. Statistical analysis was performed using one-way ANOVA. *p < 0.05, **p < 0.01, ***p < 0.001, ns not significant.

To control the cell death mechanism triggered by **11** and **85**, Huh7 and Mahlavu hepatocellular carcinoma cells and MDA-MB-231 and MCF-7 breast cancer cells were cultured according to their cell growth rate and were treated with both **11** and **85** for 48 h. The apoptotic morphological changes were observed in both breast cancer cells and hepatocellular carcinoma cells upon treatment by nuclear staining with Hoechst compared to the control group (Figure 3A). The apoptotic cell populations were further examined with Annexin V staining using flow cytometry. Compared to the control group, the percentage of apoptotic populations in both breast cancer cell lines and hepatocellular carcinoma cell lines treated with **11** or **85** was increased after 24 h (Figure 3B). For further investigation of apoptosis activation through **11** and **85** treatments, apoptosis-associated PARP protein levels were assessed using western blotting. Except for **11** treated MCF7 and Huh7 cells, both **11** and **85** compounds caused the increase in PARP cleavage in both breast cancer cells (MCF7 and MDA-MB-231) and hepatocellular carcinoma cells (Mahlavu) (Figure 3C). These results further supported the increased cytotoxic effects of compounds on breast cancer and hepatocellular carcinoma cancer cells. We next investigated *in vivo* antitumor efficacy of **11** and **85** on nude mice tumor xenografts.

### *In Vivo* Antitumor Effects of **11** and **85** in Mice Xenograft Models

**Figure 4:**
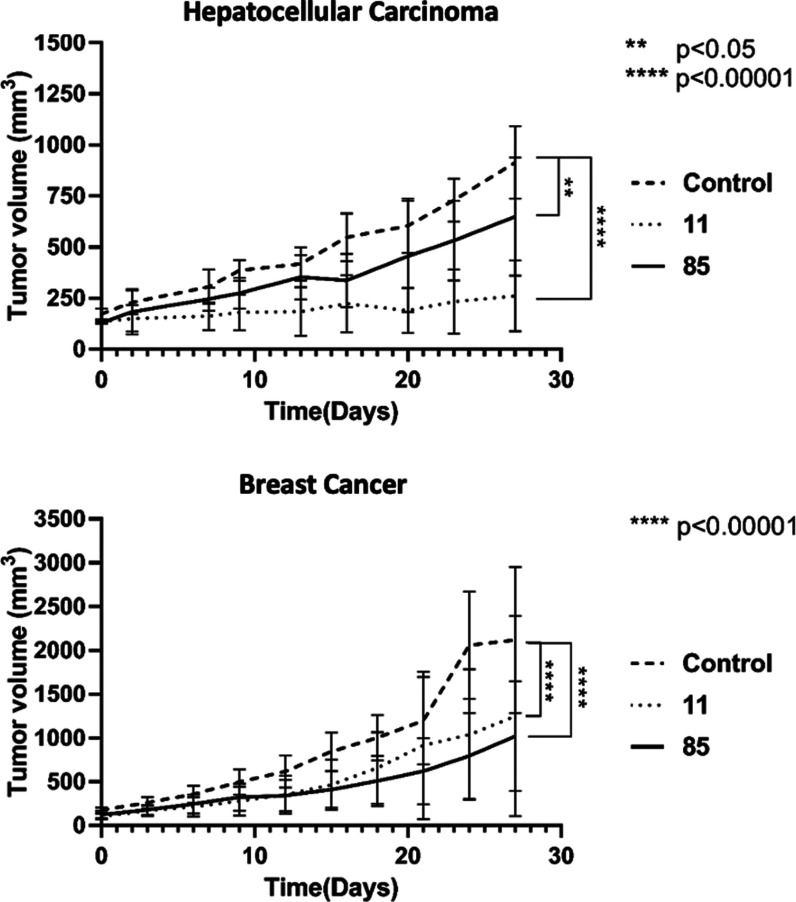
Compounds **11** and **85** reduce tumor growth. Mahlavu and MDA-MB-231 xenograft nude mice were treated with 40 mg/kg **11** and **85** prepared in 0.5% hydroxypropyl methyl cellulose plus 1% Tween 80 or with vehicle only twice a week once the tumor size reached 100 mm^3^. Tumor volumes were recorded twice a week during the experiment. Each group contained six mice (*n* = 6). Statistical analysis was performed using one-way ANOVA. **p < 0.05, ****p < 0.00001.

The antitumor effects of **11** and **85** in the hepatocellular carcinoma (Mahlavu cells) and breast (MDA-MB-231 cells) xenograft models were assessed twice a week by oral administration of **11** and **85** at 40 mg/kg for 4 weeks. Both compounds conferred a sustained antitumor efficiency (Figures 4 and S3). In the Mahlavu xenografts, mice administered with compounds **11** and **85** had a significant reduction in tumor volume following 4 weeks of treatment, i.e., 85 and 40% reductions in tumor volumes, respectively. Moreover, for MDA-MB-231 xenografts, mice treated with both compounds resulted in about a 50% decrease in tumor volumes as compared to the control group (Figure 4). In all studies, the administered dose was well tolerated and neither significant bodyweight loss nor toxic effects or mortality were observed.

## Conclusions

We synthesized a series of vicinal diaryl isoxazole and pyrazole derivatives as putative anticancer agents and checked their growth inhibitory activity against human hepatocellular carcinoma and breast cancer cell lines. Most of the derivatives represented moderate cytotoxicity in the selected cancer cells. In general, the substitution type and pattern on the benzyloxyphenyl group linked to the central heterocycle had a significant effect on the potency and the selectivity of the compounds in the tested cancer cell lines. Following a comprehensive evaluation of twelve central heterocyclics comprising a combination of different heteroatom positions and numbers, we observed that the type of the central heterocycle, i.e., isoxazole and pyrazole, was also crucial for the observed anticancer potency. Subsequently, two analogues, the 3,4-diarylisoxazole derivative **11** and 1,5-diarylpyrazole **85**, stand out as developable anticancer compounds based on their significant *in vitro* antiproliferative activities toward the 13 hepatocellular and breast cancer cell lines with IC_50_ values in the range of 0.77 to 9.53 μM. We also demonstrated that both compounds displayed dose- and time-dependent growth inhibition through the RT-CES system, which was also correlated to initial SRB screening results. Further analysis showed that both compounds induced apoptotic cell death in both hepatocellular carcinoma and breast cancer cell lines and demonstrated antitumor activity *in vivo* in the mouse hepatocellular and breast tumor xenografts with inhibition rates between 40% and 85%, and with insignificant effect on the mice bodyweight.

In conclusion, our results revealed that these diaryl-isoxazole and -pyrazole derivatives exemplified with **11** and **85** show promise as leads for further development of improved anticancer compounds against hepatocellular carcinoma and breast cancers. Hence, further investigation of novel analogues that could be integral to the current SAR in this study would be of value to identify novel diaryl heterocycles with strong anticancer and druglike properties. Elucidation of detailed molecular mechanisms associated with **11** and **85** such as direct profiling of compounds using NanoString analysis as well as target fishing experiments with biotin-labeled conjugates to identify potential molecular targets are under investigation and will be reported in due time.

## Experimental Section

### Chemistry

All chemicals were purchased from different suppliers such as Sigma-Aldrich Chemicals (Sigma-Aldrich Corp., St. Louis, MO), Merck Chemicals (Merck KGaA, Darmstadt, Germany), and ABCR (abcr GmbH, Karlsruhe, Germany). THF was dried from benzophenone-sodium. ^1^H and ^13^C NMR spectra were recorded in CDCl_3_ or DMSO-*d*_6_ on a Varian Mercury 400 MHz spectrometer using tetramethylsilane as the internal standard. Coupling constants were reported as Hertz and all chemical shifts were recorded as δ (ppm). High-resolution mass spectra data (HRMS) were collected using Waters LCT Premier XE Mass Spectrometer operating in ESI(+) or ESI(−) method, also coupled to an AQUITY Ultra Performance Liquid Chromatography system (Waters Corporation) using a UV detector monitoring at 254 nm. Purity for all final compounds was >95%, according to the UPLC-MS method using a water/MeCN solvent gradient containing 0.1% formic acid (1%/90%); flow rate: 0.3 mL/min, column: Aquity BEH C18 column (2.1 × 100 mm^2^, 1.7 mm). All microwave irradiation reactions were carried out in a Biotage Initiator + microwave apparatus with Biotage sealed microwave vials. Flash chromatography was performed on a Combiflash Rf Automatic Flash Chromatography System with RediSep silica gel columns (12, 24, and 40 g) (Teledyne-Isco, Lincoln, NE) or Reveleris PREP Purification System (Buchi, New Castle, DE). Preparative chromatography was performed on Buchi US15C18HQ-250/212-C18 silica gel columns with the following devices; Reveleris PREP Purification System or PuriFlash 4250 System (Interchim, Montluçon, France). Melting points of the compounds were determined using the SMP50 automated melting point apparatus (Stuart, Staffordshire, ST15 OSA, U.K.). The *in vitro* ADME and *in vivo* mouse PK studies were conducted at Syngene International Ltd., Bangalore, India. General synthetic procedures (Methods 1–9) and experimental data for all intermediate compounds can be found in the Supporting Information.

#### 3-(4-(Benzyloxy)phenyl)-4-(4-chlorophenyl)-5-methylisoxazole (**9**)

It was synthesized according to method 4 by the reaction of 4-chlorophenylacetone and the 1-(benzyloxy)-4-(nitrosomethyl)benzene unstable intermediate obtained in situ following the literature as shown in Scheme S1.^21^ The resulting crude product was purified by flash column chromatography (0% → 40% EtOAc in Hexane). Yield 50.0%; mp 114.7–115.7 °C. ^1^H NMR (400 MHz, CDCl_3_): δ_H_ 2.42 (3H, s), 5.06 (2H, s), 6.93 (2H, d, *J* = 8.8 Hz), 7.12 (2H, d, *J* = 8.8 Hz), 7.32–7.43 (9H, m). ^13^C NMR (100 MHz, CDCl_3_): δ_C_ 11.56, 69.99, 114.53, 114.91, 121.41, 127.49, 128.61, 129.01, 129.09, 129.75, 131.11, 133.69, 136.58, 159.78, 160.60, 166.54. HRMS (*m*/*z*) [M + H]^+^ calcd for C_23_H_19_ClNO_2_: 376.1104, found: 376.1106.

#### 4-(4-Chlorophenyl)-5-methyl-3-(4-((2-methylbenzyl)oxy)phenyl)isoxazole (**11**)

It was synthesized from compound **10** using 2-methylbenzyl bromide according to synthesis method 1a. Yield 77.0%; mp 114.1–115.5 °C. ^1^H NMR (400 MHz, CDCl_3_): δ_H_ 2.37 (3H, s), 2.43 (3H, s), 5.03 (2H, s), 6.94 (2H, d, *J* = 8.4 Hz), 7.13 (2H, d, *J* = 8.8 Hz), 7.19–7.28 (3H, m), 7.35–7.40 (5H, m). ^13^C NMR (100 MHz, CDCl_3_): δ_C_ 11.62, 18.89, 68.60, 114.57, 114.84, 121.41, 126.06, 128.39, 128.63, 129.02, 129.10, 129.78, 130.44, 131.15, 133.71, 134.39, 136.69, 159.91, 160.62, 166.58. HRMS (*m*/*z*) [M + H]^+^ calcd for C_24_H_21_NO_2_Cl: 390.1261, found: 390.1263. CAS: 2758520-84-0.

#### 4-(4-Chlorophenyl)-3-(4-((2-methoxybenzyl)oxy)phenyl)-5-methylisoxazole (**12**)

It was synthesized from compound **10** using 2-methoxybenzyl chloride according to synthesis method 1a. The resulting crude product was purified by flash column chromatography (0% → 30% EtOAc in Hexane). Yield 75.0%; mp 156.8–157.7 °C. ^1^H NMR (400 MHz, CDCl_3_): δ_H_ 2.42 (3H, s), 3.85 (3H, s), 5.11 (2H, s), 6.89-6.99 (4H, m), 7.12 (2H, d, *J* = 8.4 Hz), 7.28–7.32 (1H, m), 7.33–7.36 (4H, m), 7.43 (1H, d, *J* = 7.6 Hz). ^13^C NMR (100 MHz, CDCl_3_): δ_C_ 11.56, 55.37, 65.06, 110.27, 114.53, 114.95, 120.58, 121.17, 124.94, 128.59, 128.99, 129.04, 129.15, 129.68, 131.12, 133.67, 156.81, 160.00, 160.68, 166.48. HRMS (*m*/*z*) [M + H]^+^ calcd for C_24_H_21_NO_3_Cl: 406.1210, found: 406.1212.

#### 4-(4-Chlorophenyl)-5-methyl-3-(4-((2-(trifluoromethoxy)benzyl)oxy)phenyl)isoxazole (**13**)

It was synthesized from compound **10** using 2-trifluoromethoxybenzyl bromide according to synthesis method 1a. The resulting crude product was purified by flash column chromatography (0% → 30% EtOAc in Hexane). Yield 46.0%; mp 85.8–86.7 °C. ^1^H NMR (400 MHz, DMSO-*d*_6_): δ_H_ 2.39 (3H, s), 5.13 (2H, s), 7.04 (2H, d, *J* = 8.8 Hz), 7.23 (2H, d, *J* = 8.4 Hz), 7.28 (2H, d, *J* = 8.8 Hz), 7.40–7.52 (5H, m), 7.64 (1H, d, *J* = 7.6 Hz). ^13^C NMR (100 MHz, DMSO-*d*_6_): δ_C_ 11.20, 64.22, 113.96, 114.91, 120.12 (q, ^1^*J*_C-F_ = 255.2 Hz), 120.57, 121.14, 127.56, 128.79, 128.83, 128.97, 129.48, 130.29, 130.88, 131.41, 132.56, 146.65, 159.11, 160.03, 166.94. HRMS (*m*/*z*) [M + H]^+^ calcd for C_24_H_18_NO_3_ClF_3_: 460.0927, found: 460.0928.

#### 4-(4-Chlorophenyl)-3-(4-((2-(difluoromethoxy)benzyl)oxy)phenyl)-5-methylisoxazole (**14**)

It was synthesized from compound **10** using 2-difluoromethoxybenzyl chloride according to synthesis method 1a. The resulting crude product was purified by flash column chromatography (0% → 30% EtOAc in Hexane). Yield 86.0%; mp 95.5–96.9 °C. ^1^H NMR (400 MHz, DMSO-*d*_6_): δ_H_ 2.42 (3H, s), 5.12 (2H, s), 7.06 (2H, d, *J* = 8.8 Hz), 7.24–7.27 (5H, m), 7.70 (2H, d, *J* = 8.8 Hz), 7.42–7.47 (1H, m), 7.49 (2H, d, *J* = 8.4. Hz), 7.58 (1H, dd, *J* = 7.6 Hz, 1.2 Hz). ^13^C NMR (100 MHz, DMSO-*d*_6_): δ_C_ 11.20, 64.44, 113.95, 114.90, 116.60 (t, ^1^*J*_C-F_ = 256.5 Hz), 118.52, 121.01, 125.40, 127.56, 128.81, 128.84, 129.47, 129.89, 130.37, 131.40, 132.55, 149.13 (t, ^3^*J*_C-F_ = 3.2 Hz), 159.24, 160.05, 166.92. HRMS (*m*/*z*) [M + H]^+^ calcd for C_24_H_19_NO_3_ClF_2_: 442.1022, found: 442.1016.

#### 4-(4-Chlorophenyl)-3-(4-((2-fluorobenzyl)oxy)phenyl)-5-methylisoxazole (**15**)

It was synthesized from compound **10** using 2-fluorobenzyl chloride according to synthesis method 1a. The resulting crude product was purified by flash column chromatography (0% → 30% EtOAc in Hexane). Yield 91.0%; mp 119.5–120.9 °C. ^1^H NMR (400 MHz, CDCl_3_): δ_H_ 2.42 (3H, s), 5.13 (2H, s), 6.94 (2H, d, *J* = 8.4 Hz), 7.06–7.08 (1H, m), 7.12 (2H, d, *J* = 8.8 Hz), 7.16 (1H, td, *J* = 7.6 Hz, 1.2 Hz), 7.29–7.33 (1H, m), 7.35 (2H, d, *J* = 8.4 Hz), 7.36 (2H, d, *J* = 8.8 Hz), 7.49 (1H, td, *J* = 7.4, 1.6 Hz). ^13^C NMR (100 MHz, CDCl_3_): δ_C_ 11.56, 63.68 (d, ^3^*J*_C-F_ = 3.8 Hz), 114.54, 114.86, 115.38 (d, ^2^*J*_C-F_ = 20.6 Hz), 121.63, 123.76 (d, ^2^*J*_C-F_ = 13.7 Hz), 124.28 (d, ^3^*J*_C-F_ = 3.1 Hz), 129.01, 129.06, 129.69 (d, ^3^*J*_C-F_ = 3.8 Hz), 129.79, 129.87, 131.09, 133.71, 159.52, 160.44 (d, ^1^*J*_C-F_ = 245.4 Hz), 160.58, 166.55. HRMS (*m*/*z*) [M + H]^+^ calcd for C_23_H_18_NO_2_ClF: 394.1010, found: 394.1008.

#### 4-(4-Chlorophenyl)-3-(4-((2-chlorobenzyl)oxy)phenyl)-5-methylisoxazole (**16**)

It was synthesized from compound **10** using 2-chlorobenzyl chloride according to synthesis method 1a. The resulting crude product was purified by flash column chromatography (0% → 30% EtOAc in Hexane). Yield 91.0%; mp 105.2–106.4 °C. ^1^H NMR (400 MHz, CDCl_3_): δ_H_ 2.43 (3H, s), 5.17 (2H, s), 6.95 (2H, d, *J* = 9.2 Hz), 7.13 (2H, d, *J* = 8.8 Hz), 7.27–7.32 (2H, m), 7.35–7.41 (5H, m), 7.53–7.55 (1H, m). ^13^C NMR (100 MHz, CDCl_3_): δ_C_ 11.56, 67.11, 114.54, 114.93, 121.70, 126.98, 128.74, 129.03, 129.07, 129.08, 129.41, 129.81, 131.11, 132.58, 133.74, 134.32, 159.49, 160.56, 166.58. HRMS (*m*/*z*) [M + H]^+^ calcd for C_23_H_18_NO_2_Cl_2_: 410.0715, found: 410.0717.

#### 2-((4-(4-(4-Chlorophenyl)-5-methylisoxazol-3-yl)phenoxy)methyl)benzonitrile (**17**)

It was synthesized from compound **10** using α-bromo-o-tolunitrile according to synthesis method 1a. The resulting crude product was purified by flash column chromatography (0% → 30% EtOAc in Hexane). Yield 77.0%; mp 111.2–112.5 °C. ^1^H NMR (400 MHz, CDCl_3_): δ_H_ 2.42 (3H, s), 5.26 (2H, s), 6.95 (2H, d, *J* = 8.8 Hz), 7.12 (2H, d, *J* = 8.8 Hz), 7.35–7.39 (4H, m), 7.44 (1H, td, *J* = 7.4, 1.6 Hz), 7.61–7.71 (3H, m). ^13^C NMR (100 MHz, CDCl_3_): δ_C_ 11.56, 67.51, 111.17, 114.57, 114.96, 116.96, 122.14, 128.44, 128.52, 128.97, 129.05, 129.88, 131.09, 132.91, 133.09, 133.77, 140.16, 159.09, 160.48, 166.61. HRMS (*m*/*z*) [M + H]^+^ calcd for C_24_H_18_N_2_O_2_Cl: 401.1057, found: 401.1054.

#### 4-(4-Chlorophenyl)-5-methyl-3-(4-((2-(trifluoromethyl)benzyl)oxy)phenyl)isoxazole (**18**)

It was synthesized from compound **10** using 2-trifluoromethylbenzyl bromide according to synthesis method 1a. The resulting crude product was purified by flash column chromatography (0% → 30% EtOAc in Hexane). Yield 70.0%; mp 132.8–134.4 °C. ^1^H NMR (400 MHz, DMSO-*d*_6_): δ_H_ 2.42 (3H, s), 5.24 (2H, s), 7.06 (2H, d, *J* = 8.4 Hz), 7.26 (2H, d, *J* = 8.2 Hz), 7.32 (2H, d, *J* = 8.4 Hz), 7.49 (2H, d, *J* = 8.2 Hz), 7.60 (1H, t, *J* = 7.6 Hz), 7.73 (1H, dd, *J* = 7.6, 7.2 Hz), 7.77 (1H, d, *J* = 7.2 Hz), 7.80 (1H, d, *J* = 7.6 Hz). ^13^C NMR (100 MHz, DMSO-*d*_6_): δ_C_ 11.20, 66.26 (q, ^4^*J*_C-F_ = 2.6 Hz), 113.96, 114.92, 121.23, 124.24 (q, ^1^*J*_C-F_ = 271.9 Hz), 126.13 (q, ^3^*J*_C-F_ = 5.8 Hz), 126.96 (q, ^2^*J*_C-F_ = 30.1 Hz), 128.79, 128.84, 129.52, 130.66, 131.41, 132.56, 132.80, 134.49 (q, ^4^*J*_C-F_ = 1.3 Hz), 159.03, 160.01, 166.96. HRMS (*m*/*z*) [M + H]^+^ calcd for C_24_H_18_NO_2_ClF_3_: 444.0978, found: 444.0973.

#### 4-(4-Chlorophenyl)-5-methyl-3-(4-((3-methylbenzyl)oxy)phenyl)isoxazole (**19**)

It was synthesized from compound **10** using 3-methylbenzyl bromide according to synthesis method 1a. The resulting crude product was purified by flash column chromatography (0% → 30% EtOAc in Hexane). Yield 90.0%; mp 119.9–121.0 °C. ^1^H NMR (400 MHz, CDCl_3_): δ_H_ 2.37 (3H, s), 2.42 (3H, s), 5.02 (2H, s), 6.93 (2H, d, *J* = 8.8 Hz), 7.12 (2H, d, *J* = 8.4 Hz), 7.13–7.15 (1H, m), 7.20–7.29 (3H, m), 7.34–7.36 (4H, m). ^13^C NMR (100 MHz, CDCl_3_): δ_C_ 11.56, 21.40, 70.07, 114.53, 114.91, 121.36, 124.61, 128.25, 128.52, 128.85, 129.01, 129.11, 129.74, 131.11, 133.69, 136.47, 138.33, 159.85, 160.62, 166.52. HRMS (*m*/*z*) [M + H]^+^ calcd for C_24_H_21_NO_2_Cl: 390.1261, found: 390.1260.

#### 4-(4-Chlorophenyl)-3-(4-((3-methoxybenzyl)oxy)phenyl)-5-methylisoxazole (**20**)

It was synthesized from compound **10** using 3-methoxybenzyl chloride according to synthesis method 1a. The resulting crude product was purified by flash column chromatography (0% → 30% EtOAc in Hexane). Yield 95.0%; mp 105.1–106.8 °C. ^1^H NMR (400 MHz, CDCl_3_): δ_H_ 2.42 (3H, s), 3.81 (3H, s), 5.04 (2H, s), 6.85-6.88 (1H, m), 6.92 (2H, d, *J* = 8.8 Hz), 6.97-6.99 (2H, m), 7.11 (2H, d, *J* = 8.4 Hz), 7.29 (1H, t, *J* = 8.0 Hz), 7.34–7.37 (4H, m). ^13^C NMR (100 MHz, CDCl_3_): δ_C_ 11.56, 55.24, 69.86, 112.91, 113.56, 114.53, 114.92, 119.62, 121.44, 129.01, 129.09, 129.67, 129.74, 131.11, 133.70, 138.17, 159.73, 159.85, 160.59, 166.53. HRMS (*m*/*z*) [M + H]^+^ calcd for C_24_H_21_NO_3_Cl: 406.1210, found: 406.1191.

#### 4-(4-Chlorophenyl)-3-(4-((3-fluorobenzyl)oxy)phenyl)-5-methylisoxazole (**21**)

It was synthesized from compound **10** using 3-fluorobenzyl chloride according to synthesis method 1a. The resulting crude product was purified by flash column chromatography (0% → 30% EtOAc in Hexane). Yield 90.0%; mp 98.1–99.8 °C. ^1^H NMR (400 MHz, CDCl_3_): δ_H_ 2.42 (3H, s), 5.06 (2H, s), 6.91 (2H, d, *J* = 8.8 Hz), 6.98–7.03 (1H, m), 7.11 (2H, d, *J* = 8.4 Hz), 7.13–7.19 (2H, m), 7.32–7.36 (5H, m). ^13^C NMR (100 MHz, CDCl_3_): δ_C_ 11.56, 69.14 (d, ^4^*J*_C-F_ = 2.0 Hz), 114.22 (d, ^2^*J*_C-F_ = 22.5 Hz), 114.53, 114.89, 114.91 (d, ^2^*J*_C-F_ = 21.1 Hz), 121.69, 122.69 (d, ^4^*J*_C-F_ = 3.2 Hz), 129.02, 129.05, 129.79, 130.16 (d, ^3^*J*_C-F_ = 8.3 Hz), 131.09, 133.72, 139.19 (d, ^3^*J*_C-F_ = 7.0 Hz), 159.46, 160.53, 162.98 (d, ^1^*J*_C-F_ = 244.9 Hz), 166.58. HRMS (*m*/*z*) [M + H]^+^ calcd for C_23_H_18_NO_2_ClF: 394.1010, found: 394.1011.

#### 3-((4-(4-(4-Chlorophenyl)-5-methylisoxazol-3-yl)phenoxy)methyl)benzonitrile (**22**)

It was synthesized from compound **10** using α-bromo-m-tolunitrile according to synthesis method 1a. The resulting crude product was purified by flash column chromatography (0% → 50% EtOAc in Hexane). Yield 97.0%; mp 114.4–115.6 °C. ^1^H NMR (400 MHz, CDCl_3_): δ_H_ 2.42 (3H, s), 5.08 (2H, s), 6.91 (2H, d, *J* = 9.2 Hz), 7.11 (2H, d, *J* = 8.4 Hz), 7.36 (2H, d, *J* = 8.4 Hz), 7.37 (2H, d, *J* = 9.2 Hz), 7.50 (1H, dd, *J* = 8.0, 7.2 Hz), 7.61–7.66 (2H, m), 7.73 (1H, s). ^13^C NMR (100 MHz, CDCl_3_): δ_C_ 11.56, 68.64, 112.83, 114.53, 114.84, 118.55, 122.04, 128.99, 129.04, 129.43, 129.89, 130.68, 131.09, 131.44, 131.66, 133.76, 138.26, 159.13, 160.44, 166.65. HRMS (*m*/*z*) [M + H]^+^ calcd for C_24_H_18_N_2_O_2_Cl: 401.1057, found: 401.1059.

#### Methyl-3-((4-(4-(4-chlorophenyl)-5-methylisoxazol-3-yl)phenoxy)methyl)benzoate (**23**)

It was synthesized from compound **10** using methyl-3-(bromomethyl) benzoate according to synthesis method 1a. The resulting crude product was purified by flash column chromatography (0% → 40% EtOAc in Hexane). Yield 93.0%; mp 121.5–122.8 °C. ^1^H NMR (400 MHz, CDCl_3_): δ_H_ 2.42 (3H, s), 3.92 (3H, s), 5.10 (2H, s), 6.92 (2H, d, *J* = 8.4 Hz), 7.11 (2H, d, *J* = 8.4 Hz), 7.33–7.37 (4H, m), 7.46 (1H, t, *J* = 7.8 Hz), 7.62 (1H, d, *J* = 7.8 Hz), 8.00 (1H, d, *J* = 7.8 Hz), 8.09 (1H, s). ^13^C NMR (100 MHz, CDCl_3_): δ_C_ 11.56, 52.20, 69.42, 114.53, 114.91, 121.66, 128.53, 128.75, 129.01, 129.04, 129.26, 129.80, 130.55, 131.09, 131.84, 133.71, 137.06, 159.52, 160.55, 166.57, 166.79. HRMS (*m*/*z*) [M + H]^+^ calcd for C_25_H_21_NO_4_Cl: 434.1159, found: 434.1165.

#### 4-(4-Chlorophenyl)-5-methyl-3-(4-((4-methylbenzyl)oxy)phenyl)isoxazole (**24**)

It was synthesized from compound **10** using 4-methylbenzyl bromide according to synthesis method 1a. The resulting crude product was purified by flash column chromatography (0% → 30% EtOAc in Hexane). Yield 82.0%; mp 116.9–118.5 °C. ^1^H NMR (400 MHz, CDCl_3_): δ_H_ 2.36 (3H, s), 2.42 (3H, s), 5.02 (2H, s), 6.92 (2H, d, *J* = 9.2 Hz), 7.12 (2H, d, *J* = 8.8 Hz), 7.20 (2H, d, *J* = 7.8 Hz), 7.30 (2H, d, *J* = 7.8 Hz), 7.33–7.37 (4H, m). ^13^C NMR (100 MHz, CDCl_3_): δ_C_ 11.56, 21.19, 69.94, 114.53, 114.92, 121.32, 127.63, 128.99, 129.11, 129.29, 129.72, 131.11, 133.53, 133.68, 137.89, 159.86, 160.62, 166.51. HRMS (*m*/*z*) [M + H]^+^ calcd for C_24_H_21_NO_2_Cl: 390.1261, found: 390.1259.

#### 4-(4-Chlorophenyl)-3-(4-((4-methoxybenzyl)oxy)phenyl)-5-methylisoxazole (**25**)

It was synthesized from compound **10** using 4-methoxybenzyl chloride according to synthesis method 1a. The resulting crude product was purified by flash column chromatography (0% → 30% EtOAc in Hexane). Yield 93.0%; mp 126.1–127.4 °C. ^1^H NMR (400 MHz, CDCl_3_): δ_H_ 2.42 (3H, s), 3.81 (3H, s), 4.98 (2H, s), 6.91 (4H, m), 7.12 (2H, d, *J* = 8.8 Hz), 7.33–7.37 (6H, m). ^13^C NMR (100 MHz, CDCl_3_): δ_C_ 11.56, 55.29, 69.80, 114.03,114.53, 114.91, 121.31, 128.59, 128.99, 129.11, 129.26, 129.72, 131.11, 133.68, 159.55, 159.84, 160.62, 166.51. HRMS (*m*/*z*) [M + H]^+^ calcd for C_24_H_21_NO_3_Cl: 406.1210, found: 406.1226.

#### 4-(4-Chlorophenyl)-3-(4-((4-fluorobenzyl)oxy)phenyl)-5-methylisoxazole (**26**)

It was synthesized from compound **10** using 4-fluorobenzyl chloride according to synthesis method 1a. The resulting crude product was purified by flash column chromatography (0% → 30% EtOAc in Hexane). Yield 88.0%; mp 99.3–100.7 °C. ^1^H NMR (400 MHz, CDCl_3_): δ_H_ 2.42 (3H, s), 5.01 (2H, s), 6.91 (2H, d, *J* = 8.8 Hz), 7.06 (2H, t, *J* = 8.8 Hz), 7.11 (2H, d, *J* = 8.8 Hz), 7.34–7.41 (6H, m). ^13^C NMR (100 MHz, CDCl_3_): δ_C_ 11.55, 69.32, 114.53, 114.88, 115.53 (d, ^2^*J*_C-F_ = 21.3 Hz), 121.59, 129.01, 129.07, 129.35 (d, ^3^*J*_C-F_ = 7.6 Hz), 129.78, 131.10, 132.33 (d, ^4^*J*_C-F_ = 3.8 Hz), 133.71, 159.59, 160.55, 162.56 (d, ^1^*J*_C-F_ = 244.6 Hz), 166.57. HRMS (*m*/*z*) [M + H]^+^ calcd for C_23_H_18_NO_2_ClF: 394.1010, found: 394.1009.

#### 4-((4-(4-(4-Chlorophenyl)-5-methylisoxazol-3-yl)phenoxy)methyl)benzonitrile (**27**)

It was synthesized from compound **10** using α-bromo-p-tolunitrile according to synthesis method 1a. The resulting crude product was purified by flash column chromatography (0% → 50% EtOAc in Hexane). Yield 77.0%; mp 138.5–139.5 °C. ^1^H NMR (400 MHz, CDCl_3_): δ_H_ 2.42 (3H, s), 5.12 (2H, s), 6.90 (2H, d, *J* = 8.8 Hz), 7.11 (2H, d, *J* = 8.4 Hz), 7.34–7.37 (4H, m), 7.53 (2H, d, *J* = 8.2 Hz), 7.68 (2H, d, *J* = 8.2 Hz). ^13^C NMR (100 MHz, CDCl_3_): δ_C_ 11.55, 68.87, 111.86, 114.52, 114.84, 118.60, 122.04, 127.54, 128.99, 129.03, 129.87, 131.09, 132.43, 133.76, 142.03, 159.14, 160.42, 166.66. C_24_H_18_N_2_O_2_Cl for; HRMS (*m*/*z*) [M + H]^+^ calcd for 401.1057, found:401.1070.

#### Methyl-4-((4-(4-(4-chlorophenyl)-5-methylisoxazol-3-yl)phenoxy)methyl)benzoate (**28**)

It was synthesized from compound **10** using methyl-4-(bromomethyl) benzoate according to synthesis method 1a. The resulting crude product was purified by flash column chromatography (0% → 40% EtOAc in Hexane). Yield 86.0%; mp 110.7–111.9 °C. ^1^H NMR (400 MHz, CDCl_3_): δ_H_ 2.42 (3H, s), 3.92 (3H, s), 5.12 (2H, s), 6.92 (2H, d, *J* = 9.2 Hz), 7.11 (2H, d, *J* = 8.8 Hz), 7.34–7.37 (4H, m), 7.49 (2H, d, *J* = 8.4 Hz), 8.06 (2H, d, *J* = 8.4 Hz). ^13^C NMR (100 MHz, CDCl_3_): δ_C_ 11.56, 52.15, 69.32, 114.53, 114.91, 121.74, 126.97, 129.02, 129.04, 129.81, 129.90, 131.10, 133.73, 141.75, 159.45, 160.51, 166.59, 166.77. HRMS (*m*/*z*) [M + H]^+^ calcd for C_25_H_21_NO_4_Cl: 434.1159, found: 434.1161.

#### 4-((4-(4-(4-Chlorophenyl)-5-methylisoxazol-3-yl)phenoxy)methyl)benzoic acid (**29**)

Compound **28** (1.0 equiv) was stirred with LiOH.H_2_O (3.0 eq) in a THF:water solvent system under reflux for 3 h. At the end of the period, the reaction mixture was acidified with concentrated HCl and the precipitate formed was filtered under vacuum by washing with water. Yield 89.0%; mp 210.8–212.4 °C. ^1^H NMR (400 MHz, CDCl_3_): δ_H_ 2.43 (3H, s), 5.15 (2H, s), 6.93 (2H, d, *J* = 8.4 Hz), 7.12 (2H, d, *J* = 8.8 Hz), 7.35–7.38 (4H, m), 7.53 (2H, d, *J* = 8.8 Hz), 8.14 (2H, d, *J* = 8.0 Hz). ^13^C NMR (100 MHz, CDCl_3_): δ_C_ 11.56, 69.26, 114.56, 114.91, 121.77, 127.03, 128.91, 129.04, 129.84, 130.55, 131.11, 133.75, 142.77, 159.41, 160.50, 166.63, 171.42. HRMS (*m*/*z*) [M + H]^+^ calcd for C_24_H_19_NO_4_Cl: 420.1003, found: 420.1005.

#### 4-(4-Chlorophenyl)-3-(4-((2,3-dimethylbenzyl)oxy)phenyl)-5-methylisoxazole (**30**)

It was synthesized from compound **10** using 2,3-dimethylbenzyl bromide according to synthesis method 1a. The resulting crude product was purified by flash column chromatography (0% → 40% EtOAc in Hexane). Yield 87.0%; mp 167.2–168.5 °C. ^1^H NMR (400 MHz, CDCl_3_): δ_H_ 2.26 (3H, s), 2.32 (3H, s), 2.43 (3H, s), 5.04 (2H, s), 6.94 (2H, d, *J* = 8.4 Hz), 7.10–7.18 (4H, m), 7.24 (1H, d, *J* = 6.8 Hz), 7.35–7.38 (4H, m). ^13^C NMR (100 MHz, CDCl_3_): δ_C_ 11.56, 14.88, 20.34, 69.22, 114.53, 114.83, 121.36, 125.58, 126.87, 129.01, 129.12, 129.74, 130.21, 131.13, 133.70, 134.22, 135.50, 137.29, 159.94, 160.62, 166.54. HRMS (*m*/*z*) [M + H]^+^ calcd for C_25_H_23_NO_2_Cl: 404.1417, found: 404.1414.

#### 4-(4-Chlorophenyl)-3-(4-((2,4-dimethylbenzyl)oxy)phenyl)-5-methylisoxazole (**31**)

It was synthesized from compound **10** using 2,4-dimethylbenzyl bromide according to synthesis method 1a. The resulting crude product was purified by flash column chromatography (0% → 40% EtOAc in Hexane). Yield 77.0%; mp 119.7–122.1 °C. ^1^H NMR (400 MHz, CDCl_3_): δ_H_ 2.33 (3H, s), 2.34 (3H, s), 2.42 (3H, s), 4.99 (2H, s), 6.93 (2H, d, *J* = 8.8 Hz), 7.02 (1H, d, *J* = 7.4 Hz), 7.05 (1H, s), 7.13 (2H, d, *J* = 8.8 Hz), 7.26 (1H, d, *J* = 7.4 Hz), 7.35–7.37 (4H, m). ^13^C NMR (100 MHz, CDCl_3_): δ_C_ 11.56, 18.82, 21.09, 68.52, 114.53, 114.83, 121.30, 126.64, 128.94, 129.01, 129.13, 129.72, 131.13, 131.32, 131.39, 133.69, 136.74, 138.26, 159.99, 160.63, 166.53. HRMS (*m*/*z*) [M + H]^+^ calcd for C_25_H_23_NO_2_Cl: 404.1417, found: 404.1414.

#### 4-(4-Chlorophenyl)-5-methyl-3-(4-(pyridin-3-ylmethoxy)phenyl)isoxazole (**32**)

It was synthesized from compound **10** using 3-(chloromethyl)pyridine.HCl according to synthesis method 1a. The resulting crude product was purified by flash column chromatography (0% → 60% EtOAc in Hexane). Yield 53.0%; mp 115.5–116.4 °C. ^1^H NMR (400 MHz, CDCl_3_): δ_H_ 2.42 (3H, s), 5.14 (2H, s), 6.92 (2H, d, *J* = 8.4 Hz), 7.11 (2H, d, *J* = 8.4 Hz), 7.34–7.38 (4H, m), 7.52 (1H, dd, *J* = 8.0 Hz, 4.8 Hz), 7.98 (1H, d, *J* = 8.0 Hz), 8.64 (1H, d, *J* = 4.8 Hz), 8.77 (1H, s). ^13^C NMR (100 MHz, CDCl_3_): δ_C_ 11.56, 66.90, 114.55, 114.83, 122.26, 124.53, 128.93, 129.05, 129.94, 131.07, 133.79, 133.94, 137.83, 145.99, 146.58, 158.93, 160.40, 166.68. HRMS (*m*/*z*) [M + H]^+^ calcd for C_22_H_18_N_2_O_2_Cl: 377.1057, found: 377.1063.

#### 4-(4-Chlorophenyl)-5-methyl-3-(4-(pyridin-4-ylmethoxy)phenyl)isoxazole (**33**)

It was synthesized from compound **10** using 4-(chloromethyl)pyridine.HCl according to synthesis method 1a. The resulting crude product was purified by flash column chromatography (0% → 70% EtOAc in Hexane). Yield 25.0%; mp 124.2–125.8 °C. ^1^H NMR (400 MHz, CDCl_3_): δ_H_ 2.41 (3H, s), 5.19 (2H, s), 6.91 (2H, d, *J* = 8.8 Hz), 7.11 (2H, d, *J* = 8.4 Hz), 7.35 (2H, d, *J* = 8.4 Hz), 7.38 (2H, d, *J* = 8.8 Hz), 7.61 (2H, d, *J* = 6.4 Hz), 8.69 (2H, d, *J* = 6.4 Hz). ^13^C NMR (100 MHz, CDCl_3_): δ_C_ 11.49, 67.77, 114.54, 114.85, 122.49, 122.62, 128.95, 129.05, 129.97, 131.07, 133.84, 146.27, 150.53, 158.65, 160.29, 166.71. HRMS (*m*/*z*) [M + H]^+^ calcd for C_22_H_18_N_2_O_2_Cl: 377.1057, found: 377.1049.

#### 4-(4-Chlorophenyl)-5-methyl-3-(4-(quinolin-2-ylmethoxy)phenyl)isoxazole (**34**)

It was synthesized from compound **10** using 2-(chloromethyl)quinoline.HCl according to synthesis method 1a. The resulting crude product was purified by flash column chromatography (0% → 60% EtOAc in Hexane). Yield 49.0%; mp 164.7–165.8 °C. ^1^H NMR (400 MHz, CDCl_3_): δ_H_ 2.41 (3H, s), 5.38 (2H, s), 6.97 (2H, d, *J* = 8.4 Hz), 7.10 (2H, d, *J* = 8.4 Hz), 7.32–7.36 (4H, m), 7.53–7.56 (1H, m), 7.64 (1H, d, *J* = 7.8 Hz), 7.71–7.75 (1H, m), 7.82 (1H, d, *J* = 8.8 Hz), 8.07 (1H, d, *J* = 8.8 Hz), 8.19 (1H, d, *J* = 8.0 Hz). ^13^C NMR (100 MHz, CDCl_3_): δ_C_ 11.55, 71.27, 114.53, 115.02, 119.08, 121.76, 126.64, 127.59, 127.70, 128.88, 129.01, 129.83, 129.89, 131.07, 133.71, 137.14, 147.44, 157.40, 159.42, 160.52, 166.55. HRMS (*m*/*z*) [M + H]^+^ calcd for C_26_H_20_N_2_O_2_Cl: 427.1213, found: 427.1205.

#### 4-(4-Chlorophenyl)-5-methyl-3-(4-((2-methylpyridin-3-yl)methoxy)phenyl)isoxazole (**35**)

It was synthesized from compound **10** using 3-(chloromethyl)-2-methylpyridine according to synthesis method 1a. The resulting crude product was purified by flash column chromatography (0% → 20% MeOH in DCM). Yield 80.0%; mp 107.2–108.8 °C. ^1^H NMR (400 MHz, CDCl_3_): δ_H_ 2.42 (3H, s), 2.58 (3H, s), 5.04 (2H, s), 6.93 (2H, d, *J* = 8.8 Hz), 7.12 (2H, d, *J* = 8.8 Hz), 7.16 (1H, dd, *J* = 8.0, 5.2 Hz), 7.34–7.39 (4H, m), 7.71 (1H, dd, *J* = 8.0 Hz, 1.2 Hz), 8.47 (1H, dd, *J* = 5.2 Hz, 1.2 Hz). ^13^C NMR (100 MHz, CDCl_3_): δ_C_ 11.56, 21.90, 67.36, 114.54, 114.81, 121.32, 121.90, 129.03, 129.86, 130.03, 131.10, 133.75, 135.84, 148.51, 156.58, 159.41, 160.48, 166.63. HRMS (*m*/*z*) [M + H]^+^ calcd for C_23_H_20_N_2_O_2_Cl: 391.1213, found: 391.1205.

#### 4-(4-Chlorophenyl)-5-methyl-3-(4-((3-methylpyridin-2-yl)methoxy)phenyl)isoxazole (**36**)

It was synthesized from compound **10** using 2-(chloromethyl)-3-methylpyridine according to synthesis method 1a. The resulting crude product was purified by flash column chromatography (0% → 60% EtOAc in Hexane). Yield 62.0%; mp 138.7–141.2 °C. ^1^H NMR (400 MHz, DMSO): δ_H_ 2.33 (3H, s), 2.39 (3H, s), 5.17 (2H, s), 7.05 (2H, d, *J* = 8.8 Hz), 7.22–7.30 (5H, m), 7.46 (2H, d, *J* = 8.0 Hz), 7.63 (1H, d, *J* = 7.6, 1.2 Hz) 8.36 (1H, dd, *J* = 4.8 Hz, 1.2 Hz). ^13^C NMR (100 MHz, DMSO): δ_C_ 11.20, 17.47, 70.32, 113.94, 114.94, 120.88, 123.64, 128.82, 128.84, 129.39, 131.42, 132.54, 132.98, 138.24, 146.21, 153.72, 159.50, 160.05, 166.92. HRMS (*m*/*z*) [M + H]^+^ calcd for C_23_H_20_N_2_O_2_Cl: 391.1213, found: 391.1201.

#### 4-(4-Chlorophenyl)-5-methyl-3-(4-((6-methylpyridin-2-yl)methoxy)phenyl)isoxazole (**37**)

It was synthesized from compound **10** using 2-(bromomethyl)-6-methylpyridine according to synthesis method 1a. The resulting crude product was purified by flash column chromatography (0% → 60% EtOAc in Hexane). Yield 61.0%; mp 126.1–127.5 °C. ^1^H NMR (400 MHz, CDCl_3_): δ_H_ 2.41 (3H, s), 2.57 (3H, s), 5.16 (2H, s), 6.92 (2H, d, *J* = 8.4 Hz), 7.08 (1H, d, *J* = 8.0 Hz), 7.10 (2H, d, *J* = 8.4 Hz), 7.29 (1H, d, *J* = 8.0 Hz), 7.34 (4H, d, *J* = 8.4 Hz), 7.59 (1H, t, *J* = 8.0 Hz). ^13^C NMR (100 MHz, CDCl_3_): δ_C_ 11.50, 24.20, 70.52, 114.53, 114.96, 118.28, 121.69, 122.42, 128.99, 129.08, 129.77, 131.07, 133.73, 137.20, 156.05, 157.94, 159.48, 160.55, 166.49. HRMS (*m*/*z*) [M + H]^+^ calcd for C_23_H_20_N_2_O_2_Cl: 391.1213, found: 391.1205.

#### 4-(4-Chlorophenyl)-3-(4-((6-chloropyridin-3-yl)methoxy)phenyl)-5-methylisoxazole (**38**)

It was synthesized from compound **10** using 2-chloro-(5-chloromethyl)pyridine according to synthesis method 1a. The resulting crude product was purified by flash column chromatography (0% → 60% EtOAc in Hexane). Yield 70.0%; mp 168.2–170.8 °C. ^1^H NMR (400 MHz, CDCl_3_): δ_H_ 2.42 (3H, s), 5.05 (2H, s), 6.90 (2H, d, *J* = 8.4 Hz), 7.10 (2H, d, *J* = 8.4 Hz), 7.34–7.38 (5H, m), 7.73 (1H, dd, *J* = 8.0 Hz, 2.4 Hz), 8.44 (1H, d, *J* = 2.4 Hz). ^13^C NMR (100 MHz, CDCl_3_): δ_C_ 11.51, 66.72, 114.54, 114.85, 122.18, 124.29, 128.99, 129.03, 129.89, 131.08, 131.18, 133.79, 138.04, 148.67, 151.27, 159.09, 160.41, 166.63. HRMS (*m*/*z*) [M + H]^+^ calcd for C_22_H_17_N_2_O_2_Cl_2_: 411.0667, found: 411.0681.

#### 4-(4-Chlorophenyl)-3-(4-((3,5-dimethylisoxazol-4-yl)methoxy)phenyl)-5-methylisoxazole (**39**)

It was synthesized from compound **10** using 4-(chloromethyl)-3,5-dimethylisoxazole according to synthesis method 1a. The resulting crude product was purified by flash column chromatography (0% → 60% EtOAc in Hexane). Yield 87.0%; mp 138.9–141.3 °C. ^1^H NMR (400 MHz, CDCl_3_): δ_H_ 2.28 (3H, s), 2.40 (3H, s), 2.42 (3H, s), 4.79 (2H, s), 6.89 (2H, d, *J* = 8.4 Hz), 7.12 (2H, d, *J* = 8.8 Hz), 7.35–7.38 (4H, m). ^13^C NMR (100 MHz, CDCl_3_): δ_C_ 10.14, 11.15, 11.56, 59.47, 109.99, 114.53, 114.79, 121.96, 129.01, 129.04, 129.84, 131.10, 133.76, 159.30, 159.68, 160.46, 166.66, 167.56. HRMS (*m*/*z*) [M + H]^+^ calcd for C_22_H_20_N_2_O_3_Cl: 395.1162, found: 395.1153.

#### 4-(4-Chlorophenyl)-3-(4-((1,3-dimethyl-1H-pyrazol-5-yl)methoxy)phenyl)-5-methylisoxazole (**40**)

It was synthesized from compound **10** using 5-(chloromethyl)-1,3-dimethylisoxazole according to synthesis method 1a. The resulting crude product was purified by flash column chromatography (0% → 70% EtOAc in Hexane). Yield 73.0%; mp 156.4–157.9 °C. ^1^H NMR (400 MHz, CDCl_3_): δ_H_ 2.25 (3H, s), 2.42 (3H, s), 3.83 (3H, s), 4.98 (2H, s), 6.09 (1H, s), 6.90 (2H, d, *J* = 9.2 Hz), 7.11 (2H, d, *J* = 8.8 Hz), 7.34–7.37 (4H, m). ^13^C NMR (100 MHz, CDCl_3_): δ_C_ 11.55, 13.35, 36.44, 60.48, 106.82, 114.53, 114.79, 122.07, 128.99, 129.03, 129.83, 131.09, 133.76, 137.51, 147.29, 158.99, 160.44, 166.64. HRMS (*m*/*z*) [M + H]^+^ calcd for C_22_H_21_N_3_O_2_Cl: 394.1322, found: 394.1320.

#### 4-(4-Chlorophenyl)-3-(4-((1-isopropyl-1H-imidazol-5-yl)methoxy)phenyl)-5-methylisoxazole (**41**)

It was synthesized from compound **10** using 2-(chloromethyl)-1-isopropyl-1H-imidazole according to synthesis method 1a. The resulting crude product was purified by flash column chromatography (0% → 15% MeOH in DCM). Yield 69.0%; mp 168.4–170.7 °C. ^1^H NMR (400 MHz, CDCl_3_): δ_H_ 1.44 (6H, d, *J* = 6.8 Hz), 2.41 (3H, s), 4.56-4.59 (1H, m), 5.18 (2H, s), 6.98 (2H, d, *J* = 8.4 Hz), 7.03 (1H, d, *J* = 1.2 Hz), 7.06 (1H, d, *J* = 1.2 Hz), 7.10 (2H, d, *J* = 8.4 Hz) 7.34–7.36 (4H, m). ^13^C NMR (100 MHz, CDCl_3_): δ_C_ 11.54, 23.76, 48.12, 62.29, 114.54, 114.90, 116.85, 121.95, 127.87, 128.99, 129.03, 129.80, 131.09, 133.76, 141.78, 158.99, 160.46, 166.59. HRMS (*m*/*z*) [M + H]^+^ calcd for C_23_H_23_N_3_O_2_Cl: 408.1479, found: 408.1469.

#### 4-(4-Chlorophenyl)-5-methyl-3-(4-((5-(trifluoromethyl)furan-2-yl)methoxy)phenyl) isoxazole (**42**)

It was synthesized from compound **10** using 2-(bromomethyl)-5-(trifluoromethyl)furan according to synthesis method 1a. The resulting crude product was purified by flash column chromatography (0% → 40% EtOAc in Hexane). Yield 97.0%; mp 105.7–106.8 °C. ^1^H NMR (400 MHz, CDCl_3_): δ_H_ 2.42 (3H, s), 5.03 (2H, s), 6.49 (1H, d, *J* = 3.6 Hz), 6.78 (1H, d, *J* = 3.6 Hz), 6.91 (2H, d, *J* = 9.2 Hz), 7.11 (2H, d, *J* = 8.4 Hz), 7.34–7.38 (4H, m). ^13^C NMR (100 MHz, CDCl_3_): δ_C_ 11.50, 62.04, 110.38, 112.34 (q, ^3^*J*_C-F_ = 3.2 Hz), 114.56, 114.89, 118.88 (q, ^1^*J*_C-F_ = 265.4 Hz), 122.21, 129.02, 129.84, 131.07, 133.78, 138.43, 142.19 (q, ^2^*J*_C-F_ = 42.3 Hz), 152.75, 158.96, 160.45, 166.59. HRMS (*m*/*z*) [M + H]^+^ calcd for C_22_H_16_NO_3_ClF_3_: 434.0771, found: 434.0741.

#### 5-Methyl-4-(2-chlorophenyl)-3-(4-((2-methylbenzyl)oxy)phenyl)isoxazole (**44**)

It was synthesized from compound **43a** using 2-chlorophenylacetone according to synthesis method 4. The resulting crude product was purified by flash column chromatography (0% → 30% EtOAc in Hexane). Yield 42.0%; mp 103.8–104.4 °C. ^1^H NMR (400 MHz, CDCl_3_): δ_H_ 2.33 (3H, s), 2.35 (3H, s), 5.00 (2H, s), 6.89 (2H, d, *J* = 8.8 Hz), 7.18–7.38 (9H, m), 7.50 (1H, dd, *J* = 8.4, 0.8 Hz). ^13^C NMR (100 MHz, CDCl_3_): δ_C_ 11.68, 18.87, 68.53, 113.13, 114.79, 121.92, 126.03, 127.05, 128.34, 128.59, 129.01, 129.75, 129.82, 129.98, 130.40, 132.58, 134.45, 134.98, 136.66, 159.84, 160.70, 167.46. HRMS (*m*/*z*) [M + H]^+^ calcd for C_24_H_21_ClNO_2_: 390.1261, found: 390.1249.

#### 5-Methyl-4-(4-fluorophenyl)-3-(4-((2-methylbenzyl)oxy)phenyl)isoxazole (**45**)

It was synthesized from compound **43a** using 4-fluorophenylacetone according to synthesis method 4. The resulting crude product was purified by flash column chromatography (0% → 30% EtOAc in Hexane). Yield 31.0%; mp 119.0–120.5 °C. ^1^H NMR (400 MHz, CDCl_3_): δ_H_ 2.37 (3H, s), 2.42 (3H, s), 5.03 (2H, s), 6.93 (2H, d, *J* = 8.8 Hz), 7.08 (2H, t, *J* = 8.8 Hz), 7.14–7.28 (5H, m), 7.35–7.39 (3H, m). ^13^C NMR (100 MHz, CDCl_3_): δ_C_ 11.50, 18.87, 68.57, 114.65, 114.79, 115.82 (d, ^2^*J*_C-F_ = 21.4 Hz), 121.55, 126.05, 126.55 (d, ^4^*J*_C-F_ = 3.8 Hz), 128.38, 128.62, 129.71, 130.43, 131.55 (d, ^3^*J*_C-F_ = 8.4 Hz), 134.41, 136.69, 159.86, 160.64, 162.28 (d, ^1^*J*_C-F_ = 245.4 Hz), 166.44. HRMS (*m*/*z*) [M + H]^+^ calcd for C_24_H_21_FNO_2_: 374.1556, found: 374.1559.

#### 5-Methyl-4-(4-methoxyphenyl)-3-(4-((2-methylbenzyl)oxy)phenyl)isoxazole (**46**)

It was synthesized from compound **43a** using 4-methoxyphenylacetone according to synthesis method 4. The resulting crude product was purified by flash column chromatography (0% → 30% EtOAc in Hexane). Yield 43.0%; mp 121.3–122.7 °C. ^1^H NMR (400 MHz, CDCl_3_): δ_H_ 2.36 (3H, s), 2.41 (3H, s), 3.84 (3H, s), 5.02 (2H, s), 6.92 (2H, d, *J* = 9.2 Hz), 6.93 (2H, d, *J* = 8.8 Hz), 7.11 (2H, d, *J* = 8.8 Hz), 7.19–7.26 (3H, m), 7.38–7.42 (3H, m). ^13^C NMR (100 MHz, CDCl_3_): δ_C_ 15.44, 22.81, 59.17, 72.49, 118.12, 118.64, 110.09, 125.85, 126.65, 129.98, 132.29, 132.55, 133.65, 134.35, 134.96, 138.42, 140.62, 163.00, 163.69, 164.62, 170.13. HRMS (*m*/*z*) [M + H]^+^ calcd for C_25_H_24_NO_3_: 386.1756, found: 386.1753.

#### 5-Methyl-4-(4-methylphenyl)-3-(4-((2-methylbenzyl)oxy)phenyl)isoxazole (**47**)

It was synthesized from compound **43a** using 4-methylphenylacetone according to synthesis method 4. The resulting crude product was purified by flash column chromatography (0% → 30% EtOAc in Hexane). Yield 34.0%; mp 130.8–131.8 °C. ^1^H NMR (400 MHz, CDCl_3_): δ_H_ 2.37 (3H, s), 2.39 (3H, s), 2.42 (3H; s), 5.03 (2H, s), 6.93 (2H, d, *J* = 8.8 Hz), 7.08 (2H, d, *J* = 8.4 Hz), 7.18–7.28 (5H, m), 7.38–7.43 (3H, m). ^13^C NMR (100 MHz, CDCl_3_): δ_C_ 11.53, 18.88, 21.26, 68.55, 114.69, 115.44, 121.90, 126.04, 127.53, 128.35, 128.62, 129.42, 129.71, 129.74, 130.41, 134.49, 136.68, 137.36, 159.76, 160.69, 166.28. HRMS (*m*/*z*) [M + H]^+^ calcd for C_25_H_24_NO_2_: 370.1807, found: 370.1796.

#### 5-Methyl-4-(4-cyanophenyl)-3-(4-((2-methylbenzyl)oxy)phenyl)isoxazole (**48**)

It was synthesized from compound **43a** using 4-cyanophenylacetone according to synthesis method 4. The resulting crude product was purified by flash column chromatography (0% → 40% EtOAc in Hexane). Yield 34.0%; mp 135.3–137.1 °C. ^1^H NMR (400 MHz, CDCl_3_): δ_H_ 2.37 (3H, s), 2.47 (3H, s), 5.04 (2H, s), 6.95 (2H, d, *J* = 8.8 Hz), 7.19–7.33 (7H, m), 7.33–7.39 (1H, m), 7.67 (2H, d, *J* = 8.4 Hz). ^13^C NMR (100 MHz, CDCl_3_): δ_C_ 11.74, 18.89, 68.63, 111.46, 114.28, 114.98, 118.53, 120.87, 126.07, 128.45, 128.62, 129.83, 130.35, 130.47, 132.50, 134.29, 135.68, 136.69, 160.10, 160.60, 167.08. HRMS (*m*/*z*) [M + H]^+^ calcd for C_25_H_21_N_2_O_2_: 381.1603, found: 381.1600.

#### 5-Methyl-4-(3-trifluoromethylphenyl)-3-(4-((2-methylbenzyl)oxy)phenyl)isoxazole (**49**)

It was synthesized from compound **43a** using 3-(trifluoromethyl)phenylacetone according to synthesis method 4. The resulting crude product was purified by flash column chromatography (0% → 30% EtOAc in Hexane). Yield 41.0%; mp 120.7–122.4 °C. ^1^H NMR (400 MHz, CDCl_3_): δ_H_ 2.36 (3H, s), 2.46 (3H, s), 5.03 (2H, s), 6.94 (2H, d, *J* = 8.8 Hz), 7.19–7.28 (3H, m), 7.33–7.39 (4H, m), 7.48–7.52 (2H, m), 7.62 (1H, d, *J* = 8.0 Hz). ^13^C NMR (100 MHz, CDCl_3_): δ_C_ 11.60, 18.85, 68.61, 114.42, 114.90, 121.15, 123.84 (q, ^1^*J*_C-F_ = 271.2 Hz), 124.43 (q, ^3^*J*_C-F_ = 3.6 Hz), 126.05, 126.41 (q, ^3^*J*_C-F_ = 3.8 Hz), 128.39, 128.60, 129.23, 129.75, 130.43, 131.23 (q, ^2^*J*_C-F_ = 32.1 Hz), 131.60, 133.20, 134.37, 136.68, 160.00, 160.60, 166.88. HRMS (*m*/*z*) [M + H]^+^ calcd for C_25_H_21_F_3_NO_2_: 424.1524, found: 424.1523.

#### 5-Methyl-4-(3-fluoromethylphenyl)-3-(4-((2-methylbenzyl)oxy)phenyl)isoxazole (**50**)

It was synthesized from compound **43a** using 3-fluorophenylacetone according to synthesis method 4. The resulting crude product was purified by flash column chromatography (0% → 30% EtOAc in Hexane). Yield 43.0%; mp 89.3–91.2 °C. ^1^H NMR (400 MHz, CDCl_3_): δ_H_ 2.37 (3H, s), 2.44 (3H, s), 5.03 (2H, s), 6.89-6.99 (4H, m), 7.05 (1H, td, *J* = 8.4, 2.4 Hz), 7.20–7.28 (3H, m), 7.32–7.40 (4H, m). ^13^C NMR (100 MHz, CDCl_3_): δ_C_ 11.58, 18.88, 68.58, 114.66 (d, ^2^*J*_C-F_ = 20.5 Hz), 114.83, 116.74 (d, ^2^*J*_C-F_ = 21.4 Hz), 121.35, 125.63, 125.64, 126.05, 128.38, 128.64, 129.74, 130.27 (d, ^3^*J*_C-F_ = 8.3 Hz), 130.42, 132.81 (d, ^3^*J*_C-F_ = 8.4 Hz), 134.41, 136.70, 159.93, 160.61, 162.79 (d, ^1^*J*_C-F_ = 245.4 Hz), 166.70. HRMS (*m*/*z*) [M + H]^+^ calcd for C_24_H_21_FNO_2_: 374.1556, found: 374.1563.

#### 5-Methyl-4-(4-aminophenyl)-3-(4-((2-methylbenzyl)oxy)phenyl)isoxazole (**51**)

5-Methyl-4-(4-nitrophenyl)-3-(4-((2-methylbenzyl)oxy)phenyl)isoxazole was synthesized from compound **43a** using 4-nitrophenylacetone according to synthesis method 4. The resulting crude product was purified by flash column chromatography (0% → 30% EtOAc in Hexane). Yield 24.0%; mp 119.4–121.1 °C. ^1^H NMR (400 MHz, CDCl_3_): δ_H_ 2.29 (3H, s), 2.46 (3H, s), 5.08 (2H, s), 7.06 (2H, d, *J* = 8.4 Hz), 7.16–7.29 (5H, m), 7.38 (1H, d, *J* = 7.2 Hz), 7.50 (2H, d, *J* = 8.8 Hz), 8.25 (2H, d, *J* = 8.8 Hz). HRMS (*m*/*z*) [M + H]^+^ calcd for C_24_H_21_N_2_O_4_: 401.1501, found: 401.1493. In the next step, compound **51** was synthesized from the intermediate nitro product according to synthesis method 5. The resulting crude product was purified by flash column chromatography (0% → 40% EtOAc in Hexane). Yield 71.0%; mp 149.5–151.3 °C. ^1^H NMR (400 MHz, CDCl_3_): δ_H_ 2.37 (3H, s), 2.41 (3H, s), 5.03 (2H, s), 6.74 (2H, d, *J* = 8.8 Hz), 6.93 (2H, d, *J* = 8.8 Hz), 7.00 (2H, d, *J* = 8.8 Hz), 7.21–7.27 (3H, m), 7.39–7.42 (1H, m), 7.43 (2H, d, *J* = 8.8 Hz). ^13^C NMR (100 MHz, CDCl_3_): δ_C_ 11.51, 18.89, 68.55, 114.66, 115.38, 115.68, 120.97, 122.07, 126.05, 128.35, 128.63, 129.71, 130.42, 130.90, 134.51, 136.69, 144.98, 159.71, 160.70, 166.04. HRMS (*m*/*z*) [M + H]^+^ calcd for C_24_H_23_N_2_O_2_: 371.1760, found: 371.1759.

#### 5-Methyl-4-(2,4-difluoromethylphenyl)-3-(4-((2-methylbenzyl)oxy)phenyl)isoxazole (**52**)

It was synthesized from compound **43a** using 2,4-difluorophenylacetone according to synthesis method 4. The resulting crude product was purified by flash column chromatography (0% → 30% EtOAc in Hexane). Yield 34.0%; mp 116.1–117.4 °C. ^1^H NMR (400 MHz, CDCl_3_): δ_H_ 2.37 (3H, s), 2.39 (3H, s), 5.03 (2H, s), 6.90-6.96 (4H, m), 7.10–7.15 (1H, m), 7.20–7.27 (3H, m), 7.35–7.40 (3H, m). ^13^C NMR (100 MHz, CDCl_3_): δ_C_ 11.61 (d, ^5^*J*_C-F_ = 1.9 Hz), 18.88, 68.58, 104.63 (t, ^2^*J*_C-F_ = 25.3 Hz), 108.60, 111.83 (dd, ^2^*J*_C-F_ = 21.8, ^4^*J*_C-F_ = 3.8 Hz), 114.43 (dd, ^2^*J*_C-F_ = 21.8, ^4^*J*_C-F_ = 3.8 Hz), 114.86, 121.58, 126.06, 128.40, 128.63, 129.21, 130.44, 133.01 (dd, ^3^*J*_C-F_ = 9.6, ^3^*J*_C-F_ = 3.8 Hz), 134.40, 136.69, 159.94, 160.29 (d, ^1^*J*_C-F_ = 248.4 Hz, ^3^*J*_C-F_ = 11.8 Hz), 160.94, 162.84 (dd, ^1^*J*_C-F_ = 248.8, ^3^*J*_C-F_ = 11.5 Hz), 167.74. HRMS (*m*/*z*) [M + H]^+^ calcd for C_24_H_20_FNO_2_: 392.1462, found: 392.1468.

#### 5-Methyl-4-(2,4-dichlorophenyl)-3-(4-((2-methylbenzyl)oxy)phenyl)isoxazole (**53**)

It was synthesized from compound **43a** using 2,4-dichlorophenylacetone according to synthesis method 4. The resulting crude product was purified by flash column chromatography (0% → 30% EtOAc in Hexane). Yield 40.0%; mp 98.0–98.4 °C. ^1^H NMR (400 MHz, CDCl_3_): δ_H_ 2.33, (3H, s), 2.35, (3H, s), 5.01 (2H, s), 6.91, (2H, d, *J* = 8.8 Hz), 7.13 (1H, d, *J* = 8.0 Hz), 7.18–7.26 (3H, m), 7.28 (1H, dd, *J* = 8.0, 2.4 Hz), 7.32 (2H, d, *J* = 8.8 Hz), 7.36–7.38 (1H, m), 7.53 (1H, d, *J* = 2.4 Hz). ^13^C NMR (100 MHz, CDCl_3_): δ_C_ 11.66, 18.87, 68.55, 112.15, 114.89, 121.61, 126.05, 127.52, 128.37, 128.45, 128.59, 128.98, 129.93, 130.42, 133.25, 134.39, 135.04, 135.74, 136.67, 159.94, 160.65, 167.63. HRMS (*m*/*z*) [M + H]^+^ calc for C_24_H_20_Cl_2_NO_2_: 424.0871, found: 424.0870.

#### 5-Methyl-3-(4-((2-methylbenzyl)oxy)phenyl)-4-(5-methylpyridin-2-yl)isoxazole (**54**)

It was synthesized from compound **43a** using 1-(5-methylpyridin-2-yl)acetone according to synthesis method 4. The resulting crude product was purified by flash column chromatography (0% → 10% MeOH in DCM). Yield 52.0%; mp 121.4–122.3 °C. ^1^H NMR (400 MHz, CDCl_3_): δ_H_ 2.37 (6H, s), 2.57 (3H, s), 5.04 (2H, s), 6.94-6.99 (3H, m), 7.20–7.28 (3H, m), 7.38–7.44 (4H, m), 8.52 (1H, d, *J* = 1.6 Hz). ^13^C NMR (100 MHz, CDCl_3_): δ_C_ 12.09, 18.26, 18.88, 68.60, 114.80, 115.01, 121.71, 124.19, 126.05, 128.38, 128.63, 129.98, 130.43, 131.76, 134.45, 136.69, 137.06, 147.64, 150.11, 156.91, 160.81, 168.61. HRMS (*m*/*z*) [M + H]^+^ calcd for C_24_H_23_N_2_O_2_: 371.1760, found: 371.1769.

#### 5-Methyl-3-(4-((2-methylbenzyl)oxy)phenyl)-4-(pyridin-4-yl)isoxazole (**55**)

It was synthesized from compound **43a** using 4-(pyridyl)acetone according to synthesis method 4. The resulting crude product was purified by flash column chromatography (0% → 30% EtOAc in Hexane). Yield 30.0%; mp 128.5–129.3 °C. ^1^H NMR (CDCl_3_): δ_H_ 2.37 (3H, s), 2.50 (3H, s), 5.04 (2H, s), 6.96 (2H, d, *J* = 8.6 Hz), 7.13 (2H, d, *J* = 6.2 Hz), 7.19–7.28 (3H, m), 7.34 (2H, d, *J* = 8.6 Hz), 7.38–7.40 (1H, m), 8.62 (2H, d, *J* = 6.2 Hz). ^13^C NMR (100 MHz, CDCl_3_): δ_C_ 11.79, 18.88, 68.63, 113.36, 114.99, 120.81, 124.35, 126.07, 128.44, 128.64, 129.86, 130.46, 134.29, 136.70, 139.25, 149.96, 160.13, 160.62, 167.40. HRMS (*m*/*z*) [M + H]^+^ calcd for C_23_H_21_N_2_O_2_: 357.1603, found: 357.1593.

#### 4-(4-Chlorophenyl)-3-(2-fluoro-4-((2-methylbenzyl)oxy)phenyl)-5-methylisoxazole (**56**)

It was synthesized from compound **43b** using 4-chlorophenylacetone according to synthesis method 4. The resulting crude product was purified by flash column chromatography (0% → 30% EtOAc in Hexane). Yield 20.0%; mp 96.1–98.3 °C. ^1^H NMR (400 MHz, CDCl_3_): δ_H_ 2.37 (3H, s), 2.49 (3H, s), 5.03 (2H, s), 6.68 (1H, dd, *J* = 11.6 Hz, 2.4 Hz), 6.82 (1H, dd, *J* = 8.4 Hz, 2.4 Hz), 7.07 (2H, d, *J* = 8.4 Hz), 7.35–7.38 (7H, m). ^13^C NMR (100 MHz, CDCl_3_): δ_C_ 11.68, 18.88, 69.02, 102.95 (d, ^2^*J*_C-F_ = 25.1 Hz), 109.43 (d, ^2^*J*_C-F_ = 15.3 Hz), 111.07 (d, ^4^*J*_C-F_ = 3.0 Hz), 115.93, 126.12, 128.61, 128.71, 128.88, 130.04, 130.52, 131.69 (d, ^3^*J*_*C- F*_ = 4.6 Hz), 133.45, 133.83, 136.76, 157.60, 160.67 (d, ^1^*J*_C-F_ = 249.9 Hz), 161.46 (d, ^3^*J*_C-F_ = 10.7 Hz), 166.13. HRMS (*m*/*z*) [M + H]^+^ calcd for C_24_H_20_ClNO_2_F: 408.1167, found: 408.1180.

#### 4-(4-Chlorophenyl)-3-(3-fluoro-4-((2-methylbenzyl)oxy)phenyl)-5-methylisoxazole (**57**)

It was synthesized from compound **43c** using 4-chlorophenylacetone according to synthesis method 4. The resulting crude product was purified by flash column chromatography (0% → 30% EtOAc in Hexane). Yield 24.0%; mp 108.0–110.2 °C. ^1^H NMR (400 MHz, CDCl_3_): δ_H_ 2.39 (3H, s), 2.43 (3H, s), 5.12 (2H, s), 6.96–7.00 (1H, m), 7.10–7.14 (3H, m), 7.22–7.29 (4H, m), 7.37–7.41 (3H, m). ^13^C NMR (100 MHz, CDCl_3_): δ_C_ 11.53, 18.88, 69.88, 114.54, 115.19 (d, ^4^*J*_C-F_ = 1.9 Hz), 116.29 (d, ^2^*J*_C-F_ = 20.5 Hz), 122.13 (d, ^3^*J*_C-F_ = 7.1 Hz), 124.54 (d, ^3^*J*_C-F_ = 3.9 Hz), 126.06, 128.53, 128.59, 128.70, 129.16, 130.47, 131.10, 133.93, 133.98, 136.72, 147.94 (d, ^2^*J*_C-F_ = 10.2 Hz), 152.54 (d, ^1^*J*_C-F_ = 245.6 Hz), 159.69 (d, ^4^*J*_C-F_ = 1.9 Hz), 166.95. HRMS (*m*/*z*) [M + H]^+^ calcd for C_24_H_20_ClNO_2_F: 408.1167, found: 408.1169.

#### 4-(4-(4-Chlorophenyl)-5-methylisoxazol-3-yl)-N-(2-methylbenzyl)aniline (**59**)

It was synthesized from compound **58** according to synthesis method 1c. The resulting crude product was purified by flash column chromatography (0% → 30% EtOAc in Hexane). Yield 31.0%; mp 134.9–137.0 °C. ^1^H NMR (400 MHz, CDCl_3_): δ_H_ 2.35 (3H, s), 2.40 (3H, s), 4.27 (2H, s), 6.58 (2H, d, *J* = 8.8 Hz), 7.14 (2H, d, *J* = 8.4 Hz), 7.17–7.30 (6H, m), 7.35 (2H, d, *J* = 8.4 Hz). ^13^C NMR (100 MHz, CDCl_3_): δ_C_ 11.54, 18.94, 46.33, 112.74, 114.35, 126.21, 127.69, 128.39, 128.93, 129.46, 129.52, 130.49, 131.18, 133.55, 136.01, 136.40, 148.68, 160.84, 157.60, 166.27. HRMS (*m*/*z*) [M + H]^+^ calcd for C_24_H_22_N_2_OCl: 389.1421, found: 389.1418.

#### 4-(4-Chlorophenyl)-3-methyl-5-(4-((2-methylbenzyl)oxy)phenyl)isoxazole (**60**)

Compound **60** was prepared as described previously.^8^ mp 153.8-154.9 °C. ^1^H NMR (400 MHz, CDCl_3_): δ_H_ 2.23, (3H, s),2.37 (3H, s), 5.04 (2H, s), 6.94 (2H, d, *J* = 8.8 Hz), 7.20–7.29 (5H, m), 7.37–7.39 (1H, m), 7.42 (2H, d, *J* = 8.0 Hz), 7.47 (2H, d, *J* = 8.8 Hz). ^13^C NMR (100 MHz, CDCl_3_): δ_C_ 10.62, 18.89, 68.65, 113.80, 114.97, 120.49, 126.09, 128.44, 128.49, 128.62, 129.37, 129.41, 130.49, 131.25, 134.06, 134.20, 136.70, 159.80, 160.10, 164.59. CAS: 1884212-37-6.

#### 4-(4-Chlorophenyl)-5-(4-((2-methylbenzyl)oxy)phenyl)isoxazole (**63**)

It was synthesized from compound **62** using 2-methylbenzyl bromide according to synthesis method 1b. The resulting crude product was purified by flash column chromatography (0% → 20% EtOAc in Hexane). Yield 47.0%; mp 115.3–117.3 °C. ^1^H NMR (400 MHz, CDCl_3_): δ_H_ 2.38 (3H, s), 5.07 (2H, s), 7.00 (2H, d, *J* = 8.8 Hz), 7.23–7.28 (3H, m), 7.32 (2H, d, *J* = 8.8 Hz), 7.36–7.40 (3H, m), 7.56 (2H, d, *J* = 8.8 Hz), 8.31 (1H, s). ^13^C NMR (100 MHz, CDCl_3_): δ_C_ 18.89, 68.70, 113.93, 115.11, 120.10, 126.11, 128.53, 128.65, 128.85, 128.88, 129.26, 129.93, 130.51, 133.91, 134.15, 136.72, 151.55, 160.37, 164.36. HRMS (*m*/*z*) [M + H]^+^ calcd for C_23_H_19_ClNO_2_: 376.1104, found: 376.1102.

#### 4-(4-Chlorophenyl)-3-(4-((2-methylbenzyl)oxy)phenyl)isoxazole-5-amine (**65**)

Compound **64** (1.0 equiv) and NH_2_OH.HCl (1.5 equiv) were dissolved in ethanol and heated under reflux for 4.5 h. The reaction mixture was cooled, and the precipitate was filtered under vacuum and dried. The resulting crude product was purified by flash column chromatography (0% → 50% EtOAc in Hexane). Yield 75.0%; mp 163.1–165.6 °C. ^1^H NMR (400 MHz, CDCl_3_): δ_H_ 2.37 (3H, s), 5.03 (2H, s), 6.94 (2H, d, *J* = 8.8 Hz), 7.14 (2H, d, *J* = 8.4 Hz), 7.19–7.29 (3H, m), 7.33 (2H, d, *J* = 8.4 Hz), 7.36–7.39 (3H, m). ^13^C NMR (100 MHz, CDCl_3_): δ_C_ 18.88, 68.59, 93.16, 114.80, 121.57, 126.05, 128.39, 128.64, 129.25, 129.32, 129.70, 130.43, 130.54, 132.91, 134.40, 136.70, 159.98, 161.38, 165.48. HRMS (*m*/*z*) [M + H]^+^ calcd for C_23_H_20_ClN_2_O_2_: 391.1213, found: 391.1207.

#### 3-(4-Chlorophenyl)-5-methyl-4-(4-((2-methylbenzyl)oxy)phenyl)isoxazole (**67**)

3-(4-Chlorophenyl)-5-methyl-4-(4-((2-methylbenzyl)oxy)-phenyl)-4,5-dihydroisoxazol-5-ol was synthesized from compound **66** using 4-chlorophenylacetone according to method 4. Yield 18.0%. HRMS (*m*/*z*) [M + H]+ calcd for C_24_H_23_ClNO_3_: 408.1366, found: 408.1360. To the solution of obtained isoxazoline intermediate (0.2 mmol) in methanol:water (4 mL:2 mL) was added Na_2_CO_3_ (0.41 mmol) and stirred at 90 °C under reflux for 2.5 h. At the end of the period, methanol was evaporated and extracted with ethyl acetate, and the organic layer was dried with anhydrous Na_2_SO_4_. Ethyl acetate was evaporated under reduced pressure. The resulting crude product was purified by flash column chromatography (0% → 10% EtOAc in Hexane). Yield 76.0%; mp 84.5–86.5 °C. ^1^H NMR (400 MHz, CDCl_3_): δ_H_ 2.41 (3H, s), 2.43 (3H, s), 5.06 (2H, s), 7.01 (2H, d, *J* = 8.8 Hz), 7.09 (2H, d, *J* = 8.8 Hz), 7.22–7.32 (5H, m), 7.38–7.44 (3H, m). ^13^C NMR (100 MHz, CDCl_3_): δ_C_ 11.52, 18.91, 68.65, 115.13, 115.28, 122.39, 126.08, 127.78, 128.43, 128.70, 128.76, 129.68, 130.47, 130.96, 134.49, 135.42, 136.73, 158.57, 160.16, 166.65. HRMS (*m*/*z*) [M + H]^+^ calcd for C_24_H_21_ClNO_2_: 390.1261, found: 390.1251.

#### 1-(4-Chlorophenyl)-3-methyl-5-(4-((2-methylbenzyl)oxy)phenyl)-1H-pyrazole (**69**)

It was synthesized from compound **68** using 4-chlorophenylhydrazine.HCl according to synthesis method 8c. The resulting crude product was purified by preparative column chromatography (0% → 60% ACN in Water). Yield 54.0%; mp 111.8–113.6 °C. ^1^H NMR (400 MHz, CDCl_3_): δ_H_ 2.37 (3H, s), 2.38 (3H, s), 5.03 (2H, s), 6.25 (1H, s), 6.93 (2H, d, *J* = 8.8 Hz), 7.14 (2H, d, *J* = 8.8 Hz), 7.21–7.30 (7H, m), 7.38–7.41 (1H, m). ^13^C NMR (100 MHz, CDCl_3_): δ_C_ 13.52, 18.89, 68.63, 107.68, 114.83, 123.04, 126.06, 126.16, 128.43, 128.65, 128.98, 129.96, 130.46, 132.59, 134.38, 136.70, 138.68, 143.61, 149.71, 158.96. HRMS (*m*/*z*) [M + H]^+^ calcd for C_24_H_22_ClN_2_O: 389.1421, found: 389.1413.

#### 4-(4-Chlorophenyl)-5-(4-((2-methylbenzyl)oxy)phenyl)-1H-pyrazole (**71**)

It was synthesized from compound **70** using hydrazine hydrate according to synthesis method 8b. The resulting crude product was purified by flash column chromatography (0% → 30% EtOAc in Hexane). Yield 88.0%; mp 127.8–129.8 °C. ^1^H NMR (400 MHz, CDCl_3_): δ_H_ 2.38 (3H, s), 5.05 (2H, s), 6.98 (2H, d, *J* = 8.4 Hz), 7.22–7.30 (7H, m), 7.36–7.41 (3H, m), 7.65 (1H, s). ^13^C NMR (100 MHz, CDCl_3_): δ_C_ 18.90, 68.65, 115.10, 118.41, 123.08, 126.07, 128.42, 128.64, 128.97, 129.48, 129.58, 130.46, 131.51, 132.50, 134.42, 135.57, 136.69, 142.98, 159.24. HRMS (*m*/*z*) [M + H]^+^ calcd for C_23_H_20_ClN_2_O: 375.1264, found: 375.1266.

#### 4-(4-Chlorophenyl)-1-methyl-3-(4-((2-methylbenzyl)oxy)phenyl)-1H-pyrazole (**72**)

It was synthesized from compound **70** using methylhydrazine according to synthesis method 8b. The resulting crude product was purified by flash column chromatography (0% → 50% EtOAc in Hexane). Yield 56.0%; mp 142.2–143.4 °C. ^1^H NMR (400 MHz, CDCl_3_): δ_H_ 2.41 (3H, s), 3.78 (3H, s), 5.08 (2H, s), 7.06 (2H, d, *J* = 8.8 Hz), 7.11 (2H, d, *J* = 8.8 Hz), 7.18 (2H, d, *J* = 8.8 Hz), 7.21 (2H, d, *J* = 8.8 Hz), 7.23–7.31 (3H, m), 7.42–7.44 (1H, m), 7.70 (1H, s). ^13^C NMR (100 MHz, CDCl_3_): δ_C_ 18.92, 37.21, 68.70, 115.28, 119.81, 122.30, 126.10, 128.52, 128.59, 128.72, 130.50, 131.36, 131.76, 131.77, 134.32, 136.75, 137.19, 139.89, 159.41. HRMS (*m*/*z*) [M + H]^+^ calcd for C_24_H_22_ClN_2_O: 389.1421, found: 389.1429.

#### 5-(4-Chlorophenyl)-2-methyl-4-(4-((2-methylbenzyl)oxy)phenyl)thiazole (**75**)

It was synthesized from compound **74** using 2-methylbenzyl bromide according to synthesis method 1a. The resulting crude product was purified by flash column chromatography (0% → 40% EtOAc in Hexane). Yield 52.0%; mp 90.5–92.1 °C. ^1^H NMR (400 MHz, CDCl_3_): δ_H_ 2.37, (3H, s), 2.73 (3H, m), 5.03 (2H, s), 6.91 (2H, d, *J* = 8.8 Hz), 7.21–7.30 (7H, m), 7.39–7.43 (3H, m). ^13^C NMR (100 MHz, CDCl_3_): δ_C_ 18.89, 19.20, 68.55, 114.70, 126.01, 127.37, 128.28, 128.61, 128.93, 129.75, 130.25, 130.38, 130.77, 130.91, 133.77, 134.65, 136.65, 149.57, 158.65, 163.98. HRMS (*m*/*z*) [M + H]^+^ calcd for C_24_H_21_ClNOS: 406.1032, found: 406.1028.

#### 5-(4-Chlorophenyl)-1-(4-((2-methylbenyl)oxy)phenyl)-1H-pyrazole (**78**)

It was synthesized from compound **76** according to synthesis method 1a. The resulting crude product was purified by flash column chromatography (0% → 40% EtOAc in Hexane). Yield 85.0%; mp 145.2–146.4 °C. ^1^H NMR (400 MHz, CDCl_3_): δ_H_ 2.38 (3H, s), 5.04 (2H, s), 6.50 (1H, d, *J* = 2.0 Hz), 6.96 (2H, d, *J* = 9.2 Hz), 7.17 (2H, d, *J* = 8.8 Hz), 7.20–7.26 (5H, m), 7.28 (2H, d, *J* = 8.8 Hz), 7.39–7.41 (1H, m), 7.70 (1H, d, *J* = 2.0 Hz). ^13^C NMR (100 MHz, CDCl_3_): δ_C_ 18.89, 68.87, 107.44, 115.11, 126.06, 126.66, 128.43, 128.63, 128.74, 128.95, 129.92, 130.45, 133.14, 134.23, 134.32, 136.68, 139.91, 141.81, 158.31. HRMS (*m*/*z*) [M + H]^+^ calcd for C_23_H_20_ClN_2_O: 375.1264, found: 375.1262.

#### 5-(4-Chlorophenyl)-3-methyl-1-(4-((2-methylbenyl)oxy)phenyl)-1H-pyrazole (**79**)

It was synthesized from compound **77** using 2-methylbenzyl bromide according to synthesis method 1a. The resulting crude product was purified by flash column chromatography (0% → 40% EtOAc in Hexane). Yield 83.0%; mp 103.4–105.2 °C. ^1^H NMR (400 MHz, CDCl_3_): δ_H_ 2.37 (3H, s), 2.38 (3H, s), 5.03 (2H, s), 6.29 (1H, s), 6.94 (2H, d, *J* = 8.4 Hz), 7.15 (2H, d, *J* = 8.4 Hz), 7.18 (2H, d, *J* = 8.4 Hz), 7.21–7.28 (5H, m), 7.39 (1H, m). ^13^C NMR (100 MHz, CDCl_3_): δ_C_ 13.48, 18.89, 68.85, 107.25, 115.11, 126.04, 126.63, 128.38, 128.59, 128.68, 129.10, 129.78, 130.43, 133.24, 134.06, 134.38, 136.65, 142.49, 149.13, 158.10. HRMS (*m*/*z*) [M + H]^+^ calcd for C_24_H_22_ClN_2_O: 389.1421,found: 389.1416.

#### 1-(4-Chlorophenyl)-5-(4-((2-methylbenzyl)oxy)phenyl)-1H-pyrazole (**85**)

It was synthesized from compound **80** using 4-chlorophenylhydrazine.HCl according to synthesis method 8a. The resulting crude product was purified by flash column chromatography (0% → 20% EtOAc in Hexane). Yield 61.0%; mp 146.0–147.9 °C. ^1^H NMR (400 MHz, CDCl_3_): δ_H_ 2.38 (3H, s), 5.04 (2H, s), 6.45 (1H, d, *J* = 2.0 Hz), 6.94 (2H, d, *J* = 8.8 Hz), 7.16 (2H, d, *J* = 8.8 Hz), 7.21–7.29 (5H, m), 7.32 (2H, d, *J* = 8.8 Hz), 7.38–7.41 (1H, m), 7.71 (1H, d, *J* = 2.0 Hz). ^13^C NMR (100 MHz, CDCl_3_): δ_C_ 18.90, 68.65, 107.72, 114.88, 122.87, 126.08, 126.27, 128.45, 128.66, 129.05, 130.08, 130.47, 132.98, 134.36, 136.70, 138.68, 140.49, 142.91, 159.04. HRMS (*m*/*z*) [M + H]^+^ calcd for C_23_H_20_ClN_2_O: 375.1264, found: 375.1263. CAS: 2750233-50-0.

#### 2-(1-(4-Chlorophenyl)-1H-pyrazol-5-yl)-5-((2-methylbenzyl)oxy)pyridine (**86)**

It was synthesized from compound **81** using 4-chlorophenylhydrazine.HCl according to synthesis method 8a. The resulting crude product was purified by flash column chromatography (0% → 30% EtOAc in Hexane). Yield 84.0%; mp 108.4–110.4 °C. ^1^H NMR (400 MHz, CDCl_3_): δ_H_ 2.38, (3H, s), 5.09 (2H, s), 6.68 (1H, d, *J* = 1.4 Hz), 7.17 (1H, d, *J* = 8.8 Hz), 7.21–7.34 (8H, m), 7.38 (1H, d, *J* = 7.6 Hz), 7.72 (1H, d, *J* = 1.4 Hz), 8.34 (1H, d, *J* = 2.4 Hz). ^13^C NMR (100 MHz, CDCl_3_): δ_C_ 18.90, 69.11, 108.55, 121.68, 124.11, 126.16, 126.38, 128.72, 128.80, 128.97, 130.61, 133.12, 133.58, 136.76, 137.99, 139.06, 140.53, 141.91, 142.00, 154.36. HRMS (*m*/*z*) [M + H]^+^ calcd for C_22_H_19_ClN_3_O: 376.1217, found: 376.1207.

#### 5-(1-(4-Chlorophenyl)-1H-pyrazol-5-yl)-2-((2-methylbenzyl)oxy)pyridine (**87**)

It was synthesized from compound **82** using 4-chlorophenylhydrazine.HCl according to synthesis method 8a. The resulting crude product was purified by flash column chromatography (0% → 40% EtOAc in Hexane). Yield 37.0%; mp 126.5–127.8 °C. ^1^H NMR (400 MHz, CDCl_3_): δ_H_ 2.41 (3H, s), 5.38 (2H, s), 6.50 (1H, d, *J* = 1.8 Hz), 6.74 (1H, d, *J* = 8.8 Hz), 7.22- 7.28 (5H, m), 7.33–7.37 (3H, m), 7.42–7.44 (1H, m), 7.73 (1H, d, *J* = 1.8 Hz), 8.14 (1H, d, *J* = 2.0 Hz). ^13^C NMR (100 MHz, CDCl_3_): δ_C_ 18.98, 66.45, 108.07, 111.13, 119.83, 125.96, 126.33, 128.32, 129.09, 129.31, 130.35, 133.44, 134.77, 137.01, 138.35, 138.77, 139.90, 140.74, 146.58, 163.49. HRMS (*m*/*z*) [M + H]^+^ calcd for C_22_H_19_ClN_3_O: 376.1217, found: 376.1213.

#### 1-(4-Chlorophenyl)-5-(2-fluoro-4-((2-methylbenzyl)oxy)phenyl)-1H-pyrazole (**88**)

It was synthesized from compound **83** using 4-chlorophenylhydrazine.HCl according to synthesis method 8a. The resulting crude product was purified by flash column chromatography (0% → 50% EtOAc in Hexane). Yield 66.0%; mp 131.8–132.8 °C. ^1^H NMR (400 MHz, CDCl_3_): δ_H_ 2.39 (3H, s), 5.03 (2H, s), 6.51 (1H, d, *J* = 2.0 Hz), 6.71 (1H, dd, *J* = 11.6 Hz, 2.4 Hz), 6.78 (1H, dd, *J* = 8.4 Hz, 2.4 Hz), 7.13 (1H, dd, *J* = 8.8, 8.4 Hz), 7.22–7.32 (7H, m), 7.40 (1H, m), 7.75 (1H, d, *J* = 2.0 Hz). ^13^C NMR (100 MHz, CDCl_3_): δ_C_ 19.07, 69.23, 103.11 (d, ^2^*J*_C-F_ = 25.1 Hz), 109.51 (d, ^4^*J*_C-F_ = 1.5 Hz), 111.12 (d, ^2^*J*_C-F_ = 15.3 Hz), 111.24 (d, ^3^*J*_C-F_ = 3.1 Hz), 125.57, 126.32, 128.84, 128.89, 129.22, 130.74, 131.90 (d, ^3^*J*_C-F_ = 4.6 Hz), 133.12, 133.98, 136.94, 137.06, 139.05, 140.68, 160.23 (d, ^1^*J*_C-F_ = 248.4 Hz), 160.98 (d, ^2^*J*_C-F_ = 10.6 Hz). HRMS (*m*/*z*) [M + H]^+^ calcd for C_23_H_19_ClFN_2_O: 393.1170, found: 393.1172.

#### 1-(4-Chlorophenyl)-5-(3-fluoro-4-((2-methylbenzyl)oxy)phenyl)-1H-pyrazole (**89**)

It was synthesized from compound **84** using 4-chlorophenylhydrazine.HCl according to synthesis method 8a. The resulting crude product was purified by flash column chromatography (0% → 30% EtOAc in Hexane). Yield 43.0%; mp 95.0–95.4 °C. ^1^H NMR (400 MHz, CDCl_3_): δ_H_ 2.40 (3H, s), 5.12 (2H, s), 6.46 (1H, d, *J* = 1.6 Hz), 6.88-6.91 (1H, m), 6.96–7.02 (2H, m), 7.20–7.27 (5H, m), 7.32–7.35 (2H, m), 7.40–7.41 (1H, m), 7.71 (1H, d, *J* = 1.6 Hz).^13^C NMR (100 MHz, CDCl_3_): δ_C_ 19.07, 70.17, 108.23, 115.62 (d, ^3^*J*_C-F_ = 2.0 Hz), 116.93 (d, ^2^*J*_C-F_ = 19.2 Hz), 123.75 (d, ^3^*J*_C-F_ = 7.1 Hz), 125.02 (d, ^4^*J*_C-F_ = 3.8 Hz), 126.24, 126.46, 128.76, 128.82, 129.35, 130.68, 133.46, 134.09, 136.92, 138.62, 140.77, 141.91 (d, ^4^*J*_C-F_ = 1.9 Hz), 147.18 (d, ^2^*J*_C-F_ = 10.3 Hz), 152.67 (d, ^1^*J*_C-F_ = 246.2 Hz). HRMS (*m*/*z*) [M + H]^+^ calcd for C_23_H_19_ClFN_2_O: 393.1170, found: 393.1173.

#### N-(4-(1-(4-Chlorophenyl)-1H-pyrazol-5-yl)phenyl)-2-methylbenzamide (**91**)

*o*-Toluic acid was dissolved in dichloromethane, then oxalyl chloride and a catalytic amount of DMF were added, and it was stirred for 1 h at rt in a nitrogen atmosphere. At the end of the reaction, the reaction mixture was evaporated. The residue was dissolved in dichloromethane and then DIEA, and compound **90** was added and stirred at rt overnight. It was extracted with dichloromethane; the organic layer was dried with anhydrous Na_2_SO_4_ and evaporated. The resulting crude product was purified by flash column chromatography (0% → 40% EtOAc in Hexane). Yield 63.0%; mp 179.1–180.0 °C. ^1^H NMR (400 MHz, CDCl_3_): δ_H_ 2.48 (3H, s), 6.48 (1H, d, *J* = 1.6 Hz), 7.18–7.37 (9H, m), 7.44 (1H, d, *J* = 7.6 Hz), 7.57 (2H, d, *J* = 8.4 Hz), 7.69 (1H, d, *J* = 1.6 Hz), 7.70 (1H, m). ^13^C NMR (100 MHz, CDCl_3_): δ_C_ 19.82, 108.02, 119.74, 125.92, 126.22, 126.31, 126.59, 129.12, 129.48, 130.47, 131.34, 133.15, 136.05, 136.52, 138.22, 138.57, 140.60, 142.55, 168.14. HRMS (*m*/*z*) [M + H]^+^ calcd for C_23_H_19_ClN_3_O: 388.1217, found: 388.1213.

#### 4-(1-(4-Chlorophenyl)-1H-pyrazol-5-yl)-N-(2-methylbenzyl)aniline (**92**)

It was synthesized from compound **90** according to synthesis method 1c. The resulting crude product was purified by preparative column chromatography (0% → 20% ACN in Water). Yield 32.0%; mp 141.5–142.8 °C. ^1^H NMR (400 MHz, CDCl_3_): δ_H_ 2.36 (3H, s), 4.27 (2H, s), 6.39 (1H, d, *J* = 1.2 Hz), 6.56 (2H, d, *J* = 8.4 Hz), 7.03 (2H, d, *J* = 8.8 Hz), 7.18–7.32 (8H, m), 7.67 (1H, d, *J* = 1.2 Hz). ^13^C NMR (100 MHz, CDCl_3_): δ_C_ 18.93, 46.23, 107.13, 112.54, 119.15, 126.21, 126.25, 127.66, 128.29, 128.94, 129.85, 130.52, 132.69, 136.29, 136.35, 139.00, 140.49, 143.55, 148.07. HRMS (*m*/*z*) [M + H]^+^ calcd for C_23_H_21_ClN_3_: 374.1424, found: 374.1425.

### Biological Studies

#### Cell Culture

HepG2, FOCUS, Hep3B, Mahlavu, Huh7, Hep40, and PLC-PRF-5 hepatocellular carcinoma cells; MCF-7, MDA-MB-231, MDA-MB-468, SKBR3, and ZR-75 breast cancer cells; and normal-like epithelial MCF-10A breast cells were cultured in low-glucose Dulbecco’s modified Eagle’s medium (DMEM) (Biological Industries-BI) supplemented with 10% fetal bovine serum (FBS) (Gibco/Thermo Fisher Scientific), 2 mM l-Glutamine (Gibco/Thermo Fisher Scientific), 100 Units/mL Penicillin/Streptomycin, and 0.1 mM no*n-*essential amino acid (Gibco/Thermo Fisher Scientific). SNU475 hepatocellular carcinoma cells were maintained in RPMI (Biological Industries-BI) supplemented with 10% fetal bovine serum (FBS) (Gibco/Thermo Fisher Scientific), l-Glutamine (Gibco/Thermo Fisher Scientific), and 100 Units/mL Penicillin/Streptomycin, while ZR-75 breast cancer cells were cultured RPMI (Biological Industries-BI) supplemented with 10% fetal bovine serum (FBS) (Gibco/Thermo Fisher Scientific), 1× sodium pyruvate, and %4.5 glucose. Normal-like MCF-10A breast cells were sustained in DMEM/HAM’S F12 (Hyclone) supplemented with 10% fetal bovine serum (FBS) (Gibco/Thermo Fisher Scientific), EGF (20 ng/mL), Hydrocortisone (0.5 mg/mL), cholera toxin (100 ng/mL), insulin (10 μg/mL), and 100 Units/mL Penicillin/Streptomycin. The cells were cultivated at 37 °C in a humidified incubator under 5% CO_2_.

#### NCI-60 Sulforhodamine B (SRB) Cytotoxicity Assay

Mahlavu, FOCUS, SNU475 (1000 cell/well in 150 μL/well); Huh7, MCF7 (2500 cell/well in 150 μL/well); HepG2, Hep3B, SKBR3 (3000 cell/well in 150 μL/well); PLC-PRF-5, Hep40 (5000 cell/well in 150 μL/well); MDA-MB-231, MDA-MB-468, MCF10A (6000 cell/well in 150 μL/well); and ZR-75 (7500 cell/well in 150 μL/well) were cultured in 96-well plates and were inoculated in an incubator for 24 hours. The compounds were dissolved in dimethyl sulfoxide (DMSO) (Sigma, St Louis, MO) as a 20 mM stock solution. The treatment of cells with the compounds was done in a concentration range starting from 40 to 2.5 μM. The compounds which were below 2.5 μM were tested in a concentration range from 2.5 to 0.15 μM. End of the 72h compound treatment, the cells were fixed using 10% (v/v) trichloroacetic acid (MERCK) for an hour and washed with ddH_2_O, and left for air-drying. The fixed plates were stained with 50 μL of sulforhodamine B (SRB) solution (Sigma) at RT for 10 min. To remove unbound SRB dye, acetic acid was used to wash cells three times and left for drying. The protein-bound SRB was solubilized with 10 mM Tris-base (Sigma) and their absorbance was measured with a 96-well plate reader at 515 nm wavelength (ELx800, Biotek). The IC_50_ values of compounds were calculated in comparison with control group DMSO. Data with *R*^2^ values larger than 0.9 are considered significant.

#### Real-Time Cell Growth Surveillance by Electronic Sensing (RT-CES)

To monitor the real-time cell growth of cells and cytotoxicity of compounds, the xCELLigence system (Roche Applied Sciences) was used. The cells were seeded onto 96-well E-Plate Mahlavu (1500 cell/well), MCF7, Huh7 (2500 cell/well), MDA-MB-231 (5000 cell/well), and MCF10A (6000 cell/well) in 100 μL of the medium/well. To examine the proliferation of cells, the cellular growth was monitored as cell index (CI) every 30 min for 24 h. When the cells were in a log growth phase, treatment with compounds **11** and **85** in 2.5, 5, and 10 μM concentrations was done, the CI values were recorded every 10 min for 24 h for controlling fast drug response and then every 30 min for the remaining 48 h for long-term dose response. After 72 h of incubation, the cellular growth ratios were calculated by CI_Drug_/CI_DMSO_.

#### Cell Death Analysis with Flow Cytometry

Mahlavu, Huh7, MCF7, and MDA-MB-231 were seeded onto six-well plates. After 24 h, the cells were incubated with 5 μM compounds **11** and **85** by keeping DMSO as a negative control. After 48 h incubation, the cells were collected to identify programmed cellular death with Annexin V assay. PI and Annexin V dyes were added onto cell lysates for 15 min at room temperature in the dark to stain the cells for detecting apoptotic cell populations. The cells were analyzed by flow cytometry.

#### Immunofluorescence Staining for the Detection of Cell Death

Mahlavu, Huh7, MCF7, and MDA-MB-231 cells were cultured onto cover slides in six-well plates for 24 h according to their cell growth rate. The cells were treated with compounds **11** and **85** with their IC_100_ concentrations or DMSO as a negative control for 48 h. The 1× PBS was used to wash the cells three times, and the cells were fixed with 100% ice-cold methanol. Then, the cells were stained with 1 μg/mL Hoechst (#33258, Sigma). The cells were analyzed under the fluorescence microscope (Nikon Eclipse 50i).

#### Western Blotting

Cells were treated for 5 μM compounds **11** and **85** or control (DMSO). The treated cells were collected with a scraper at end of 48 h incubation and lysates were prepared. Protein concentration was calculated with a BCA assay. The protein levels were analyzed using protein electrophoresis according to the manufacturer’s protocol for near-infrared (NIR) Western Blot analysis (Mini-PROTEAN Tetra Cell Systems, Bio-Rad). The samples (40 μg /well) were loaded onto TGX precast gels, and protein transfer from gel to an LF-PVDF membrane was done using Trans-Blot Turbo System (Bio-Rad). Primary antibodies (PARP, CST, #9532S) and Calnexin (CST, #2679) and secondary antibodies IRDye 680RD Goat Anti Rabbit (Li-Cor, # 92668071) and IRDye- 800CW Goat Anti Rabbit (Licor, # 926e32211) were used for western blotting, and the proteins were monitored in fluorescence system using Odyssey CLx- LICOR imaging system.^32^

#### *In Vivo* Mouse Xenograft Experiments

Mahlavu cells (10 × 10^6^ cells/mouse) prepared in 150 μL of DMEM were injected subcutaneously to the flank of 6–8 weeks old male athymic nude mice, while MDA-MB-231 cells (2 × 10^6^cells/mouse) in 1:1 DMEM and matrigel were injected into mammary fat pad (MFP) of 6–8 weeks old female athymic nude mice. Tumor volume and mouse weight were measured twice a week using calipers. When the tumor volume reached ∼150 mm^3^, xenografts were randomized into groups (6 mice per group), and the mice were administered subsequently with **11** or **85** (40 mg/kg in 0.5% hydroxypropyl methylcellulose plus 1% Tween 80) or vehicle by gavage feeding (3–4 days/week) for 4 weeks. The mice were sacrificed 30 days after initiation of the compound treatment and tumors were collected. Two-way ANOVA was performed for statistical significance by GraphPad Prism. All of the mice were maintained under a temperature-controlled environment with a 12 h light/dark cycle and received a standard diet and water ad libitum.

#### Statistical Analysis

All data were obtained from three independent experiments and standard deviation (S.D) values were accessed. All experiments except western blotting were done two times with *n* ≥ 3 biological replicates. One-way ANOVA and two-way ANOVA were applied using GraphPad (Prism) for statistical analysis. Results were shown as follows: ns: not significant, *p < 0.05, **p < 0.01, ***p < 0.001, and ****p < 0.0001.

## Supporting Information Available

Chemical procedures and experimental data of intermediates, NCI-60 cancer cell panel results for compounds **11** and **85**, X-ray data, PK and *in vivo* test data, and copies of ^1^H and ^13^C NMR spectra of final compounds (PDF)
