# Supplementary material for: Vicinal Diaryl-Substituted Isoxazole and Pyrazole Derivatives with In Vitro Growth Inhibitory and In Vivo Antitumor Activity
Source: ACS Omega. 2022 Oct 3;7(41):36206–26. doi: 10.1021/acsomega.2c03405 (PMC9583322; doi:10.1021/acsomega.2c03405)

Supporting Information

Vicinal diaryl-substituted isoxazole and pyrazole derivatives with *in vitro* growth inhibitory and *in vivo* antitumor activity

Sümeyye Turanlı [a,†], Esra Nalbat [b], Deniz Lengerli [a], Kübra İbiş [a], Sezen Güntekin Ergün [b,‡], Ece Akhan Güzelcan [b,§], Mesut Muyan [c], Rengul Cetin-Atalay [b,ϕ], Burcu Çalışkan [a], Erden Banoglu [a,*]

[a] Department of Pharmaceutical Chemistry, Faculty of Pharmacy, Gazi University, Yenimahalle, 06560, Ankara, Turkey

[b] Cancer Systems Biology Laboratory, Graduate School of Informatics, Middle East Technical University, Ankara 06800, Turkey

[c] Department of Biological Sciences, Middle East Technical University, Ankara 06800, Turkey

[†] Department of Pharmaceutical Chemistry, Faculty of Pharmacy, Adıyaman University, Adıyaman, Turkey.

[‡] Department of Medical Biology, Hacettepe University, Ankara 06800, Turkey.

[§] Center for Genomics and Rare Diseases & Biobank for Rare Diseases, Hacettepe University, Ankara, 06800, Turkey.

[ϕ] Section of Pulmonary and Critical Care Medicine, University of Chicago, Chicago, Illinois 60637, USA.

Table of Contents

General synthetic procedures (Methods 1-9)…… S2

Scheme S1 (Synthesis of Compounds **C1-C3, 9**)…… S8

Scheme S2 (Synthesis of Compounds **C4a-c**, **C5a-c**, **43a-c**)…… S9

Scheme S3 (Synthesis of Compounds **C6-C8, 58**)…… S11

Scheme S4 (Synthesis of Compounds **C9-61**)…… S12

Scheme S5 (Synthesis of Compounds **C10-C12, 64**)…… S13

Scheme S6 (Synthesis of Compounds **C13, 66**)…… S15

Scheme S7 (Synthesis of Compounds **C14, 68**)…… S15

Scheme S8 (Synthesis of Compounds **C15, C16, 70, 73, 74**)…… S16

Scheme S9 (Synthesis of Compounds **C17-C20, 76, 77**)…… S18

Scheme S10 (Synthesis of Compounds **C21-C24, 80-84**)…… S20

Scheme S11 (Synthesis of Compounds **C25, C26, 90**)…… S22

Table S1. *In vitro* growth inhibitory values of **11** and **85** at 10 µM in NCI60 cancer cell panel…… S24

Figure S1. ORTEP drawings of the single crystal structures of compound **11** and **85**…… S26

Figure S2. Plasma concentrations vs. time profile of **85** after single dose IV (1mg/kg) and PO (10 mg/kg) administrations in male CD-1 mice…… S27

Figure S3. Representative images of dissected tumors from the xenograft models used in the study…… S27

References……………………………………………………………………………. S28
Figure S4. ^{1}H-NMR and ^{13}C-NMR spectrum of Compound **9**…………………………. S31
Figure S5. ^{1}H-NMR and ^{13}C-NMR spectrum of Compound **11**………………………... S33
Figure S6. ^{1}H-NMR and ^{13}C-NMR spectrum of Compound **12**………………………... S35
Figure S7. ^{1}H-NMR and ^{13}C-NMR spectrum of Compound **13**………………………... S37
Figure S8. ^{1}H-NMR and ^{13}C-NMR spectrum of Compound **14**……………………… S39
Figure S9. ^{1}H-NMR and ^{13}C-NMR spectrum of Compound **15**……………………… S41
Figure S10. ^{1}H-NMR and ^{13}C-NMR spectrum of Compound **16**…………………….. S43
Figure S11. ^{1}H-NMR and ^{13}C-NMR spectrum of Compound **17**…………………….. S45
Figure S12. ^{1}H-NMR and ^{13}C-NMR spectrum of Compound **18**………………………. S47
Figure S13. ^{1}H-NMR and ^{13}C-NMR spectrum of Compound **19**………………………. S49
Figure S14. ^{1}H-NMR and ^{13}C-NMR spectrum of Compound **20**………………………. S51
Figure S15. ^{1}H-NMR and ^{13}C-NMR spectrum of Compound **21**…………………….. S53
Figure S16. ^{1}H-NMR and ^{13}C-NMR spectrum of Compound **22**………………………. S55
Figure S17. ^{1}H-NMR and ^{13}C-NMR spectrum of Compound **23**…………………….. S57
Figure S18. ^{1}H-NMR and ^{13}C-NMR spectrum of Compound **24**…………………….. S59
Figure S19. ^{1}H-NMR and ^{13}C-NMR spectrum of Compound **25**………………………. S61
Figure S20. ^{1}H-NMR and ^{13}C-NMR spectrum of Compound **26**…………………….. S63
Figure S21. ^{1}H-NMR and ^{13}C-NMR spectrum of Compound **27**…………………….. S65
Figure S22. ^{1}H-NMR and ^{13}C-NMR spectrum of Compound **28**…………………….. S67
Figure S23. ^{1}H-NMR and ^{13}C-NMR spectrum of Compound **29**…………………….. S69
Figure S24. ^{1}H-NMR and ^{13}C-NMR spectrum of Compound **30**…………………….. S71
Figure S25. ^{1}H-NMR and ^{13}C-NMR spectrum of Compound **31**…………………….. S73
Figure S26. ^{1}H-NMR and ^{13}C-NMR spectrum of Compound **32**…………………….. S75
Figure S27. ^{1}H-NMR and ^{13}C-NMR spectrum of Compound **33**………………………. S77
Figure S28. ^{1}H-NMR and ^{13}C-NMR spectrum of Compound **34**………………………. S79
Figure S29. ^{1}H-NMR and ^{13}C-NMR spectrum of Compound **35**………………………. S81
Figure S30. ^{1}H-NMR and ^{13}C-NMR spectrum of Compound **36**…………………….. S83
Figure S31.^{1}H-NMR and ^{13}C-NMR spectrum of Compound **37**……………………….. S85
Figure S32.^{1}H-NMR and ^{13}C-NMR spectrum of Compound **38**……………………….. S87

Figure S33.^{1}H-NMR and ^{13}C-NMR spectrum of Compound **39**……………………….. S89
Figure S34. ^{1}H-NMR and ^{13}C-NMR spectrum of Compound **40**…………………….. S91
Figure S35. ^{1}H-NMR and ^{13}C-NMR spectrum of Compound **41**……………………… S93
Figure S36. ^{1}H-NMR and ^{13}C-NMR spectrum of Compound **42**……………………… S95
Figure S37. ^{1}H-NMR and ^{13}C-NMR spectrum of Compound **44**……………………… S97
Figure S38. ^{1}H-NMR and ^{13}C-NMR spectrum of Compound **45**……………………… S99
Figure S39. ^{1}H-NMR and ^{13}C-NMR spectrum of Compound **46**……………………… S101
Figure S40. ^{1}H-NMR and ^{13}C-NMR spectrum of Compound **47**……………………… S103
Figure S41. ^{1}H-NMR and ^{13}C-NMR spectrum of Compound **48**……………………… S105
Figure S42. ^{1}H-NMR and ^{13}C-NMR spectrum of Compound **49**……………………… S107
Figure S43. ^{1}H-NMR and ^{13}C-NMR spectrum of Compound **50**……………………… S109
Figure S44. ^{1}H-NMR and ^{13}C-NMR spectrum of Compound **51**……………………… S111
Figure S45. ^{1}H-NMR and ^{13}C-NMR spectrum of Compound **52**……………………… S113
Figure S46. ^{1}H-NMR and ^{13}C-NMR spectrum of Compound **53**……………………… S115
Figure S47. ^{1}H-NMR and ^{13}C-NMR spectrum of Compound **54**……………………… S117
Figure S48. ^{1}H-NMR and ^{13}C-NMR spectrum of Compound **55**……………………… S119
Figure S49. ^{1}H-NMR and ^{13}C-NMR spectrum of Compound **56**……………………… S121
Figure S50. ^{1}H-NMR and ^{13}C-NMR spectrum of Compound **57**……………………… S123
Figure S51. ^{1}H-NMR and ^{13}C-NMR spectrum of Compound **59**……………………… S125
Figure S52. ^{1}H-NMR and ^{13}C-NMR spectrum of Compound **63**……………………… S127
Figure S53. ^{1}H-NMR and ^{13}C-NMR spectrum of Compound **65**……………………… S129
Figure S54. ^{1}H-NMR and ^{13}C-NMR spectrum of Compound **67**……………………… S131
Figure S55. ^{1}H-NMR and ^{13}C-NMR spectrum of Compound **69**……………………… S133
Figure S56. ^{1}H-NMR and ^{13}C-NMR spectrum of Compound **71**……………………… S135
Figure S57. ^{1}H-NMR and ^{13}C-NMR spectrum of Compound **72**……………………… S137
Figure S58. ^{1}H-NMR and ^{13}C-NMR spectrum of Compound **75**……………………… S139
Figure S59. ^{1}H-NMR and ^{13}C-NMR spectrum of Compound **78**……………………… S141
Figure S60. ^{1}H-NMR and ^{13}C-NMR spectrum of Compound **79**……………………… S143
Figure S61. ^{1}H-NMR and ^{13}C-NMR spectrum of Compound **85**……………………… S145
Figure S62. ^{1}H-NMR and ^{13}C-NMR spectrum of Compound **86**……………………… S147

Figure S63. ^{1}H-NMR and ^{13}C-NMR spectrum of Compound **87**……………………… S149

Figure S64. ^{1}H-NMR and ^{13}C-NMR spectrum of Compound **88**……………………… S151

Figure S65. ^{1}H-NMR and ^{13}C-NMR spectrum of Compound **89**……………………… S153

Figure S66. ^{1}H-NMR and ^{13}C-NMR spectrum of Compound **91**……………………… S155

Figure S67. ^{1}H-NMR and ^{13}C-NMR spectrum of Compound **92**……………………… S157

General Synthetic Procedures

Method 1: Synthesis Methods of Compounds Obtained by Alkylation Reaction

*Method 1a (Compound **C1**, **11-28**, **30-42**, **C4b-c**, **C14**, **75**, **78**, **79**):* The corresponding phenol derivative (1.0 eq) and the appropriate benzyl bromide/chloride derivative (1.2 eq) and K_2CO_3 (1.7 eq) were dissolved in acetonitrile (DMF was preferred as solvent when benzylchloride derivatives and KI (0.2 eq) was added to the reaction and the amount of K_2CO_3 (3.0 eq) was increased when HCl salts of aryl chloride derivatives were used) and heated under reflux. After 3h, ice-water mixture was added to the reaction flask and the precipitated solid was filtered and evaporated to give crude product.

*Method 1b (Compound **C4a**, **63**, **C10**, **70**, **C21**, **C23**, **C25** and **C29**):* The corresponding phenol derivative (1.0 eq) and the appropriate benzyl bromide derivative (1.2 eq) and K_2CO_3 (1.7 eq) were taken into a microwave vial and acetonitrile was added. The reaction was heated by microwave irradiation at 120°C for 20 min. The reaction mixture was cooled to rt, poured into water, and then extracted with ethyl acetate. The organic layer was dried, filtered, and evaporated to give the crude product.

*Method 1c (Compound **59** and **92**):* To the solution of the Compound **58** or **90** (1.0 eq) in DMF was added N,N-diisopropylethylamine (DIEA) (1.0 eq), and 2-methylbenzyl bromide (1.0 eq). The reaction was stirred under reflux at 65°C for 5h. At the end of the reaction, the mixture was poured into water. The precipitate formed was washed with water and filtered under vacuum.

Method 2: Synthesis Method of Oxime Derivatives Obtained from Aldehyde Starting Materials (Compound C2, C5a-c, C6, C13)

$NH_2OH.HCl$ (1.1 eq) was added to the solution of the related aldehyde derivative (1.0 eq) in ethanol: ice: water mixture, then NaOH 50% (w/v) solution (2.5 eq) was added dropwise. It was stirred at room temperature for 2h. After completion of the reaction, water was added and acidified with concentrated HCl. The precipitate formed was filtered under vacuum to dry.

Method 3: Synthesis Method of Compounds Obtained by Chlorination of Oxime Derivatives (Compound C3, 43a-c, C7, 66)

N-Chlorosuccinimide (NCS) (1.0 eq) was added in the solution of oxime derivative (1.0 eq) in DMF. The reaction was catalyzed by HCl gas and stirred at room temperature for 3h. Then the reaction mixture was poured into ice-water and formed precipitate was filtrated under vacuum and dried.

Method 4: Synthesis Method of Isoxazole Derivatives Obtained from Nitrile Oxide Intermediate (Compound 9, 44-57, C7, 67)

To the solution of the related chlorinated oxime (1.0 eq) in diethyl ether, Et_3N (1.0 eq) was added and stirred at 0°C for 2h and the precipitate formed was filtered. The filtrate was evaporated in vacuo to form aryl nitrile oxide. NaH (1.6 eq) was dissolved in dry tetrahydrofuran (THF) under nitrogen atmosphere at 0°C. Phenylacetone derivative (1.0 eq) was added dropwise and mixed for 1h. At the end of the time, arylnitrileoxide (1.0 eq) was dissolved in dry THF and added to the reaction mixture and stirred at rt overnight under nitrogen atmosphere. The reaction mixture was quenched with 2M NH_4Cl and extracted with ethyl acetate. The organic phase was washed with water, brine and dried over anhydrous Na_2SO_4 and concentrated to give product.

Method 5: Synthesis Method of Compounds Obtained by Reduction of Nitro Group (Compound 51, 58 and 90)

The nitro derivative (1.0 eq) was dissolved in methanol, added $SnCl_2.2H_2O$ and heated under reflux overnight. Excess of solvent was evaporated then dilueted with water, partitioned between $NaHCO_3$ solution and ethyl acetate. The organic layer dried and evaporated and the

crude product was purified by automated flash chromatography using RediSep Silica columns (12g), eluting with hexane:ethyl acetate (60:40) as mobile phase.

Method 6: Synthesis Methods of Compounds Obtained by Demethylation

*Method 6a (Compound **62, 74, 76** and **77**):* Starting material containing methoxy group (1.0 eq) was dissolved in dichloromethane under nitrogen atmosphere and 1M BBr_3 solution in dichloromethane (3.0 eq) was added dropwise and stirred for 4h at rt under dry conditions. Then it was quenched by methanol (1ml), extracted with dichloromethane, the organic layer was dried and evaporated.

*Method 6b (Compound **C15**):* To a solution of compound **C9** (1.0 eq) in acetic acid, HBr (16.5 eq) was added. The reaction was heated under reflux overnight. The reaction mixture was dilueted with water, neutralized, and the precipitate was filtered and dried.

Method 7: Synthesis Method of Intermediate Compounds Obtained Using DMFDMA Reagent (Compound C17, 80-84 and C25)

Acetophenone derivative (1.0 eq) and N,N-dimethylformamide dimethylacetal (DMFDMA) (15.0 eq) were dissolved in toluene and heated overnight in a sealed tube at 130 °C. Then the reaction mixture was evaporated, and the crude product was used in the next step without purification.

Method 8: Synthesis Methods of Pyrazole Derivatives

*Method 8a (Compounds **C18, 85-89** and **C26**):* Enaminone derivative (1.0 eq) and the hydrazine derivative (1.0 eq) were dissolved in absolute ethanol, heated under reflux for 4h. After completion of the reaction, ethanol was evaporated, reaction mixture was extracted with dichloromethane, the organic layer was dried and evaporated. The crude product was purified by automated flash chromatography using RediSep Silica columns (12g), eluting with hexane:ethyl acetate gradient as mobile phase.

*Method 8b (Compound **71** and **72**):* A solution of compound **70** (1.0 eq) and DMFDMA (5.0 eq) in DMF was stirred at rt overnight. The reaction mixture was evaporated to obtain the *2-(4-*

chlorophenyl)-3-(dimethylamino)-1-(4-((2-methylbenzyl)oxy)phenyl)prop-2-en-1-one, which was then dissolved in methanol and heated with hydrazine derivative (1.5 eq) under reflux for 2h. The reaction mixture was poured into water, extracted with ethyl acetate, the organic layer was dried and evaporated under vacuum. The crude product was purified by automated flash chromatography using RediSep Silica columns (12g), eluting with hexane:ethyl acetate as mobile phase. (For Compound **72**, a catalytic amount of sulfuric acid was added to the reaction mixture.)

*Method 8c (Compound **69** and **C20**):* Compound **68** or **C19** (1.0 eq) and the appropriate hydrazine derivative (1.1 eq) were dissolved in methanol, Et_3N (1.0 eq) was added then heated under reflux for 4h. Excess of the solvent was evaporated. The reaction mixture was poured into water and extracted with ethyl acetate, the organic layer was dried and evaporated. The crude product was purified by automated flash chromatography using RediSep Silica columns (24g), eluting with hexane:ethyl acetate (70:30) as mobile phase.

Method 9: General Synthesis Method of Biotinylated Derivatives (Compound 93, 94, 95 and 96)

Compound **10** (for **93**), compound **51** (for **94**) or compound **C32** (for **95** and **96**) (1.0 eq) was dissolved in DMF after then biotin or PEG-biotin (5.9 eq), 1-ethyl-3-(3-dimethylaminopropyl)carbodiimide (EDCI) (8.9 eq) and 4-dimethylaminopyridine (DMAP) (2.2 eq) were added. The reaction was stirred at rt overnight.[1] At the end of the time, the mixture was poured into water and neutralized with $NaHCO_3$, then extracted with ethyl acetate, dried with anhydrous Na_2SO_4.

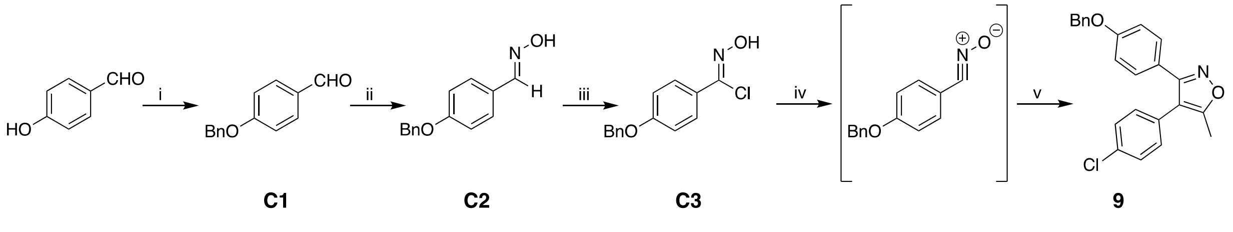

Scheme S1. Reactions conditions and reagents: i) benzyl chloride, K_2CO_3, DMF, 80°C; ii) NH_2OH•HCl, NaOH, H_2O, EtOH, rt; iii) NCS, DMF, rt; iv) TEA, diethyl ether, 0°C; v) 4-chlorophenylacetone, NaH, THF, 0°C.

4-(Benzyloxy)benzaldehyde (C1) [CAS: 4397-5-9]

It was synthesized from 4-hydroxybenzaldehyde using benzyl chloride according to the synthesis method 1a. Yield 82.0%; mp 70.9-73.0°C (lit. mp 71.0-72.0°C).[2] HRMS (m/z) $[M+H]^+$ calcd for $C_{14}H_{13}O_2$: 213.0916, found: 213.0907.

4-(Benzyloxy)benzaldehyde oxime (C2) [CAS: 76193-67-4]

It was synthesized from compound **C1** according to the synthesis method 2. Yield 98.0%; mp 108.5-110.6°C (lit. mp 109.0-111.0°C).[3] HRMS (m/z) $[M+H]^+$ calcd for $C_{14}H_{14}NO_2$: 228.1025, found: 228.1001.

4-(Benzyloxy)-N-hydroxybenzimidoyl chloride (C3) [CAS: 188038-42-8]

It was synthesized from compound **C2** according to the synthesis method 3. Yield 86%; mp 86.7-88.1°C (lit. mp 83.0-84.0°C).[4] HRMS (m/z) $[M+H]^+$ calcd for $C_{14}H_{13}ClNO_2$: 262.0635, found: 262.0621.

4-(4-(4-Chlorophenyl)-5-methylisoxazol-3-yl)phenol (10)

To the solution of compound **C3** (1.0 eq) in anhydrous ethanol was added concentrated HCl (1.0 eq), and 10% of the starting material amount of Pd/C (10% w/w) was added and stirred under H_2 atmosphere for 3h.[5, 6] The reaction mixture was filtered through celite pad, and the filtrate was evaporated in vacuo. The resulting crude product was purified by automated-flash chromatography using RediSep Silica columns (12g), eluting with hexane:ethyl acetate (70:30) as mobile phase. Yield 79.0%; mp 200.0-202.5°C. ^{1}H NMR (400 MHz, DMSO-d_6): δ_H 2.38 (3H, s), 6.74 (2H, d, J = 8.6 Hz), 7.14 (2H, d, J = 8.6 Hz), 7.21 (2H, d, J = 8.8 Hz), 7.45 (2H, d, J = 8.8 Hz), 9.79 (1H, s). ^{13}C-NMR (100 MHz, DMSO-d$_6$): δ_C 11.23, 113.84, 115.52, 118.93,

128.77, 128.95, 129.46, 131.35, 132.44, 158.66, 160.32, 166.67. HRMS (m/z) [M+H]+ calcd for $C_{16}H_{13}ClNO_2$: 286.0635, found: 286.0633.

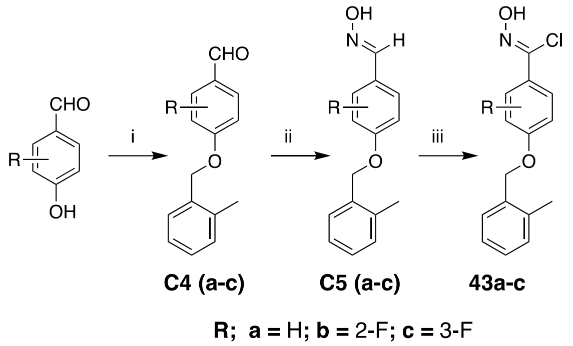

Scheme S2. Reactions conditions and reagents: i) 2-methylbenzyl bromide, K_2CO_3, MeCN, MWI, 120°C, 20 min (for compound C4a) or benzyl bromide derivative, K_2CO_3, MeCN, Δ (for compound C4b-c); ii) NH_2OH•HCl, NaOH, H_2O, EtOH, rt; iii) NCS, DMF, rt.

4-((2-Methylbenzyl)oxy)benzaldehyde (C4a) [CAS: 400825-69-6]

It was synthesized from 4-hydroxybenzaldehyde using 2-methylbenzyl bromide according to the synthesis method 1b. Yield 77.0%; mp 60.6-61.5°C (lit. mp 60.1-61.2°C).[7] HRMS (m/z) $[M+H]^+$ calcd for $C_{15}H_{14}O_2$: 227.1067, found: 227.1072.

2-Fluoro-4-((2-methylbenzyl)oxy)benzaldehyde (C4b)

It was synthesized from 2-fluoro-4-hydroxybenzaldehyde using 2-methyl benzyl bromide according to the synthesis method 1a. Yield 47.0%; mp 83.6-85.3°C. ^1H NMR (400 MHz, DMSO-d_6): δ_H 2.32 (3H, s), 5.23 (2H, s), 7.05 (1H, dd, J = 8.4, 2.2 Hz), 7.14 (1H, dd, J = 13.2, 2.0 Hz), 7.20-7.30 (3H, m), 7.42 (1H, d, J = 7.2 Hz), 7.80 (1H, t, J = 8.6 Hz), 10.08 (1H, s). HRMS (m/z) $[M+H]^+$ calcd for $C_{15}H_{14}O_2F$: 245.0978, found: 245.096.

3-Fluoro-4-((2-methylbenzyl)oxy)benzaldehyde (C4c) [CAS: 1916189-40-6]

It was synthesized from 3-fluoro-4-hydroxybenzaldehyde using 2-methyl benzyl bromide according to the synthesis method 1a. Yield 80.0%; mp 52.6-53.5°C. HRMS (m/z) $[M+H]^+$ calcd for $C_{15}H_{14}O_2F$: 245.0978, found: 245.0985.

4-((2-Methylbenzyl)oxy)benzaldehyde oxime (C5a) [CAS: 1331635-71-2]

It was synthesized from compound **C4a** according to the synthesis method 2. Yield 96.0%; mp 108.9-109.5°C. ^{1}H-NMR ($CDCl_3$): δ_H 2.30 (3H, s), 5.09 (2H, s), 7.03 (2H, d, *J* = 8.6 Hz), 7.16-7.25 (3H, m), 7.39 (1H, d, *J* = 6.8 Hz), 7.51 (2H, d, *J* = 8.6 Hz), 8.05 (1H, s), 10.95 (1H, s). HRMS (m/z) $[M+H]^+$ calcd for $C_{15}H_{15}NO_2$: 242.1173, found: 242.1181.

2-Fluoro-4-((2-methyl benzyl)oxy)benzaldehyde oxime (C5b)

It was synthesized from compound **C4b** according to synthesis method 2. Yield 96.0%; mp 129.1-130.4°C. ^{1}H NMR (400 MHz, DMSO-d_6): δ_H 2.32 (3H, s), 5.13 (2H, s), 6.92 (1H, dd, *J* = 8.8, 2.0 Hz), 7.02 (1H, dd, *J* = 12.8, 2.4 Hz), 7.18-7.28 (3H, m), 7.41 (1H, d, *J* = 7.2 Hz), 7.66 (1H, t, *J* = 8.6 Hz), 8.14 (1H, s), 11.32 (1H, s). HRMS (m/z) $[M+H]^+$ calcd for $C_{15}H_{15}NO_2F$: 260.1087, found: 260.1083.

3-Fluoro-4-((2-methylbenzyl)oxy)benzaldehyde oxime (C5c)

It was synthesized from compound **C4c** according to synthesis method 2. Yield 92.0%; mp 113.5-115.3°C. ^{1}H NMR (400 MHz, DMSO-d_6): δ_H 2.34 (3H, s), 5.19 (2H, s), 7.19-7.45 (7H, m), 8.08 (1H, s), 11.17 (1H, s). HRMS (m/z) $[M+H]^+$ calcd for $C_{15}H_{15}NO_2F$: 260.1087, found: 260.1074.

***N*-hydroxy-4-((2-methylbenzyl)oxy)benzimidoyl chloride (43a)**

It was synthesized from compound **C5a** according to the synthesis method 3. Yield 97.0%; mp 115.8-117.7°C. ^{1}H-NMR ($CDCl_3$): δ_H 2.31 (3H, s), 5.12 (2H, s), 7.10 (2H, d, *J* = 8.8 Hz), 7.16-7.26 (3H, m), 7.39 (1H, d, *J* = 7.2 Hz), 7.71 (2H, d, *J* = 8.8 Hz), 12.14 (1H, s). HRMS (m/z) $[M+H]^+$ calcd for $C_{15}H_{14}ClNO_2$: 276.0791, found: 276.0784.

2-Fluoro-*N*-hydroxy-4-((2-methylbenzyl)oxy)benzimidoyl chloride (43b)

It was synthesized from compound **C5b** according to the synthesis method 3. Yield 86.0%; mp 105.3-107.7°C. ^{1}H NMR (400 MHz, DMSO-*d$_6$*): δ_H 2.32 (3H, s), 5.16 (2H, s), 6.98 (1H, dd, *J* = 9.2, 2.2 Hz), 7.09 (1H, dd, *J* = 13.2, 2.4 Hz), 7.19-7.28 (3H, m), 7.41 (1H, d, *J* = 7.2 Hz), 7.57 (1H, t, *J* = 8.4 Hz), 12.42 (1H, s). HRMS (m/z) $[M+H]^+$ calcd for $C_{15}H_{14}ClNO_2F$: 294.0697, found: 294.0691.

3-Fluoro-*N*-hydroxy-4-((2-methylbenzyl)oxy)benzimidoyl chloride (43c):

It was synthesized from compound **C5c** according to the synthesis method 3. Yield 53.0%; mp 97.4-99.0°C. ^{1}H NMR (400 MHz, DMSO-*d$_6$*): δ_H 2.34 (3H, s), 5.23 (2H, s), 7.20-7.30 (3H, m), 7.39-7.43 (2H, m), 7.57-7.60 (2H, m), 12.36 (1H, s). HRMS (m/z) $[M+H]^+$ calcd for $C_{15}H_{14}ClNO_2F$: 294.0697, found: 294.0684.

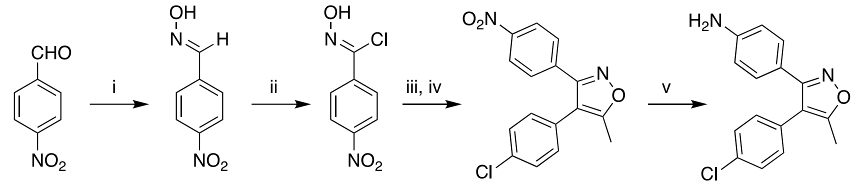

C6 **C7** **C8** **58**

Scheme S3. Reactions conditions and reagents: i) $NH_2OH \cdot HCl$, NaOH, H_2O, EtOH, rt; ii) NCS, DMF, rt; iii) TEA, diethyl ether, 0°C; iv) 4-chlorophenylacetone, NaH, THF, 0°C; v) $SnCl_2 \cdot 2H_2O$, EtOH, Δ.

4-Nitrobenzaldehyde oxime (C6) [CAS: 1129-37-9]

It was synthesized from 4-nitrobenzaldehyde according to the synthesis method 2. Yield 77.0%; mp 126.1-127.6°C (lit. mp 122.0-124.0°C).[8] HRMS (m/z) $[M+H]^+$ calcd for $C_7H_6N_2O_3$:166.0504, found:166.0515.

***N*-hydroxy-4-nitrobenzimidoyl chloride (C7) [CAS: 1011-84-3]**

It was synthesized from compound **C6** according to synthesis method 3. Yield: 79.0%; mp 114.9-117.1°C (lit. mp 107.0-110.0°C).[9] HRMS (m/z) $[M+H]^+$ calcd for $C_7H_6ClN_2O_3$: 201.0067, found: 201.0069.

4-(4-Chlorophenyl)-5-methyl-3-(4-nitrophenyl)isoxazole (C8)

It was synthesized from compound **C7** using 4-chlorophenylacetone according to synthesis method 4. The resulting crude product was purified by flash column chromatography (0% → 30% EtOAc in Hexane). Yield 28.0%; mp 118.9-120.7°C. ^1H NMR (400 MHz, DMSO-d_6): δ_H 2.47 (3H, s), 7.27 (2H, d, J = 8.4 Hz), 7.50 (2H, d, J = 8.4 Hz), 7.63 (2H, d, J = 8.8 Hz), 8.27 (2H, d, J = 8.8 Hz). HRMS (m/z) $[M+H]^+$ calcd for $C_{16}H_{12}N_2O_3Cl$: 315.0536, found: 315.0550.

4-(4-(4-Chlorophenyl)-5-methylisoxazol-3-yl)aniline (58)

It was synthesized from compound **C8** according to synthesis method 5. Yield 74.0%; mp 151.5-153.1°C. ^1H NMR (400 MHz, DMSO-d_6): δ_H 2.37 (3H, s), 5.44 (2H, s), 6.51 (2H, d, J = 8.4 Hz), 6.99 (2H, d, J = 8.4 Hz), 7.23 (2H, d, J = 8.0 Hz), 7.47 (2H, d, J = 8.0 Hz). HRMS (m/z) $[M+H]^+$ calcd for $C_{16}H_{14}N_2OCl$: 285.0795, found: 285.0793.

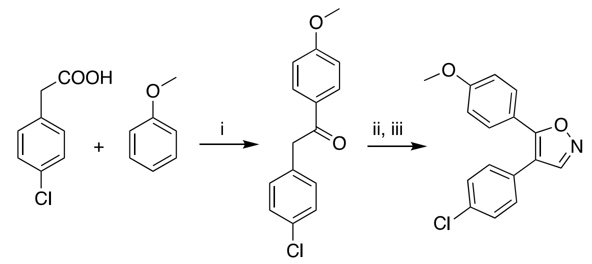

C9 **61**

Scheme S4. Reactions conditions and reagents: i) H_3PO_4, trifluoroacetic anhydride, 0°C; ii) DMFDMA, DMF; iii) NH_2OH•HCl, EtOH, Δ.

2-(4-Chlorophenyl)-1-(4-methoxyphenyl)ethanone (C9) [CAS: 52578-11-7]

H_3PO_4 (1.2 eq) was added in an ice bath to the reaction flask containing 4-chlorophenylacetic acid (1.0 eq) and anisole (1.2 eq), then trifluoroacetic acid (4.0 eq) was added and stirred for 5 min. At the end of the process, ice-water was added to the reaction mixture and the precipitated

white solid was filtered. Yield 81.0%; mp 131.7-133.0°C (lit. mp 137.5°C).[10] HRMS (m/z) $[M+H]^+$ calcd for $C_{15}H_{14}ClO_2$: 261.0682, found: 261.0680.

4-(4-Chlorophenyl)-5-(4-methoxyphenyl)isoxazole (61)

Compound **C9** (1.0 eq) and DMFDMA (5.0 eq) were dissolved in DMF and stirred at rt overnight. The reaction mixture was evaporated, the obtained 2-(4-chlorophenyl)-3-(dimethylamino)-1-(4-methoxyphenyl)prop-2-en-1-one and $NH_2OH.HCl$ (1.25 eq) were dissolved in absolute ethanol. It was heated under reflux for 1.5h. At the end of the period, ethanol was evaporated and then extracted with dichloromethane, the organic layer was dried anhydrous Na_2SO_4 and evaporated. Yield 62.0%; mp 96.2-97.5°C. ^{1}H-NMR ($CDCl_3$): δ_H 3.79 (3H, s), 7.03 (2H, d, J = 8.8 Hz), 7.43 (2H, d, J = 8.8 Hz), 7.47-7.51 (4H, m), 8.85 (1H, s). HRMS (m/z) $[M+H]^+$ calcd for $C_{16}H_{13}ClNO_2$: 286.0628, found: 286.0635.

4-(4-(4-Chlorophenyl)isoxazol-5-yl)phenol (62)

It was synthesized from compound **61** according to the synthesis method 6a. Yield 97.0%; mp 185.9-187.1°C. ^{1}H-NMR ($CDCl_3$): δ_H 6.83 (2H, d, J = 8.8 Hz), 7.36-7.48 (6H, m), 8.82 (1H, s), 10.07 (1H, s). HRMS (m/z) $[M+H]^+$ calcd for $C_{15}H_{11}ClNO_2$: 272.0482, found: 272.0478.

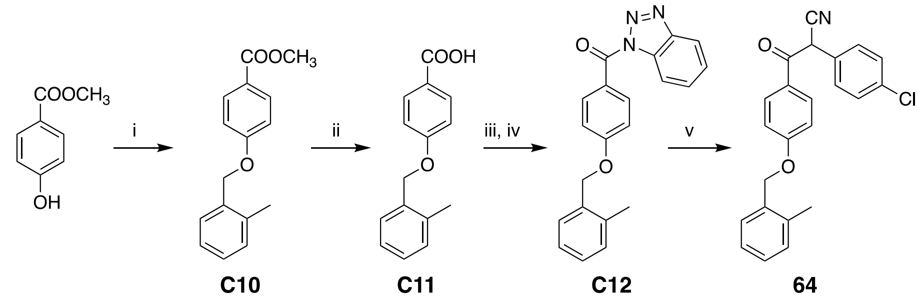

Scheme S5. Reactions conditions and reagents: i) 2-methylbenzyl bromide, K_2CO_3, MeCN, MWI, 120°C, 20 min; ii) $LiOH•H_2O$, $MeOH:H_2O$, Δ; iii) oxalyl chloride, DMF (cat.); iv) Benzotriazole, DCM, rt; v) (4-chlorophenyl)acetonitrile, LHMDS, THF, -70°C.

Methyl 4-((2-methylbenzyl)oxy)benzoate (C10) [CAS: 667433-10-5]

It was synthesized from methyl 4-hydroxybenzoate using 2-methylbenzyl bromide according to the synthesis method 1b. The resulting crude product was purified by flash column chromatography (0% → 30% EtOAc in Hexane). Yield 69.0%; mp 52.4-53.6°C. ^{1}H-NMR ($CDCl_3$): δ_H 3.31 (3H, s), 3.80 (3H, s), 5.16 (2H, s), 7.13 (2H, d, *J*=9.0 Hz), 7.17-7.27 (3H, m), 7.39 (1H, d, *J*=8.0 Hz), 7.91 (2H, d, *J*=9.0 Hz). HRMS (m/z) $[M+H]^+$ calcd for $C_{16}H_{17}O_3$: 257.1177, found: 257.1178.

4-((2-Methylbenzyl)oxy)benzoic acid (C11) [CAS: 149289-01-0]

Compound **C10** (1.0 eq) and LiOH.H_2O (2.5 eq) were dissolved in methanol-water and heated under reflux for 2h, then the reaction mixture was cooled and acidified with concentrated HCl, the precipitated solid was filtered under vacuum. Yield 96.0%; mp 171.2-173.2°C (lit. mp 169.0°C).[11] HRMS (m/z) $[M+H]^+$ calcd for $C_{15}H_{15}O_3$: 243.1021, found: 243.1017.

(1-*H*-Benzo[*d*][1,2,3]triazol-1-yl)(4-((2-methylbenzyl)oxy)phenyl)methanone (C12)

Compound **C11** (1.0 eq) was dissolved in dichloromethane, oxalyl chloride (2.0 eq) and a catalytic amount of DMF were added under a nitrogen atmosphere. It was stirred at rt for 1h, after the solvent was evaporated under vacuum. Then benzotriazole (3.0 eq) and dichloromethane were added and stirred at rt overnight. The reaction mixture was poured into K_2CO_3 solution, and the precipitate was filtered under vacuum. Yield 96.0%; mp 131.4-132.6°C. ^{1}H-NMR ($CDCl_3$): δ_H 2.34 (3H, s), 5.25 (2H, s), 7.19-7.29 (5H, m), 7.44 (1H, d, *J* = 6.8 Hz), 7.63 (1H, t, *J* = 7.6 Hz), 7.80 (1H, t, *J* = 7.6 Hz), 8.16 (2H, d, *J* = 9.2 Hz), 8.28 (1H, dd, *J* = 7.6, 6.0 Hz). HRMS (m/z) $[M+H]^+$ calcd for $C_{21}H_{18}N_3O_2$: 344.1393, found: 344.1399.

2-(4-Chlorophenyl)-3-(4-((2-methylbenzyl)oxy)phenyl)-3-oxopropanenitrile (64)

4-Chlorophenylacetonitrile (1.0 eq) was dissolved in dry THF under nitrogen atmosphere in acetone/dry ice bath (-78°C), then lithium bis(trimethylsilyl)amide (2.5 eq) was added and stirred for 30 min. Compound **C12** was dissolved in dry THF, and then the solution was added slowly with a syringe in the reaction mixture. After addition, the reaction was warmed to rt, and stirred overnight. The reaction was poured into 2M HCl solution and then extracted with dichloromethane, the organic layer dried and evaporated. The resulting crude product was

purified by flash column chromatography (0% → 50% EtOAc in Hexane). Yield 55.0%; mp 117.2-119.2°C. HRMS (m/z) $[M-H]^-$ calcd for $C_{23}H_{17}ClNO_2$: 374.0948, found: 374.0916.

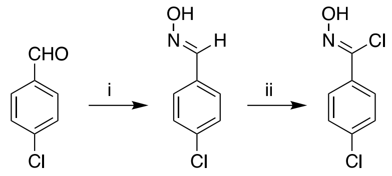

C13 66

Scheme S6. Reactions conditions and reagents: i) NH_2OH•HCl, NaOH, H_2O, EtOH, rt; ii) NCS, DMF, rt.

4-Chlorobenzaldehyde oxime (C13) [CAS: 3848-36-0]

It was synthesized from 4-chlorobenzaldehyde according to the synthesis method 2. Yield 91.0%; mp 106.1-108.3°C (lit. mp 110.0°C).[12] HRMS (m/z) $[M+H]^+$ calcd for C_7H_6ClNO: 156.0216, found: 156.0210.

***N*-hydroxy-4-chlorobenzimidoyl chloride (66) [CAS: 28123-63-9]**

It was synthesized from compound **C13** according to synthesis method 3. Yield 93.0%, mp 73.0-74.1°C (lit. mp 76.0-77.0.0°C).[13] HRMS (m/z) $[M+H]^+$ calcd for $C_7H_6Cl_2NO$: 188.9748, found: 188.9758.

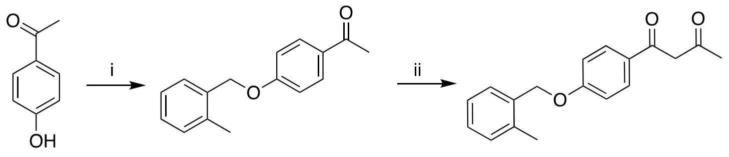

C14 68

Scheme S7. Reactions conditions and reagents: i) 2-methylbenzyl bromide, K_2CO_3, MeCN, Δ; ii) NaH, EtOAc, rt.

1-(4-((2-Methylbenzyl)oxy)phenyl)ethanone (C14) [CAS: 72293-94-8]

It was synthesized from 1-(4-hydroxyphenyl)ethanone using 2-methylbenzyl bromide according to the synthesis method 1a. It was crystallized with hexane. Yield 95.8%; mp 95.8-98.2°C (lit. mp 97.0-99.0.0°C).[14] HRMS (m/z) $[M+H]^+$ calcd for $C_{16}H_{17}O_2$: 241.1223, found: 241.1229.

1-(4-((2-Methylbenzyl)oxy)phenyl)butan-1,3-dione (68)

To a solution of compound **C14** (1.0 eq) in ethyl acetate, NaH (4.0 eq) was added and stirred at rt overnight. The reaction mixture was evaporated, and the crude product was treated with ether and filtered, then poured into water and acidified with concentrated HCl, the precipitate was filtered in vacuo and dried. Yield 72.0%; mp 111.2-113.1 °C. ^{1}H-NMR ($CDCl_3$): δ_H 2.17 (3H, s), 2.38 (3H, s), 5.10 (2H, s), 6.12 (1H, s), 7.03 (2H, d, J = 9.0 Hz), 7.21-7.29 (3H, m), 7.39 (1H, d, J = 6.8 Hz), 7.88 (2H, d, J = 9.0 Hz). HRMS (m/z) $[M+H]^+$ calcd for $C_{18}H_{19}O_3$: 283.1334, found: 283.1341.

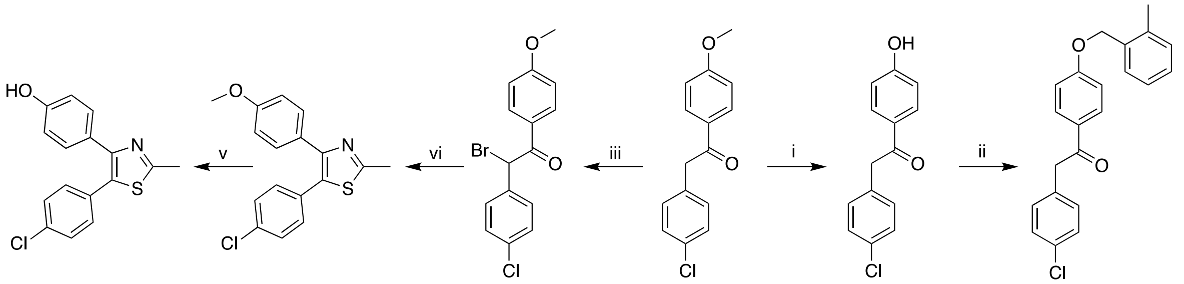

74 **73** **C16** **C9** **C15** **70**

Scheme S8. Reactions conditions and reagents: i) HBr, AcOH, Δ; ii) 2-methylbenzyl bromide, K_2CO_3, MeCN, Δ; iii) Br_2, AcOH, rt; iv) thioacetamide, EtOH, Δ; v) BBr_3, DCM, 0°C.

2-(4-Chlorophenyl)-1-(4-hydroxyphenyl)ethanone (C15) [CAS: 54419-40-8]

It was synthesized according to the synthesis method 6b. Yield 72.0%; mp 196.5-198.2°C (lit. mp. 198.0°C).[15] HRMS (m/z) $[M+H]^+$ calcd for $C_{14}H_{12}ClNO_2$: 247.0526, found: 247.0524.

2-(4-Chlorophenyl)-1-(4-((2-methylbenzyl)oxy)phenyl)ethanone (70)

It was synthesized from compound **C15** using 2-methylbenzyl bromide according to synthesis method 1b. Yield 73.0%; mp 139.0-141.0°C. ^{1}H-NMR ($CDCl_3$): δ_H 2.31 (3H, s), 4.33 (2H, s), 5.18 (2H, s), 7.13-7.27 (7H, m), 7.36-7.40 (3H, m), 8.01 (2H, d, J = 8.4 Hz). HRMS (m/z) $[M+H]^+$ calcd for $C_{22}H_{20}ClO_2$: 351.1161, found: 351.1152.

2-Bromo-2-(4-chlorophenyl)-1-(4-methoxyphenyl)ethanone (C16) [CAS: 42445-05-6]

Compound **C9** (1.0 eq) was taken up in glacial acetic acid and bromine (1.2 eq) was added dropwise. The reaction was stirred at rt overnight. The reaction mixture was poured into sodium metabisulfite solution and extracted with dichloromethane. The organic layer was dried with anhydrous Na_2SO_4, dichloromethane was evaporated under vacuum. The resulting crude product was purified by flash column chromatography (0% → 15% EtOAc in Hexane). Yield 65.0%; mp 106.8-108.0°C (lit. mp. 106.5°C).[16] HRMS (m/z) $[M+H]^+$ calcd for $C_{15}H_{13}BrClO_2$: 338.9787, found: 338.9789.

5-(4-Chlorophenyl)-4-(4-methoxyphenyl)-2-methylthiazole (73)

Compound **C16** (1 eq) and thioacetamide (1.2 eq) were dissolved in ethanol and stirred under reflux for 5h. At the end of the time, the reaction mixture was evaporated, and the residue was extracted with ethyl acetate and dried with anhydrous Na_2SO_4. Ethyl acetate was evaporated under vacuum. The resulting crude product was purified by flash column chromatography (0% → 15% EtOAc in Hexane)[17]. Yield 66.0%; mp 111.7-113.4°C. ^{1}H-NMR ($CDCl_3$): δ_H 2.67 (3H, s), 3.73 (3H, s), 6.44 (2H, d, J = 9.2 Hz), 7.29 (2H, d, J = 8.4 Hz), 7.32 (2H, d, J = 9.2 Hz), 7.42 (2H, d, J = 8.4 Hz). HRMS (m/z) $[M+H]^+$ calcd for $C_{17}H_{15}ClNOS$: 316.0563, found: 316.0555.

4-(5-(4-Chlorophenyl)-2-methylthiazol-4-yl)phenol (74)

It was synthesized from compound **73** according to the synthesis method 6a. The crude product was purified by automated flash chromatography using RediSep Silica columns (24 g), eluting with hexane:ethyl acetate (60:40) as mobile phase. Yield 68.0%; mp 203.6-204.8°C. ^{1}H-NMR ($CDCl_3$): δ_H 2.66 (3H, s), 6.68 (2H, d, J = 8.8 Hz), 7.21 (2H, d, J = 8.8 Hz), 7.29 (2H, d, J = 8.8 Hz), 7.41 (2H, d, J = 8.8 Hz), 9.58, (1H, s). HRMS (m/z) $[M+H]^+$ calcd for $C_{16}H_{13}ClNOS$: 302.0406, found: 302.0396.

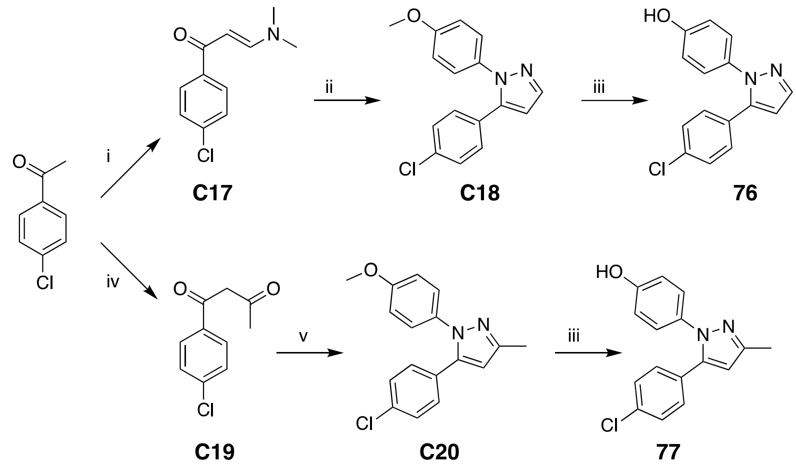

Scheme S9. Reactions conditions and reagents: i) DMFDMA, toluene, 130°C; ii) 4-methoxyphenylhydrazine•HCl, EtOH, Δ; iii) BBr_3, DCM, 0°C; iv) NaH, EtOAc, rt; v) 4-methoxyphenylhydrazine•HCl, TEA, MeOH, Δ.

1-(4-Chlorophenyl)-3-(dimethylamino)prop-2-en-1-one (C17) [CAS: 28587-05-5]

It was synthesized from 4-chloroacetophenone according to the synthesis method 7. Yield 82.0%; mp 81.8-83.8°C (lit. mp. 79.0-81.0°C).[18] HRMS (m/z) $[M+H]^+$ calcd for $C_{11}H_{13}ClNO$: 210.0686, found: 210.0678.

5-(4-Chlorophenyl)-1-(4-methoxyphenyl)-1H-pyrazole (C18)

It was synthesized from compound **C17** using 4-chlorophenylhydrazine.HCl according to the synthesis method 8a. The resulting crude product was purified by flash column chromatography (0% → 20% EtOAc in Hexane). Yield 58.0%; mp 111.2-112.2°C. ^{1}H-NMR ($CDCl_3$): δ_H 3.76 (3H, s), 6.64 (1H, d, J = 2.0 Hz), 6.95 (2H, d, J = 9.2 Hz), 7.17 (2H, d, J = 9.2 Hz), 7.21 (2H, d, J = 8.8 Hz), 7.40 (2H, d, J = 8.8 Hz), 7.70 (1H, d, J = 2.0 Hz). HRMS (m/z) $[M+H]^+$ calcd for $C_{16}H_{14}ClN_2O$: 285.0795, found: 285.0789.

4-(5-(4-Chlorophenyl)-1H-pyrazol-1-yl)phenol (76)

It was synthesized from compound **C18** according to the synthesis method 6a. Yield 38.0%; mp 185.9-187.1°C. ^{1}H-NMR (CDCl$_3$): δ_H 6.63 (1H, m), 6.76 (2H, d, J = 7.6 Hz), 7.04 (2H, d, J = 7.6 Hz), 7.20 (2H, d, J = 7.6 Hz), 7.39 (2H, d, J = 7.6 Hz), 7.66 (1H, s), 9.75 (1H, s). HRMS (m/z) [M+H]$^+$ calcd for $C_{15}H_{11}ClN_2O$: 271.0638, found: 271.0634.

1-(4-Chlorophenyl)butane-1,3-dione (C19) [CAS: 6302-55-2]

It was synthesized from 4-chloroacetophenone according to the compound **68**. Yield 37.0%; mp 68.1-69.1°C (lit. mp. 73.0-74.0°C).[19] HRMS (m/z) [M+H]$^+$ calcd for $C_{10}H_9ClO_2$: 197.0369, found: 197.0372.

5-(4-Chlorophenyl)-1-(4-methoxyphenyl)-3-methyl-1H-pyrazole (C20)

It was synthesized from compound **C19** using 4-methoxyphenylhydrazine.HCl according to the synthesis method 8c. Yield 78.0%; mp 113.2-114.8 °C. ^{1}H-NMR (CDCl$_3$): δ_H 2.23 (3H, s), 3.74 (3H, s), 6.43 (1H, s), 6.92 (2H, d, J = 9.2 Hz), 7.13 (2H, d, J = 9.2 Hz), 7.18 (2H, d, J = 8.6 Hz), 7.38 (2H, d, J = 8.6 Hz). HRMS (m/z) [M+H]$^+$ calcd for $C_{17}H_{16}ClN_2O$: 299.0951, found: 299.0945.

4-(5-(4-Chlorophenyl)-3-methyl-1H-pyrazol-1-yl)phenol (78) [CAS: 1397184-54-1]

It was synthesized from compound **C20** according to the synthesis method 6a. Yield 65.0%; mp 205.6-207.6°C. ^{1}H-NMR (CDCl$_3$): δ_H 2.22 (3H, s), 6.41 (1H, s), 6.73 (2H, d, J=8.4 Hz), 7.00 (2H, d, J=9.0 Hz), 7.17 (2H, d, J=8.4 Hz), 7.37 (2H, d, J=9.0 Hz), 9.70 (1H, s). HRMS (m/z) [M+H]$^+$ calcd for $C_{16}H_{14}ClN_2O$: 285.0795, found: 285.0787.

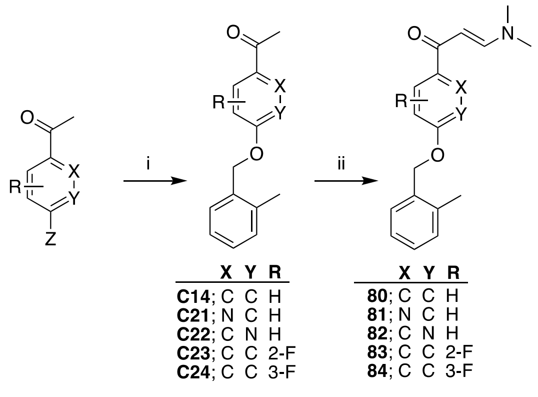

Scheme S10. Reactions conditions and reagents: I) 2-methylbenzyl bromide, K_2CO_3, MeCN, 120°C (2-methylbenzyl alcohol, NaH, THF for 114); ii) DMFDMA, toluene, 130°C.

1-(5-((2-Methylbenzyl)oxy20thenonen-2-yl20thenonee (C21)

It was synthesized from 1-(5-hydroxypyridin-2-yl20thenonee using 2-methylbenzyl bromide according to the synthesis method 1b. It was crystallized with hexane. Yield 82.0%; mp 49.5-51.3°C. ^{1}H-NMR ($CDCl_3$): δ_H 2.32 (3H, s), 2.56 (3H, s), 5.26 (2H, s), 7.18-7.28 (3H, m), 7.42 (1H, d, *J* = 7.6 Hz), 7.63 (1H, dd, *J* = 8.8, 2.8 Hz), 7.96 (1H, d, *J* = 8.8 Hz), 8.46 (1H, d, *J* = 2.8 Hz). HRMS (m/z) $[M+H]^+$ calcd for $C_{15}H_{16}NO_2$: 242.1181, found: 242.1173.

1-(6-((2-Methylbenzyl)oxy20thenonen-3-yl20thenonee (C22)

2-Methylbenzyl alcohol and NaH were dissolved in dry THF under nitrogen atmosphere and stirred in an ice bath for 1h. Then, a solution of 1-(6-chloro-3-pyridinyl)-1-ethanone in THF was added to the reaction mixture and stirred at rt. overnight. THF was evaporated and the reaction mixture was taken up in aqueous ammonium chloride solution and extracted with dichloromethane and dried with anhydrous Na_2SO_4, then organic layer was evaporated under vacuum. The resulting crude product was purified by flash column chromatography (0% → 30% EtOAc in Hexane). Yield 18.0%; mp 56.4-58.0°C. ^{1}H-NMR ($CDCl_3$): δ_H 2.31 (3H, s), 2.54 (3H, s), 5.42 (2H, s), 6.96 (1H, d, *J* = 8.6 Hz), 7.15-7.25 (3H, m), 7.38 (1H, d, *J* = 7.2 Hz), 8.17 (1H, dd, *J* = 8.6, 2.6 Hz), 8.82 (1H, d, *J* = 2.6 Hz). HRMS (m/z) $[M+H]^+$ calcd for $C_{15}H_{16}NO_2$: 242.1181, found: 242.1183.

1-(2-Fluoro-4-((2-methylbenzyl)oxy)phenyl)ethanone (C23) [CAS: 1992054-68-8]

It was synthesized from 2-fluoro-4-hydroxyacetophenone using 2-methyl benzyl bromide according to the synthesis method 1b. It was crystallized with hexane. Yield 86.0%; mp 62.6-63.9°C. HRMS (m/z) $[M+H]^+$ calcd for $C_{16}H_{16}FO_2$: 259.1134, found: 259.1133.

1-(3-Fluoro-4-((2-methylbenzyl)oxy)phenyl)ethanone (C24) [CAS: 1714202-57-9]

It was synthesized from 3-fluoro-4-hydroxyacetophenone using 2-methyl benzyl bromide according to the synthesis method 1b. Yield 72.0%; mp 96.6-97.9°C. HRMS (m/z) $[M+H]^+$ calcd for $C_{16}H_{16}FO_2$: 259.1134, found: 259.1136.

3-(Dimethylamino)-1-(4-((2-methylbenzyl)oxy)phenyl)prop-2-en-1-one (80)

It was synthesized from compound **C14** according to synthesis method 7. Yield 95.0%; mp 139.8-141.8°C. ^{1}H-NMR ($CDCl_3$): δ_H 2.31 (3H, s), 2.88 (3H, bs), 3.10 (3H, bs), 5.12 (2H, s), 5.79 (1H, d, *J* = 12.0 Hz), 7.04 (2H, d, *J* = 8.8 Hz), 7.16-7.26 (3H, m), 7.40 (1H, d, *J* = 7.2 Hz), 7.64 (1H, d, *J* = 12.0 Hz), 7.87 (2H, d, *J* = 8.8 Hz). HRMS (m/z) $[M+H]^+$ calcd for $C_{19}H_{21}NO_2$: 296.1638, found: 296.1651.

3-(Dimethylamino)-1-(5-((2-methylbenzyl)oxy)pyridin-2-yl)prop-2-en-1-one (81)

It was synthesized from compound **C21** according to synthesis method 7. Yield 91.0%; mp 153.6-155.1°C. ^{1}H-NMR ($CDCl_3$): δ_H 2.32 (3H, s), 2.87 (3H, bs), 3.13 (3H, bs), 5.21 (2H, s), 6.32 (1H, d, *J* = 12.8 Hz), 7.17-7.26 (3H, m), 7.41 (1H, d, *J* = 7.6 Hz), 7.56 (1H, dd, *J* = 8.4, 3.2 Hz), 7.72 (1H, d, *J* = 12.8 Hz), 7.96 (1H, d, *J* = 8.4 Hz), 8.36 (1H, d, *J* = 3.2 Hz). HRMS (m/z) $[M+H]^+$ calcd for $C_{18}H_{21}N_2O_2$: 297.1603, found: 297.1591.

3-(Dimethylamino)-1-(6-((2-methylbenzyl)oxy)pyridin-3-yl)prop-2-en-1-one (82)

It was synthesized from compound **C22** according to synthesis method 7. Yield 71.0%; mp 109.3-111.4°C. ^{1}H-NMR ($CDCl_3$): δ_H 2.31 (3H, s), 2.89 (3H, bs), 3.12 (3H, bs), 5.38 (2H, s), 5.80 (1H, d, *J* = 12.2 Hz), 6.88 (1H, d, *J* = 8.6 Hz), 7.15-7.25 (3H, m), 7.38 (1H, d, *J* = 7.6 Hz), 7.69 (1H, d, *J* = 12.2 Hz), 8.16 (1H, dd, *J* = 8.6, 2.4 Hz), 8.74 (1H, d, *J* = 2.4 Hz). HRMS (m/z) $[M+H]^+$ calcd for $C_{18}H_{21}N_2O_2$: 297.1603, found: 297.1591.

3-(Dimethylamino)-1-(2-fluoro-4-((2-methylbenzyl)oxy)phenyl)prop-2-en-1-one (83)

Starting from the compound **C23**, it was synthesized according to the synthesis method 7. Yield 90.0%; mp 112.8-113.6°C. ^{1}H-NMR (DMSO-d_6): δ_H 2.32 (3H, s), 2.84 (3H, bs), 3.10 (3H, bs), 5.15 (2H, s), 5.50 (1H, d, *J* = 12.0 Hz), 6.89-6.96 (2H, m), 7.19-7.28 (3H, m), 7.41 (1H, d, *J* =

7.2 Hz), 7.62-7.66 (2H, m). HRMS (m/z) $[M+H]^+$ calcd for $C_{19}H_{21}FNO_2$: 314.1556, found: 314.1552.

3-(Dimethylamino)-1-(3-fluoro-4-((2-methylbenzyl)oxy)phenyl)prop-2-en-1-one (84)

Starting from the compound **C24**, it was synthesized according to the synthesis method 7. Yield 97.0%; mp 131.8-132.9°C. ^{1}H-NMR (DMSO-d_6): δ_H 2.34 (3H, s), 2.91 (3H, bs), 3.13 (3H, bs), 5.23 (2H, s), 5.83 (1H, d, J = 12.4 Hz), 7.19-7.29 (3H, m), 7.31 (1H, t, J = 8.6 Hz), 7.42 (1H, d, J = 7.2 Hz), 7.69 (1H, d, J = 12.4 Hz), 7.73-7.76 (2H, m). HRMS (m/z) $[M+H]^+$ calcd for $C_{19}H_{21}FNO_2$: 314.1556, found: 314.1563.

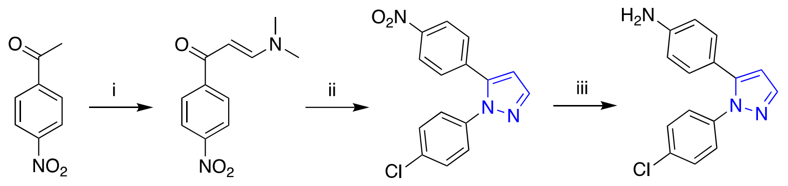

C25 **C26** **90**

Scheme S11. Reactions conditions and reagents: i) DMFDMA, toluene, 130°C; ii) 4-chlorophenylhydrazine, EtOH, Δ; iii) $SnCl_2•2H_2O$, EtOH, Δ.

3-(Dimethylamino)-1-(4-nitrophenyl)prop-2-en-1-one (C25) [CAS: 68760-11-2]

It was synthesized starting from 4-nitroacetophenone according to the synthesis method 7. The resulting crude product was purified by flash column chromatography (0% → 40% EtOAc in Hexane). Yield 76.0%; mp 146.3-147.3°C. HRMS (m/z) $[M+H]^+$ calcd for $C_{11}H_{13}N_2O_3$: 221.0926, found: 221.0919.

1-(4-Chlorophenyl)-5-(4-nitrophenyl)-1H-pyrazole (C26) [CAS: 1539280-41-5]

It was synthesized from compound **C25** using 4-chlorophenylhydrazine.HCl according to the synthesis method 8a. The resulting crude product was purified by flash column chromatography (0% → 40% EtOAc in Hexane). Yield 78.0%; mp 155.3-156.7°C (lit. mp. 156°C).[20] HRMS (m/z) $[M+H]^+$ calcd for $C_{15}H_{11}ClN_3O_2$: 300.0540, found: 300.0538.

4-(1-(4-Chlorophenyl)-1*H*-pyrazol-5-yl)aniline (90)

It was synthesized from compound **C26** according to synthesis method 5. The resulting crude product was purified by flash column chromatography (0% → 50% EtOAc in Hexane). Yield 83.0%; mp 129.3-131.0°C. ^{1}H NMR (400 MHz, DMSO-d_6): δ_H 5.35 (2H, s), 6.45 (1H, d, J = 1.8 Hz), 6.51 (2H, d, J = 8.4 Hz), 6.87 (2H, d, J = 8.8 Hz), 7.28 (2H, d, J = 8.8 Hz), 7.46 (2H, d, J = 8.4 Hz), 7.68 (1H, d, J = 1,8 Hz). HRMS (m/z) $[M+H]^+$ calcd for $C_{15}H_{13}ClN_3$: 270.0798, found: 270.0790.

Table S1. In vitro growth inhibitory values of **11** and **85** at 10 μM in NCI60 cancer cell panel

**Panel**	**Cell Line**	**% Growth Inhibition**	
		**Cmpd. 11**	**Cmpd. 85**
**Leukemia**	CCRF-CEM	61.75	57.64
	HL-60 (TB)	82.31	86.68
	K-562	85.59	86.33
	MOLT-4	67.19	73.70
	RPMI-8226	19.01	22.65
	SR	66.03	66.23
**Non-Small Cell Lung Cancer**	A549/ATCC	50.72	50.45
	EKVX	24.92	22.95
	HOP-62	48.38	50.69
	HOP-92	27.38	25.83
	NCI-H226	42.83	29.68
	NCI-H23	26.23	23.40
	NCI-H322M	2.44	3.12
	NCI-H460	45.31	41.69
	NCI-H522	74.52	70.91
**Colon Cancer**	COLO 205	29.02	28.76
	HCC-2998	13.42	10.87
	HCT-116	65.97	61.92
	HCT-15	62.45	60.08
	HT29	83.10	82.41
	KM12	45.72	49.31
	SW-620	68.31	70.35
**CNS Cancer**	SF-268	24.36	19.50
	SF-295	50.56	46.90
	SF-539	36.48	28.17
	SNB-19	44.49	37.98
	SNB-75	47.10	39.28

	U251	58.73	54.02
**Melanoma**	LOX-IMVI	32.05	35.73
	MALME-3M	40.19	32.80
	M14	67.78	61.01
	MDA-MB-435	100	99.19
	SK-MEL-2	62.88	60.82
	SK-MEL-28	29.55	30.64
	SK-MEL-5	26.57	33.38
	UACC-257	29.08	24.32
	UACC-62	51.47	39.32
**Ovarian Cancer**	IGROV1	47.48	42.48
	OVCAR-3	79.31	73.38
	OVCAR-4	17.33	14.28
	OVCAR-5	12.38	8.29
	OVCAR-8	24.25	24.75
	NCI/ADR-RES	60.79	62.00
	SK-OV-3	40.53	36.32
**Renal Cancer**	786-0	24.49	17.20
	A498	61.39	42.45
	ACHN	33.01	22.37
	CAKI-1	51.26	40.28
	RXF 393	29.83	39.71
	SN12C	31.68	32.4
	TK-10	15.70	16.23
	UO-31	25.57	25.63
**Prostate Cancer**	DU-145	22.64	11.90
**Breast Cancer**	MCF7	68.32	68.10
	MDA-MB-231	79.19	75.75
	HS 578T	40.58	39.09
	BT-549	33.82	25.02

	T-47D	38.92	34.63
	MDA-MB-468	63.91	58.87

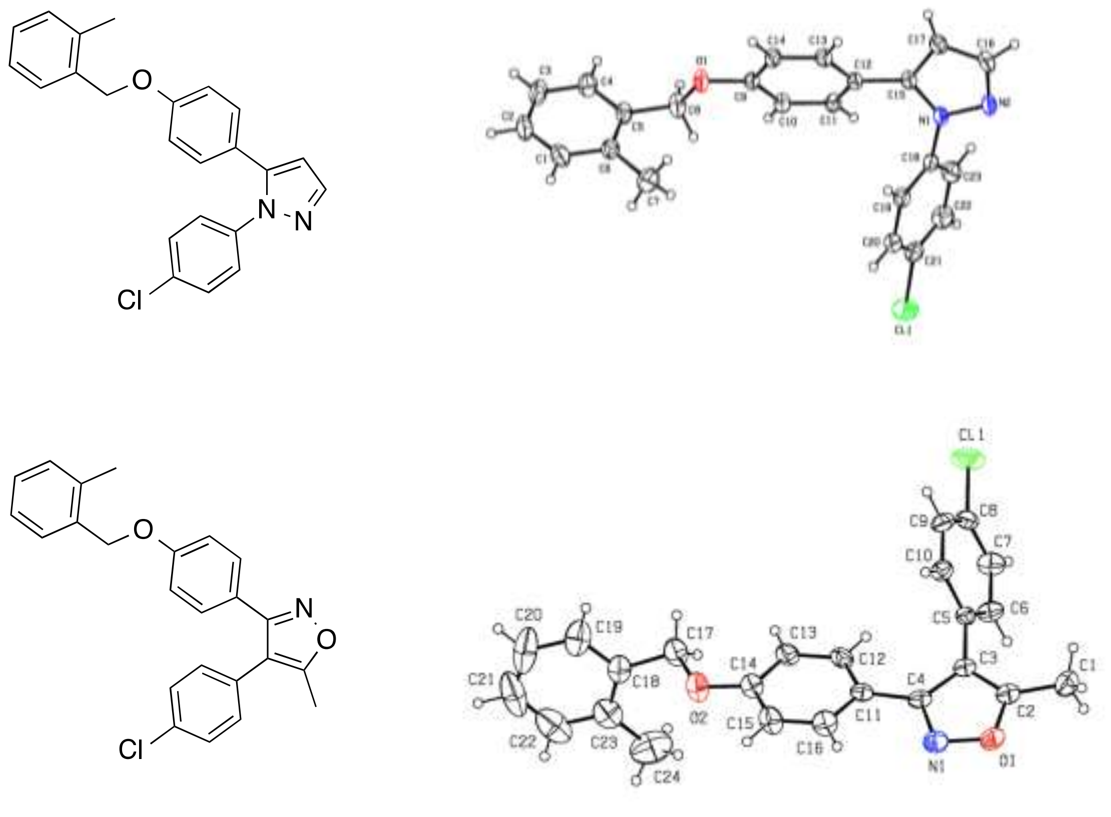

Figure S1. ORTEP drawings of the single crystal structures of compound **11** and **85**[21, 22]

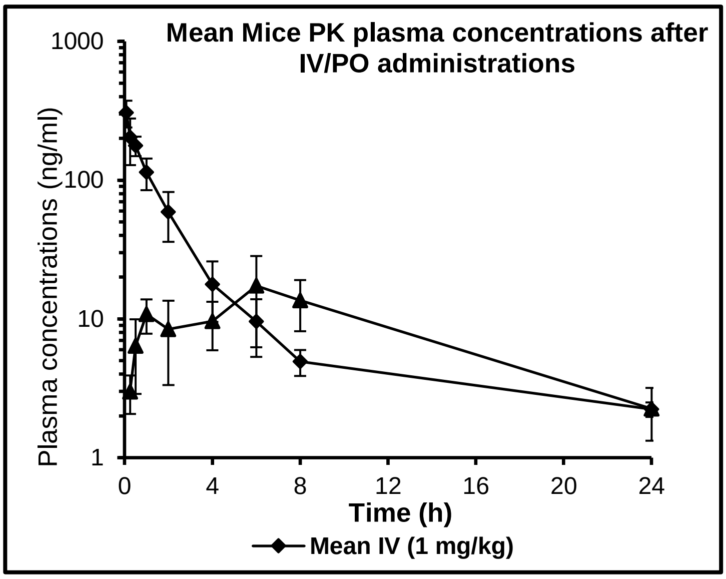

Figure S2. Plasma concentrations vs. time profile of **85** after single dose IV (1 mg/kg) and PO (10 mg/kg) administrations in male CD-1 mice. The PK study was done at Syngene International, Ltd., India.

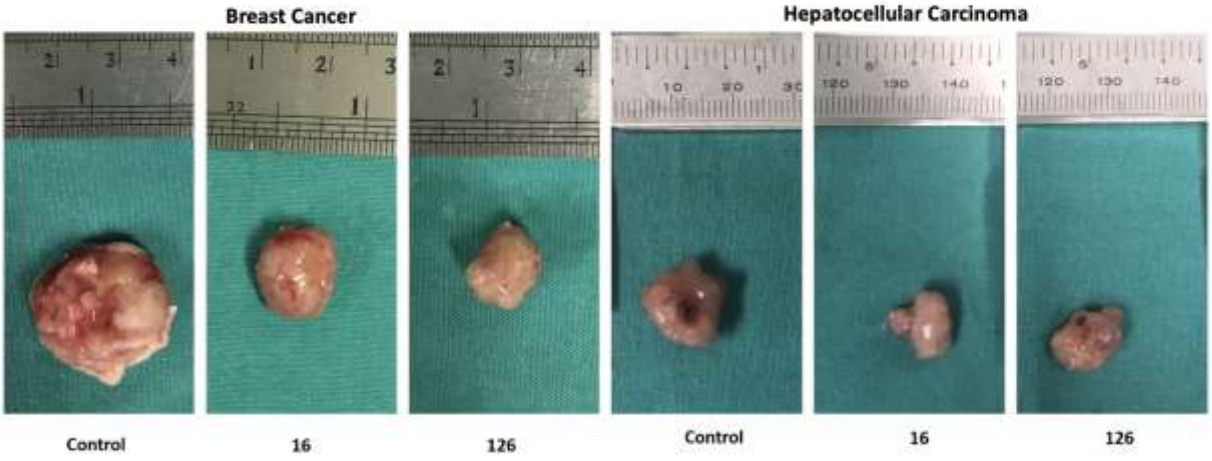

Figure S3. Representative images of dissected tumors from the xenograft models used in the study. Mahlavu and MDA-MB-231 xenograft nude mice were treated with 40 mg/kg **11**and **85** prepared in 0.5% hydroxypropyl methyl cellulose plus 1% Tween 80 or with vehicle only twice a week once the tumor size reached 100 mm^3. Each group contains 6 mice (n=6).

References

(1) Liu, H.; Wang, J.; Zhang, R.; Cairns, N.; Liu, J. Compounds, compositions and methods for reducing lipid levels. WO2009002873A1, **2009**.

(2) Pradhan, S.; Sharma, V.; Chatterjee, I. Nitrosoarene-Catalyzed HFIP-Assisted Transformation of Arylmethyl Halides to Aromatic Carbonyls under Aerobic Conditions. *Org Lett* **2021**, *23* (15), 6148-6152. DOI: 10.1021/acs.orglett.1c02272.

(3) Tyman, J. H. P.; Payne, P. B. The synthesis of phenolic propane-1, 2- and 1, 3-diols as intermediates in immobilised chelatants for the borate anion1. *J Chem Res* **2006**, *2006* (11), 691-695. DOI: 10.3184/030823406779173479.

(4) Tóth, M.; Kun, S.; Bokor, É.; Benltifa, M.; Tallec, G.; Vidal, S.; Docsa, T.; Gergely, P.; Somsák, L.; Praly, J.-P. Synthesis and structure–activity relationships of C-glycosylated oxadiazoles as inhibitors of glycogen phosphorylase. *Bioorg. Med. Chem.* **2009**, *17* (13), 4773-4785. DOI: 10.1016/j.bmc.2009.04.036.

(5) David, A.; Vannice, M. A. Control of catalytic debenzylation and dehalogenation reactions during liquid-phase reduction by H2. *J Catal* **2006**, *237* (2), 349-358. DOI: 10.1016/j.jcat.2005.11.017.

(6) Harada, H.; Fujii, A.; Kato, S. An Efficient and Practical Synthesis of N , N -Diethyl-7-indolyloxyacetamide via 7-Hydroxyindole. *Synth Commun* **2003**, *33* (3), 507-514. DOI: 10.1081/SCC-120015783.

(7) Jia, R.; Zhang, J.; Bertagnin, C.; Cherukupalli, S.; Ai, W.; Ding, X.; Li, Z.; Zhang, J.; Ju, H.; Ma, X.; et al. Discovery of highly potent and selective influenza virus neuraminidase inhibitors targeting 150-cavity. *Eur. J. Med. Chem.* **2021**, *212*, 113097. DOI: 10.1016/j.ejmech.2020.113097.

(8) Wan, M.; Xu, L.; Hua, L.; Li, A.; Li, S.; Lu, W.; Pang, Y.; Cao, C.; Liu, X.; Jiao, P. Synthesis and evaluation of novel isoxazolyl chalcones as potential anticancer agents. *Bioorg. Chem.* **2014**, *54*, 38-43. DOI: 10.1016/j.bioorg.2014.03.004.

(9) D'Ascenzio, M.; Carradori, S.; De Monte, C.; Secci, D.; Ceruso, M.; Supuran, C. T. Design, synthesis and evaluation of N-substituted saccharin derivatives as selective inhibitors of tumor-associated carbonic anhydrase XII. *Bioorg. Med. Chem.* **2014**, *22* (6), 1821-1831. DOI: 10.1016/j.bmc.2014.01.056.

(10) Khider, A. K.; Hawaiz, F. E.; Taha, S. O.; Ahmed, M. S. Synthesis and Antimicrobial Ctivity of 3-(4-(4-chlorobenzyloxy) phenyl)-1-phenyl-5-(2-chlorophenyl) Pyrazoline (3) on" E. coli" in Mice. *Asian J Med Sci* **2011**, *3* (4), 158-163.

(11) Jones, B. 426. The halogenation of phenolic ethers and anilides. Part V. Alkyl and ω-substituted-alkyl ethers. *Journal of the Chemical Society (Resumed)* **1935**, 1831-1835.

(12) Celik, H.; Ekmekci, G.; Ludvík, J.; Pícha, J.; Zuman, P. Electroreduction of aromatic oximes: Diprotonation, adsorption, imine formation, and substituent effects. *J. Phys. Chem. B* **2006**, *110* (13), 6785-6796.

(13) Ismail, T.; Shafi, S.; Singh, P. P.; Qazi, N. A.; Sawant, S. D.; Ali, I.; Khan, I. A.; Kumar, H. M. S.; Qazi, G. N.; Alam, M. S. Biologically active hydroxymoyl chlorides as antifungal agents. *Indian J Chem - B Org Med Chem* **2008**, *47* (5), 740-747.

(14) Wang, Z.-M.; Li, X.-M.; Xu, W.; Li, F.; Wang, J.; Kong, L.-Y.; Wang, X.-B. Acetophenone derivatives: novel and potent small molecule inhibitors of monoamine oxidase B. *MedChemComm* **2015**, *6* (12), 2146-2157.

(15) Arasavelli, A. M.; Raghava, G. S. V.; Vidavalur, S. Design and synthesis of 4, 5-diaryl/heteroarylthiophene-2-carboxylic acid derivatives and evaluation of their biological activities. *Heterocycl Comm* **2017**, *23* (1), 9-14.

(16) Jenkins, S. S. The Grignard Reaction in the Synthesis of Ketones. V. The Preparation of the Isomeric p-Chlorobenzanisoins. *J. Am. Chem. Soc.* **1934**, *56* (5), 1137-1138.
(17) Pal, P.; Gandhi, H. P.; Kanhed, A. M.; Patel, N. R.; Mankadia, N. N.; Baldha, S. N.; Barmade, M. A.; Murumkar, P. R.; Yadav, M. R. Vicinal diaryl azole-based urea derivatives as potential cholesterol lowering agents acting through inhibition of SOAT enzymes. *Eur J Med Chem* **2017**, *130*, 107-123. DOI: 10.1016/j.ejmech.2017.02.038.
(18) Lin, Y.-i.; Lang Jr, S. A. New synthesis of isoxazoles and isothiazoles. A convenient synthesis of thioenaminones from enaminones. *J. Org. Chem.* **1980**, *45* (24), 4857-4860.
(19) Rosenberg, D.; Strehlke, P. Chemotherapeutische Nitroheterocyclen, XXVI. 5- Nitro- 2- thiazolylpyrazole und 5- Nitro- 2- thiazolylisoxazole aus 1, 3- Dicarbonylverbindungen der Nitrothiazolreihe. *Justus Liebigs Ann. Chem.* **1976**, *1976* (1), 13-21.
(20) Ceccarelli, S. M. J., Ravi; Jakob-Roetne, Roland; Wichmann, Juergen. Benzisoxazole Modulators of Neurogenesis. WO2014016267A1, **2014**.
(21) Aydin, A.; Akkurt, M.; Turanlı, S.; Banoglu, E.; Ozcelık, N. Crystal Structure and Hirshfeld Surface Analysis of 1-(4-Chlorophenyl)-5-{4-[(2-methylphenyl) methoxy] phenyl}-1H-Pyrazole. *X-ray Struct Anal Online* **2021**, *37*, 77-79.
(22) Aydin, A.; Akkurt, M.; Turanli, S.; Lengerli, D.; Banoglu, E.; Ozcelik, N. D. Crystal structure and Hirshfeld surface analysis of 4-(4-chlorophenyl)-5-methyl-3-{4-[(2-methylphenyl) methoxy] phenyl}-1, 2-oxazole. *Acta Crystallogr E Crystallogr Commun* **2021**, *77* (4), 346-350.

NMR SPECTRA OF THE FINAL COMPOUNDS

Figure S4. 1H-NMR and 13C-NMR spectrum of Compound **9**

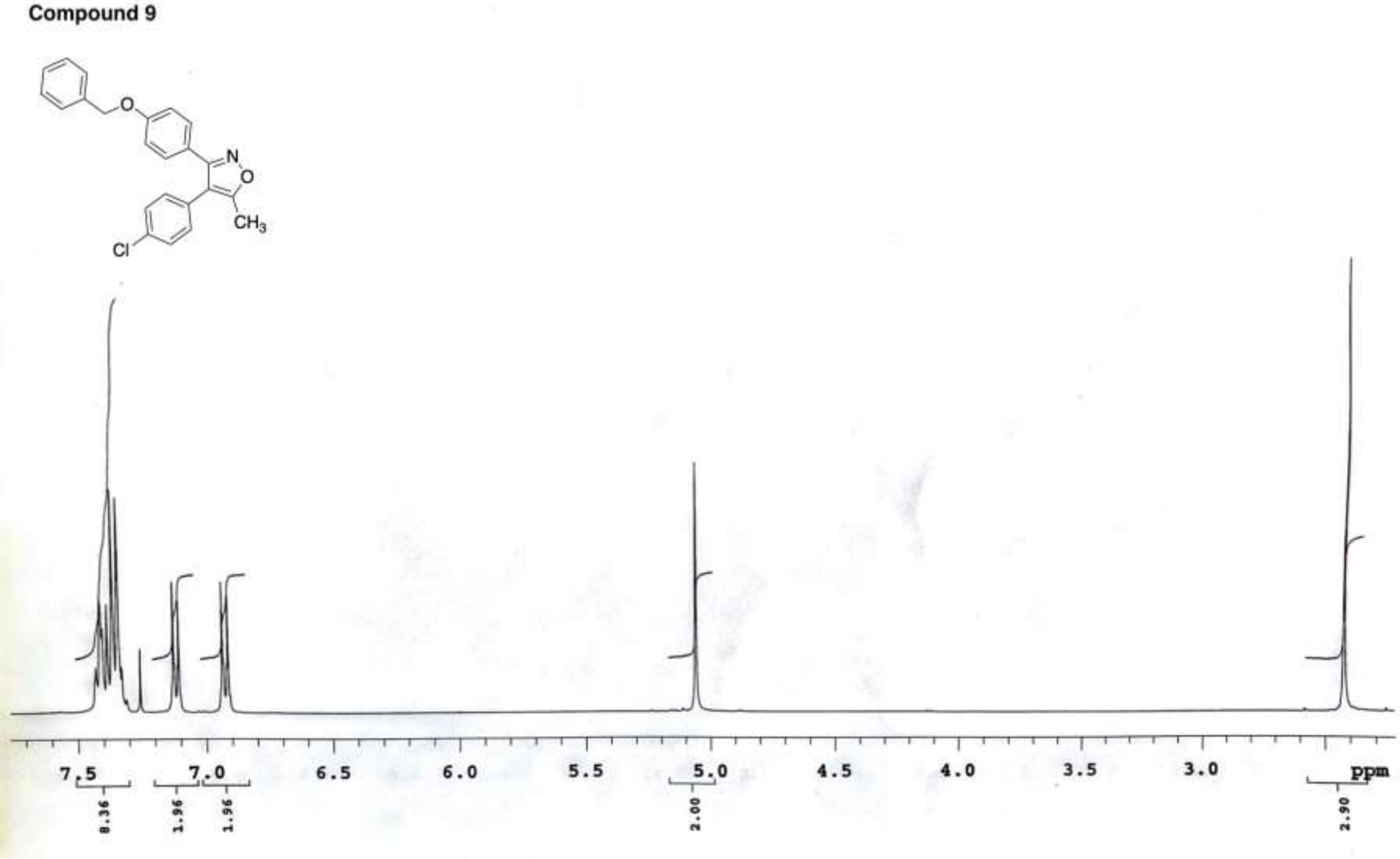

DNZ9

Sample Name:
DNZ9
Data Collected on:
mercury400-mercury400
Archive directory:
/home/vnmr1/vnmrsys/data
Sample directory:
DNZ9_20161001_01
FidFile: current

Pulse Sequence: CARBON (s2pul)
Solvent: cdcl3
Data collected on: Oct 1 2016

Temp. 25.0 C / 298.1 K
Operator: vnmr1

Relax. delay 1.000 sec
Pulse 45.0 degrees
Acq. time 1.550 sec
Width 21141.6 Hz
256 repetitions
OBSERVE C13, 100.6238513 MHz
DECOUPLE H1, 400.1760547 MHz
Power 38 dB
continuously on
WALTZ-16 modulated
DATA PROCESSING
Line broadening 0.5 Hz
FT size 65536
Total time 1 hr, 28 min

Compound 9

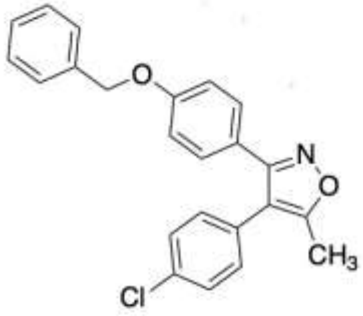

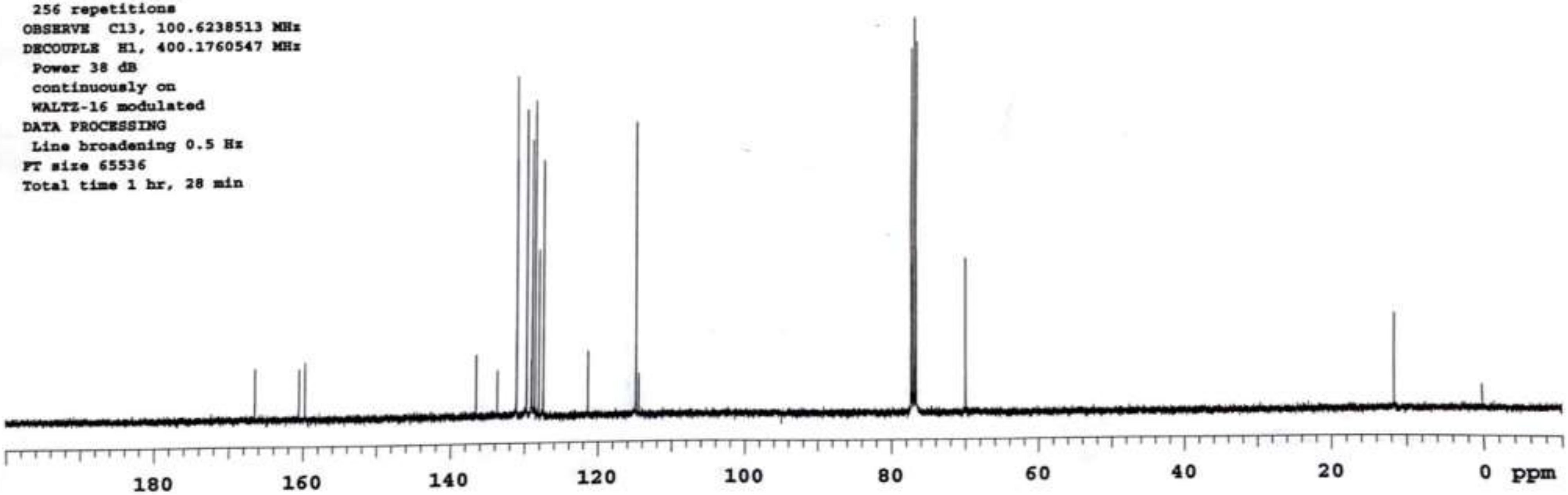

Figure S5. ^{1}H-NMR and ^{13}C-NMR spectrum of Compound **11**

Compound 11

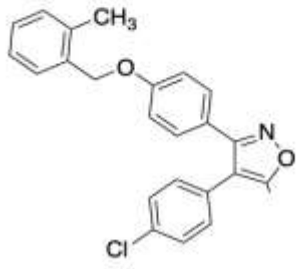

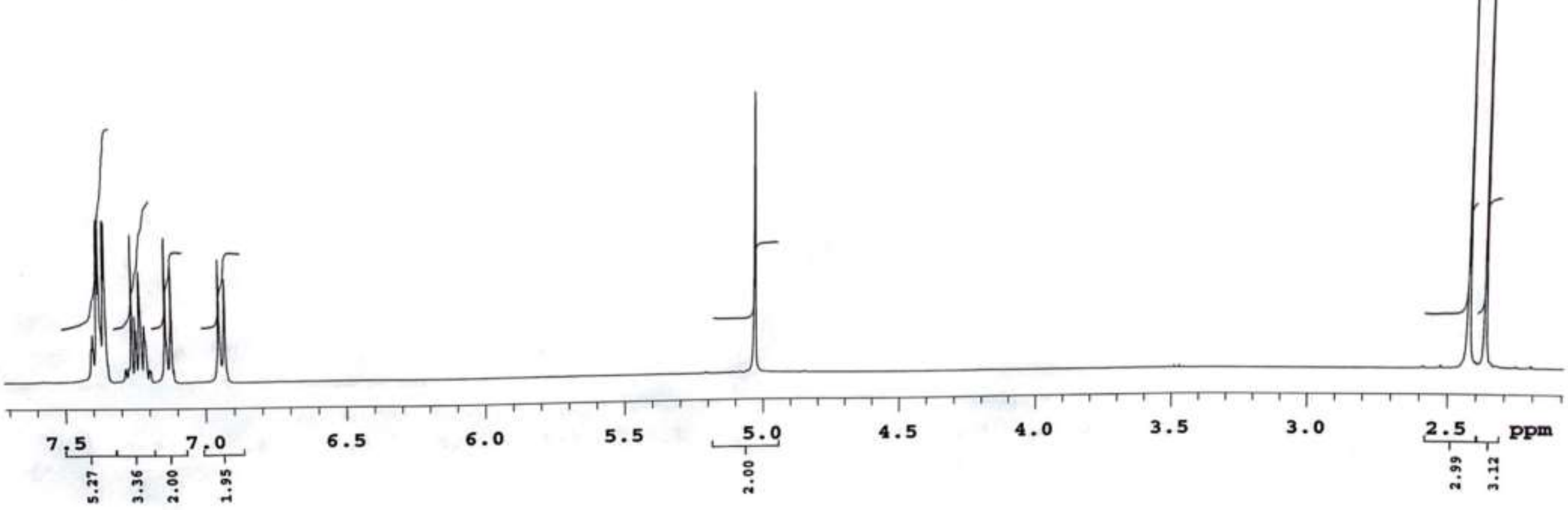

DNZ-78

Sample Name:
DNZ-78
Data Collected on:
mercury400-mercury400
Archive directory:
/home/vnmr1/vnmrsys/data
Sample directory:
DNZ-78_20161001_01
FidFile: current

Pulse Sequence: CARBON (s2pul)
Solvent: cdcl3
Data collected on: Oct 1 2016

Temp. 25.0 C / 298.1 K
Operator: vnmr1

Relax. delay 1.000 sec
Pulse 45.0 degrees
Acq. time 1.304 sec
Width 25125.6 Hz
128 repetitions
OBSERVE C13, 100.6238513 MHz
DECOUPLE H1, 400.1760547 MHz
Power 38 dB
continuously on
WALTZ-16 modulated
DATA PROCESSING
Line broadening 0.5 Hz
FT size 65536
Total time 1 hr

Compound 11

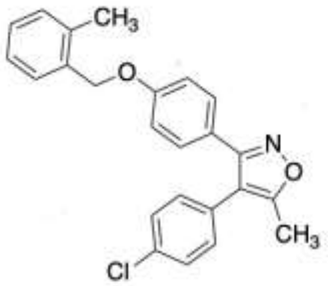

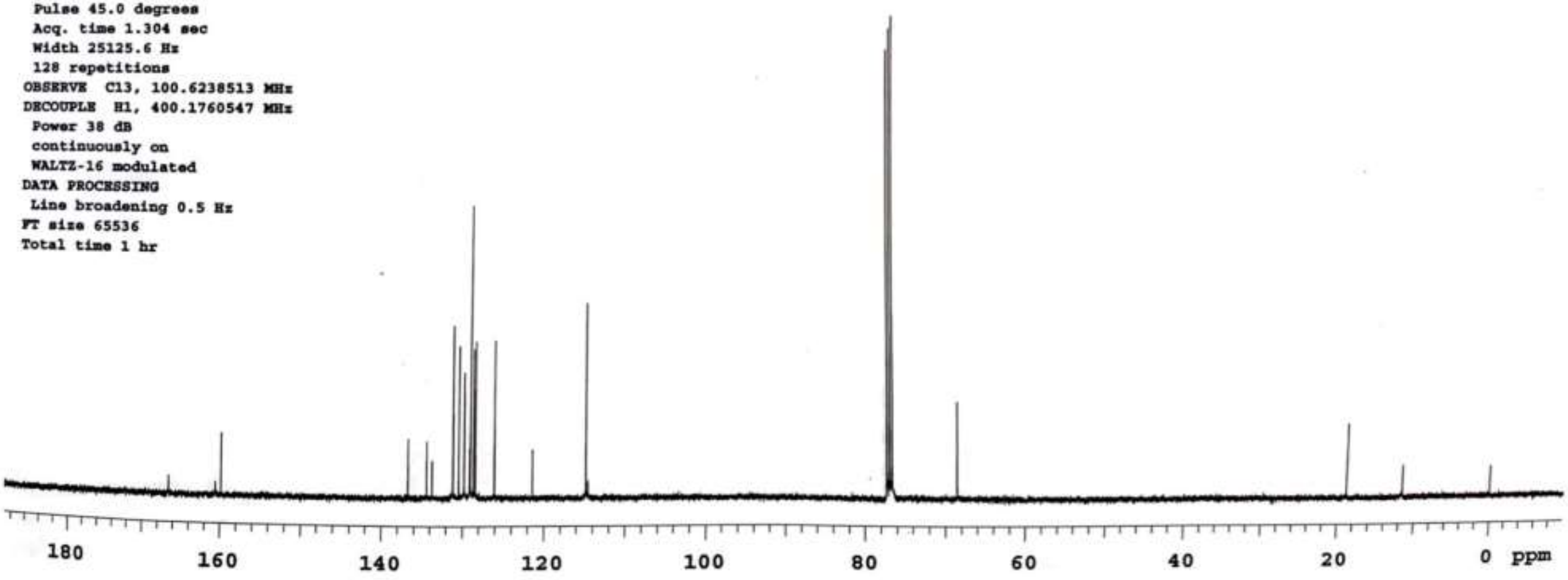

Figure S6. ^{1}H-NMR and ^{13}C-NMR spectrum of Compound **12**

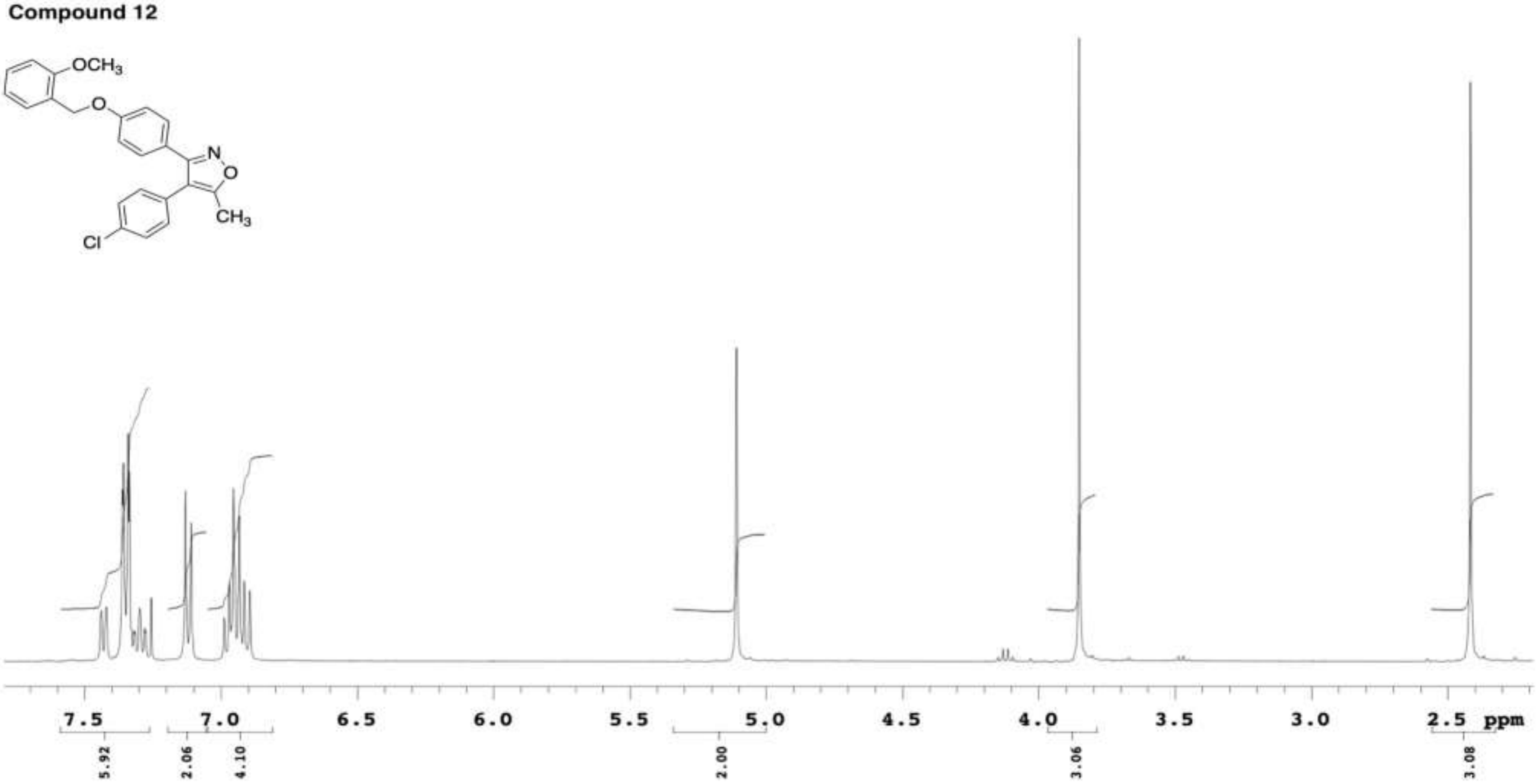

DNZ88

Sample Name:
DNZ88
Data Collected on:
mercury400-mercury400
Archive directory:
/home/vnmr1/vnmrsys/data
Sample directory:
DNZ88_20161124_01
FidFile: CARBON_01

Pulse Sequence: CARBON (s2pul)
Solvent: cdcl3
Data collected on: Nov 24 2016

Temp. 26.0 C / 299.1 K
Operator: vnmr1

Relax. delay 1.000 sec
Pulse 45.0 degrees
Acq. time 1.304 sec
Width 25125.6 Hz
2000 repetitions
OBSERVE C13, 100.6238513 MHz
DECOUPLE H1, 400.1760547 MHz
Power 38 dB
continuously on
WALTZ-16 modulated
DATA PROCESSING
Line broadening 0.5 Hz
FT size 65536
Total time 1 hr, 20 min

Compound 12

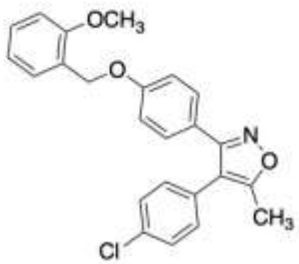

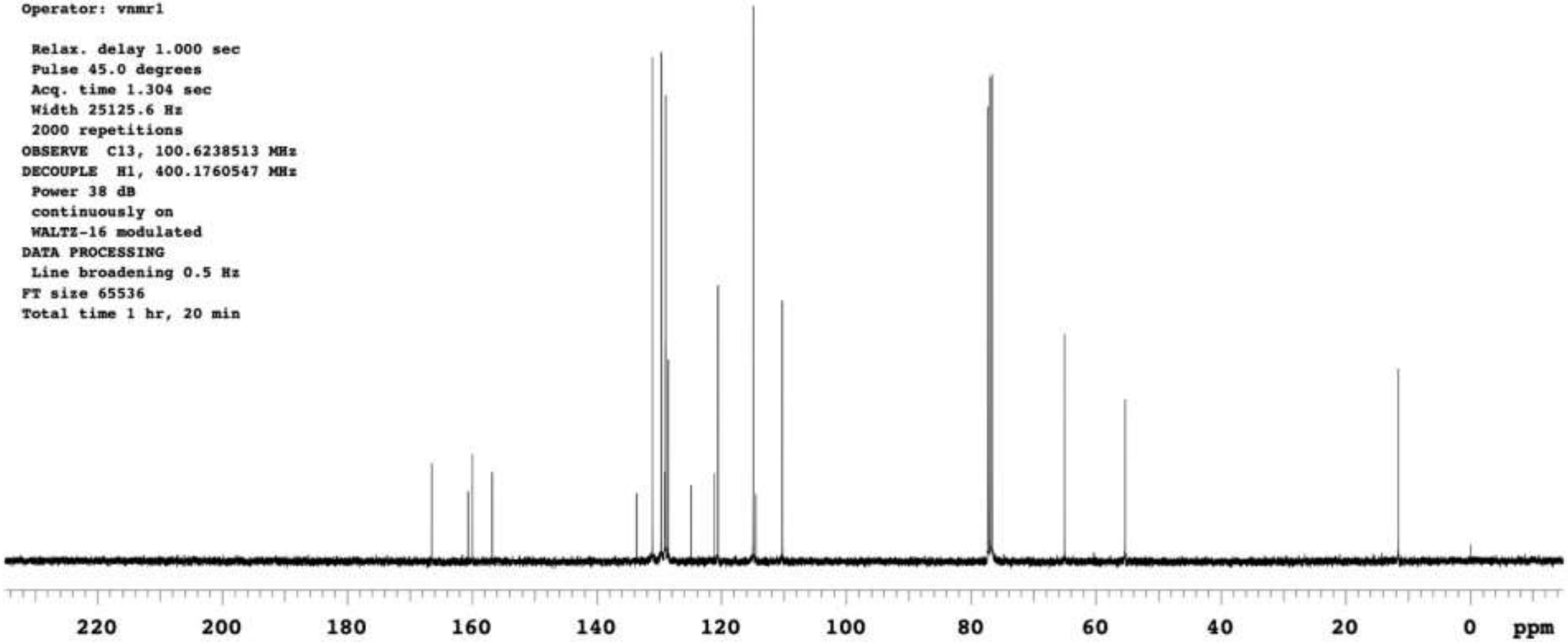

Figure S7. ^{1}H-NMR and ^{13}C-NMR spectrum of Compound **13**

Compound 13

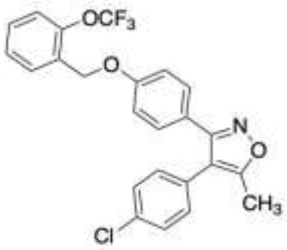

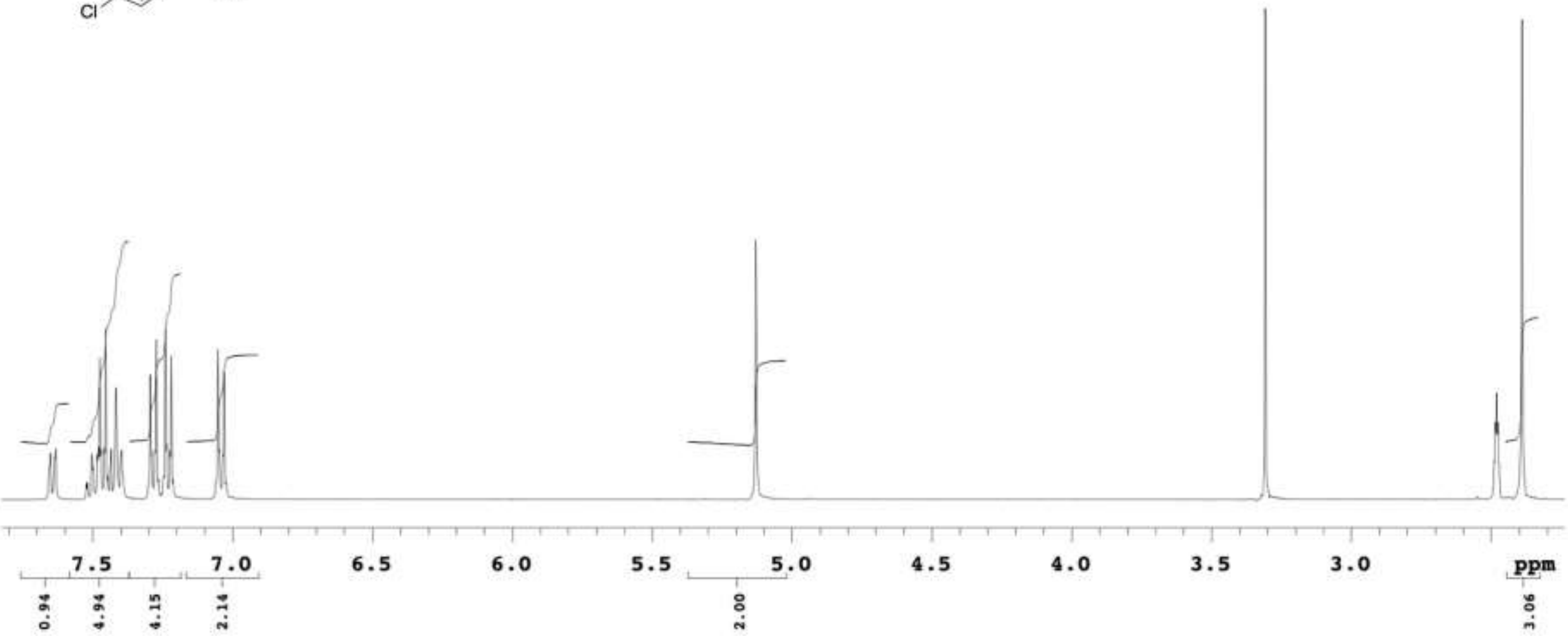

DNZ-140

Sample Name:
DNZ-140
Data Collected on:
mercury400-mercury400
Archive directory:
/home/vnmr1/vnmrsys/data
Sample directory:
DNZ-140_20170415_01
FidFile: current

Pulse Sequence: CARBON (s2pul)
Solvent: dmso
Data collected on: Apr 15 2017

Temp. 26.0 C / 299.1 K
Operator: vnmr1

Relax. delay 1.000 sec
Pulse 45.0 degrees
Acq. time 1.550 sec
Width 21141.6 Hz
64 repetitions
OBSERVE C13, 100.6243781 MHz
DECOUPLE H1, 400.1779555 MHz
Power 38 dB
continuously on
WALTZ-16 modulated
DATA PROCESSING
Line broadening 0.5 Hz
FT size 65536
Total time 1 hr, 28 min

Compound 13

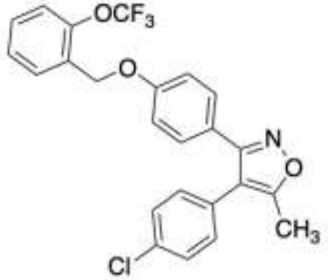

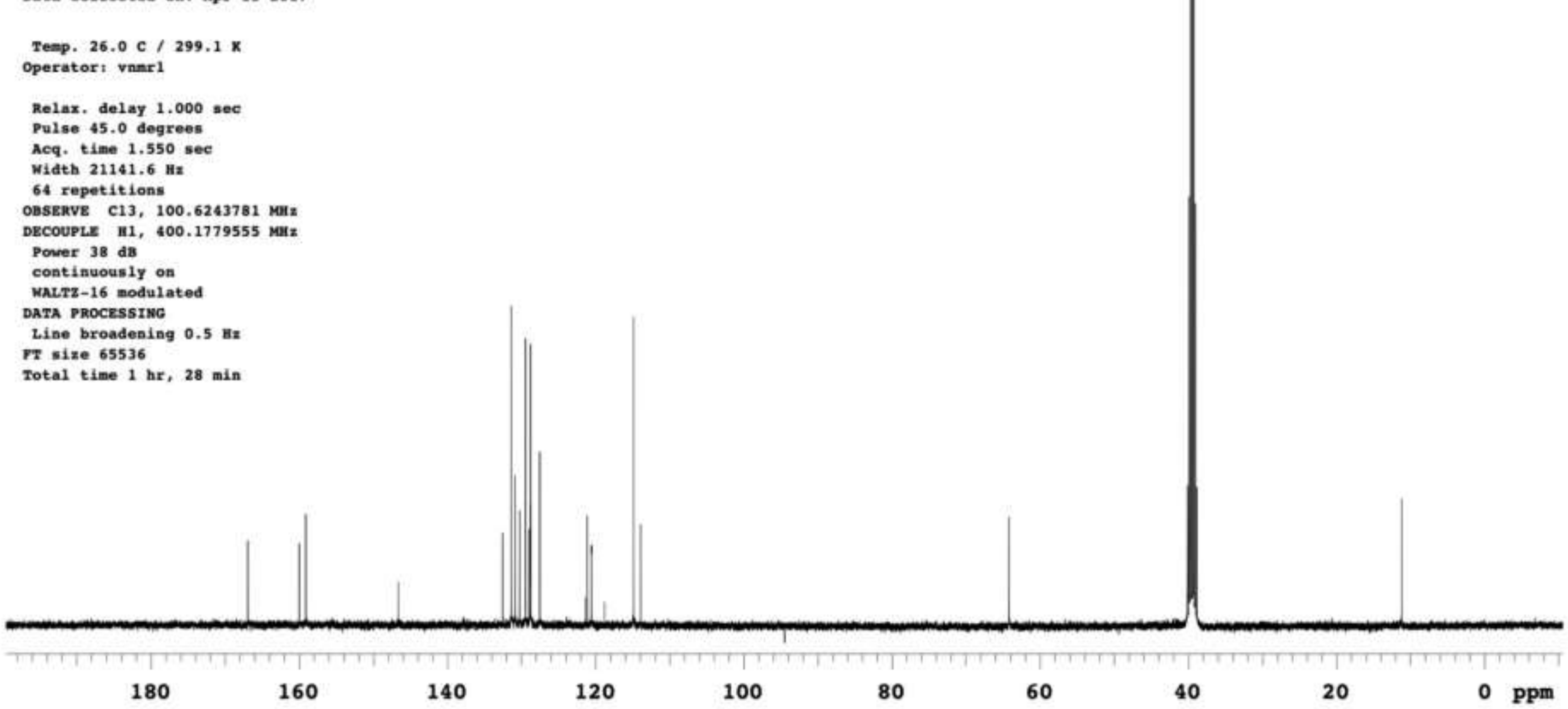

Figure S8. ^{1}H-NMR and ^{13}C-NMR spectrum of Compound **14**

Compound 14

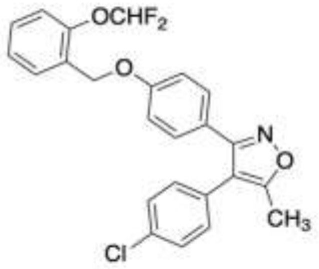

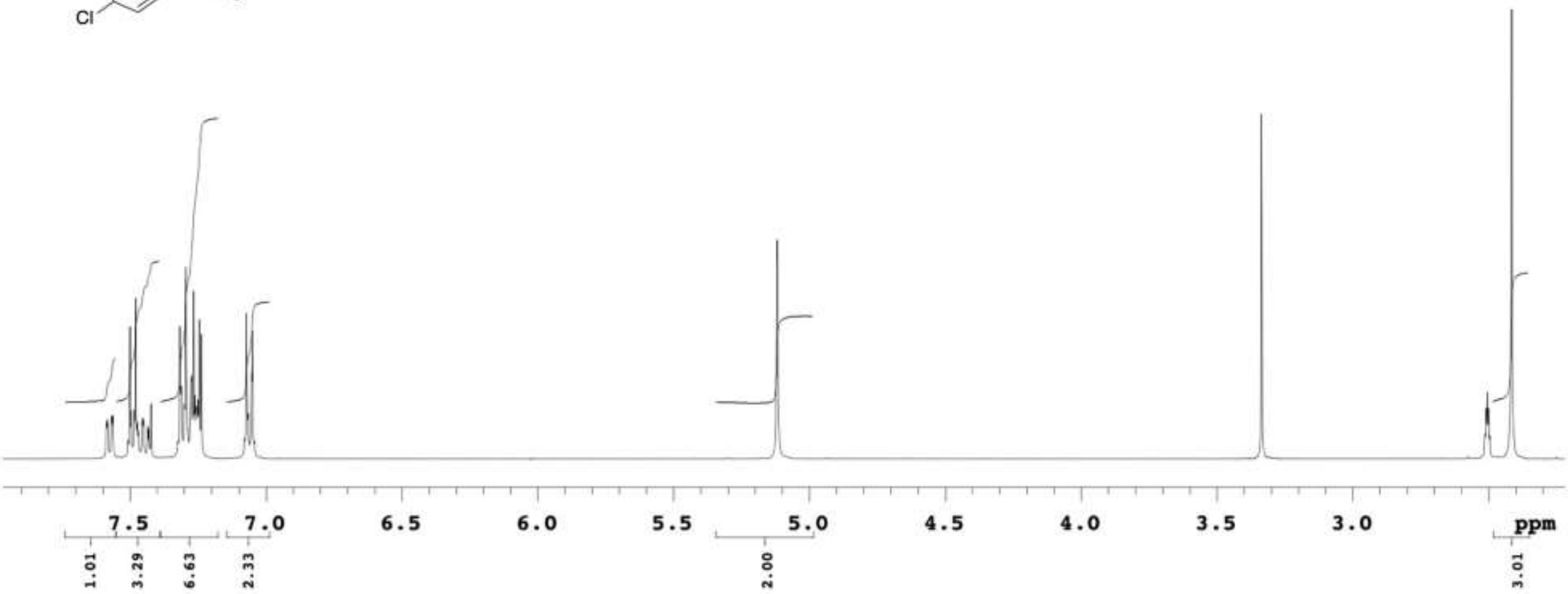

DNZ139

Sample Name:
DNZ139
Data Collected on:
mercury400-mercury400
Archive directory:
/home/vnmr1/vnmrsys/data
Sample directory:
DNZ139_20170415_01
FidFile: current

Pulse Sequence: CARBON (s2pul)
Solvent: dmso
Data collected on: Apr 15 2017

Temp. 26.0 C / 299.1 K
Operator: vnmr1

Relax. delay 1.000 sec
Pulse 45.0 degrees
Acq. time 1.550 sec
Width 21141.6 Hz
64 repetitions
OBSERVE C13, 100.6243781 MHz
DECOUPLE H1, 400.1779555 MHz
Power 38 dB
continuously on
WALTZ-16 modulated
DATA PROCESSING
Line broadening 0.5 Hz
FT size 65536
Total time 1 hr, 28 min

Compound 14

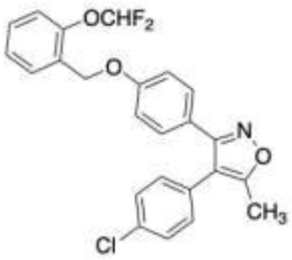

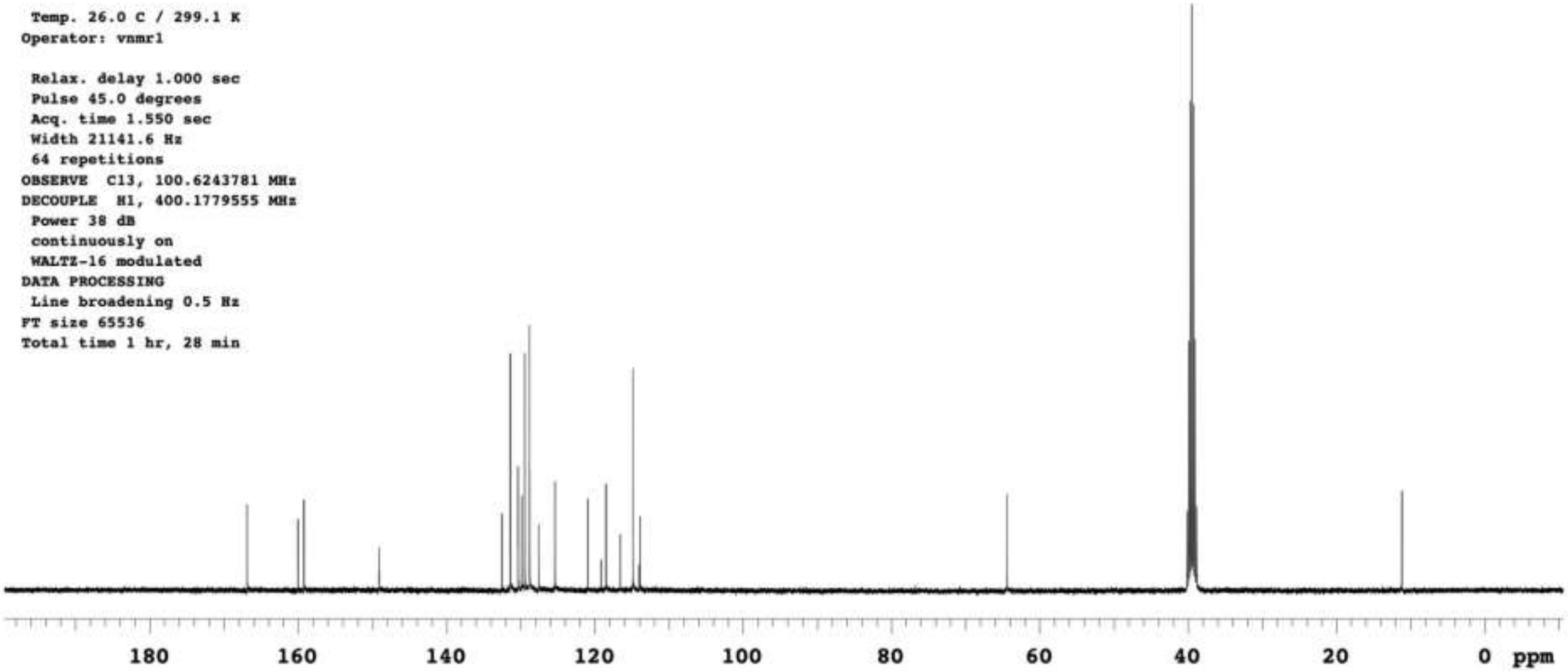

Figure S9. ^{1}H-NMR and ^{13}C-NMR spectrum of Compound **15**

Compound 15

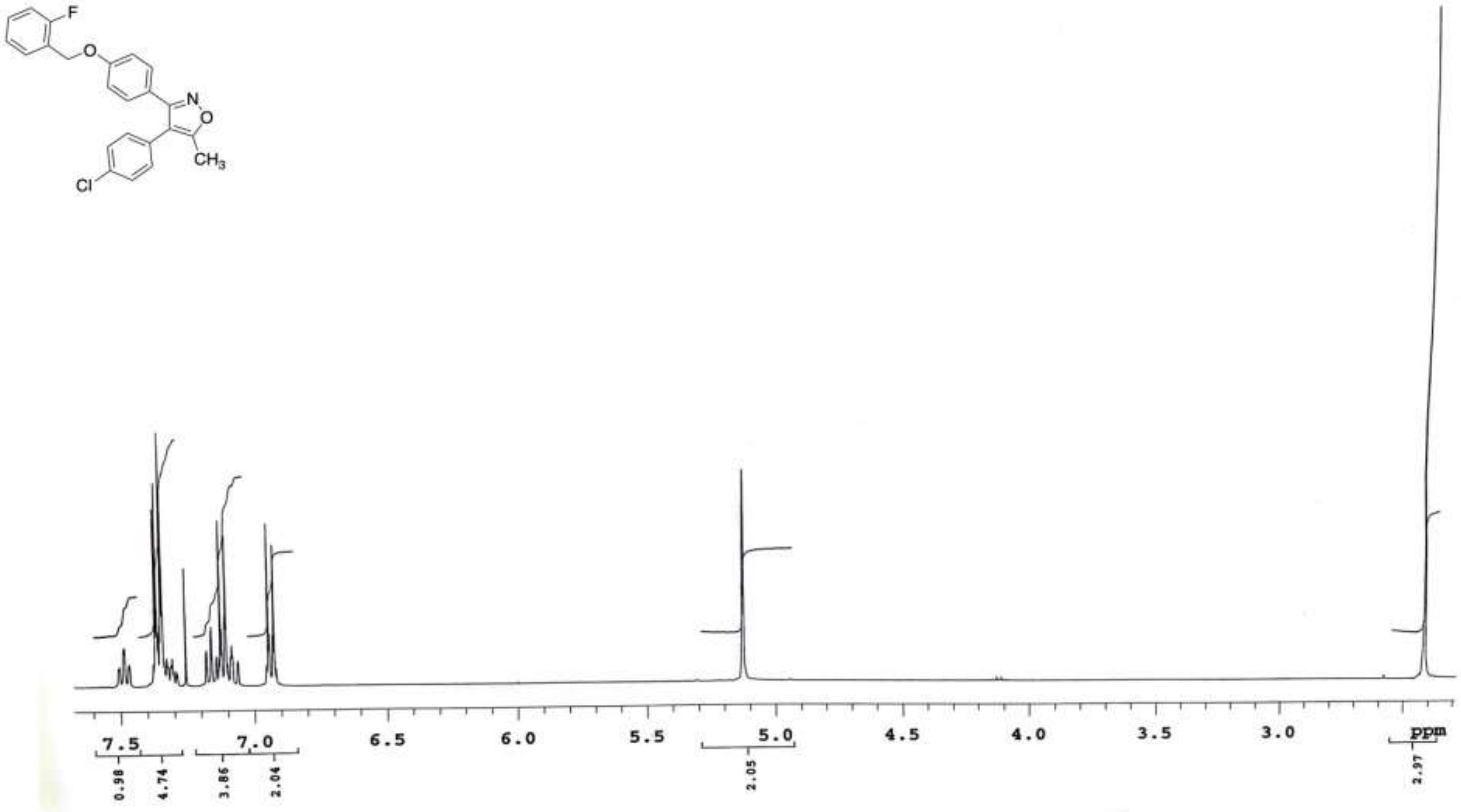

DNZ-76

Sample Name:
DNZ-76
Data Collected on:
mercury400-mercury400
Archive directory:
/home/vnmr1/vnmrsys/data
Sample directory:
DNZ-76_20160824_01
FidFile: CARBON

Pulse Sequence: CARBON (s2pul)
Solvent: cdcl3
Data collected on: Aug 24 2016

Temp. 25.0 C / 298.1 K
Operator: vnmr1

Relax. delay 1.000 sec
Pulse 45.0 degrees
Acq. time 1.304 sec
Width 25125.6 Hz
1000 repetitions
OBSERVE C13, 100.6238513 MHz
DECOUPLE H1, 400.1760547 MHz
Power 38 dB
continuously on
WALTZ-16 modulated
DATA PROCESSING
Line broadening 0.5 Hz
FT size 65536
Total time 40 min

Compound 15

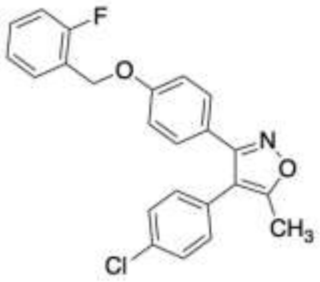

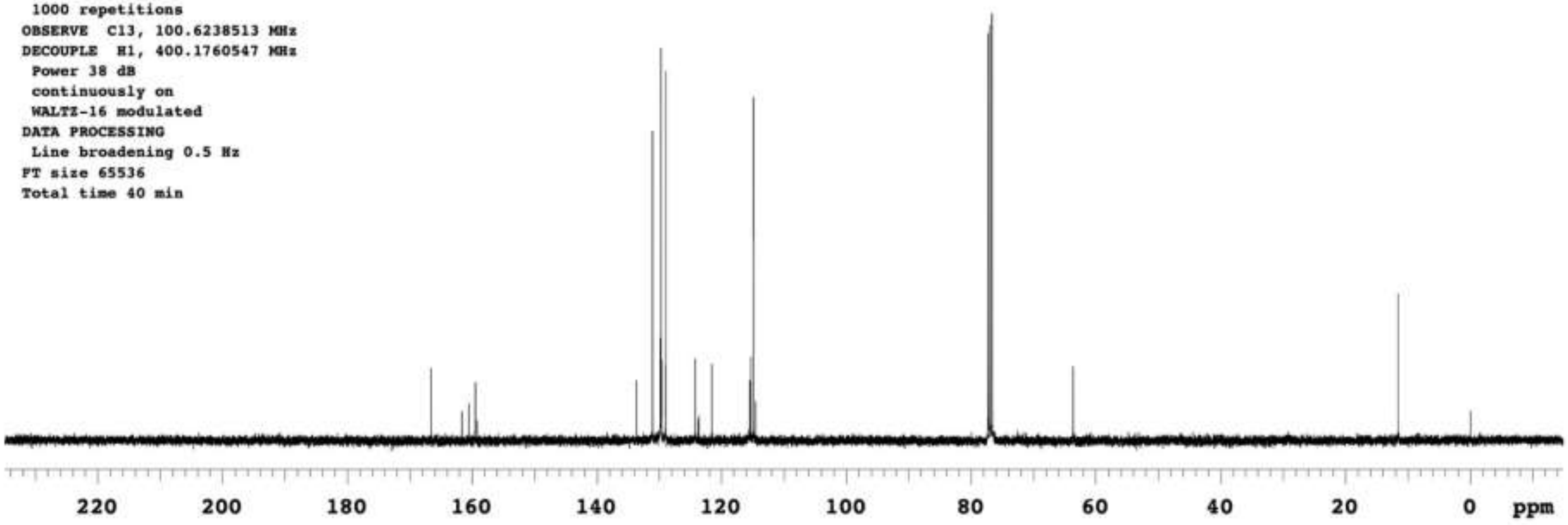

Figure S10. ^{1}H-NMR and ^{13}C-NMR spectrum of Compound **16**

Compound 16

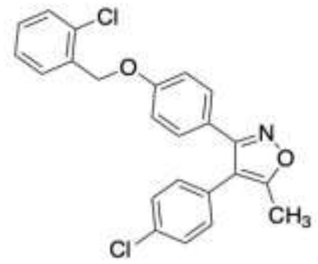

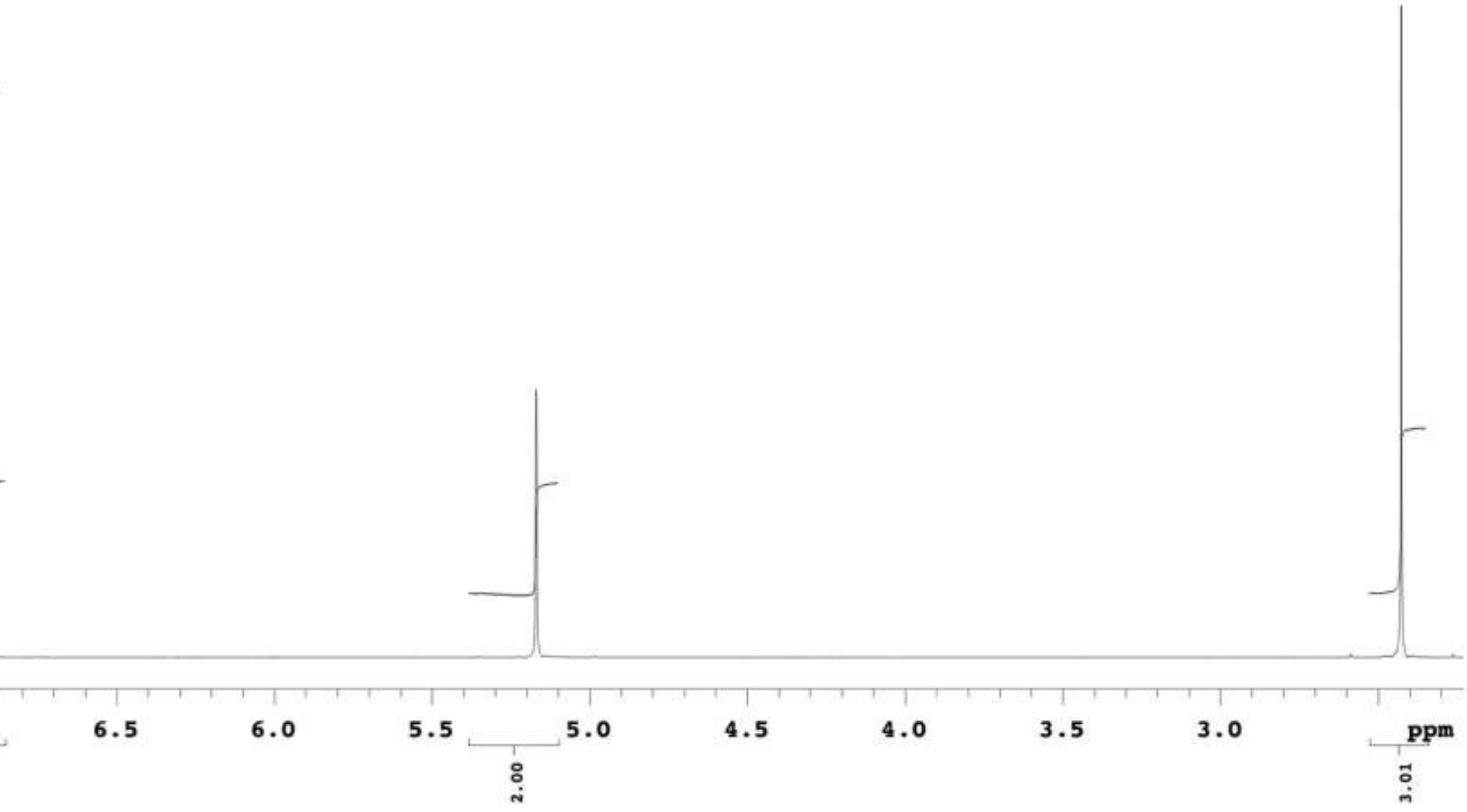

DNZ103

Sample Name:
DNZ103
Data Collected on:
mercury400-mercury400
Archive directory:
/home/vnmr1/vnmrsys/data
Sample directory:
DNZ103_20161208_01
FidFile: CARBON_01

Pulse Sequence: CARBON (s2pul)
Solvent: cdcl3
Data collected on: Dec 8 2016

Temp. 25.0 C / 298.1 K
Operator: vnmr1

Relax. delay 1.000 sec
Pulse 45.0 degrees
Acq. time 1.304 sec
Width 25125.6 Hz
858 repetitions
OBSERVE C13, 100.6238513 MHz
DECOUPLE H1, 400.1760547 MHz
Power 38 dB
continuously on
WALTZ-16 modulated
DATA PROCESSING
Line broadening 0.5 Hz
FT size 65536
Total time 34 min

Compound 16

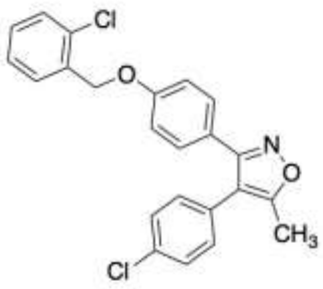

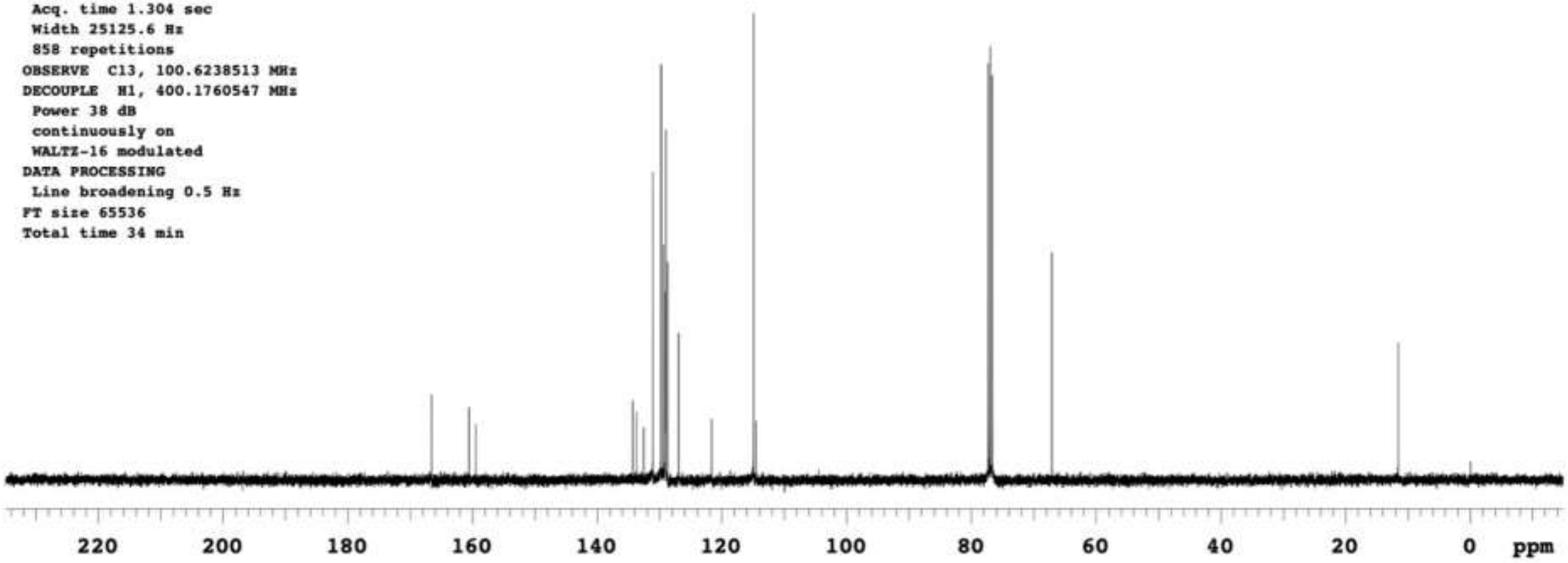

Figure S11. ^{1}H-NMR and ^{13}C-NMR spectrum of Compound **17**

Compound 17

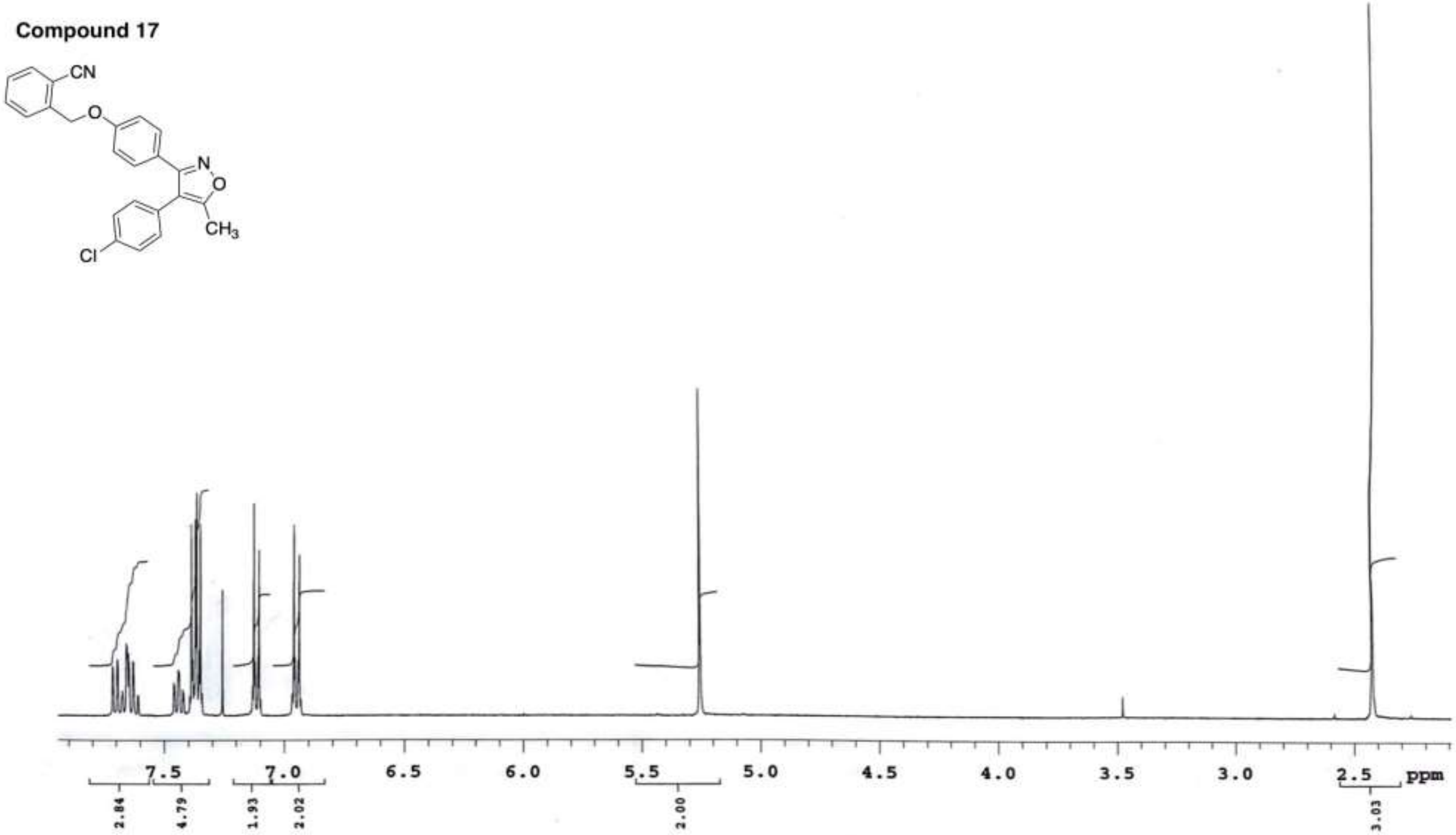

DNZ51

Sample Name:
DNZ51
Data Collected on:
mercury400-mercury400
Archive directory:
/home/vnmr1/vnmrsys/data
Sample directory:
DNZ51_20160824_01
FidFile: CARBON

Pulse Sequence: CARBON (s2pul)
Solvent: cdcl3
Data collected on: Aug 24 2016

Temp. 25.0 C / 298.1 K
Operator: vnmr1

Relax. delay 1.000 sec
Pulse 45.0 degrees
Acq. time 1.304 sec
Width 25125.6 Hz
2000 repetitions
OBSERVE C13, 100.6238513 MHz
DECOUPLE H1, 400.1760547 MHz
Power 38 dB
continuously on
WALTZ-16 modulated
DATA PROCESSING
Line broadening 0.5 Hz
FT size 65536
Total time 1 hr, 20 min

Compound 17

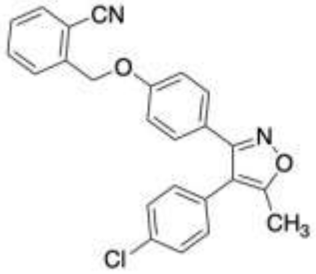

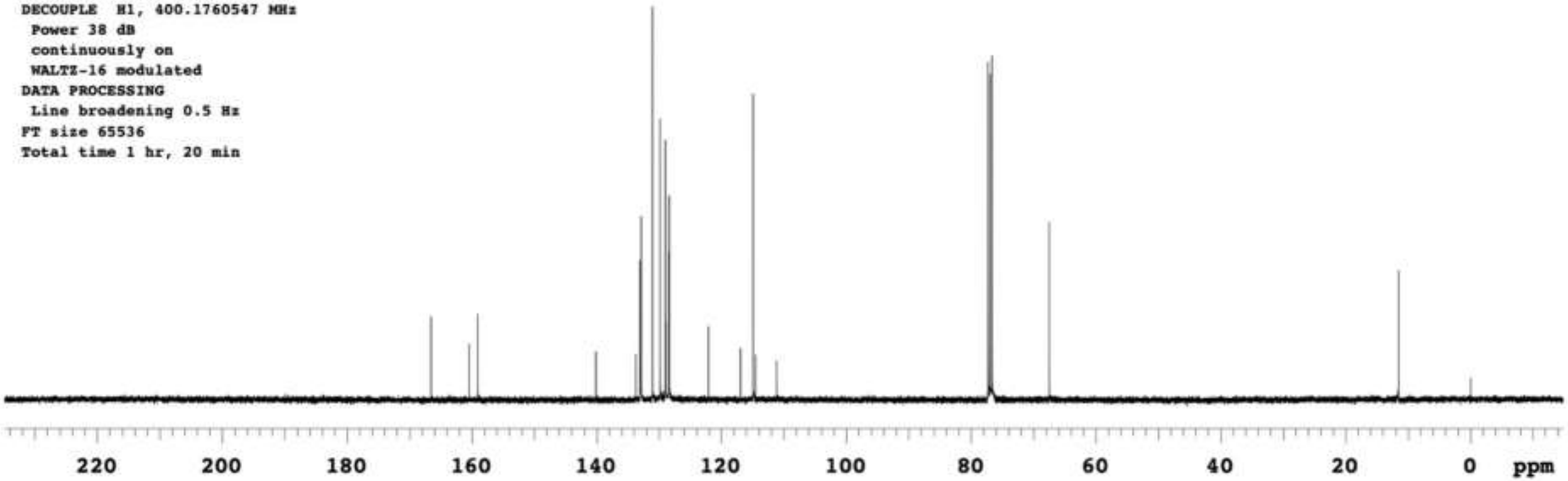

Figure S12. ^{1}H-NMR and ^{13}C-NMR spectrum of Compound **18**

Compound 18

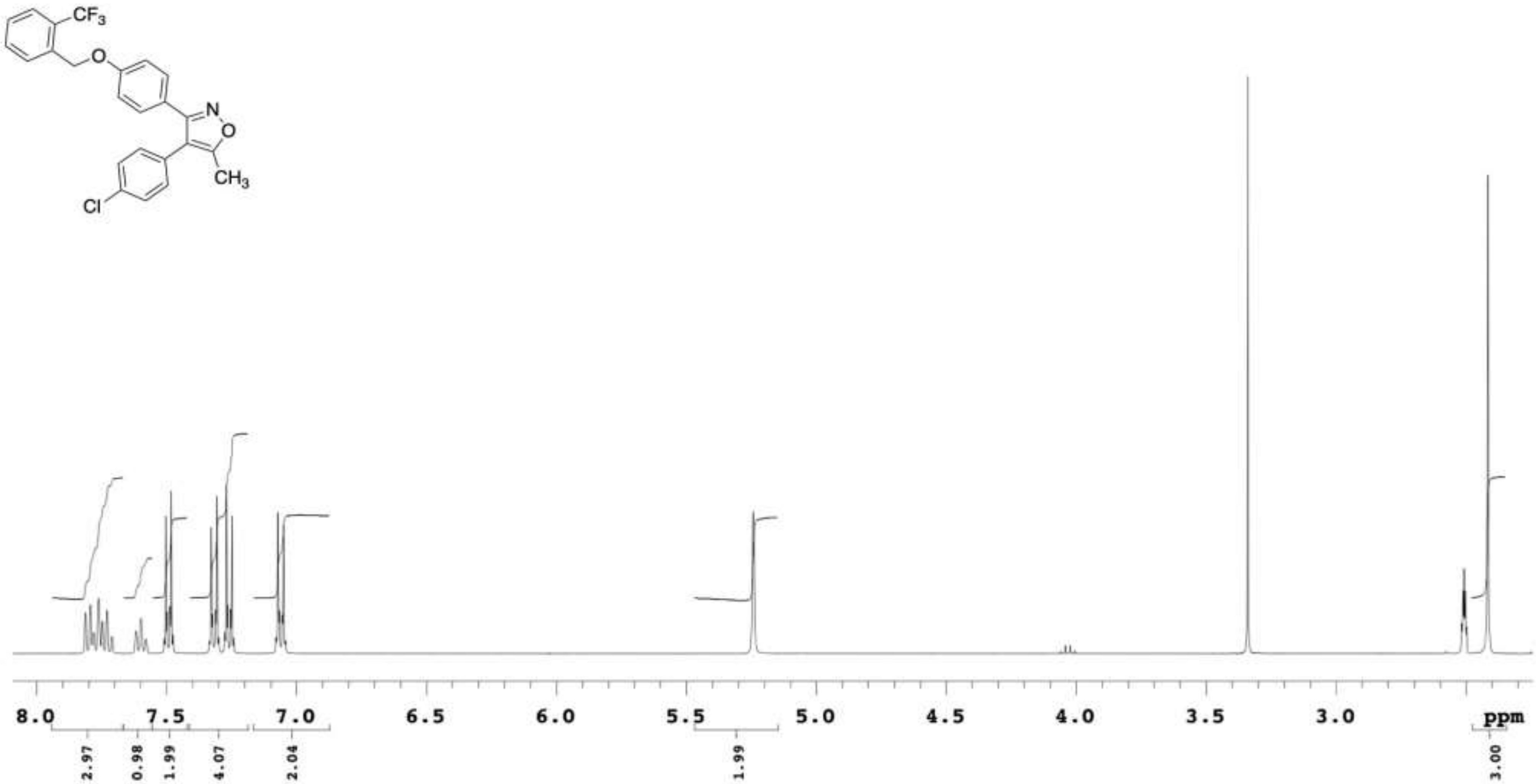

DNZ138

Sample Name:
DNZ138
Data Collected on:
mercury400-mercury400
Archive directory:
/home/vnmr1/vnmrsys/data
Sample directory:
DNZ138_20170416_01
FidFile: current

Pulse Sequence: CARBON (s2pul)
Solvent: dmso
Data collected on: Apr 16 2017

Temp. 26.0 C / 299.1 K
Operator: vnmr1

Relax. delay 1.000 sec
Pulse 45.0 degrees
Acq. time 1.550 sec
Width 21141.6 Hz
128 repetitions
OBSERVE C13, 100.6243781 MHz
DECOUPLE H1, 400.1779555 MHz
Power 38 dB
continuously on
WALTZ-16 modulated
DATA PROCESSING
Line broadening 0.5 Hz
FT size 65536
Total time 3 hr, 40 min

Compound 18

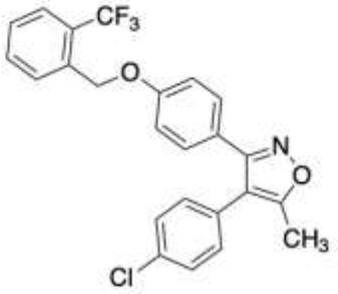

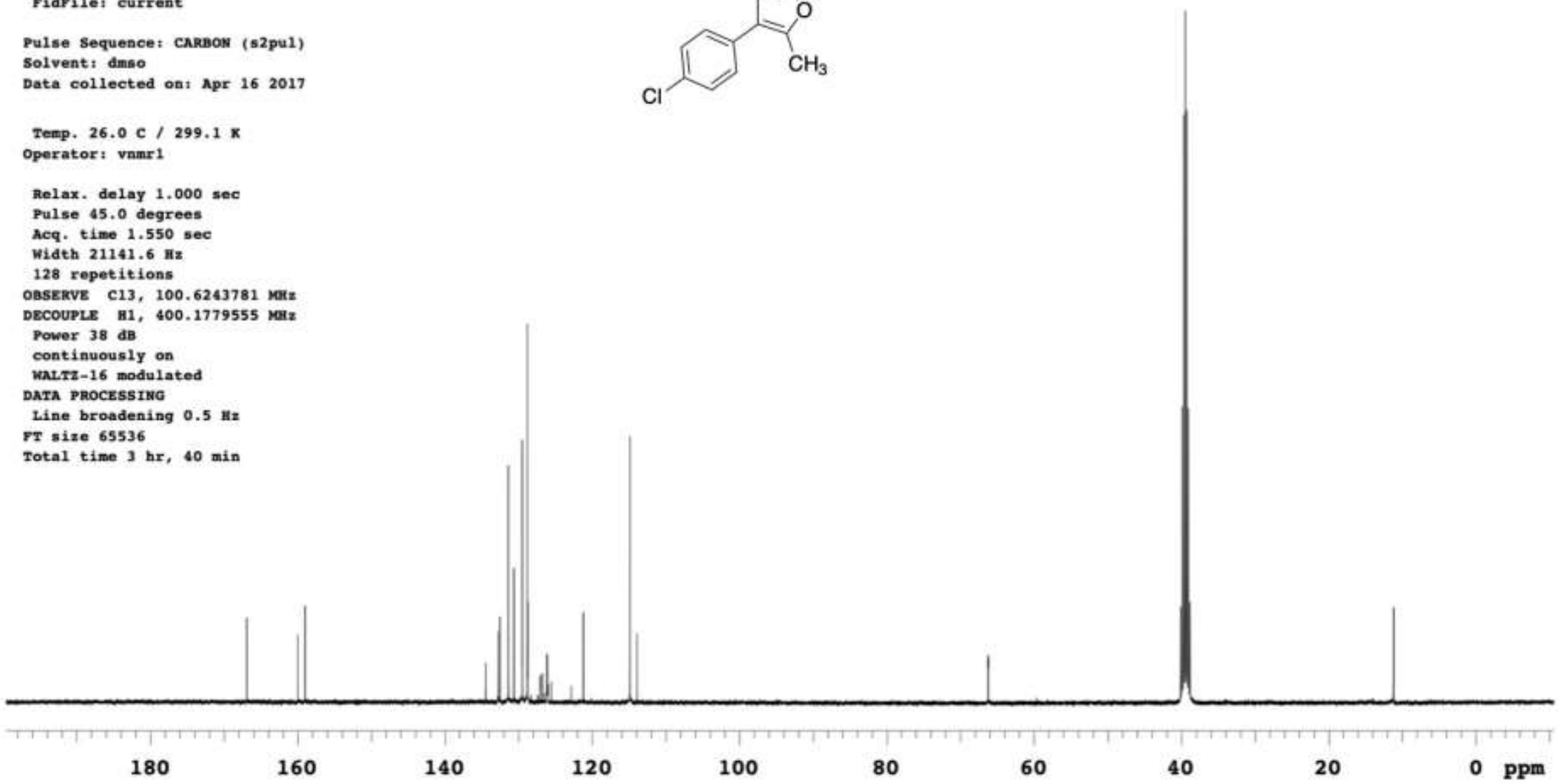

Figure S13. ^{1}H-NMR and ^{13}C-NMR spectrum of Compound **19**

Compound 19

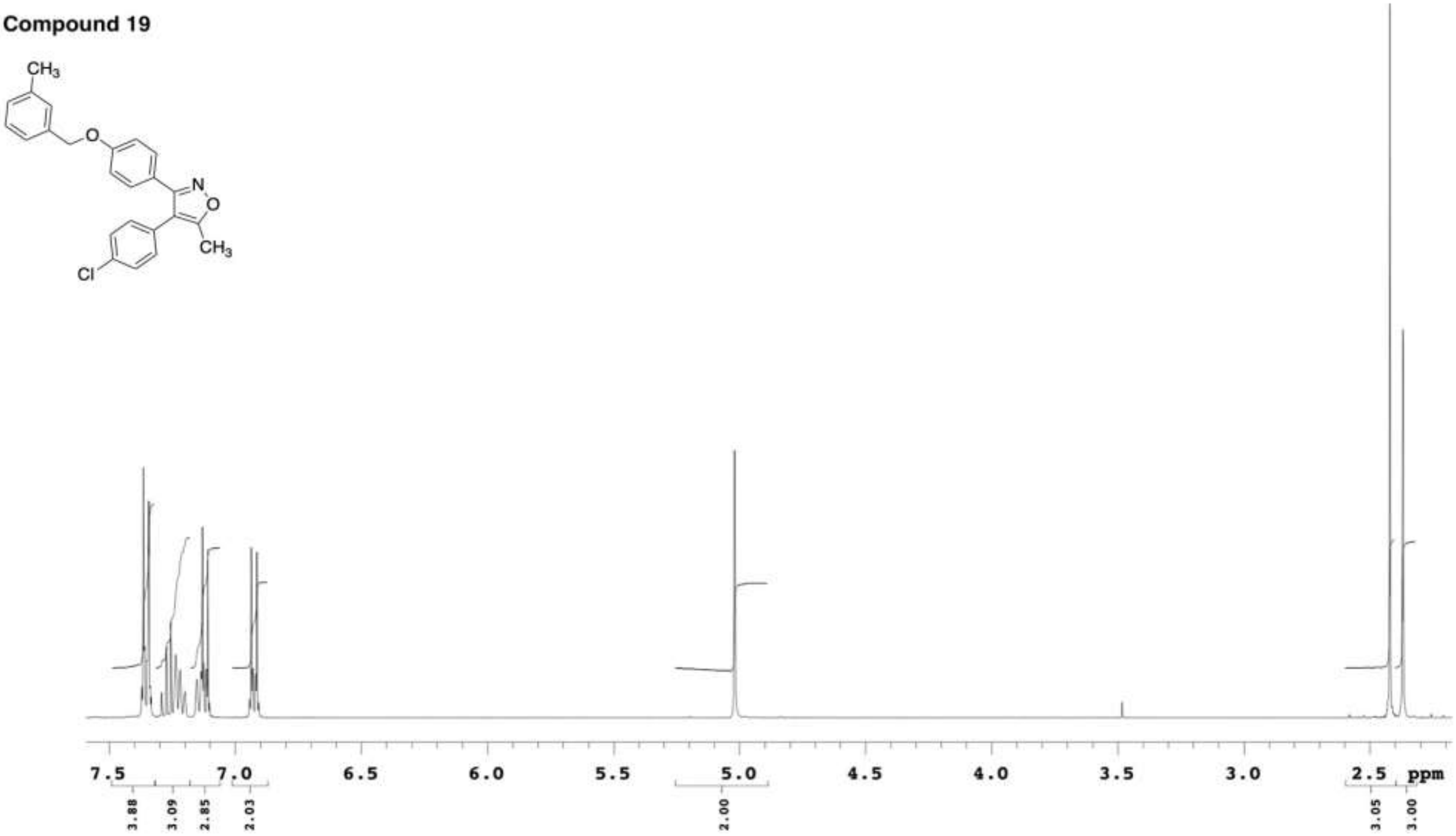

DNZ89

Sample Name:
DNZ89
Data Collected on:
mercury400-mercury400
Archive directory:
/home/vnmr1/vnmrsys/data
Sample directory:
DNZ89_20161124_01
FidFile: CARBON_01

Pulse Sequence: CARBON (s2pul)
Solvent: cdcl3
Data collected on: Nov 24 2016

Temp. 26.0 C / 299.1 K
Operator: vnmr1

Relax. delay 1.000 sec
Pulse 45.0 degrees
Acq. time 1.304 sec
Width 25125.6 Hz
2000 repetitions
OBSERVE C13, 100.6238513 MHz
DECOUPLE H1, 400.1760547 MHz
Power 38 dB
continuously on
WALTZ-16 modulated
DATA PROCESSING
Line broadening 0.5 Hz
FT size 65536
Total time 1 hr, 20 min

Compound 19

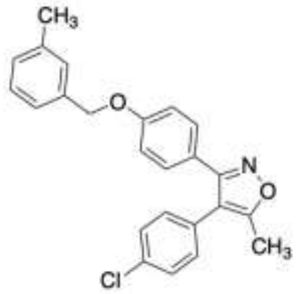

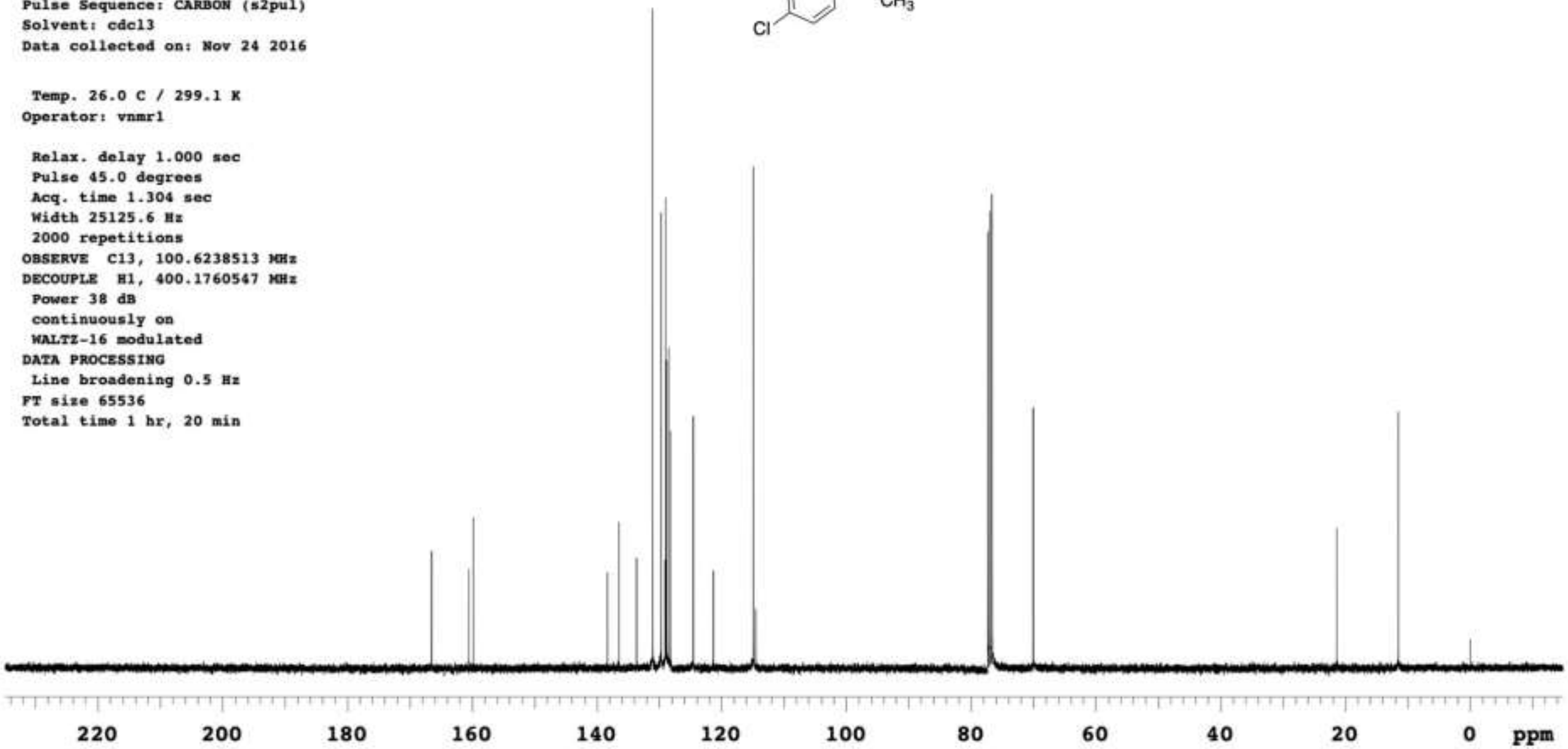

Figure S14. ^{1}H-NMR and ^{13}C-NMR spectrum of Compound **20**

Compound 20

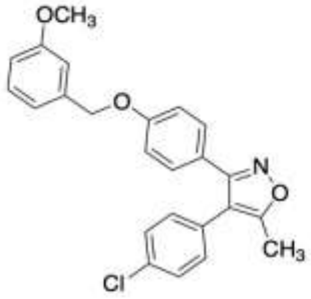

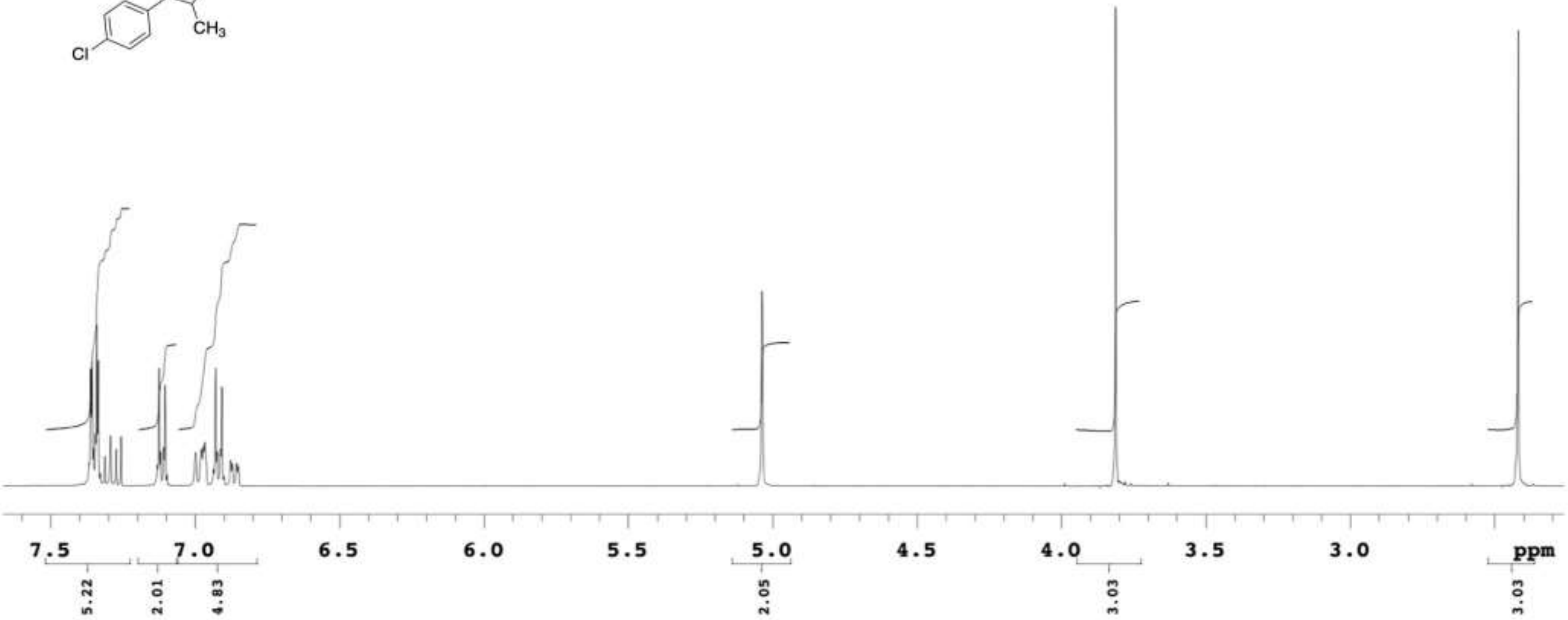

Compound 20

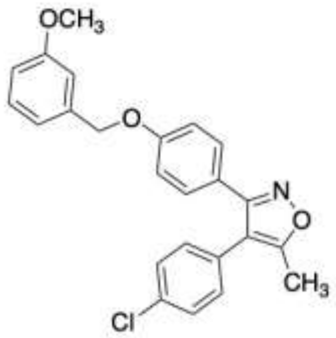

DNZ-85

Sample Name:
DNZ-85
Data Collected on:
mercury400-mercury400
Archive directory:
/home/vnmr1/vnmrsys/data
Sample directory:
DNZ-85_20161123_01
FidFile: CARBON_01

Pulse Sequence: CARBON (s2pul)
Solvent: cdcl3
Data collected on: Nov 23 2016

Temp. 25.0 C / 298.1 K
Operator: vnmr1

Relax. delay 1.000 sec
Pulse 45.0 degrees
Acq. time 1.304 sec
Width 25125.6 Hz
2000 repetitions
OBSERVE C13, 100.6238513 MHz
DECOUPLE H1, 400.1760547 MHz
Power 38 dB
continuously on
WALTZ-16 modulated
DATA PROCESSING
Line broadening 0.5 Hz
FT size 65536
Total time 1 hr, 20 min

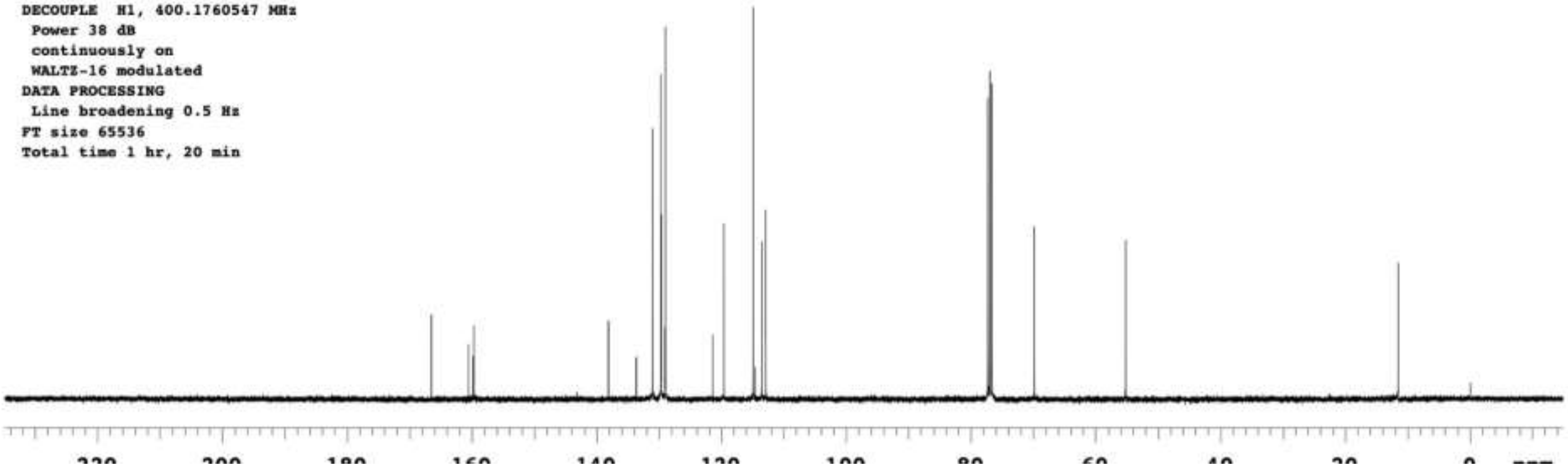

Figure S15. ^{1}H-NMR and ^{13}C-NMR spectrum of Compound **21**

Compound 21

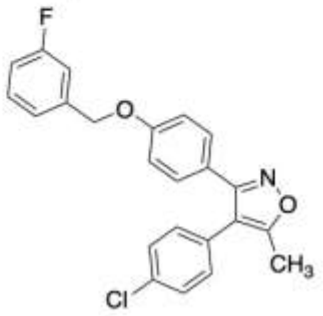

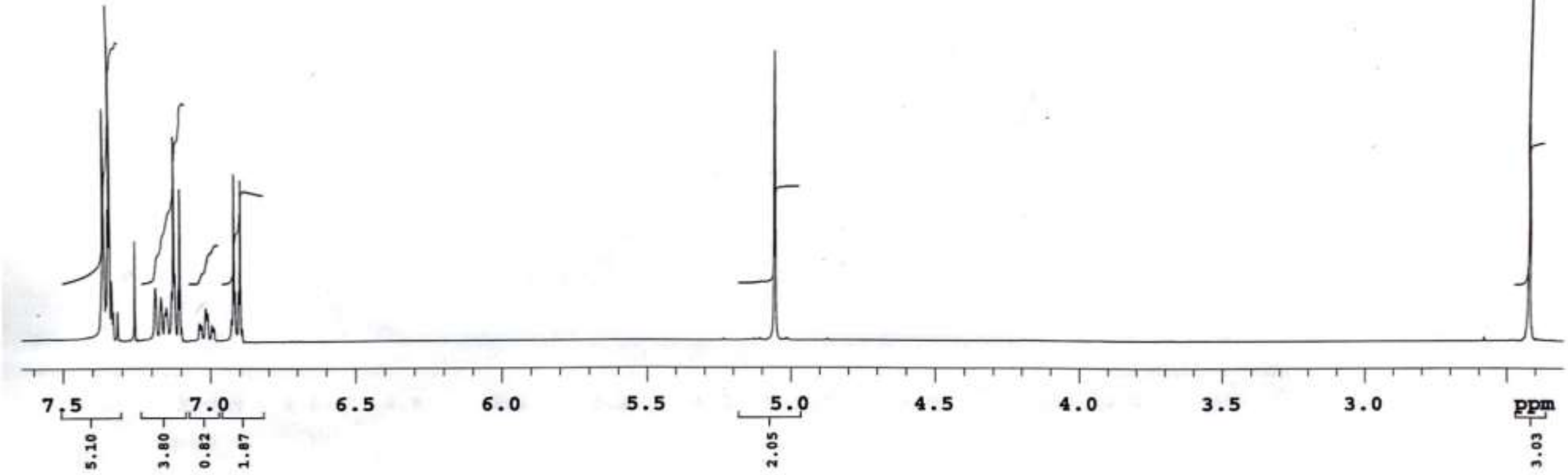

DNZ77

Sample Name:
DNZ77
Data Collected on:
mercury400-mercury400
Archive directory:
/home/vnmr1/vnmrsys/data
Sample directory:
DNZ77_20161001_01
FidFile: current

Pulse Sequence: CARBON (s2pul)
Solvent: cdcl3
Data collected on: Oct 1 2016

Temp. 25.0 C / 298.1 K
Operator: vnmr1

Relax. delay 1.000 sec
Pulse 45.0 degrees
Acq. time 1.550 sec
Width 21141.6 Hz
64 repetitions
OBSERVE C13, 100.6238513 MHz
DECOUPLE H1, 400.1760547 MHz
Power 38 dB
continuously on
WALTZ-16 modulated
DATA PROCESSING
Line broadening 0.5 Hz
FT size 65536
Total time 1 hr, 6 min

Compound 21

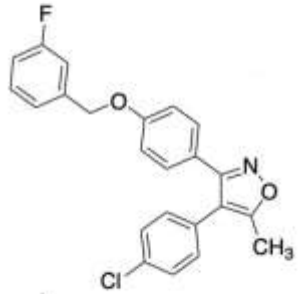

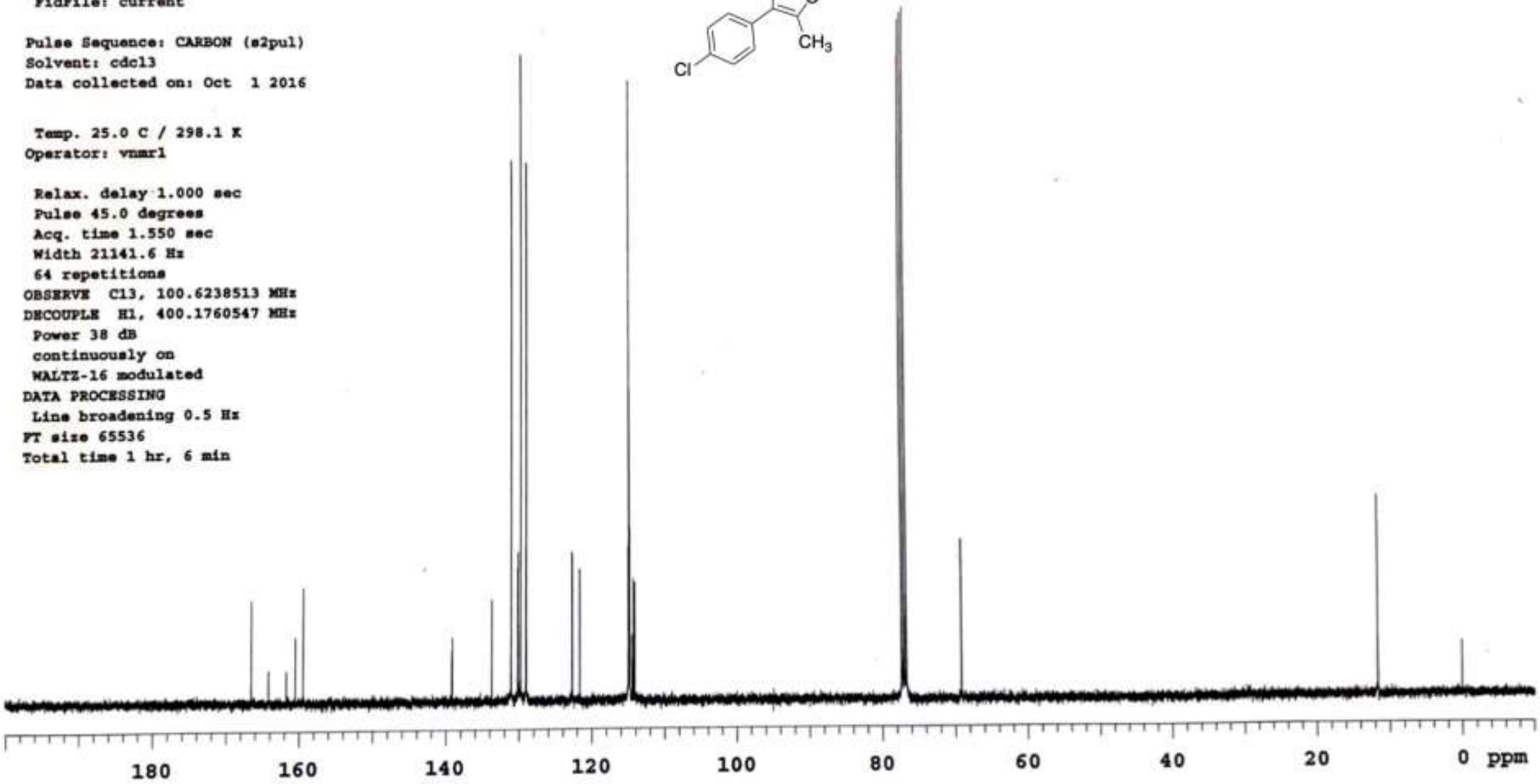

Figure S16. ¹H-NMR and ¹³C-NMR spectrum of Compound **22**

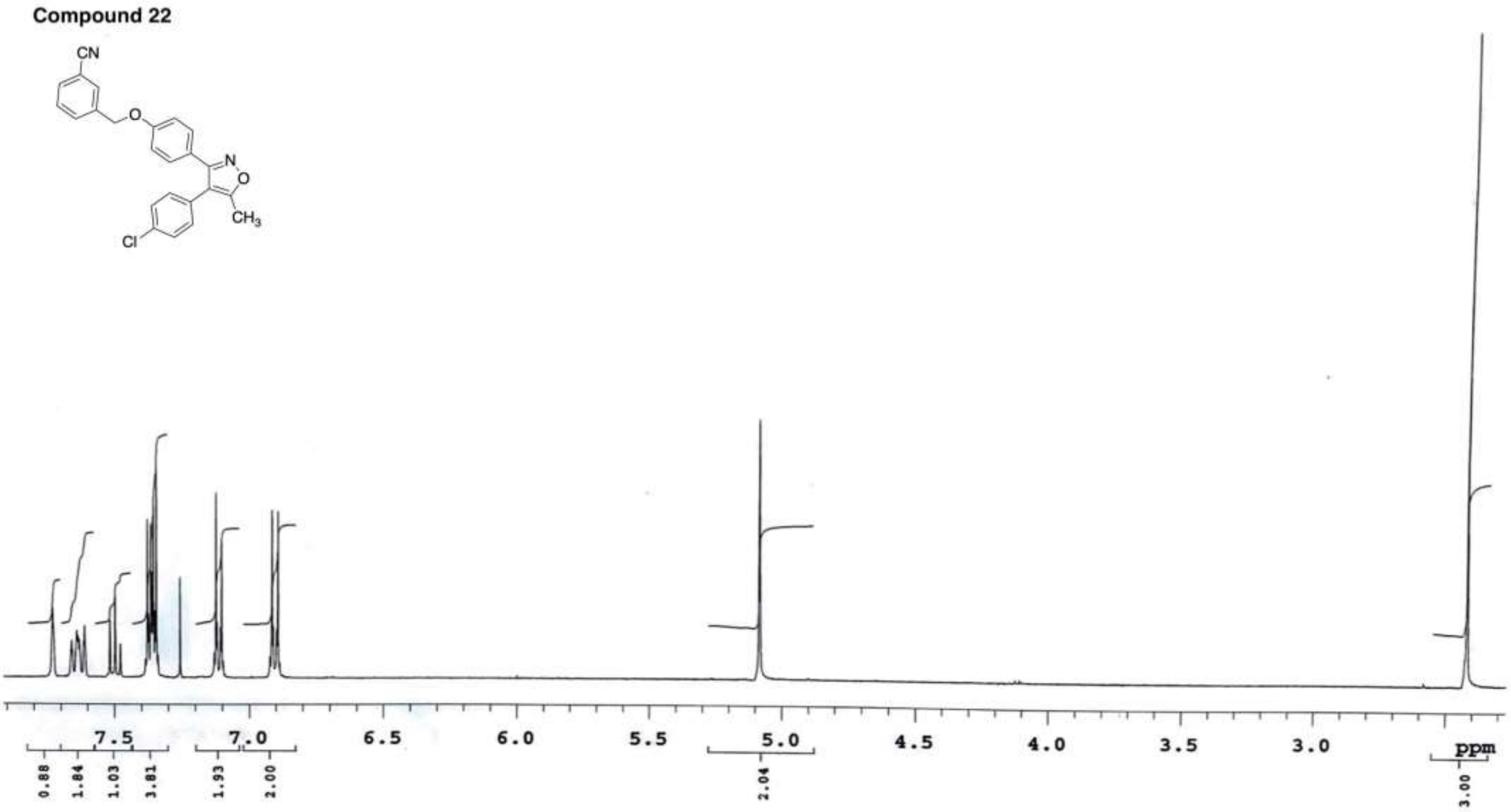

DNZ63

Sample Name:
DNZ63
Data Collected on:
mercury400-mercury400
Archive directory:
/home/vnmr1/vnmrsys/data
Sample directory:
DNZ63_20160824_01
FidFile: CARBON_01

Pulse Sequence: CARBON (s2pul)
Solvent: cdcl3
Data collected on: Aug 24 2016

Temp. 25.0 C / 298.1 K
Operator: vnmr1

Relax. delay 1.000 sec
Pulse 45.0 degrees
Acq. time 1.304 sec
Width 25125.6 Hz
1000 repetitions
OBSERVE C13, 100.6238513 MHz
DECOUPLE H1, 400.1760547 MHz
Power 38 dB
continuously on
WALTZ-16 modulated
DATA PROCESSING
Line broadening 0.5 Hz
FT size 65536
Total time 40 min

Compound 22

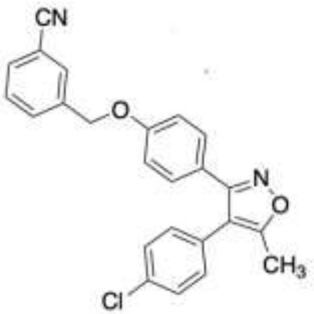

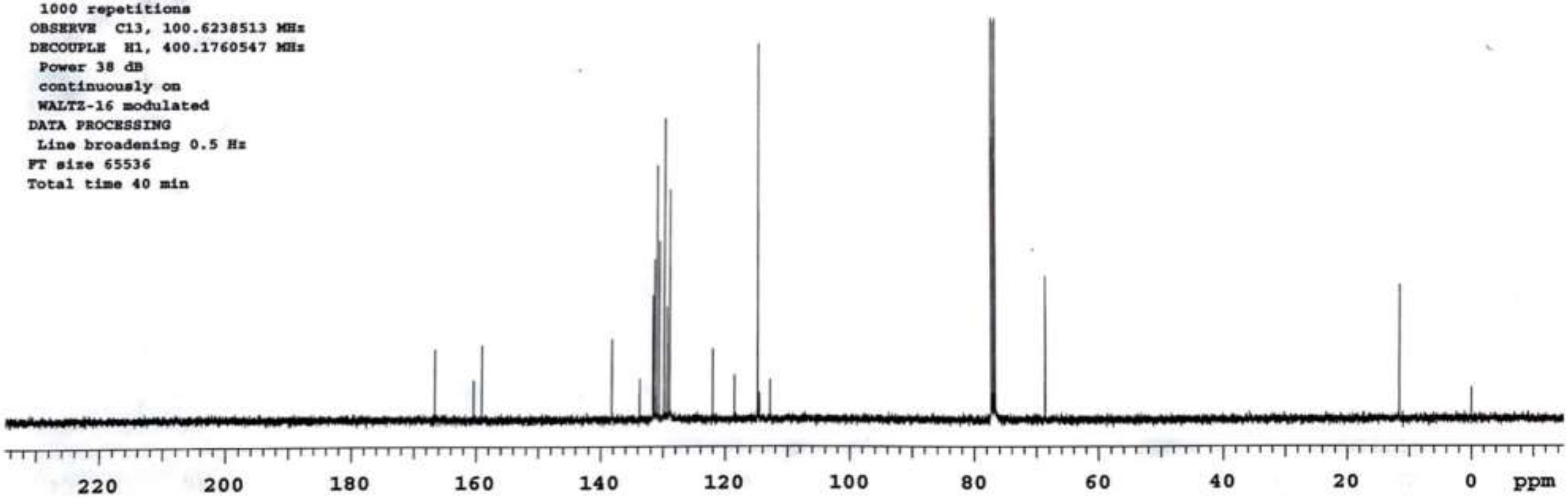

Figure S17. ^{1}H-NMR and ^{13}C-NMR spectrum of Compound **23**

Compound 23

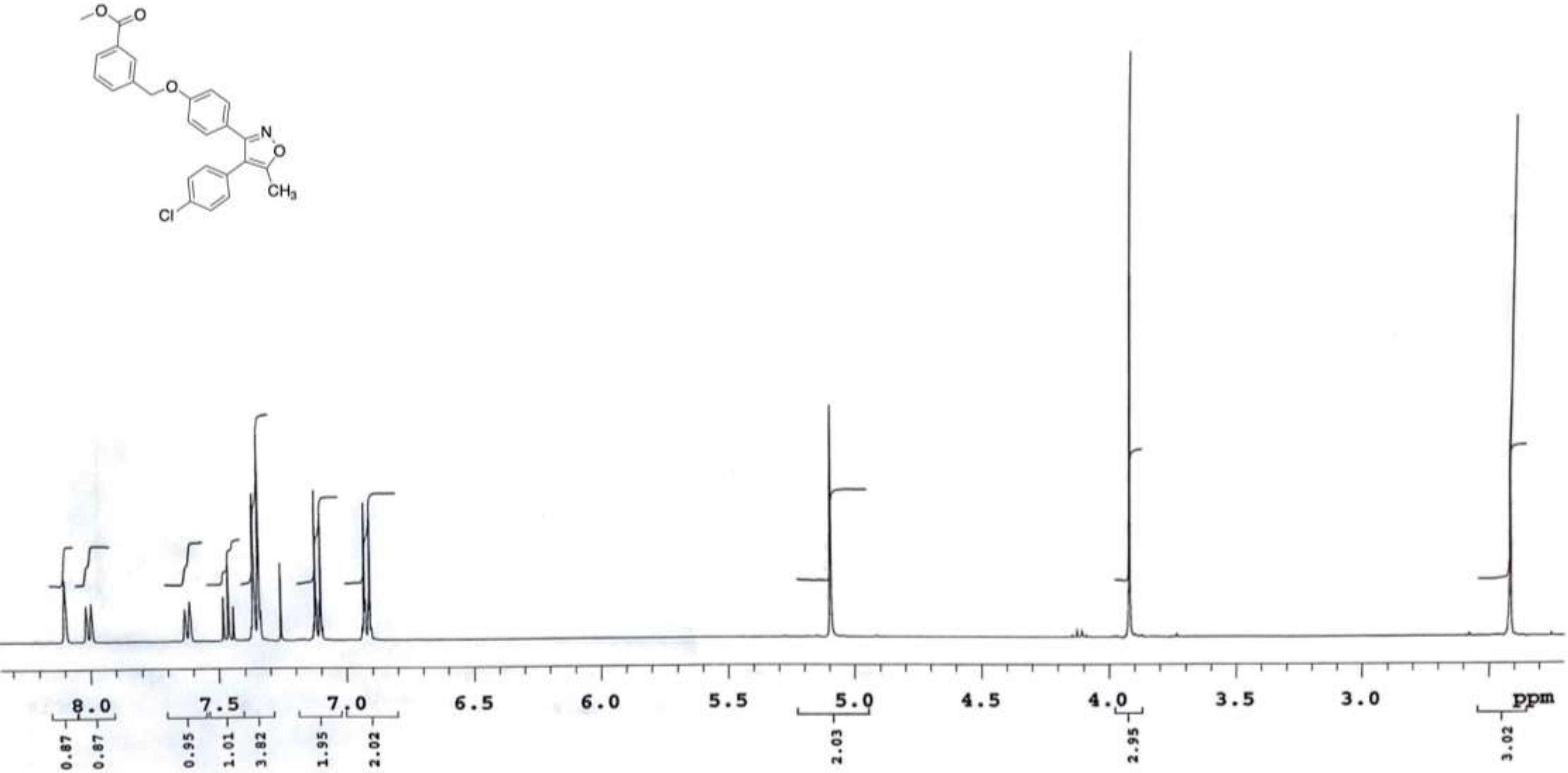

DNZ73

Sample Name:
DNZ73
Data Collected on:
mercury400-mercury400
Archive directory:
/home/vnmr1/vnmrsys/data
Sample directory:
DNZ73_20160824_01
FidFile: CARBON_01

Pulse Sequence: CARBON (s2pul)
Solvent: cdcl3
Data collected on: Aug 24 2016

Temp. 25.0 C / 298.1 K
Operator: vnmr1

Relax. delay 1.000 sec
Pulse 45.0 degrees
Acq. time 1.304 sec
Width 25125.6 Hz
1000 repetitions
OBSERVE C13, 100.6238513 MHz
DECOUPLE H1, 400.1760547 MHz
Power 38 dB
continuously on
WALTZ-16 modulated
DATA PROCESSING
Line broadening 0.5 Hz
FT size 65536
Total time 40 min

Compound 23

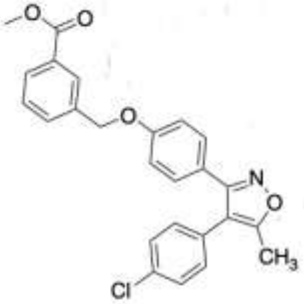

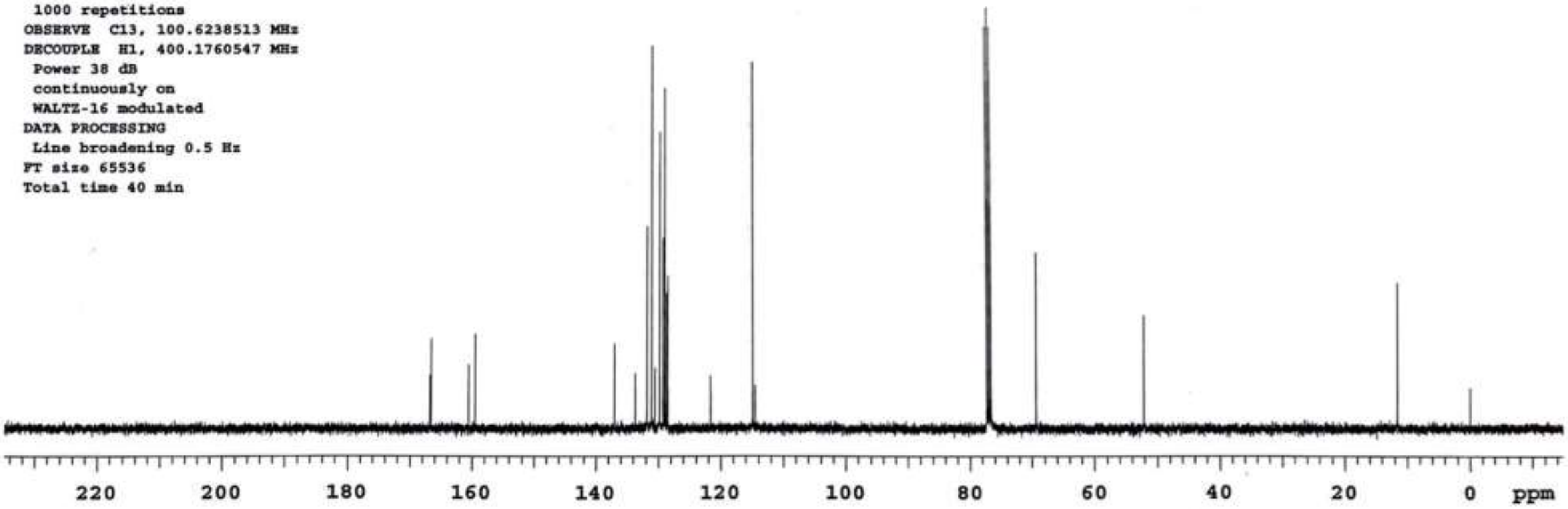

Figure S18. ^{1}H-NMR and ^{13}C-NMR spectrum of Compound **24**

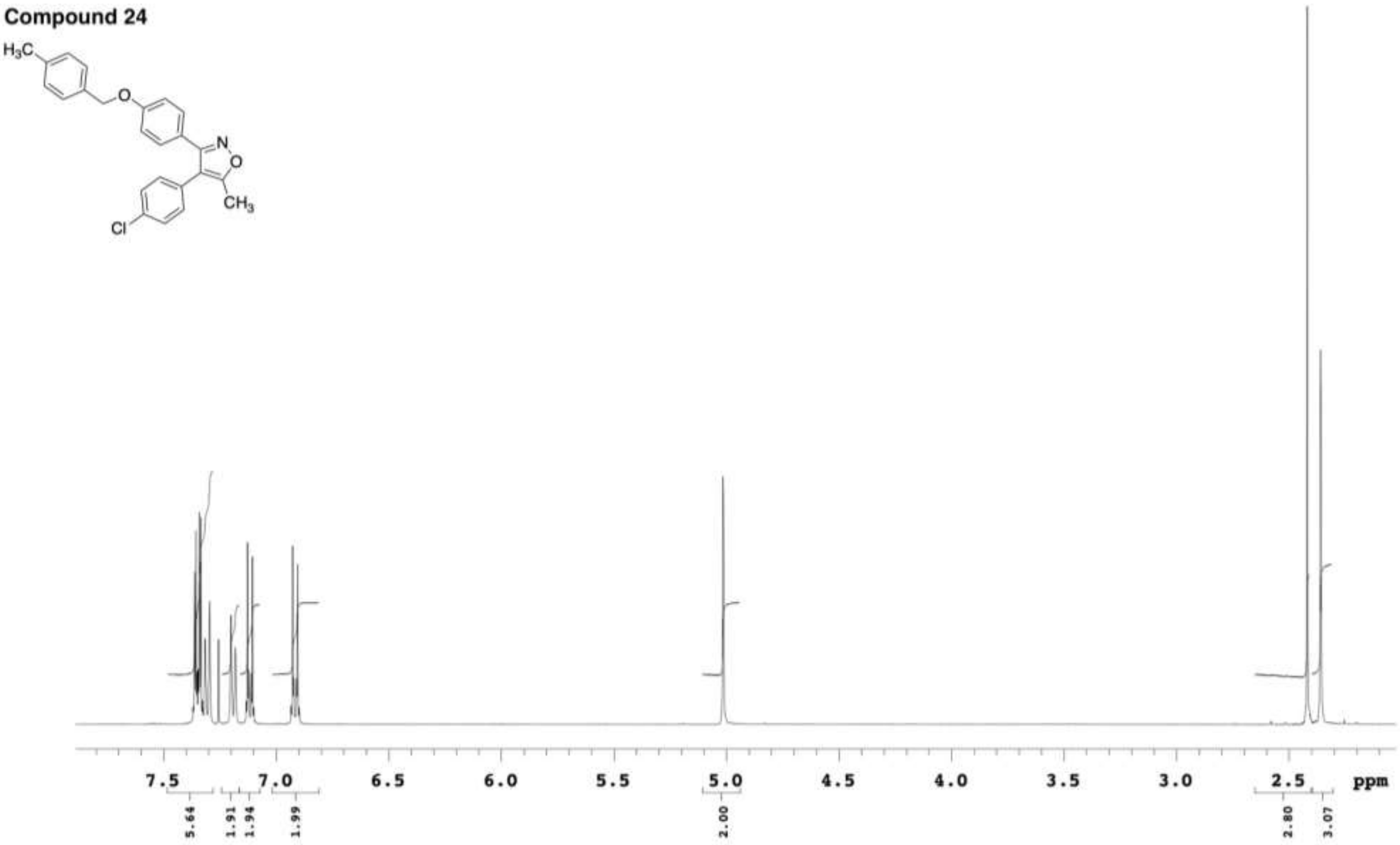

DNZ90

Sample Name:
DNZ90
Data Collected on:
mercury400-mercury400
Archive directory:
/home/vnmr1/vnmrsys/data
Sample directory:
DNZ90_20161124_01
FidFile: CARBON_01

Pulse Sequence: CARBON (s2pul)
Solvent: cdcl3
Data collected on: Nov 24 2016

Temp. 26.0 C / 299.1 K
Operator: vnmr1

Relax. delay 1.000 sec
Pulse 45.0 degrees
Acq. time 1.304 sec
Width 25125.6 Hz
1512 repetitions
OBSERVE C13, 100.6238513 MHz
DECOUPLE H1, 400.1760547 MHz
Power 38 dB
continuously on
WALTZ-16 modulated
DATA PROCESSING
Line broadening 0.5 Hz
FT size 65536
Total time 1 hr

Compound 24

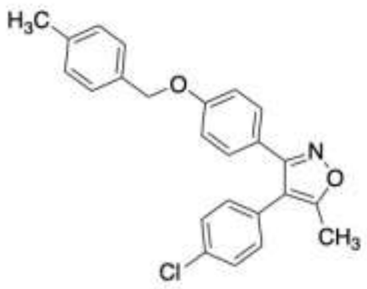

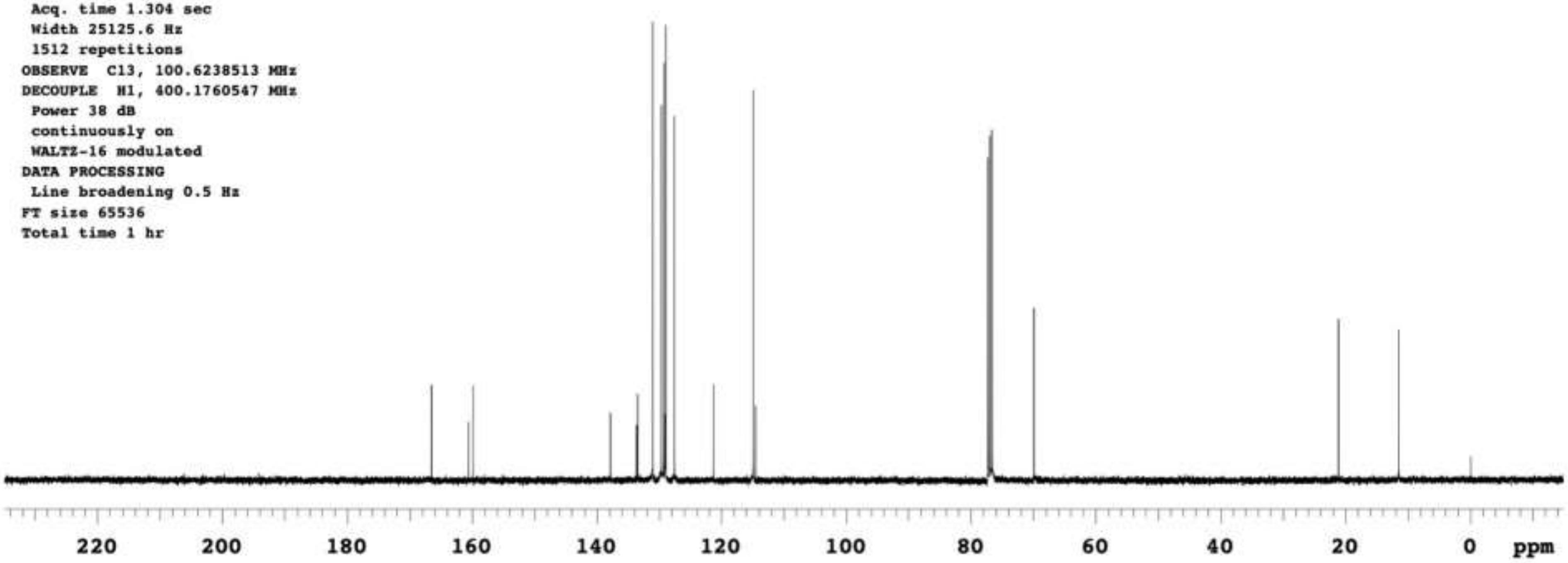

Figure S19. ^{1}H-NMR and ^{13}C-NMR spectrum of Compound **25**

Compound 25

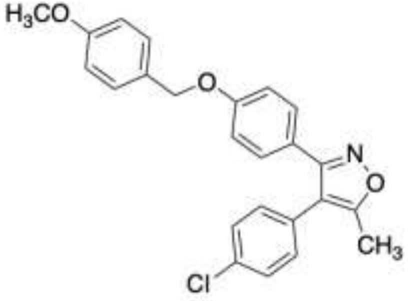

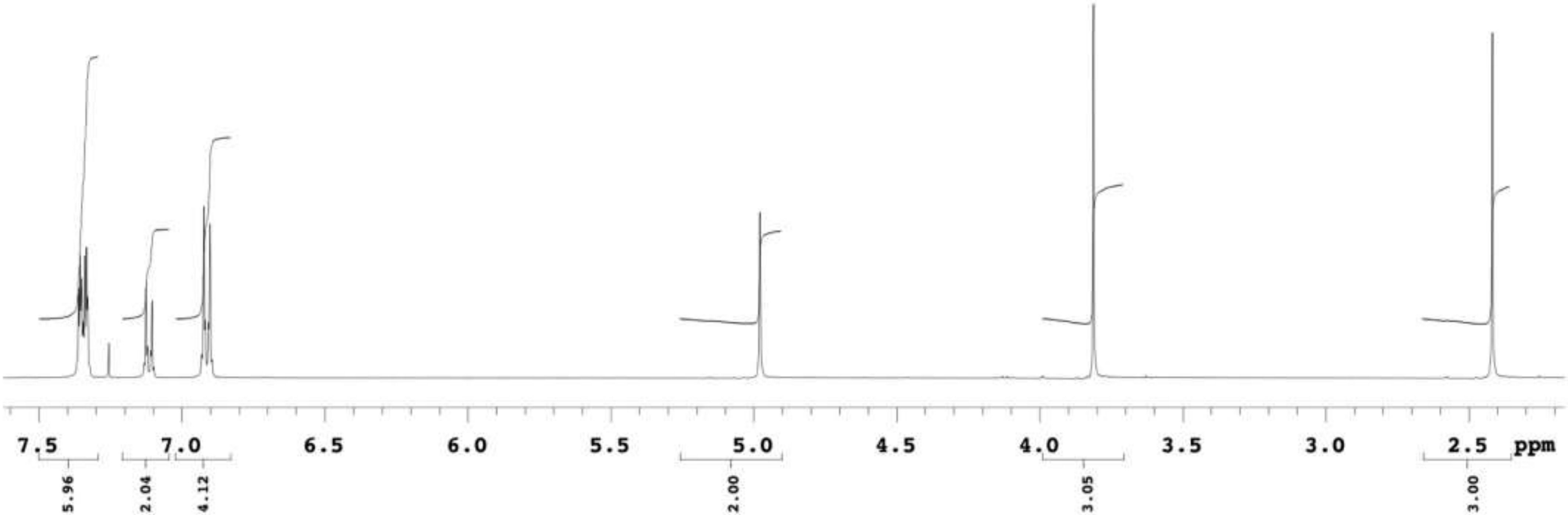

DNZ87

Sample Name:
DNZ87
Data Collected on:
mercury400-mercury400
Archive directory:
/home/vnmr1/vnmrsys/data
Sample directory:
DNZ87_20161124_01
FidFile: CARBON_01

Pulse Sequence: CARBON (s2pul)
Solvent: cdcl3
Data collected on: Nov 24 2016

Temp. 26.0 C / 299.1 K
Operator: vnmr1

Relax. delay 1.000 sec
Pulse 45.0 degrees
Acq. time 1.304 sec
Width 25125.6 Hz
2000 repetitions
OBSERVE C13, 100.6238513 MHz
DECOUPLE H1, 400.1760547 MHz
Power 38 dB
continuously on
WALTZ-16 modulated
DATA PROCESSING
Line broadening 0.5 Hz
FT size 65536
Total time 1 hr, 20 min

Compound 25

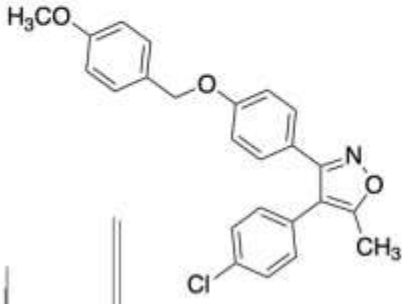

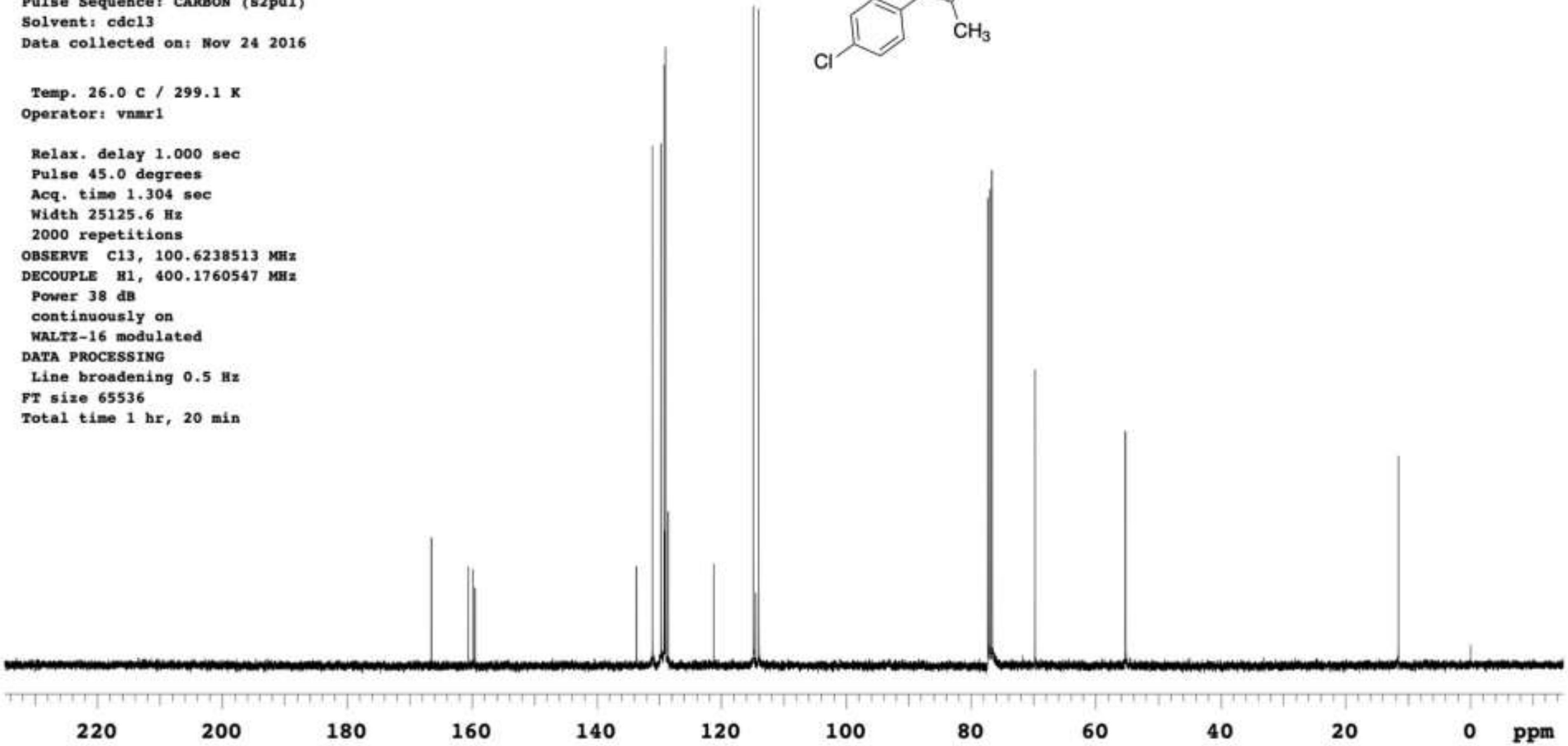

Figure S20. ^{1}H-NMR and ^{13}C-NMR spectrum of Compound **26**

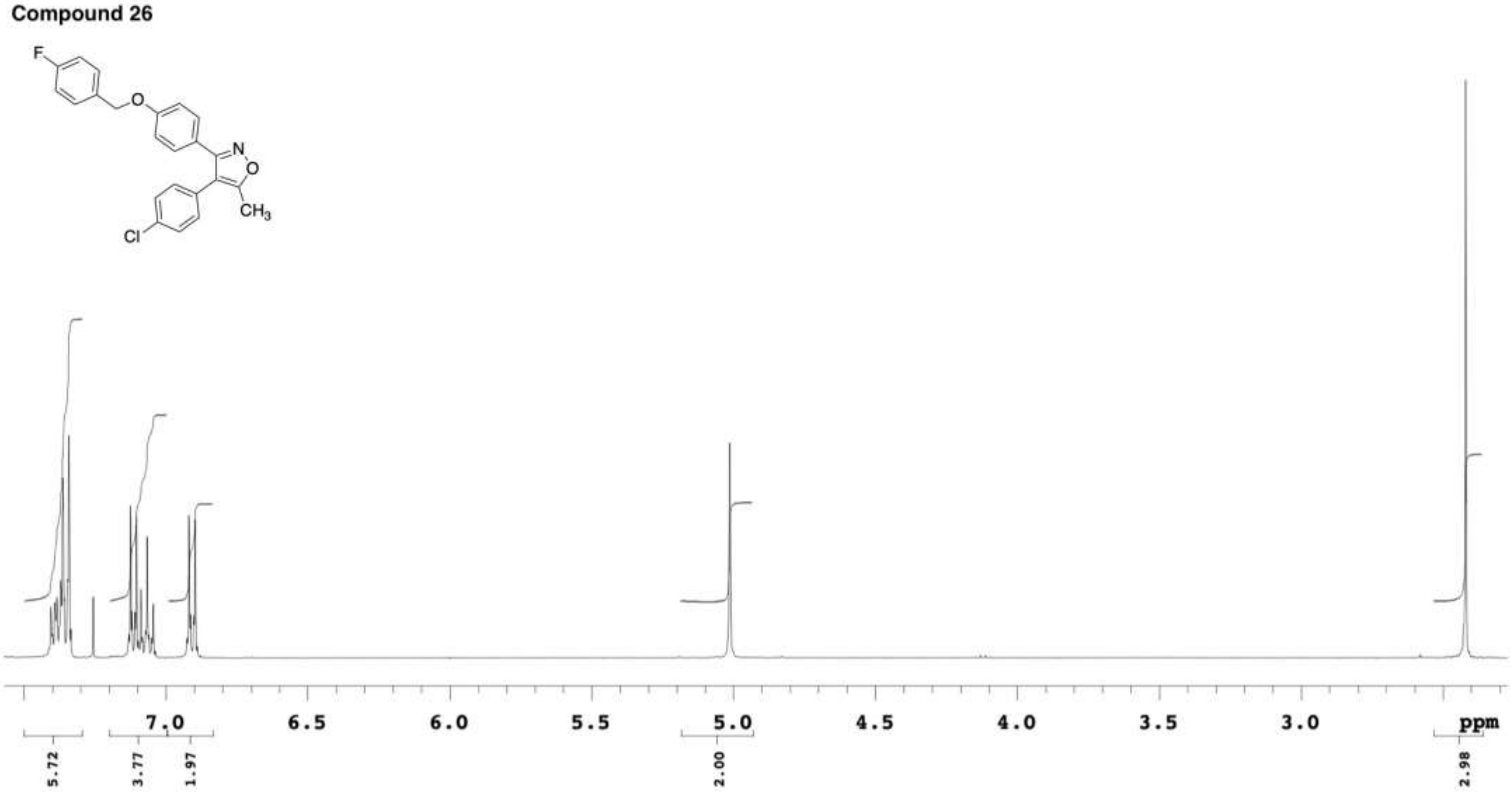

DNZ84

Sample Name:
DNZ84
Data Collected on:
mercury400-mercury400
Archive directory:
/home/vnmr1/vnmrsys/data
Sample directory:
DNZ84_20161125_01
FidFile: CARBON_01

Pulse Sequence: CARBON (s2pul)
Solvent: cdcl3
Data collected on: Nov 25 2016

Temp. 26.0 C / 299.1 K
Operator: vnmr1

Relax. delay 1.000 sec
Pulse 45.0 degrees
Acq. time 1.304 sec
Width 25125.6 Hz
2000 repetitions
OBSERVE C13, 100.6238513 MHz
DECOUPLE H1, 400.1760547 MHz
Power 38 dB
continuously on
WALTZ-16 modulated
DATA PROCESSING
Line broadening 0.5 Hz
FT size 65536
Total time 1 hr, 20 min

Compound 26

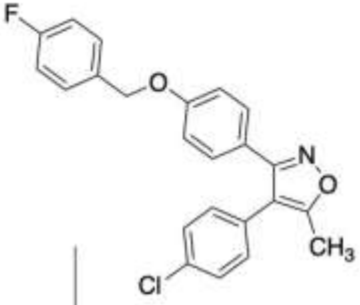

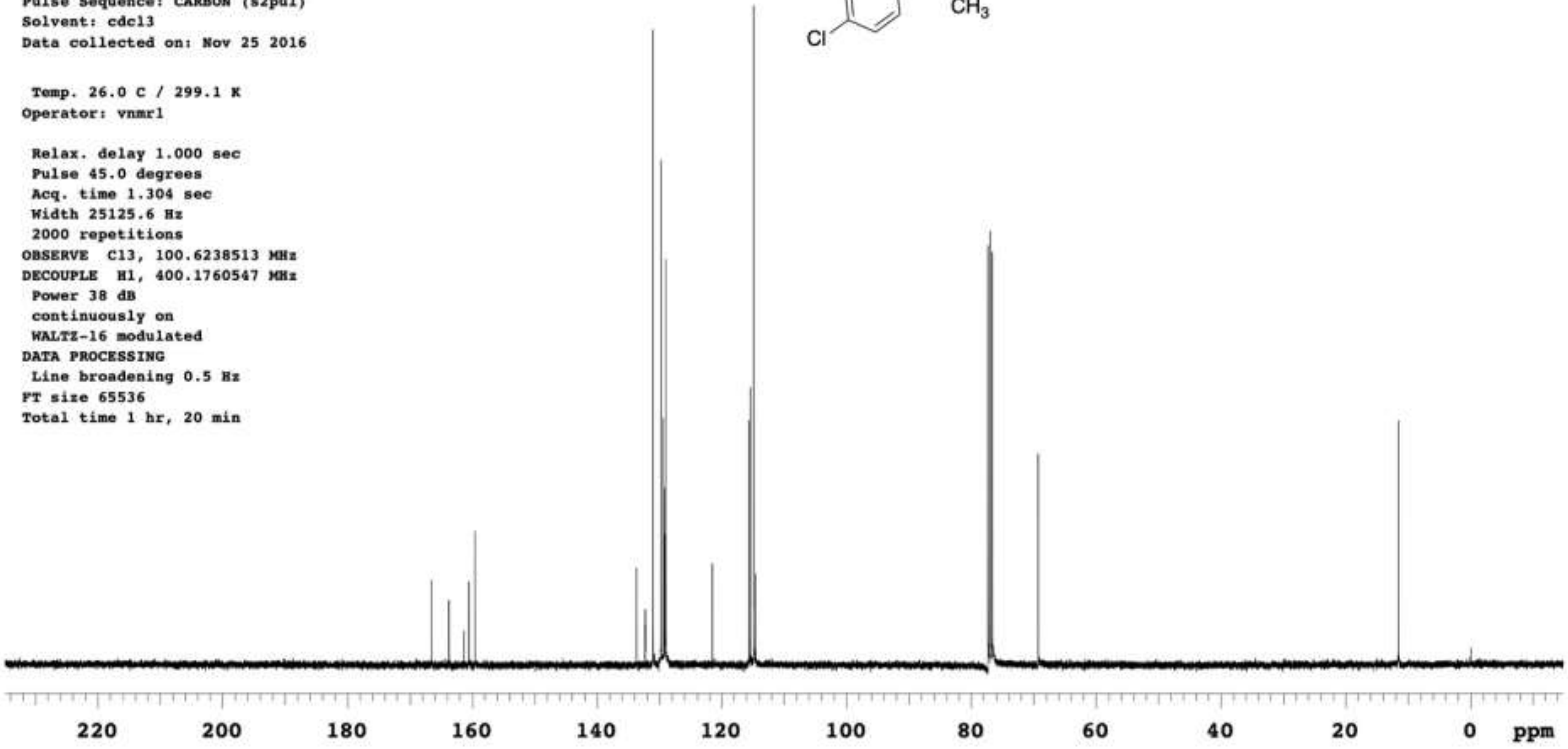

Figure S21. ^{1}H-NMR and ^{13}C-NMR spectrum of Compound **27**

Compound 27

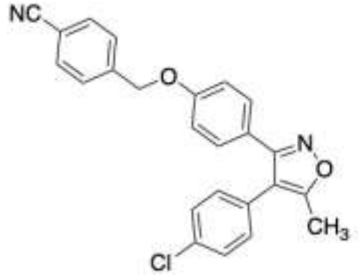

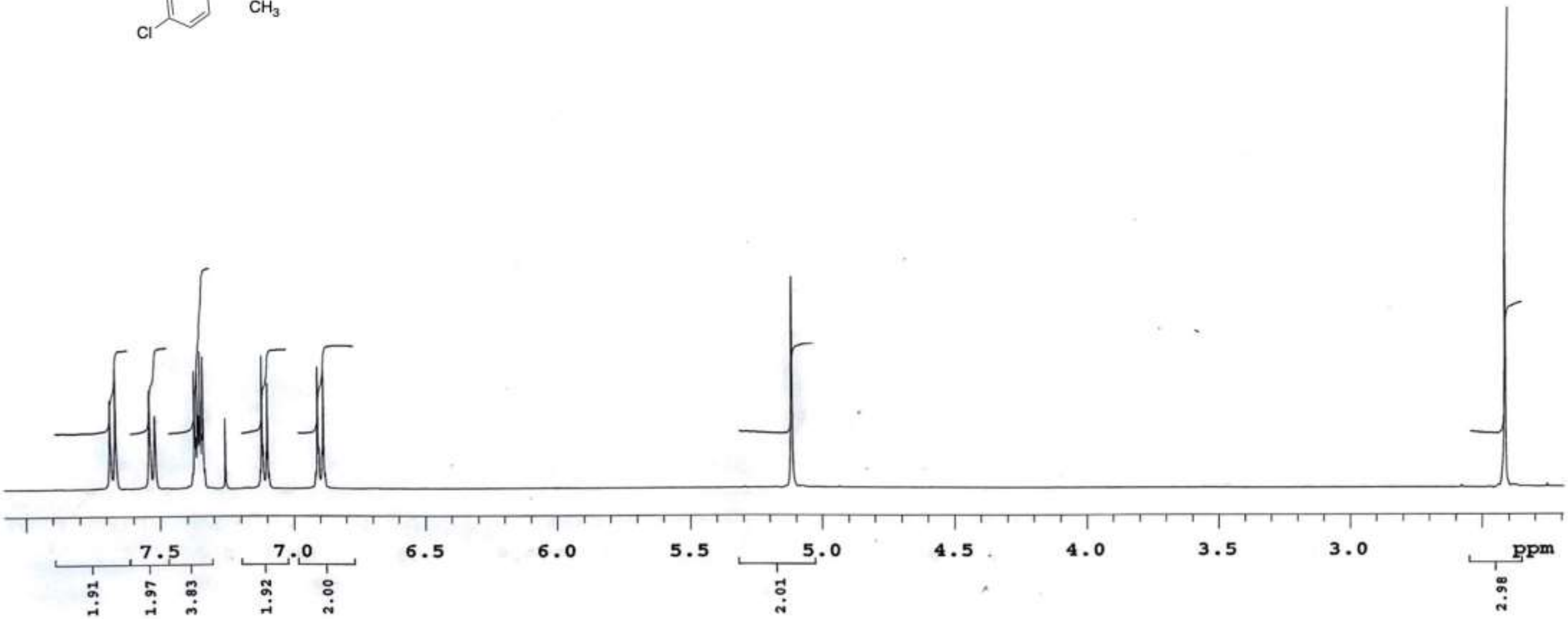

DNZ64

Sample Name:
DNZ64
Data Collected on:
mercury400-mercury400
Archive directory:
/home/vnmr1/vnmrsys/data
Sample directory:
DNZ64_20160824_01
FidFile: CARBON_01

Pulse Sequence: CARBON (s2pul)
Solvent: cdcl3
Data collected on: Aug 24 2016

Temp. 25.0 C / 298.1 K
Operator: vnmr1

Relax. delay 1.000 sec
Pulse 45.0 degrees
Acq. time 1.304 sec
Width 25125.6 Hz
1256 repetitions
OBSERVE C13, 100.6238513 MHz
DECOUPLE H1, 400.1760547 MHz
Power 38 dB
continuously on
WALTZ-16 modulated
DATA PROCESSING
Line broadening 0.5 Hz
FT size 65536
Total time 50 min

Compound 27

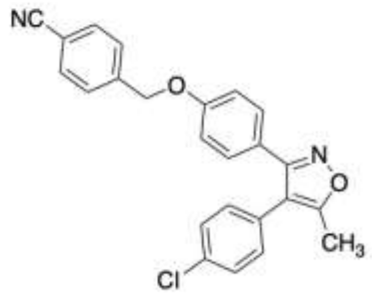

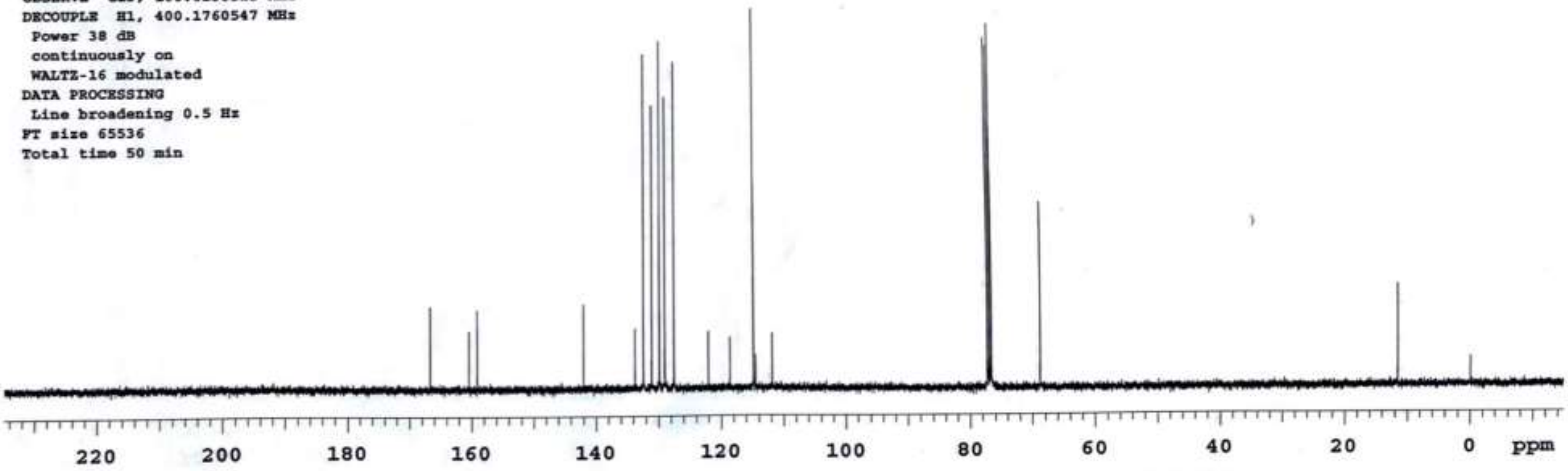

Figure S22. ^{1}H-NMR and ^{13}C-NMR spectrum of Compound **28**

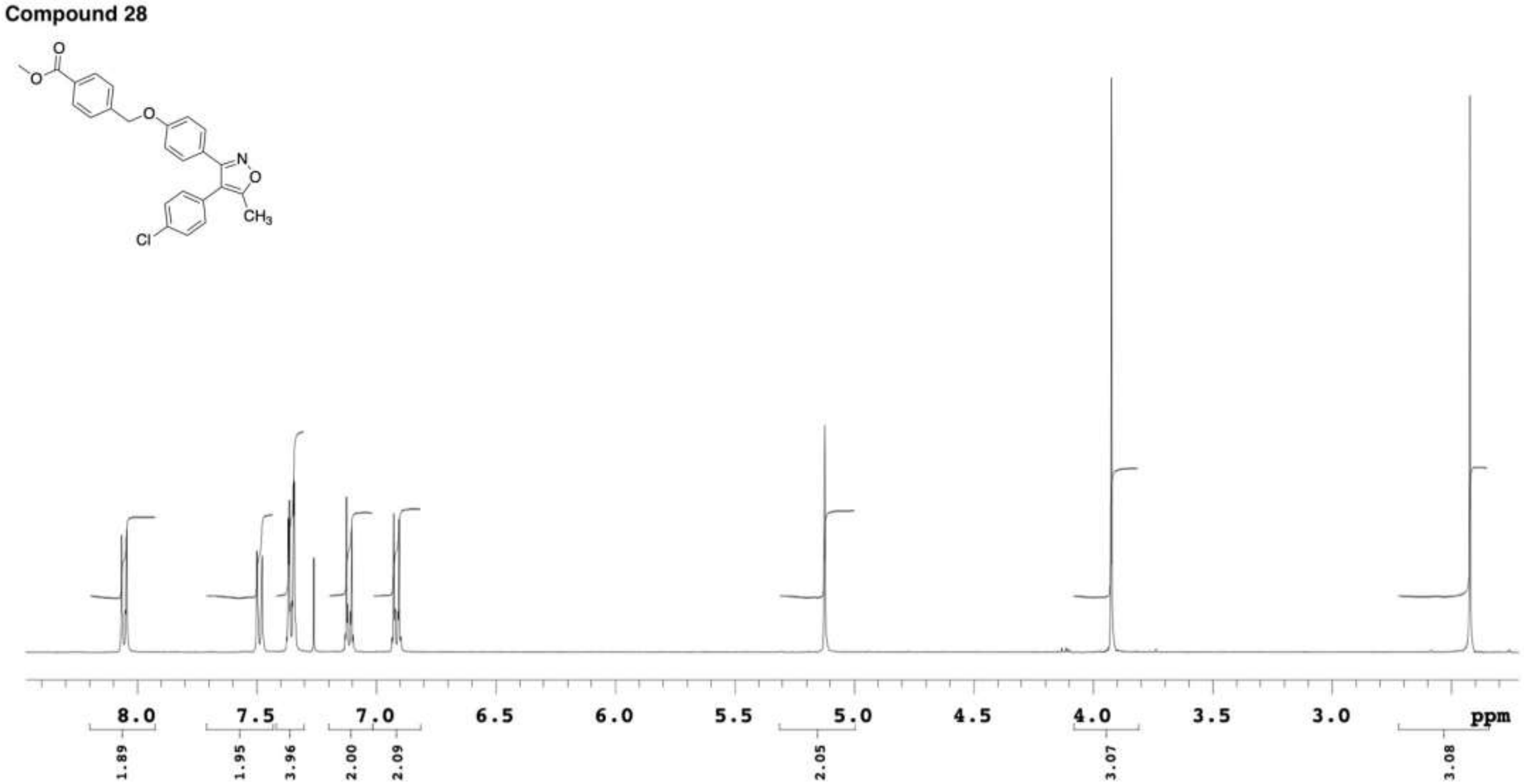

DNZ98

Sample Name:
DNZ98
Data Collected on:
mercury400-mercury400
Archive directory:
/home/vnmr1/vnmrsys/data
Sample directory:
DNZ98_20161208_01
FidFile: CARBON_01

Pulse Sequence: CARBON (s2pul)
Solvent: cdcl3
Data collected on: Dec 8 2016

Temp. 25.0 C / 298.1 K
Operator: vnmr1

Relax. delay 1.000 sec
Pulse 45.0 degrees
Acq. time 1.304 sec
Width 25125.6 Hz
1256 repetitions
OBSERVE C13, 100.6238513 MHz
DECOUPLE H1, 400.1760547 MHz
Power 38 dB
continuously on
WALTZ-16 modulated
DATA PROCESSING
Line broadening 0.5 Hz
FT size 65536
Total time 50 min

Compound 28

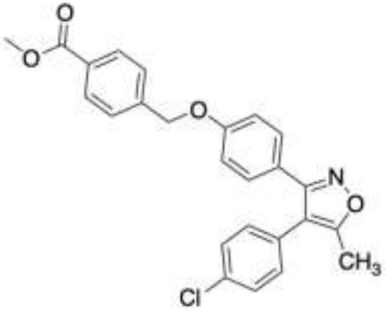

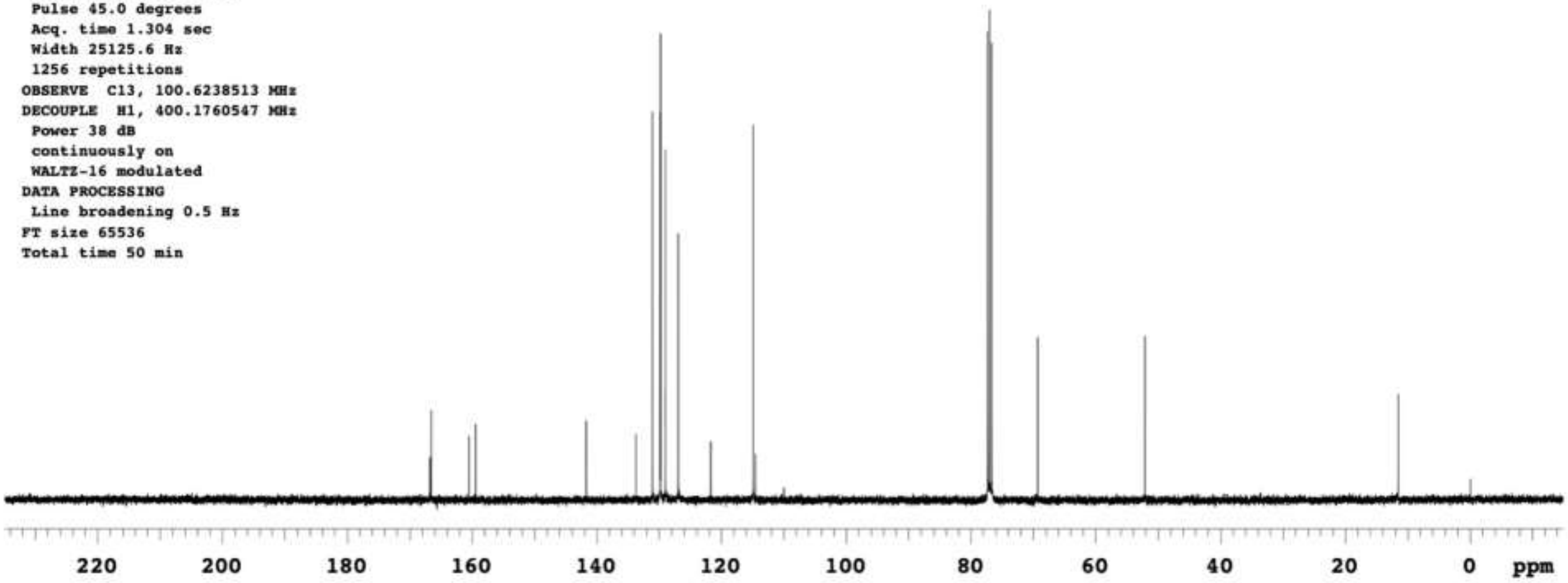

Figure S23. ^{1}H-NMR and ^{13}C-NMR spectrum of Compound **29**

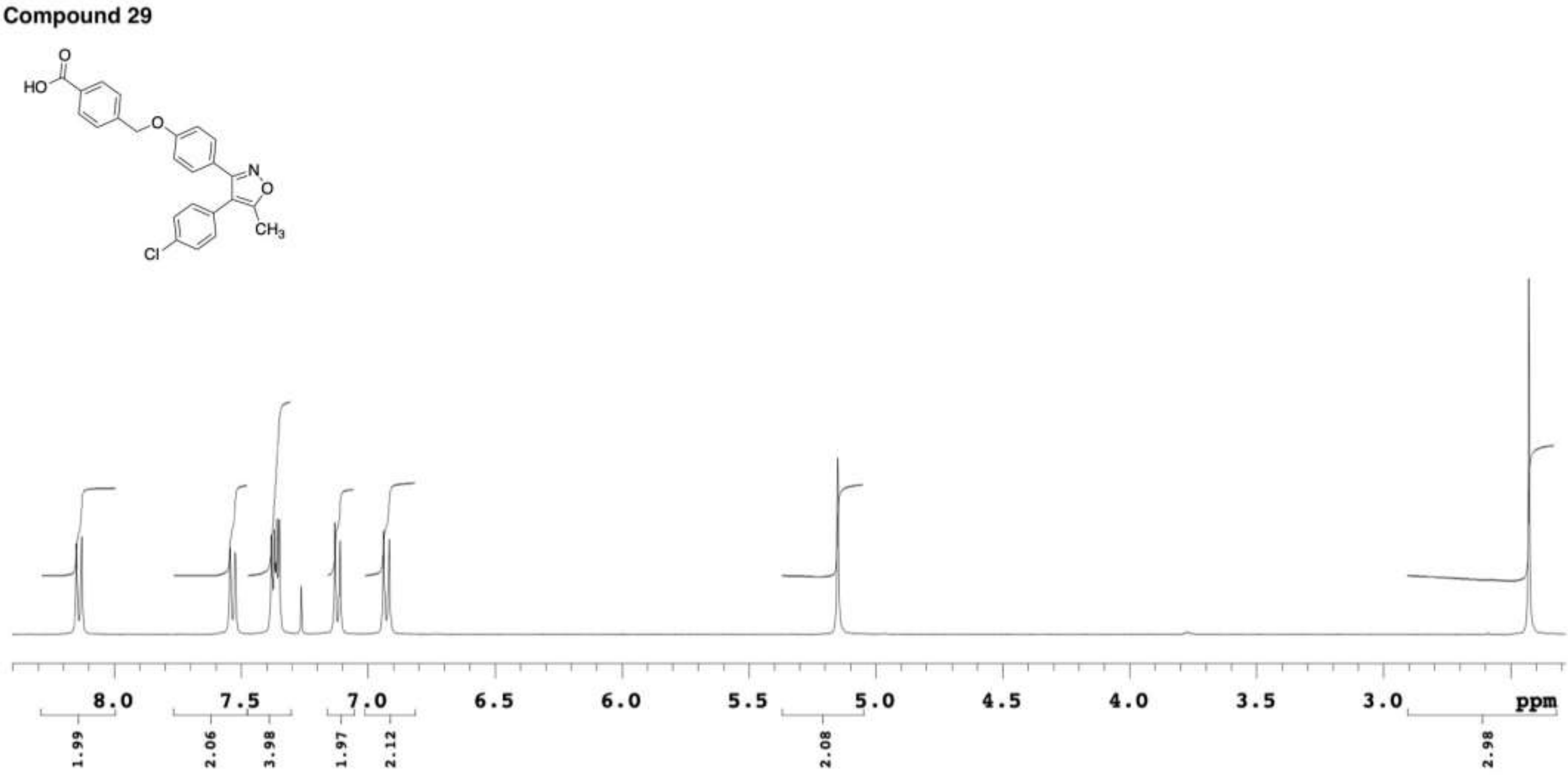

DNZ99

Sample Name:
DNZ99
Data Collected on:
mercury400-mercury400
Archive directory:
/home/vnmr1/vnmrsys/data
Sample directory:
DNZ99_20161208_01
FidFile: CARBON_01

Pulse Sequence: CARBON (s2pul)
Solvent: cdcl3
Data collected on: Dec 8 2016

Temp. 25.0 C / 298.1 K
Operator: vnmr1

Relax. delay 1.000 sec
Pulse 45.0 degrees
Acq. time 1.304 sec
Width 25125.6 Hz
2000 repetitions
OBSERVE C13, 100.6238513 MHz
DECOUPLE H1, 400.1760547 MHz
Power 38 dB
continuously on
WALTZ-16 modulated
DATA PROCESSING
Line broadening 0.5 Hz
FT size 65536
Total time 1 hr, 20 min

Compound 29

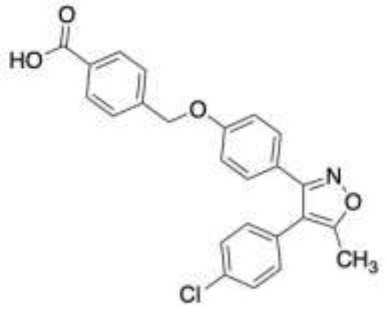

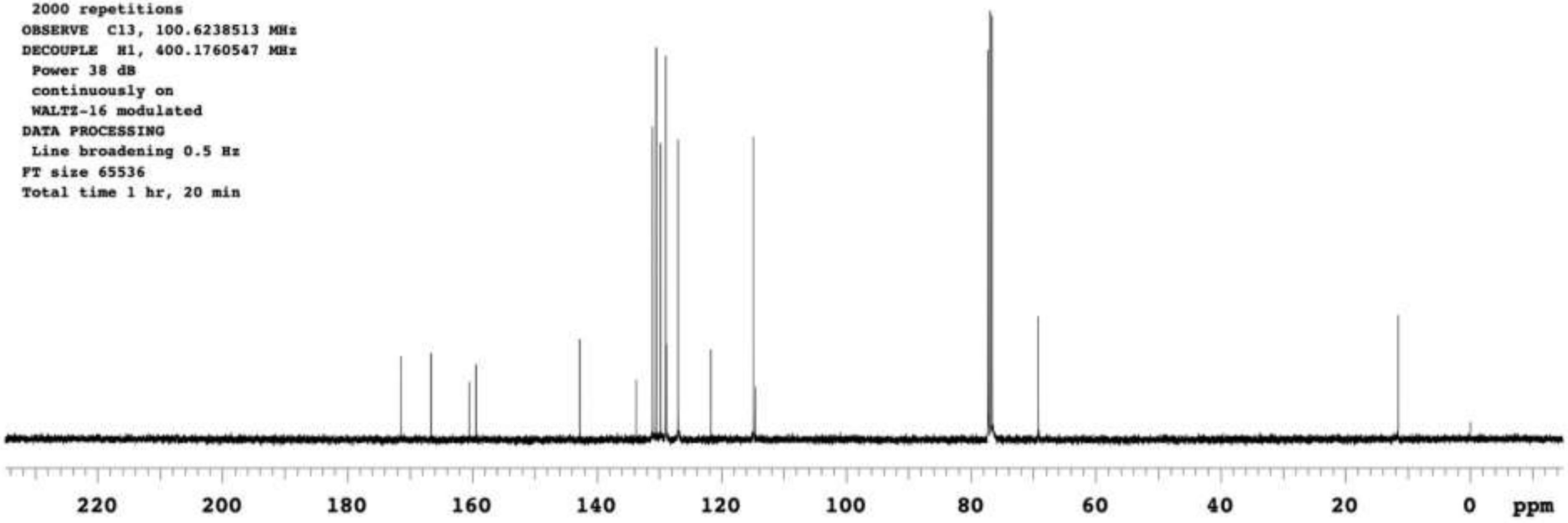

Figure S24. ^{1}H-NMR and ^{13}C-NMR spectrum of Compound **30**

Compound 30

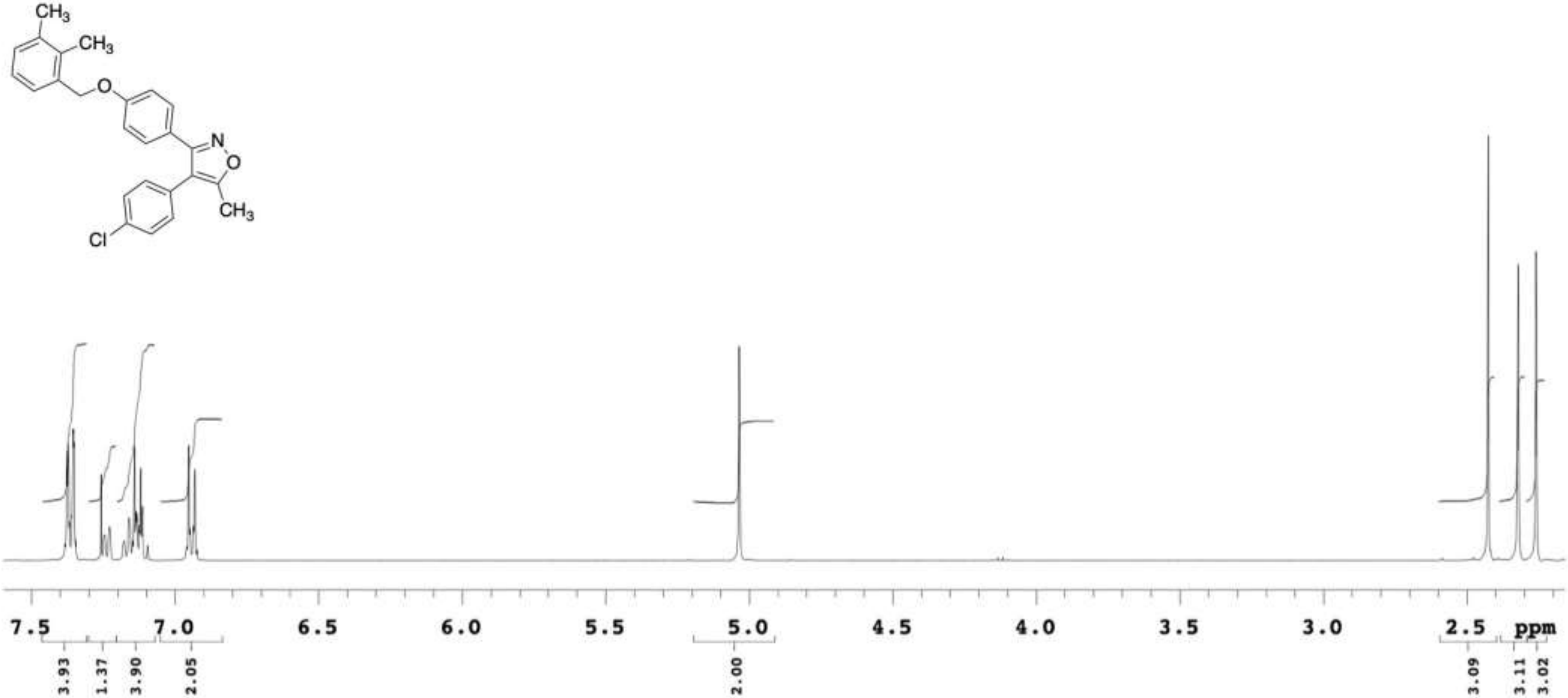

DNZ134

Sample Name:
DNZ134
Data Collected on:
mercury400-mercury400
Archive directory:
/home/vnmr1/vnmrsys/data
Sample directory:
DNZ134_20170401_01
FidFile: current

Pulse Sequence: CARBON (s2pul)
Solvent: cdcl3
Data collected on: Apr 1 2017

Temp. 25.0 C / 298.1 K
Operator: vnmr1

Relax. delay 1.000 sec
Pulse 45.0 degrees
Acq. time 1.550 sec
Width 21141.6 Hz
320 repetitions
OBSERVE C13, 100.6238513 MHz
DECOUPLE H1, 400.1760547 MHz
Power 38 dB
continuously on
WALTZ-16 modulated
DATA PROCESSING
Line broadening 0.5 Hz
FT size 65536
Total time 1 hr, 6 min

Compound 30

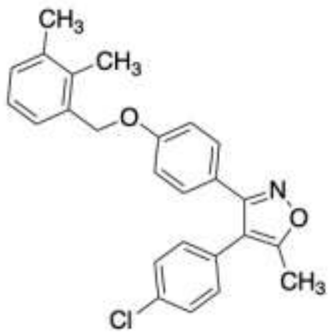

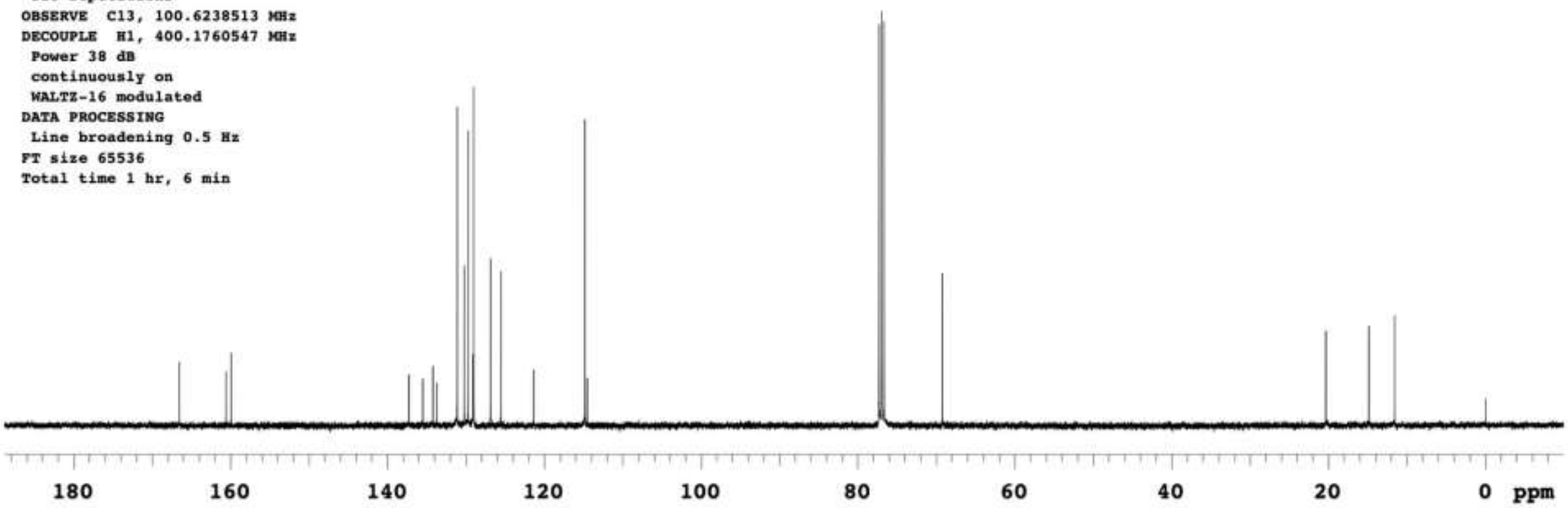

Figure S25. ^{1}H-NMR and ^{13}C-NMR spectrum of Compound **31**

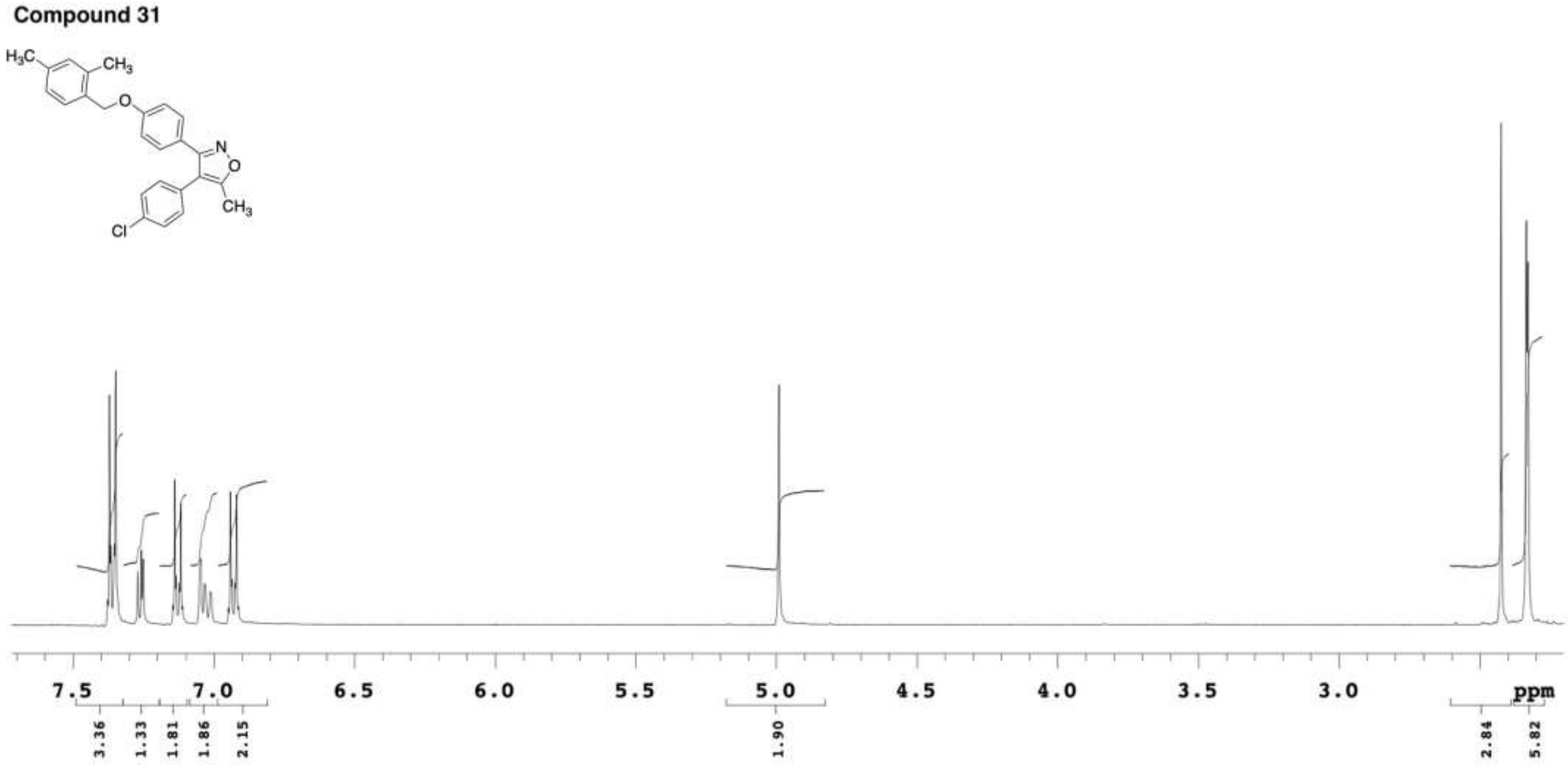

DNZ136

Sample Name:
DNZ136
Data Collected on:
mercury400-mercury400
Archive directory:
/home/vnmr1/vnmrsys/data
Sample directory:
DNZ136_20170405_01
FidFile: CARBON_01

Pulse Sequence: CARBON (s2pul)
Solvent: cdcl3
Data collected on: Apr 5 2017

Temp. 25.0 C / 298.1 K
Operator: vnmr1

Relax. delay 1.000 sec
Pulse 45.0 degrees
Acq. time 1.550 sec
Width 21141.6 Hz
1000 repetitions
OBSERVE C13, 100.6238513 MHz
DECOUPLE H1, 400.1760547 MHz
Power 38 dB
continuously on
WALTZ-16 modulated
DATA PROCESSING
Line broadening 0.5 Hz
FT size 65536
Total time 44 min

Compound 31

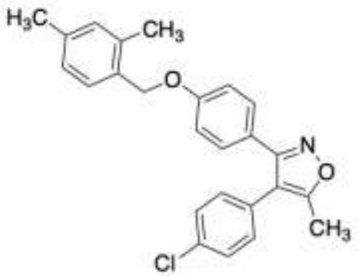

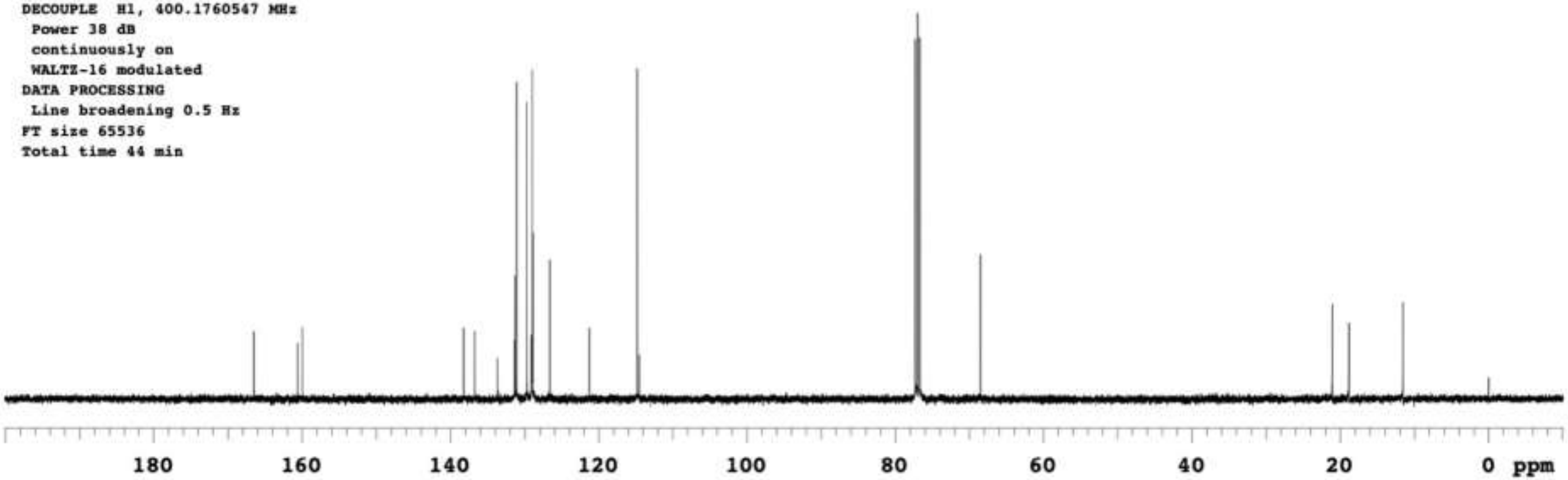

Figure S26. ^{1}H-NMR and ^{13}C-NMR spectrum of Compound **32**

Compound 32

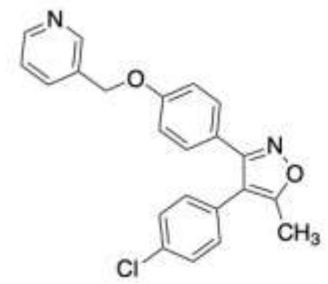

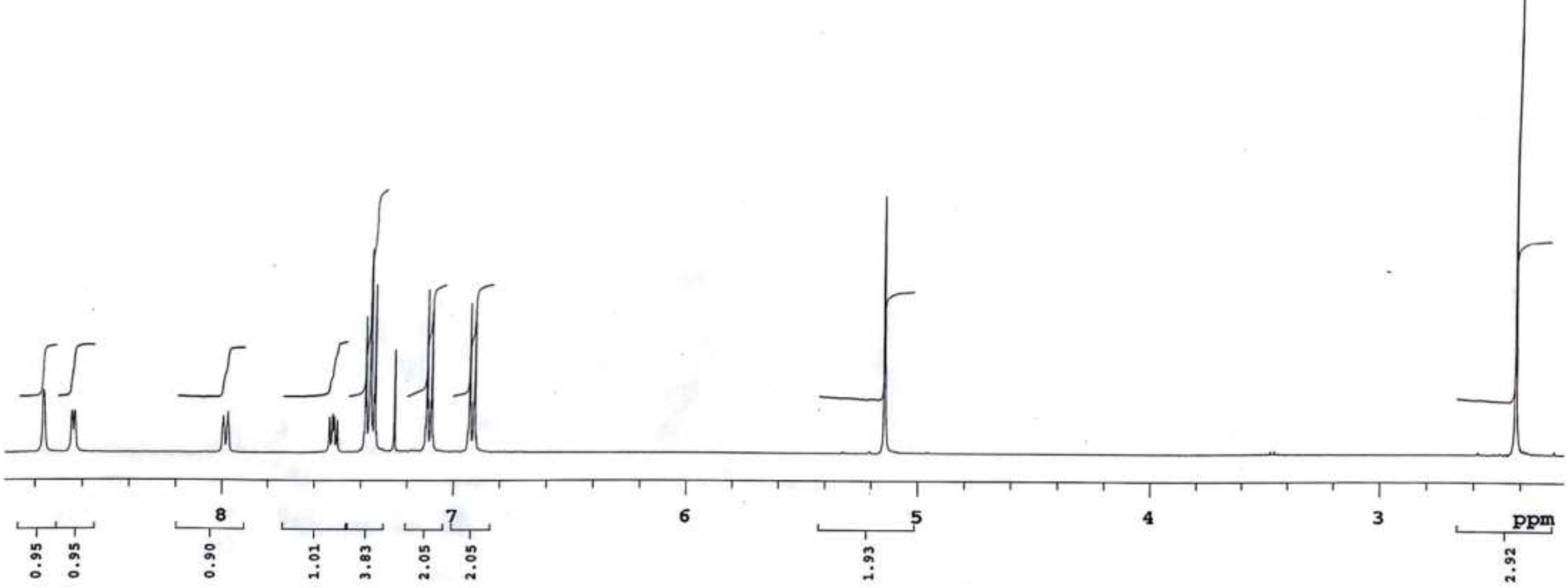

DNZ62

Sample Name:
DNZ62
Data Collected on:
mercury400-mercury400
Archive directory:
/home/vnmr1/vnmrsys/data
Sample directory:
DNZ62_20160824_01
FidFile: current

Pulse Sequence: CARBON (s2pul)
Solvent: cdcl3
Data collected on: Aug 24 2016

Temp. 25.0 C / 298.1 K
Operator: vnmr1

Relax. delay 1.000 sec
Pulse 45.0 degrees
Acq. time 1.550 sec
Width 21141.6 Hz
64 repetitions
OBSERVE C13, 100.6238513 MHz
DECOUPLE H1, 400.1760547 MHz
Power 38 dB
continuously on
WALTZ-16 modulated
DATA PROCESSING
Line broadening 0.5 Hz
FT size 65536
Total time 55 min

Compound 32

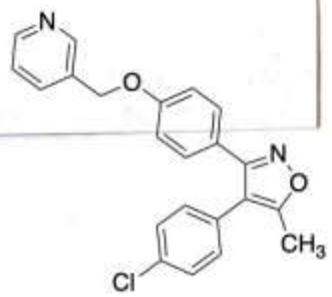

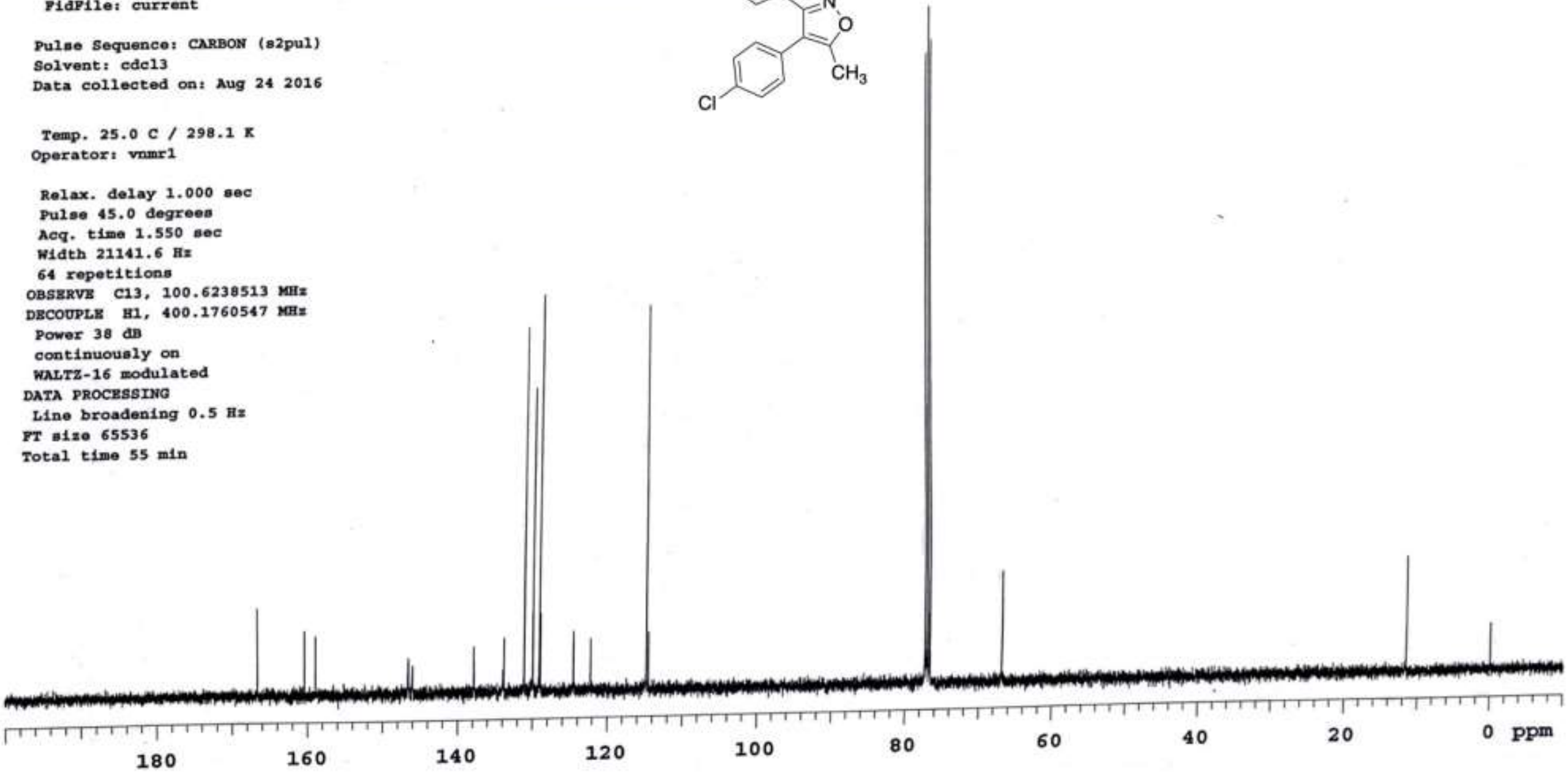

Figure S27. ^{1}H-NMR and ^{13}C-NMR spectrum of Compound **33**

Compound 33

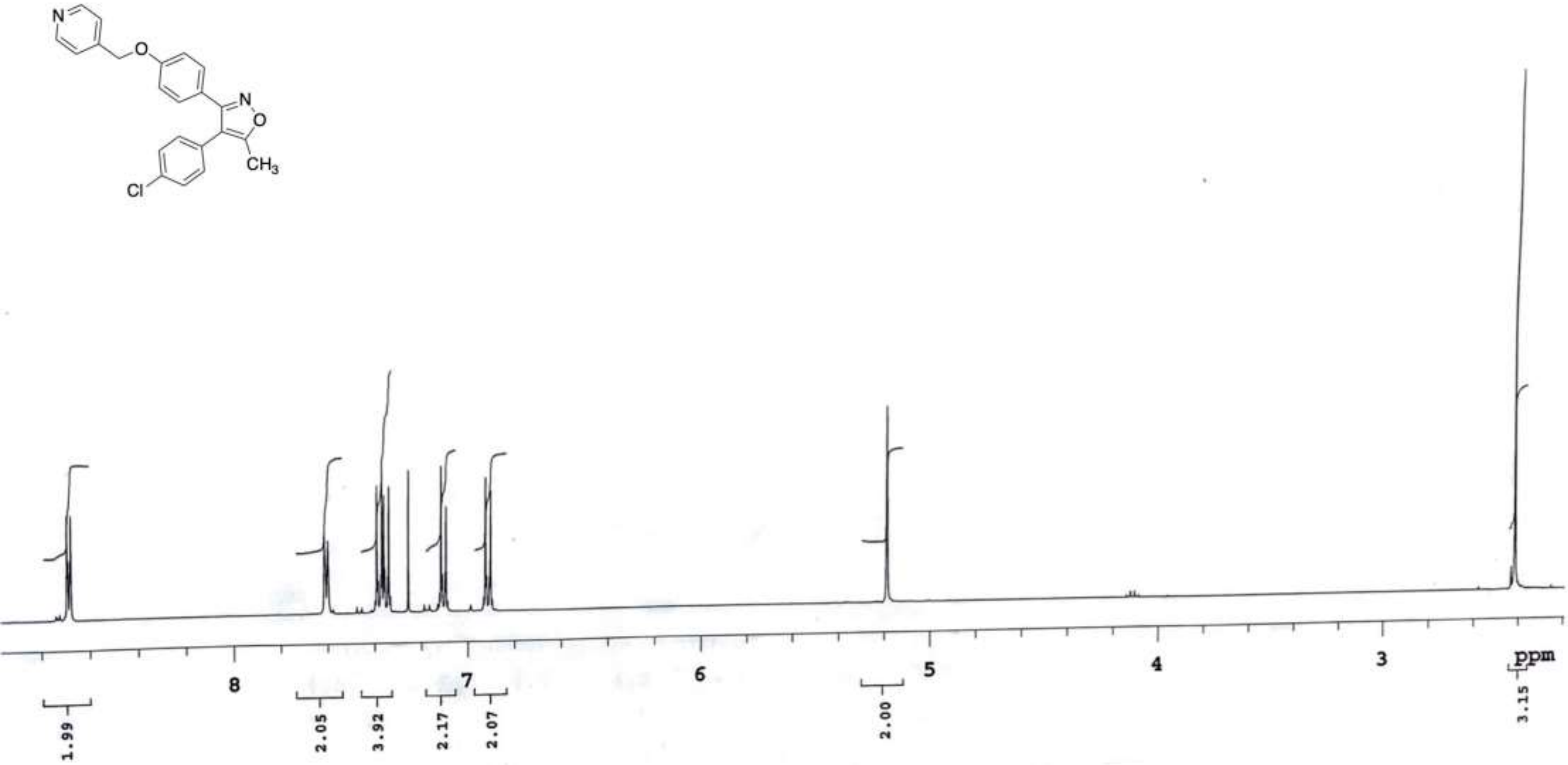

DNZ56

Sample Name:
DNZ56
Data Collected on:
mercury400-mercury400
Archive directory:
/home/vnmr1/vnmrsys/data
Sample directory:
DNZ56_20160825_01
FidFile: current

Pulse Sequence: CARBON (s2pul)
Solvent: cdcl3
Data collected on: Aug 25 2016

Temp. 35.0 C / 308.1 K
Operator: vnmr1

Relax. delay 1.000 sec
Pulse 45.0 degrees
Acq. time 1.550 sec
Width 21141.6 Hz
1512 repetitions
OBSERVE C13, 100.6238513 MHz
DECOUPLE H1, 400.1760547 MHz
Power 38 dB
continuously on
WALTZ-16 modulated
DATA PROCESSING
Line broadening 0.5 Hz
FT size 65536
Total time 1 hr, 6 min

Compound 33

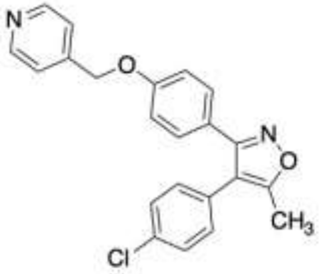

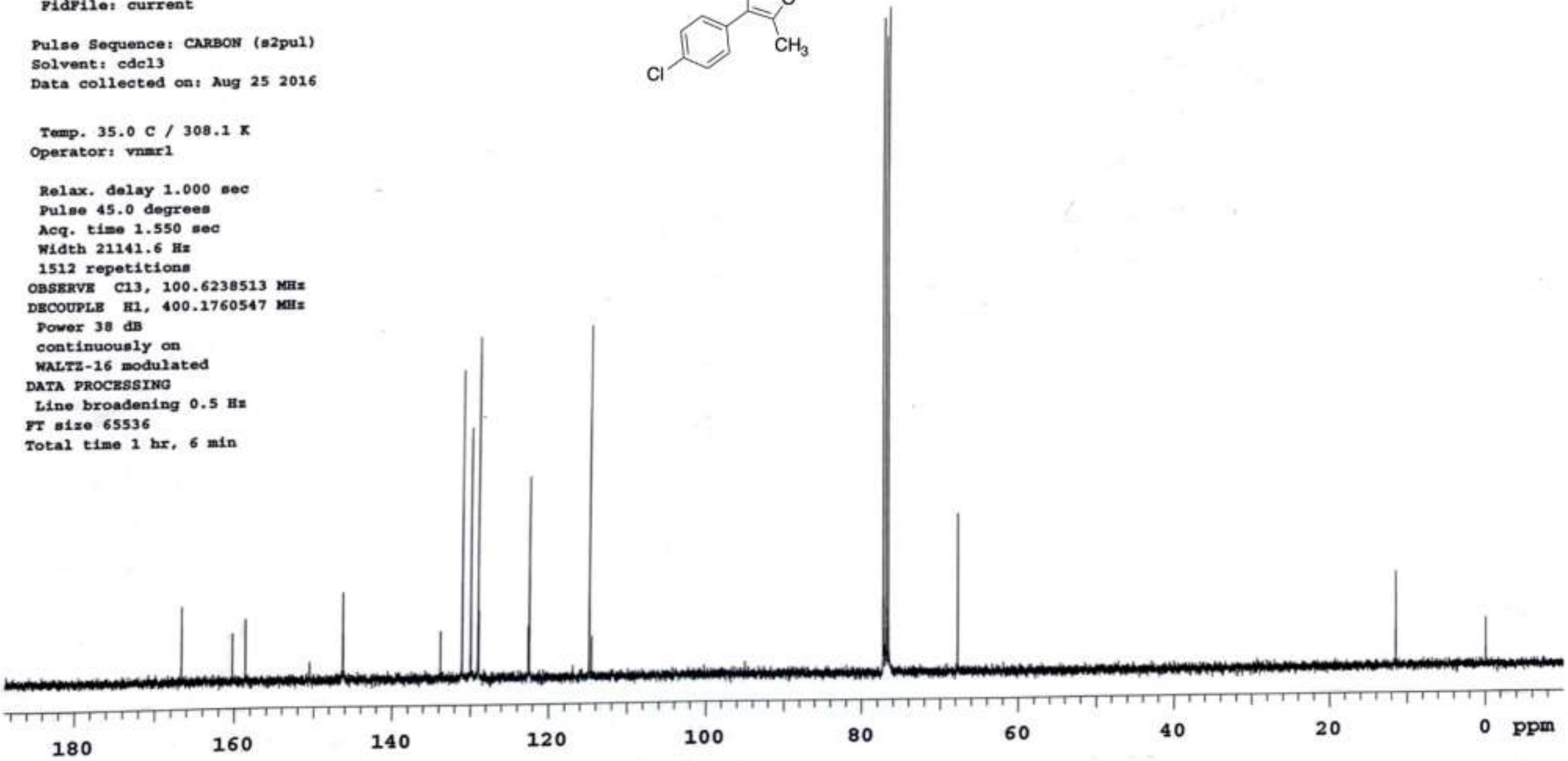

Figure S28. ^{1}H-NMR and ^{13}C-NMR spectrum of Compound **34**

Compound 34

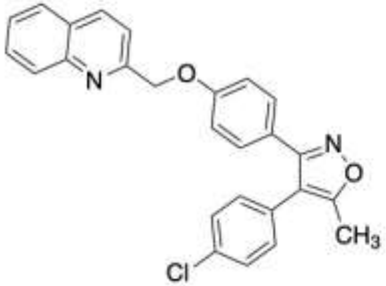

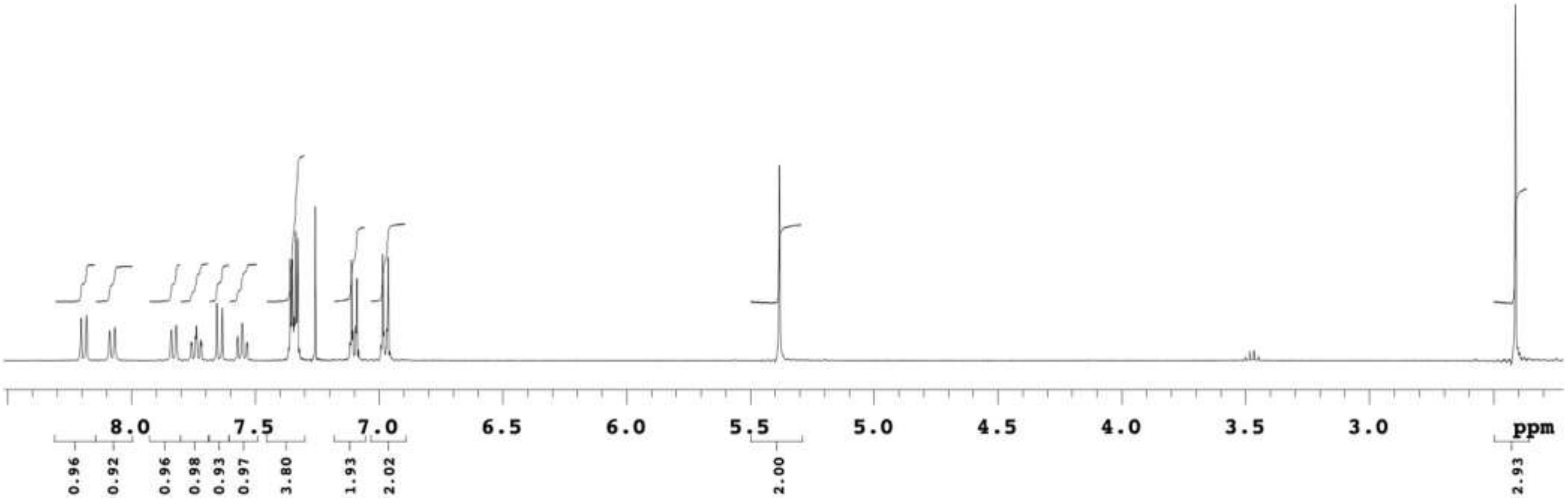

DNZ58

Sample Name:
DNZ58
Data Collected on:
mercury400-mercury400
Archive directory:
/home/vnmr1/vnmrsys/data
Sample directory:
DNZ58_20160924_01
FidFile: CARBON_01

Pulse Sequence: CARBON (s2pul)
Solvent: cdcl3
Data collected on: Sep 24 2016

Temp. 37.0 C / 310.1 K
Operator: vnmr1

Relax. delay 1.000 sec
Pulse 45.0 degrees
Acq. time 1.304 sec
Width 25125.6 Hz
3512 repetitions
OBSERVE C13, 100.6238513 MHz
DECOUPLE H1, 400.1760547 MHz
Power 38 dB
continuously on
WALTZ-16 modulated
DATA PROCESSING
Line broadening 0.5 Hz
FT size 65536
Total time 2 hr, 20 min

Compound 34

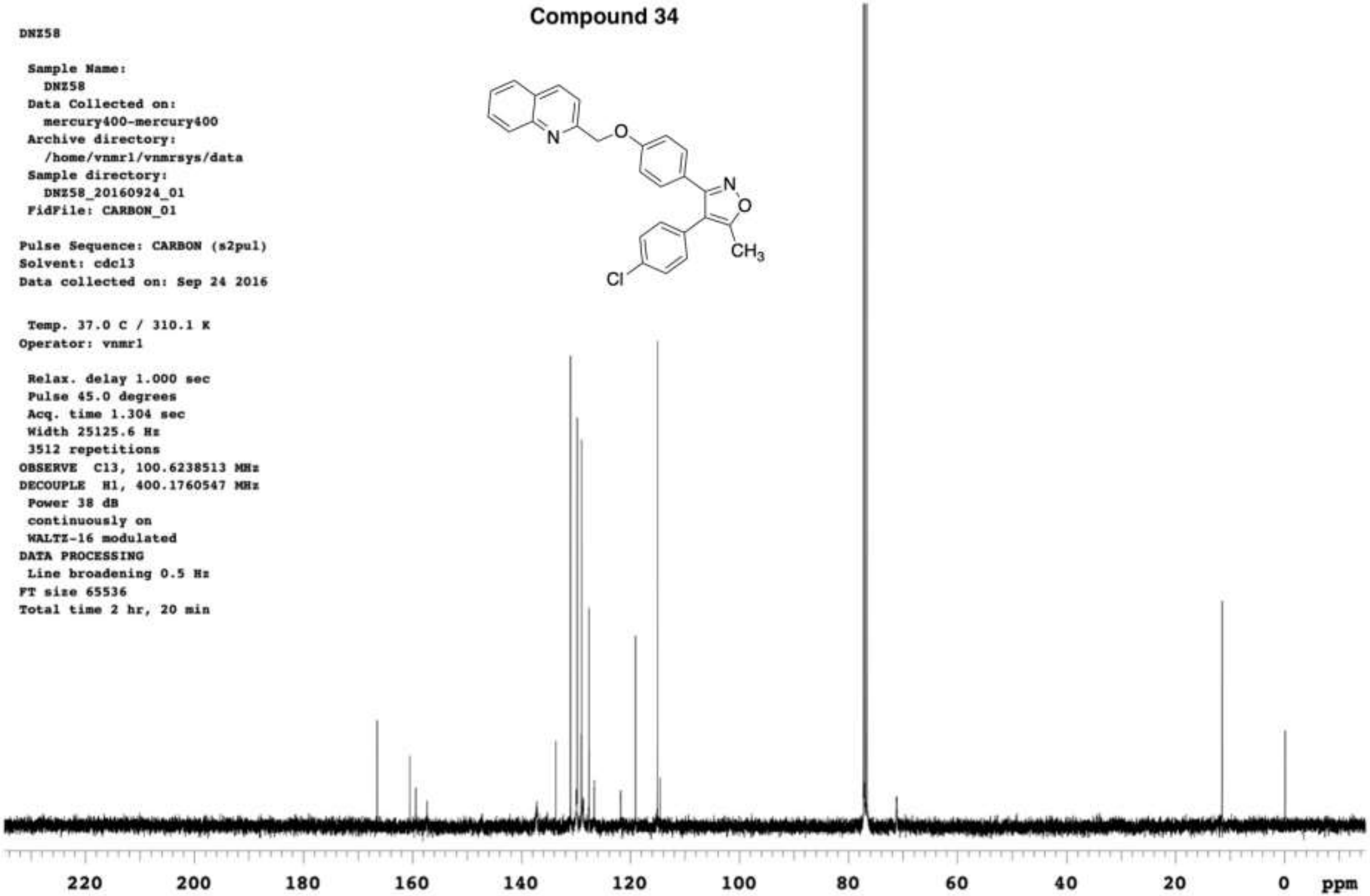

Figure S29. ^{1}H-NMR and ^{13}C-NMR spectrum of Compound **35**

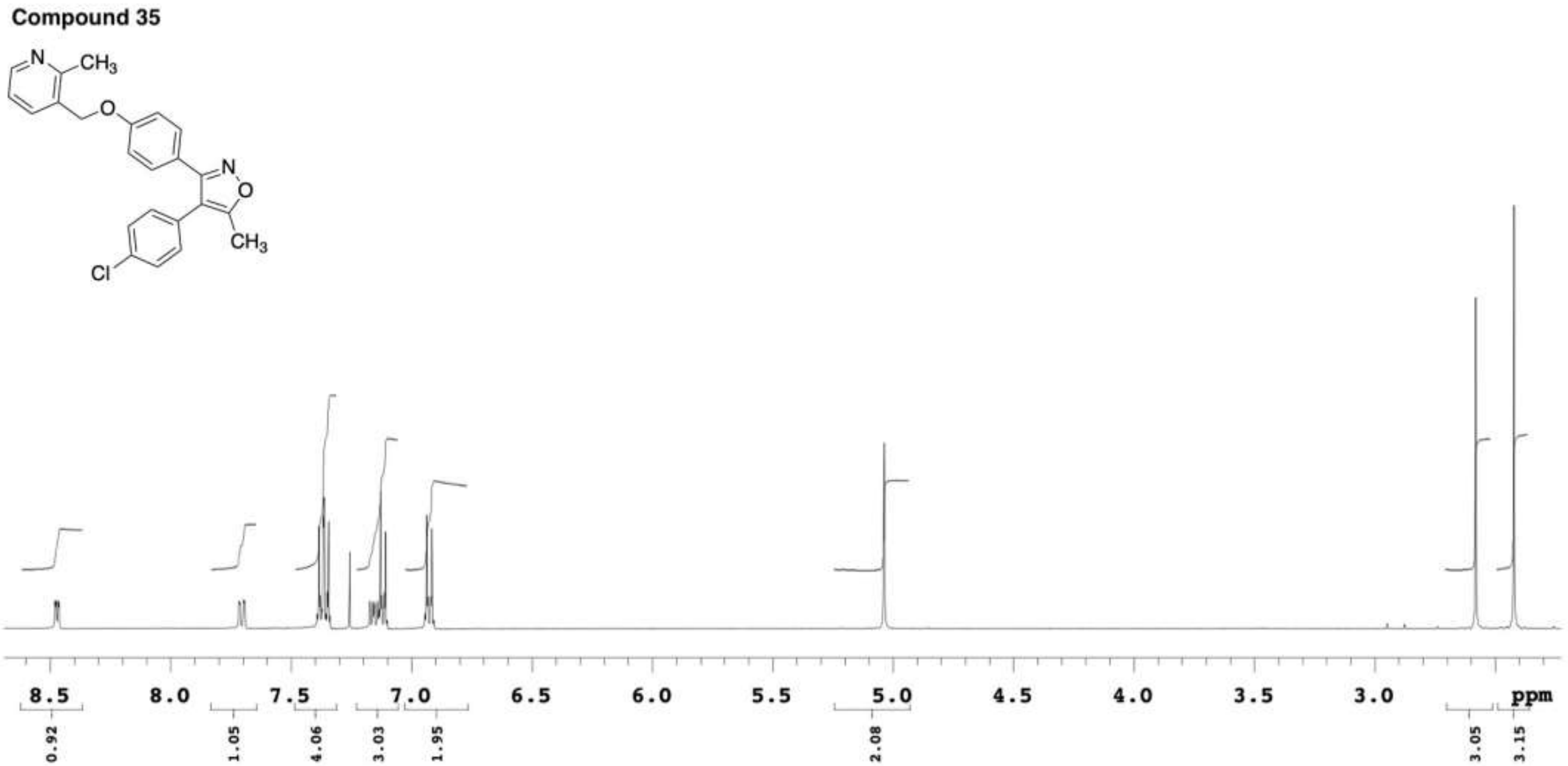

DNZ135

Sample Name:
DNZ135
Data Collected on:
mercury400-mercury400
Archive directory:
/home/vnmr1/vnmrsys/data
Sample directory:
DNZ135_20170401_01
FidFile: current

Pulse Sequence: CARBON (s2pul)
Solvent: cdcl3
Data collected on: Apr 1 2017

Temp. 25.0 C / 298.1 K
Operator: vnmr1

Relax. delay 1.000 sec
Pulse 45.0 degrees
Acq. time 1.550 sec
Width 21141.6 Hz
1088 repetitions
OBSERVE C13, 100.6238513 MHz
DECOUPLE H1, 400.1760547 MHz
Power 38 dB
continuously on
WALTZ-16 modulated
DATA PROCESSING
Line broadening 0.5 Hz
FT size 65536
Total time 55 min

Compound 35

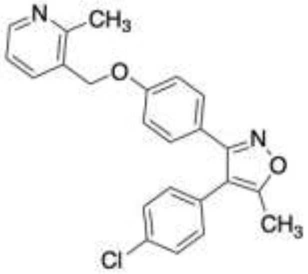

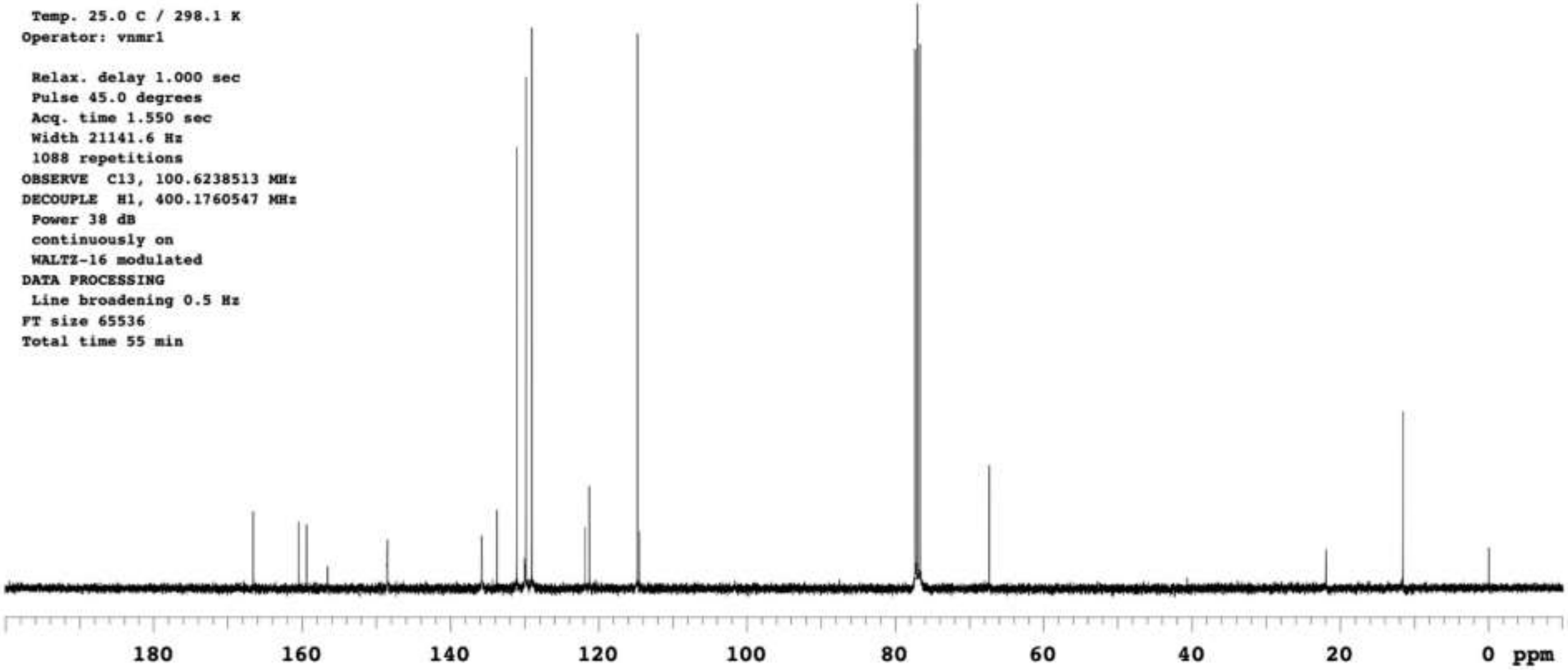

Figure S30. ^{1}H-NMR and ^{13}C-NMR spectrum of Compound **36**

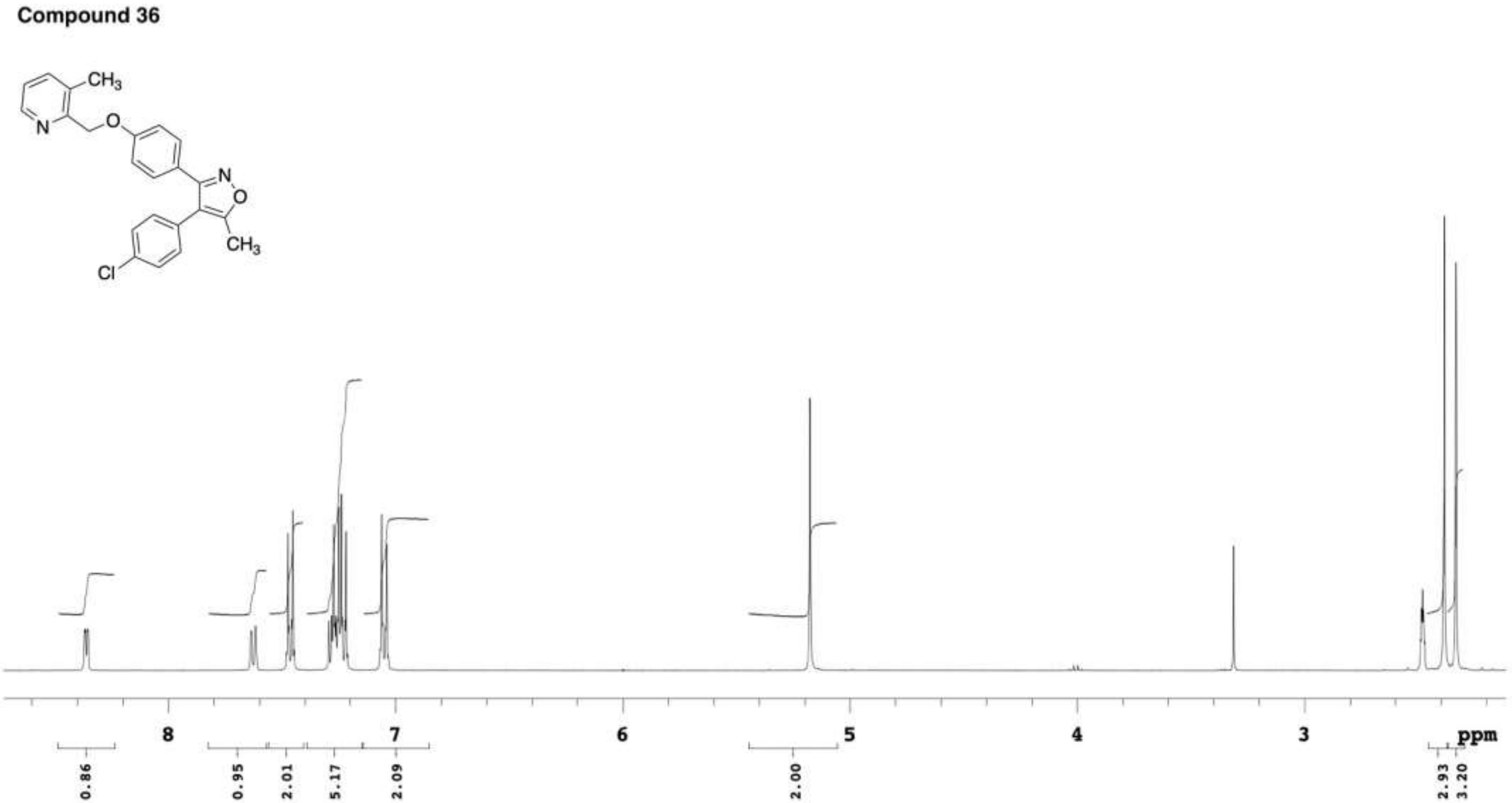

DNZ137

Sample Name:
DNZ137
Data Collected on:
mercury400-mercury400
Archive directory:
/home/vnmr1/vnmrsys/data
Sample directory:
DNZ137_20170406_01
FidFile: current

Pulse Sequence: CARBON (s2pul)
Solvent: dmso
Data collected on: Apr 6 2017

Temp. 25.0 C / 298.1 K
Operator: vnmr1

Relax. delay 1.000 sec
Pulse 45.0 degrees
Acq. time 1.550 sec
Width 21141.6 Hz
768 repetitions
OBSERVE C13, 100.6243781 MHz
DECOUPLE H1, 400.1779555 MHz
Power 38 dB
continuously on
WALTZ-16 modulated
DATA PROCESSING
Line broadening 0.5 Hz
FT size 65536
Total time 1 hr, 28 min

Compound 36

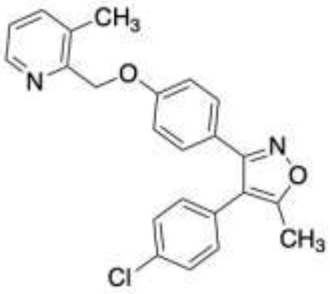

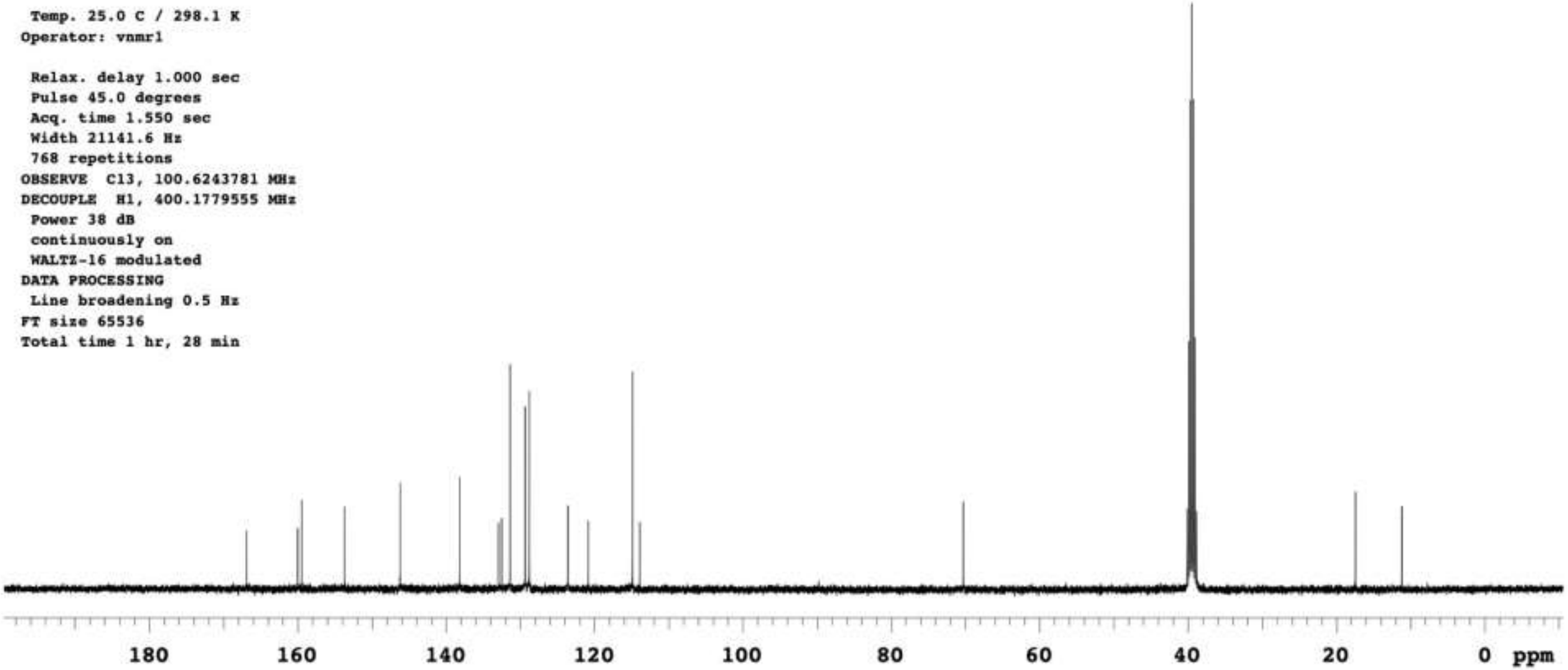

Figure S31. ^{1}H-NMR and ^{13}C-NMR spectrum of Compound **37**

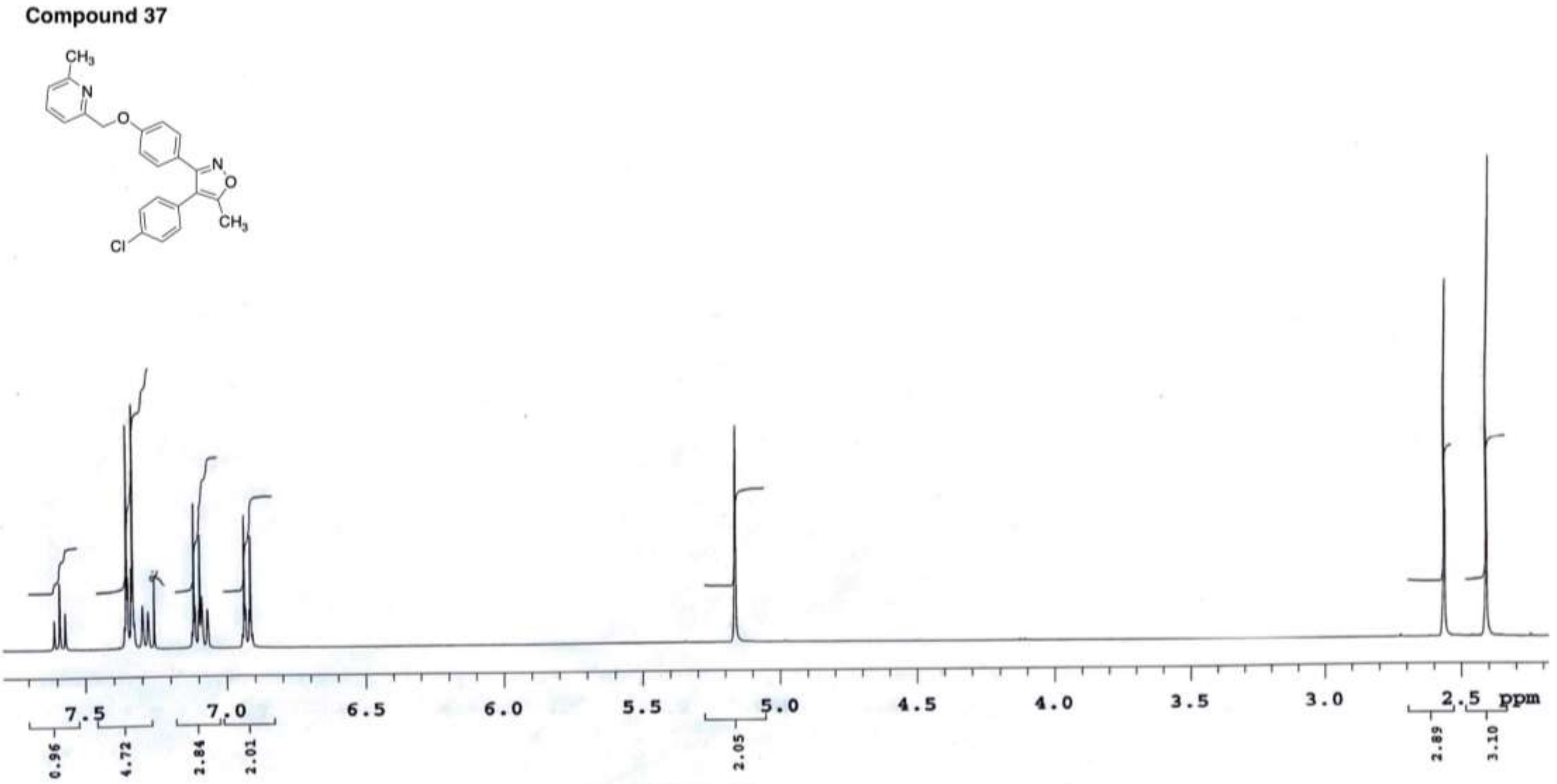

DNZ52

Sample Name:
DNZ52
Data Collected on:
mercury400-mercury400
Archive directory:
/home/vnmr1/vnmrsys/data
Sample directory:
DNZ52_20160825_01
FidFile: CARBON_01

Pulse Sequence: CARBON (s2pul)
Solvent: cdcl3
Data collected on: Aug 25 2016

Temp. 35.0 C / 308.1 K
Operator: vnmr1

Relax. delay 1.000 sec
Pulse 45.0 degrees
Acq. time 1.550 sec
Width 21141.6 Hz
1256 repetitions
OBSERVE C13, 100.6238513 MHz
DECOUPLE H1, 400.1760547 MHz
Power 38 dB
continuously on
WALTZ-16 modulated
DATA PROCESSING
Line broadening 0.5 Hz
FT size 65536
Total time 55 min

Compound 37

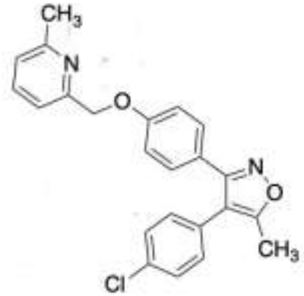

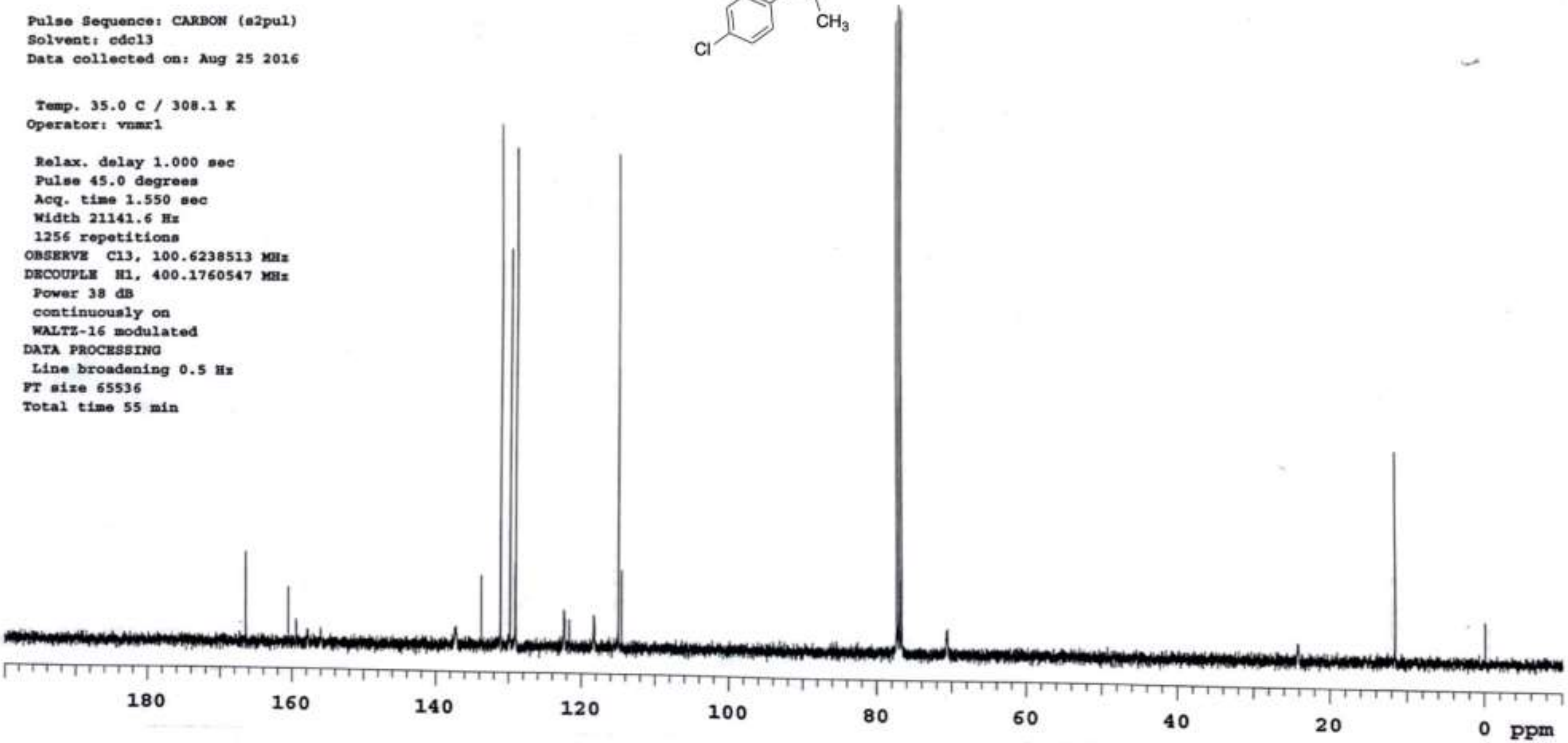

Figure S32. ^{1}H-NMR and ^{13}C-NMR spectrum of Compound **38**

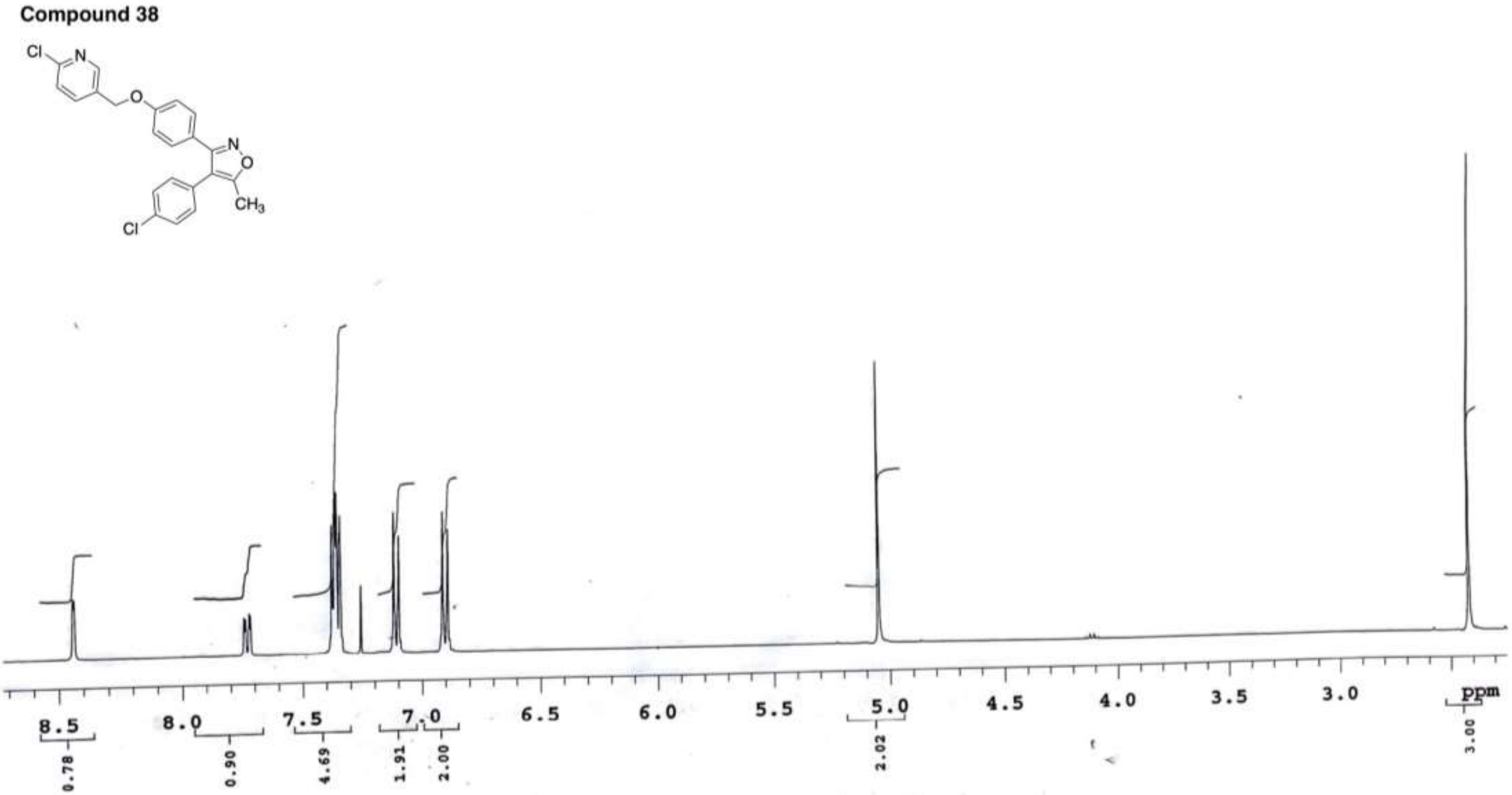

DNZ66

Sample Name:
DNZ66
Data Collected on:
mercury400-mercury400
Archive directory:
/home/vnmr1/vnmrsys/data
Sample directory:
DNZ66_20160825_01
FidFile: CARBON_01

Pulse Sequence: CARBON (s2pul)
Solvent: cdcl3
Data collected on: Aug 25 2016

Temp. 32.0 C / 305.1 K
Operator: vnmr1

Relax. delay 1.000 sec
Pulse 45.0 degrees
Acq. time 1.550 sec
Width 21141.6 Hz
1256 repetitions
OBSERVE C13, 100.6238513 MHz
DECOUPLE H1, 400.1760547 MHz
Power 38 dB
continuously on
WALTZ-16 modulated
DATA PROCESSING
Line broadening 0.5 Hz
FT size 65536
Total time 55 min

Compound 38

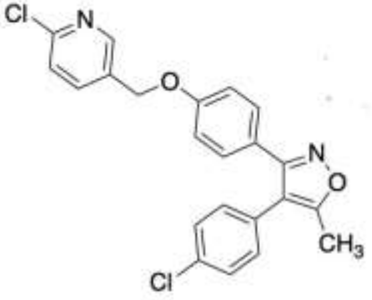

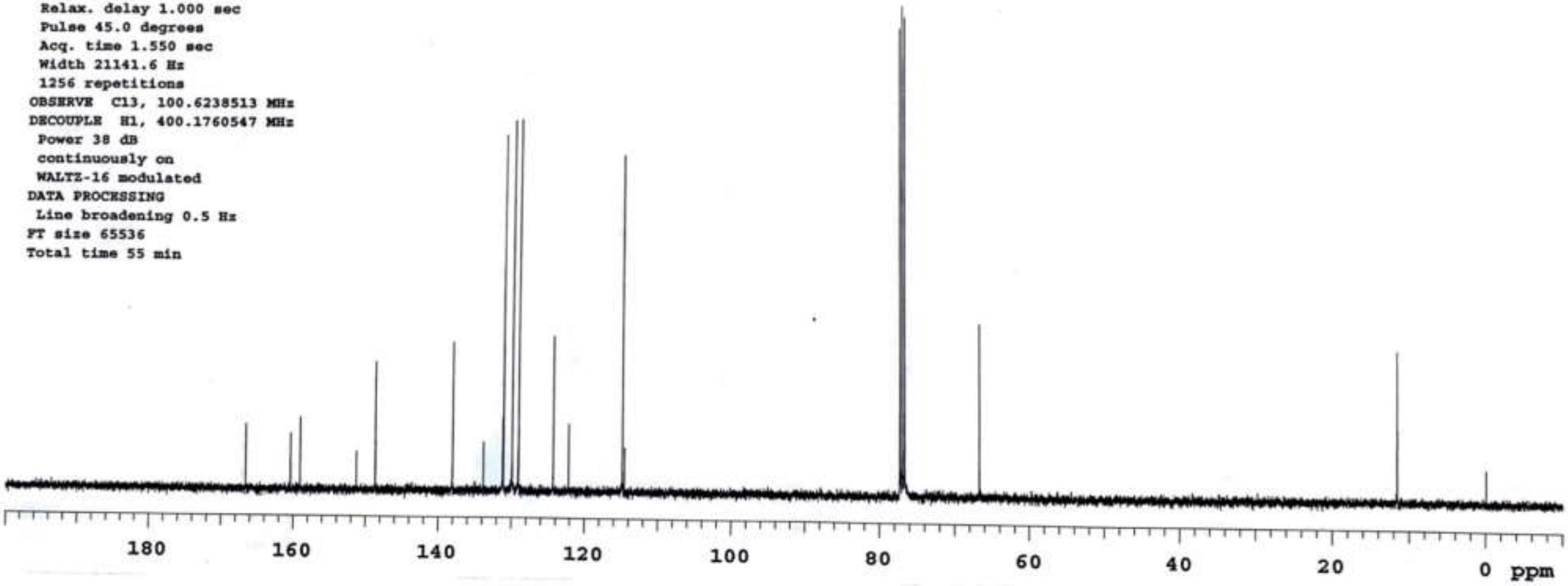

Figure S33. ^{1}H-NMR and ^{13}C-NMR spectrum of Compound **39**

Compound 39

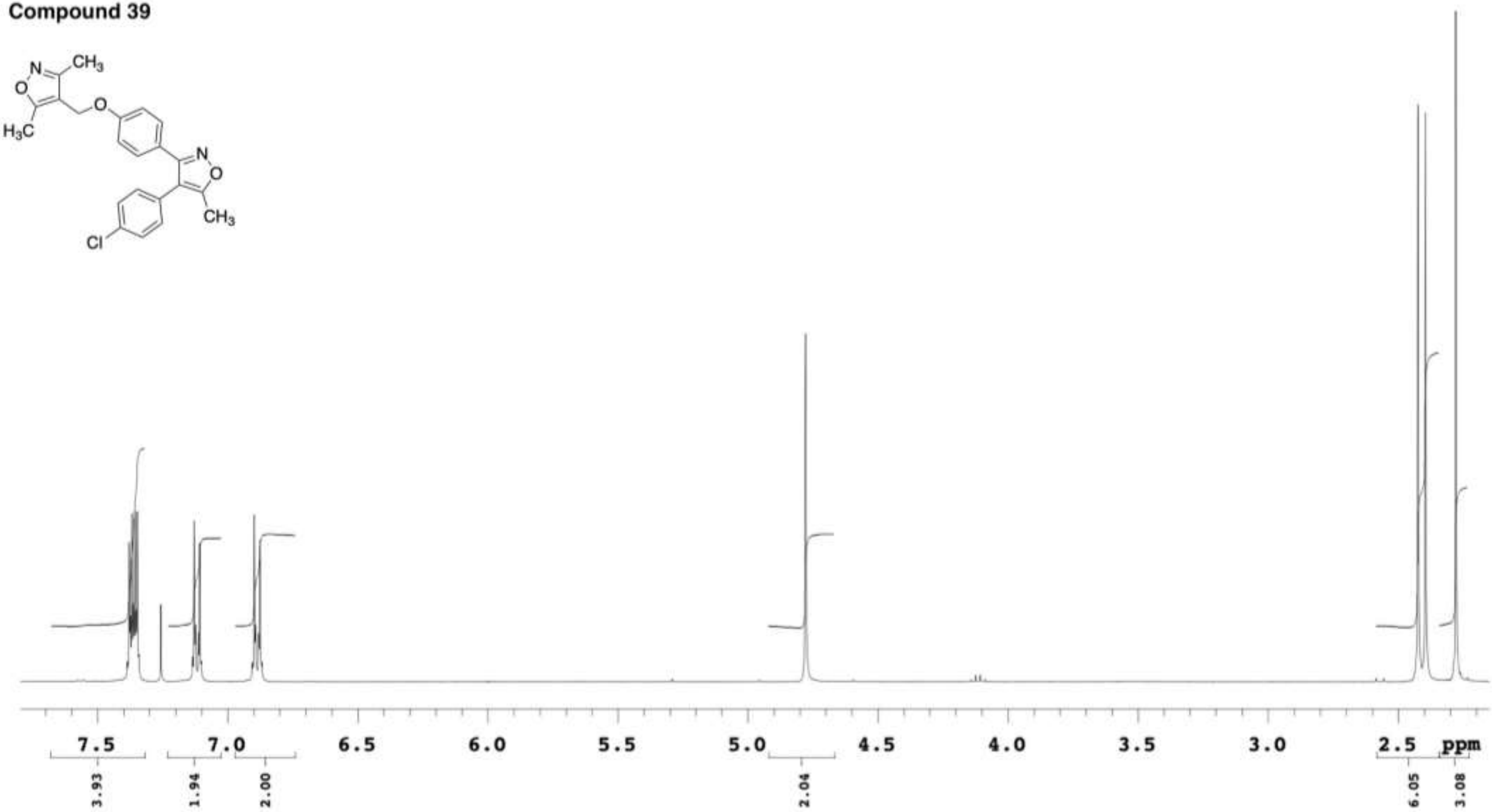

DNZ157

Sample Name:
DNZ157
Data Collected on:
mercury400-mercury400
Archive directory:
/home/vnmr1/vnmrsys/data
Sample directory:
DNZ157_20170601_01
FidFile: CARBON_02

Pulse Sequence: CARBON (s2pul)
Solvent: cdcl3
Data collected on: Jun 1 2017

Temp. 25.0 C / 298.1 K
Operator: vnmr1

Relax. delay 1.000 sec
Pulse 45.0 degrees
Acq. time 1.304 sec
Width 25125.6 Hz
1512 repetitions
OBSERVE C13, 100.6238513 MHz
DECOUPLE H1, 400.1760547 MHz
Power 38 dB
continuously on
WALTZ-16 modulated
DATA PROCESSING
Line broadening 0.5 Hz
FT size 65536
Total time 1 hr

Compound 39

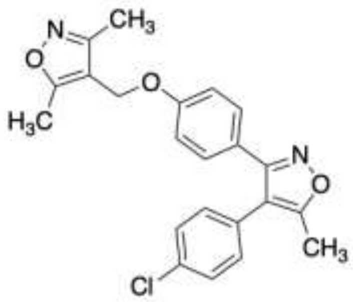

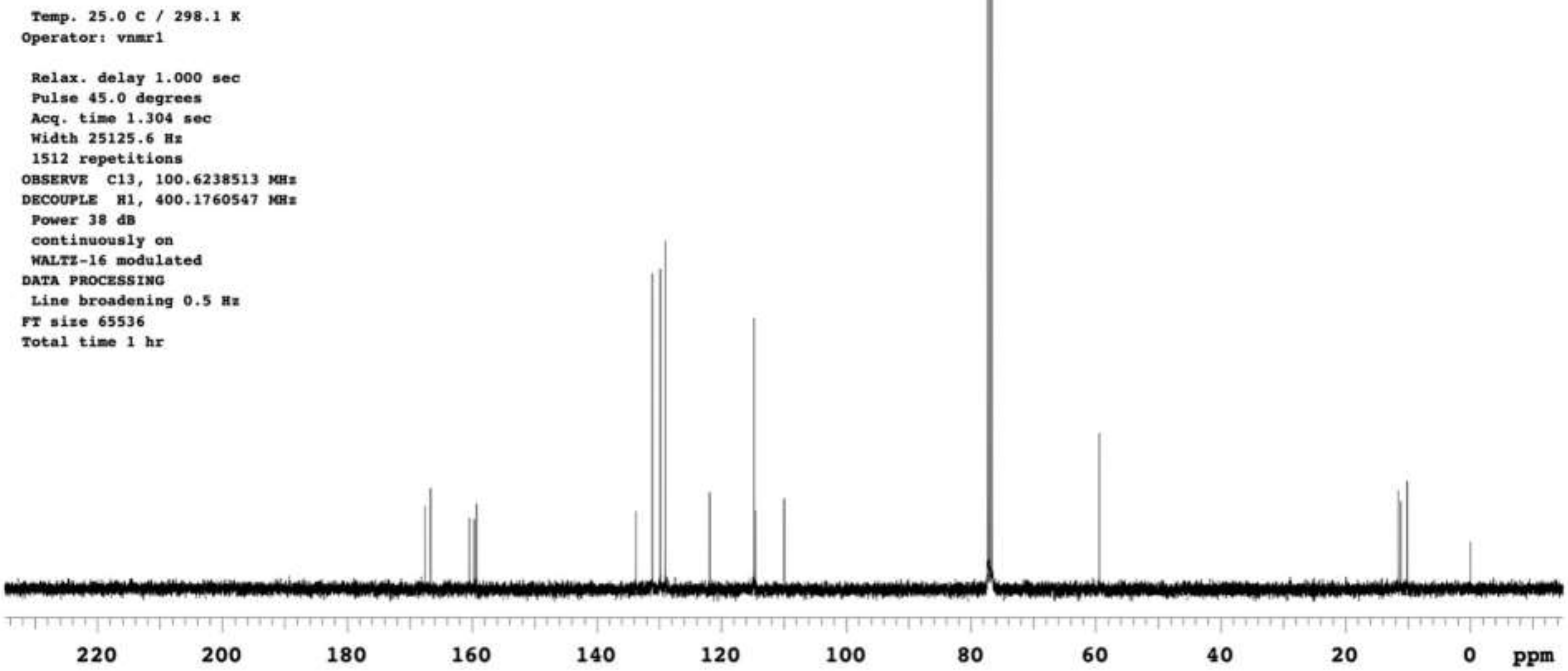

Figure S34. ^{1}H-NMR and ^{13}C-NMR spectrum of Compound **40**

Compound 40

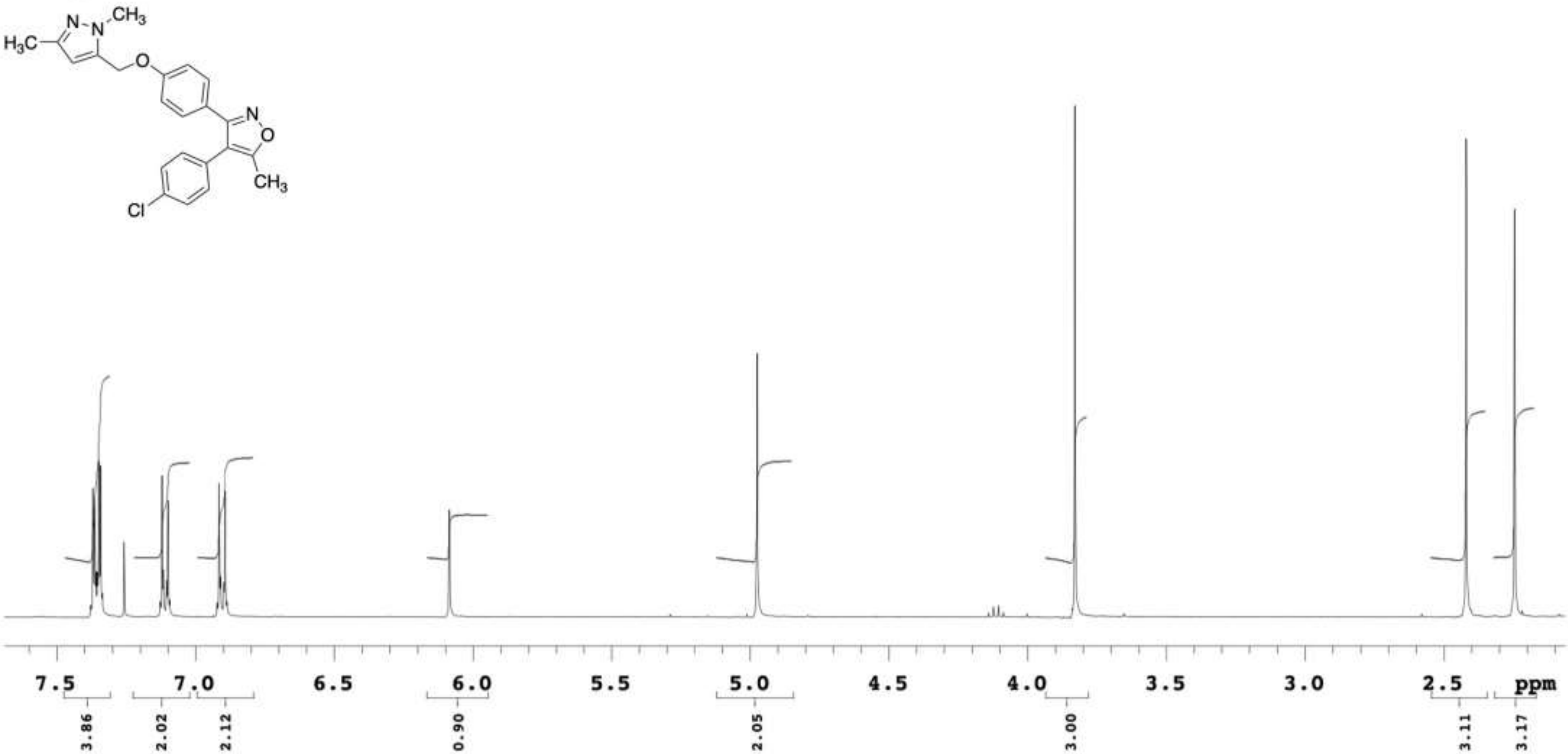

Compound 40

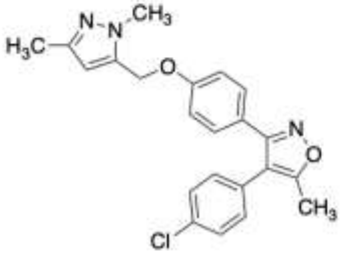

DNZ158

Sample Name:
DNZ158
Data Collected on:
mercury400-mercury400
Archive directory:
/home/vnmr1/vnmrsys/data
Sample directory:
DNZ158_20170601_01
FidFile: current

Pulse Sequence: CARBON (s2pul)
Solvent: cdcl3
Data collected on: Jun 1 2017

Temp. 25.0 C / 298.1 K
Operator: vnmr1

Relax. delay 1.000 sec
Pulse 45.0 degrees
Acq. time 1.550 sec
Width 21141.6 Hz
1792 repetitions
OBSERVE C13, 100.6238513 MHz
DECOUPLE H1, 400.1760547 MHz
Power 38 dB
continuously on
WALTZ-16 modulated
DATA PROCESSING
Line broadening 0.5 Hz
FT size 65536
Total time 2 hr, 12 min

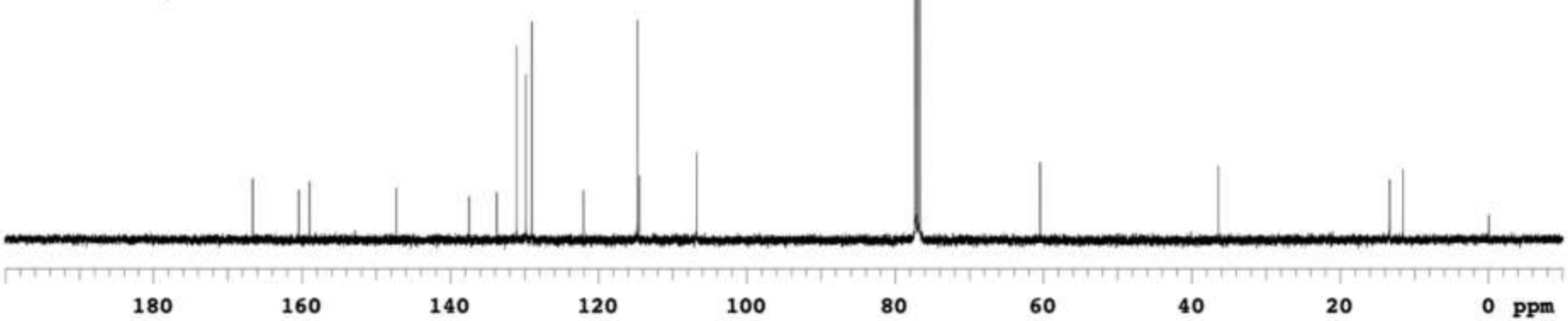

Figure S35. ^{1}H-NMR and ^{13}C-NMR spectrum of Compound **41**

Compound 41

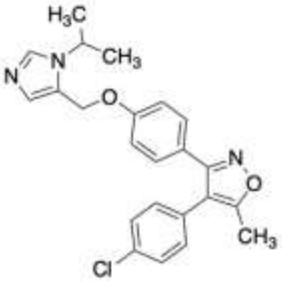

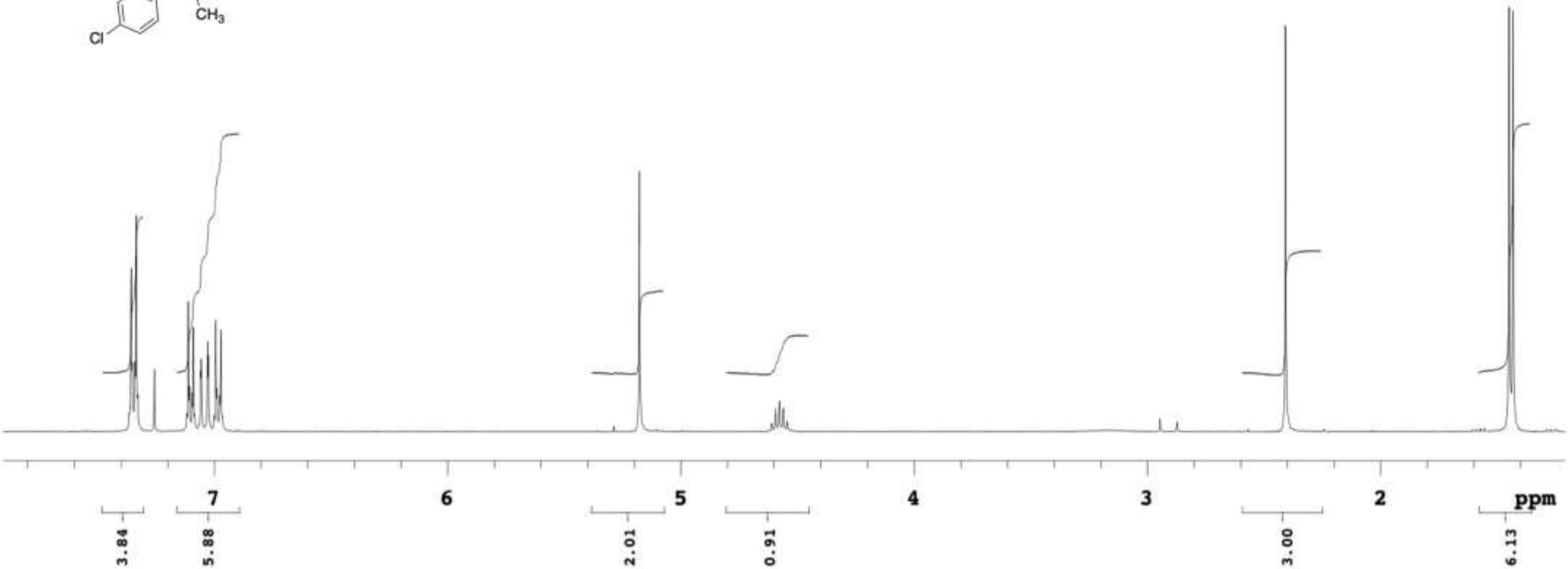

DNZ159

Sample Name:
DNZ159
Data Collected on:
mercury400-mercury400
Archive directory:
/home/vnmr1/vnmrsys/data
Sample directory:
DNZ159_20170601_01
FidFile: CARBON_02

Pulse Sequence: CARBON (s2pul)
Solvent: cdcl3
Data collected on: Jun 1 2017

Temp. 25.0 C / 298.1 K
Operator: vnmr1

Relax. delay 1.000 sec
Pulse 45.0 degrees
Acq. time 1.550 sec
Width 21141.6 Hz
2000 repetitions
OBSERVE C13, 100.6238513 MHz
DECOUPLE H1, 400.1760547 MHz
Power 38 dB
continuously on
WALTZ-16 modulated
DATA PROCESSING
Line broadening 0.5 Hz
FT size 65536
Total time 1 hr, 28 min

Compound 41

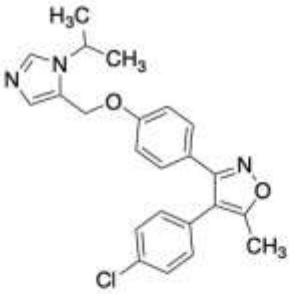

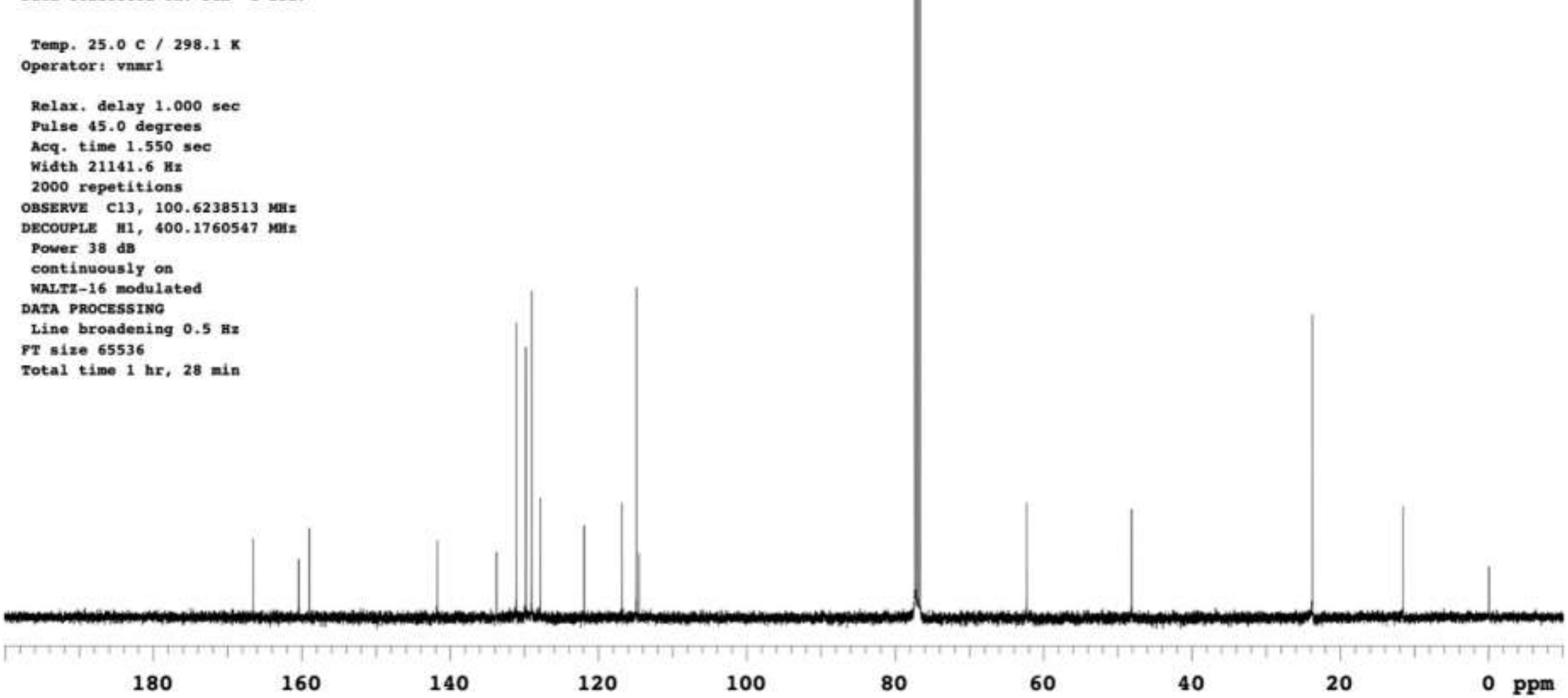

Figure S36. ^{1}H-NMR and ^{13}C-NMR spectrum of Compound **42**

Compound 42

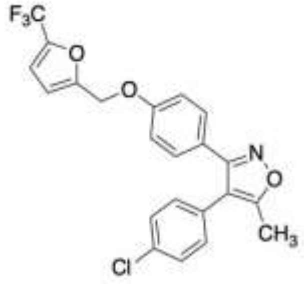

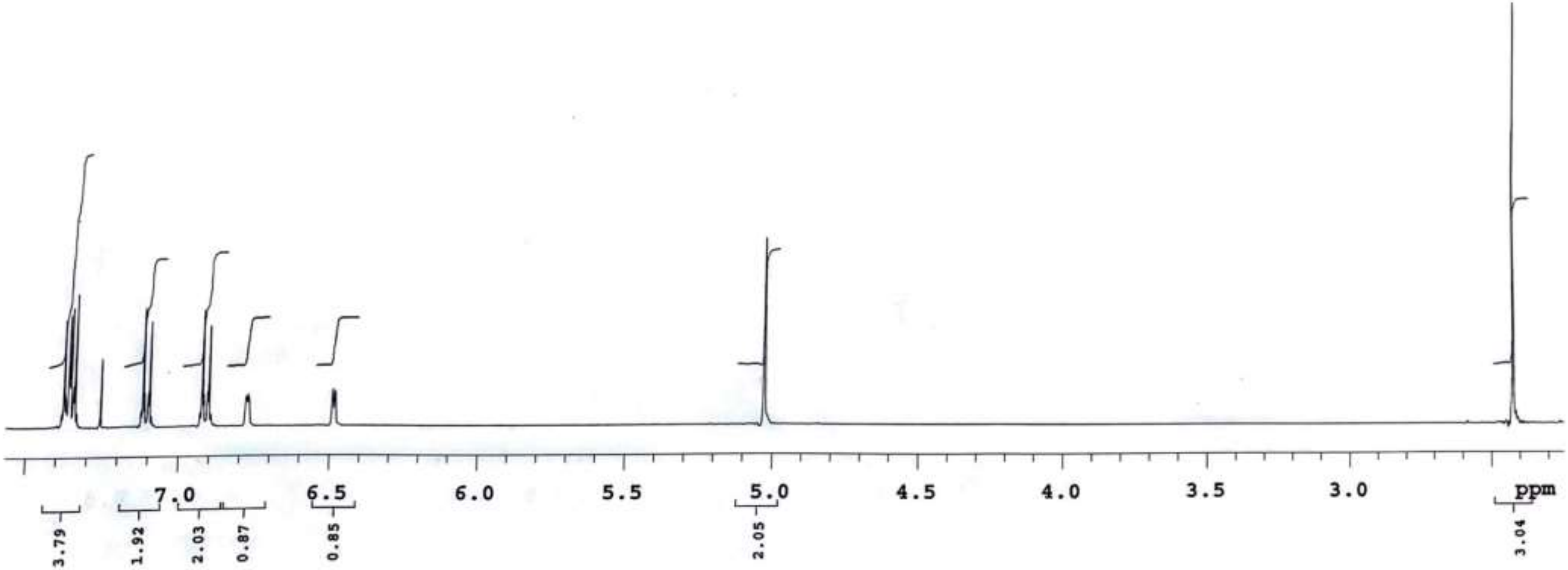

DNZ71

Sample Name:
DNZ71
Data Collected on:
mercury400-mercury400
Archive directory:
/home/vnmr1/vnmrsys/data
Sample directory:
DNZ71_20160825_01
FidFile: CARBON

Pulse Sequence: CARBON (s2pul)
Solvent: cdcl3
Data collected on: Aug 25 2016

Temp. 32.0 C / 305.1 K
Operator: vnmr1

Relax. delay 1.000 sec
Pulse 45.0 degrees
Acq. time 1.550 sec
Width 21141.6 Hz
1256 repetitions
OBSERVE C13, 100.6238513 MHz
DECOUPLE H1, 400.1760547 MHz
Power 38 dB
continuously on
WALTZ-16 modulated
DATA PROCESSING
Line broadening 0.5 Hz
FT size 65536
Total time 55 min

Compound 42

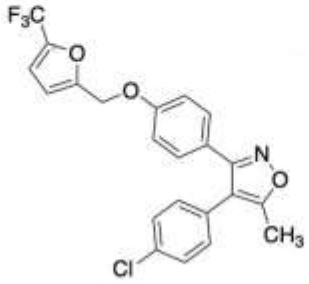

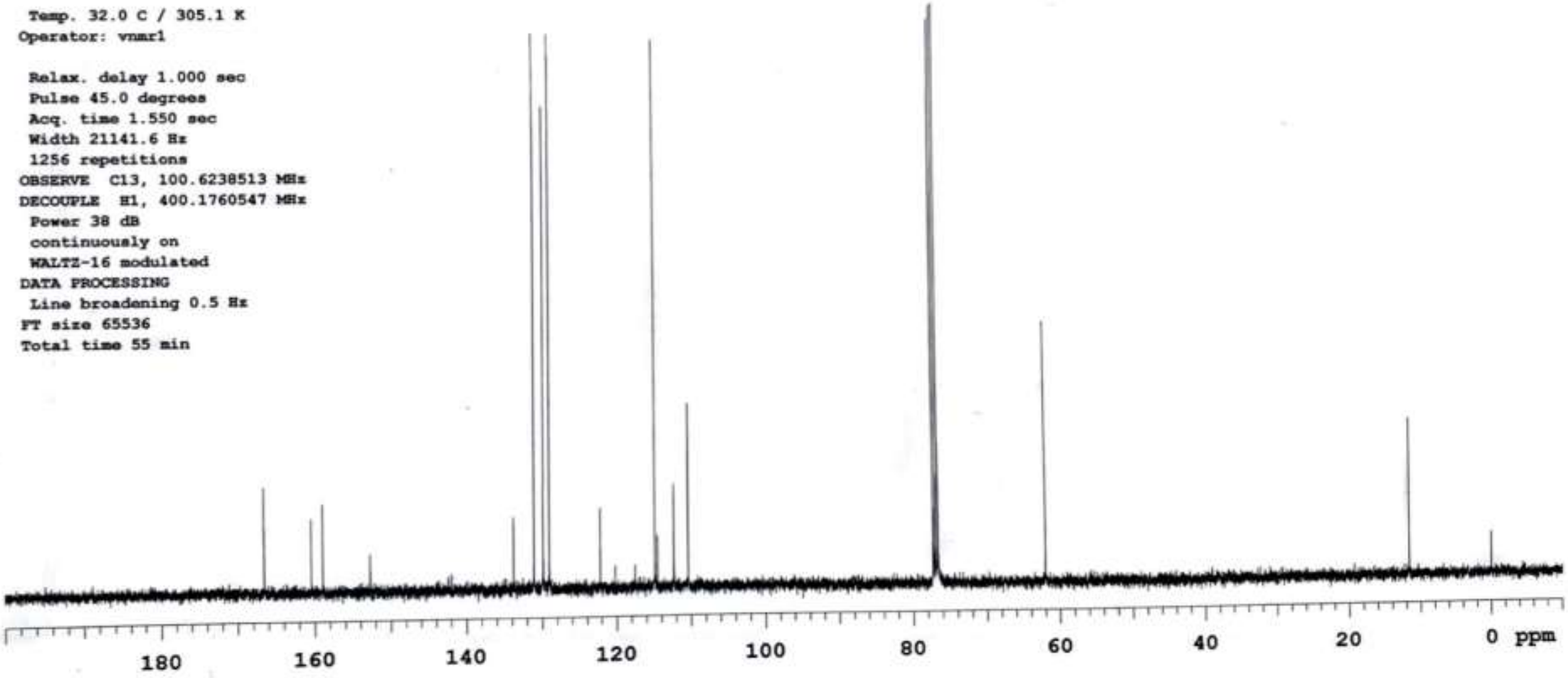

Figure S37. ^{1}H-NMR and ^{13}C-NMR spectrum of Compound **44**

Compound 44

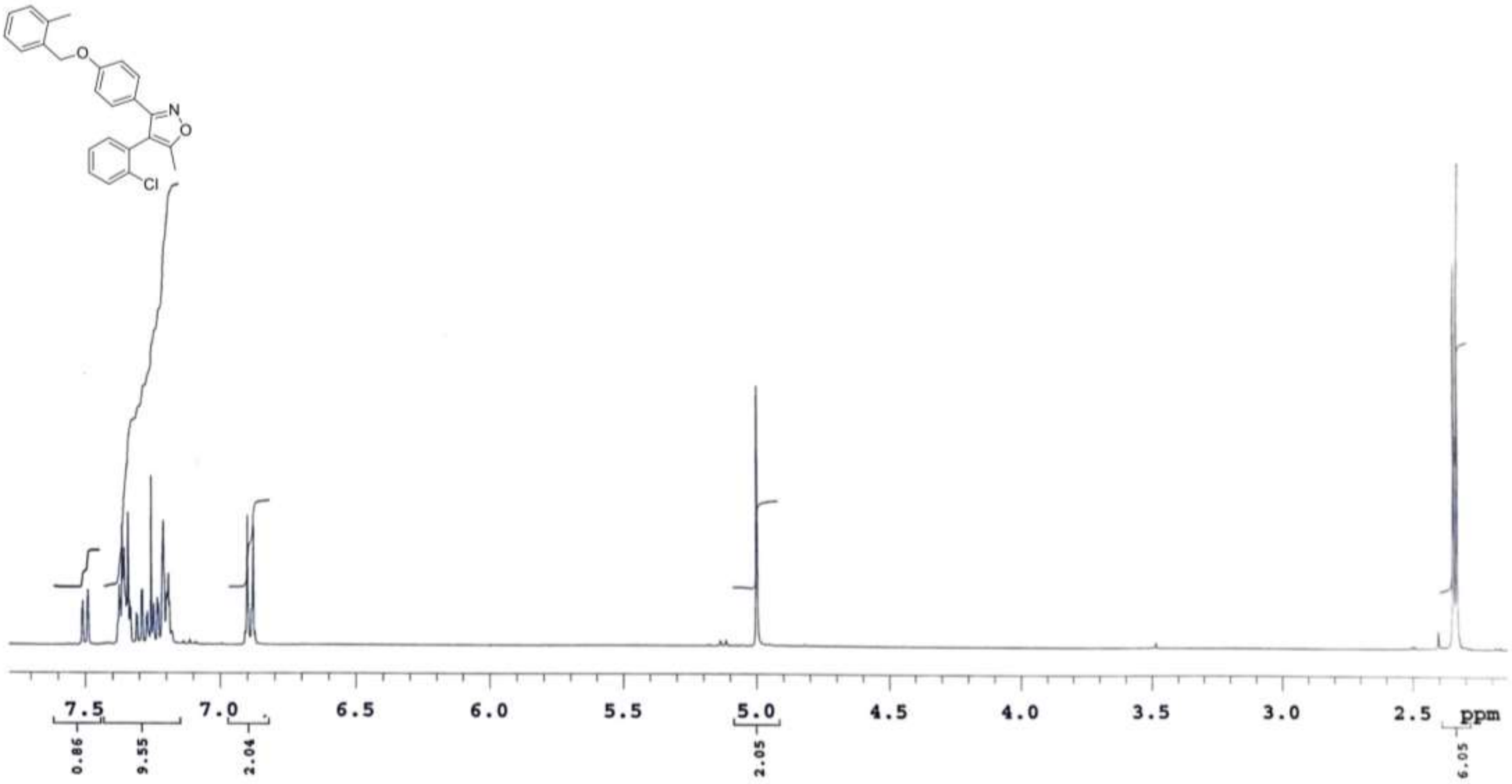

SMY130

Sample Name:
SMY130
Data Collected on:
mercury400-mercury400
Archive directory:
/home/vnmr1/vnmrsys/data
Sample directory:
SMY130_20160826_01
FidFile: CARBON_01

Pulse Sequence: CARBON (s2pul)
Solvent: cdcl3
Data collected on: Aug 26 2016

Temp. 25.0 C / 298.1 K
Operator: vnmr1

Relax. delay 1.000 sec
Pulse 45.0 degrees
Acq. time 1.550 sec
Width 21141.6 Hz
2256 repetitions
OBSERVE C13, 100.6238513 MHz
DECOUPLE H1, 400.1760547 MHz
Power 38 dB
continuously on
WALTZ-16 modulated
DATA PROCESSING
Line broadening 0.5 Hz
FT size 65536
Total time 1 hr, 39 min

Compound 44

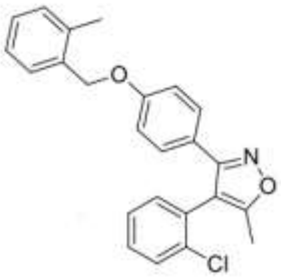

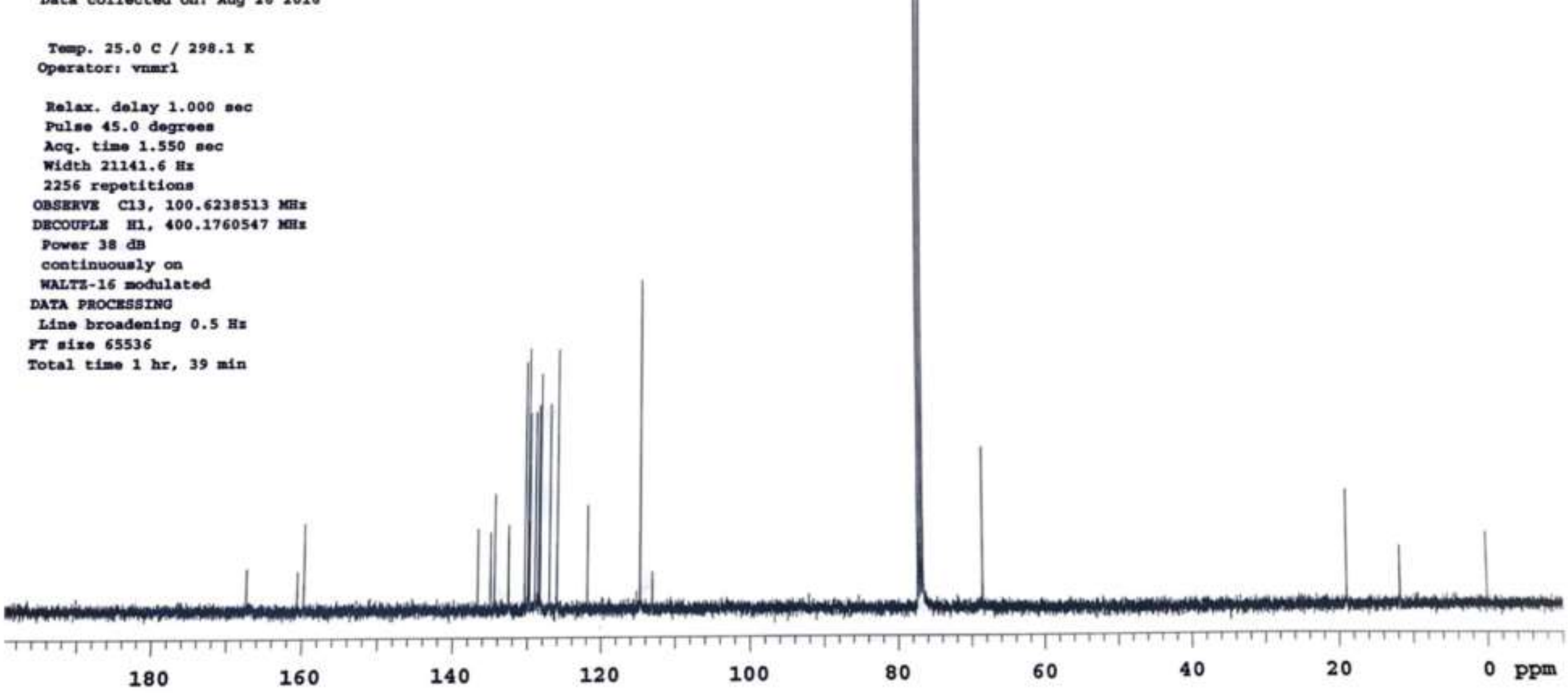

Figure S38. ^{1}H-NMR and ^{13}C-NMR spectrum of Compound **45**

Compound 45

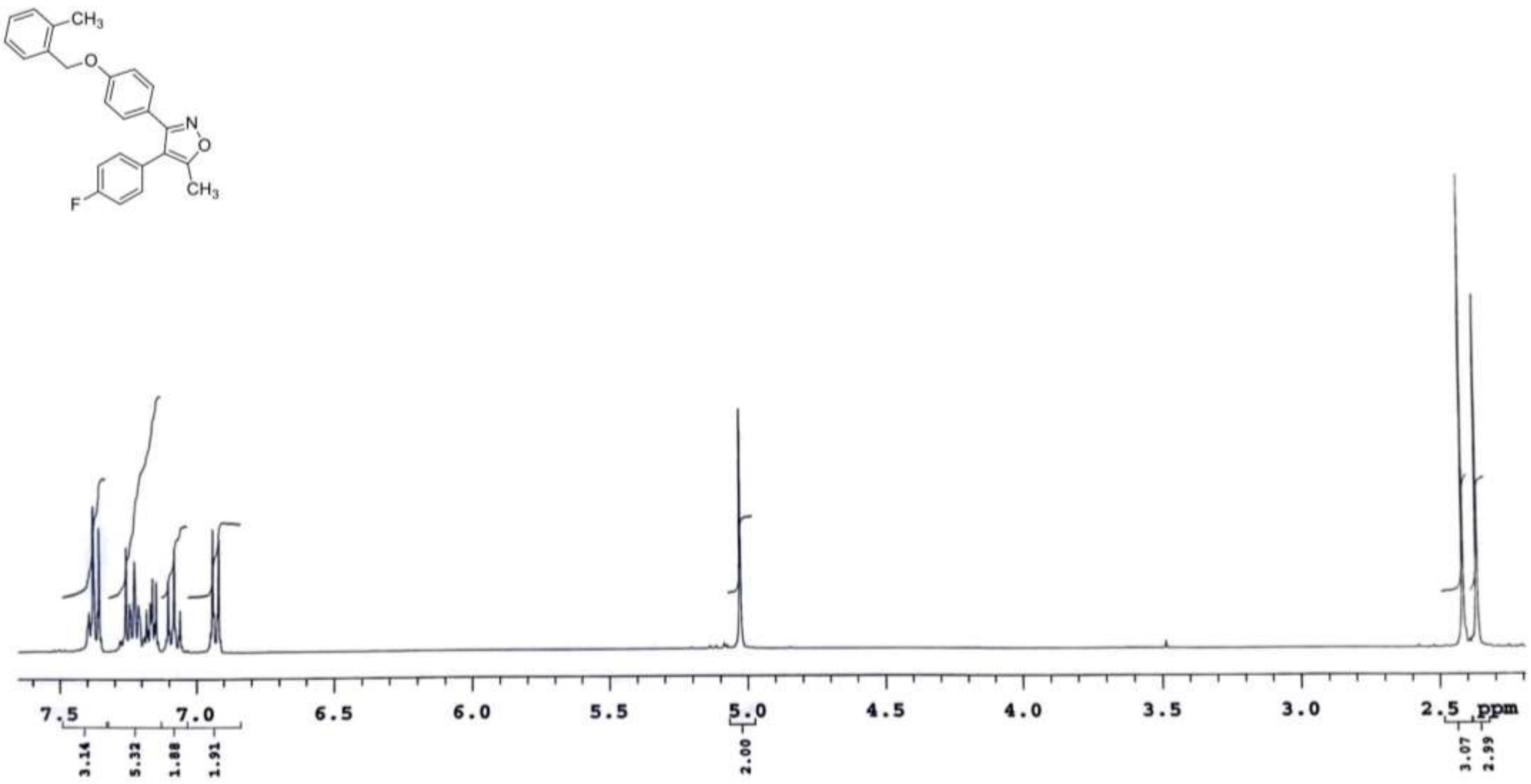

SMY-132

Sample Name:
SMY-132
Data Collected on:
mercury400-mercury400
Archive directory:
/home/vnmr1/vnmrsys/data
Sample directory:
SMY-132_20160930_01
FidFile: current

Pulse Sequence: CARBON (s2pul)
Solvent: cdcl3
Data collected on: Sep 30 2016

Temp. 25.0 C / 298.1 K
Operator: vnmr1

Relax. delay 1.000 sec
Pulse 45.0 degrees
Acq. time 1.304 sec
Width 25125.6 Hz
1216 repetitions
OBSERVE C13, 100.6238513 MHz
DECOUPLE H1, 400.1760547 MHz
Power 38 dB
continuously on
WALTZ-16 modulated
DATA PROCESSING
Line broadening 0.5 Hz
FT size 65536
Total time 1 hr

Compound 45

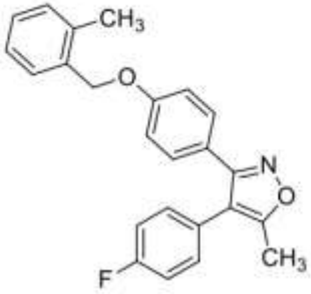

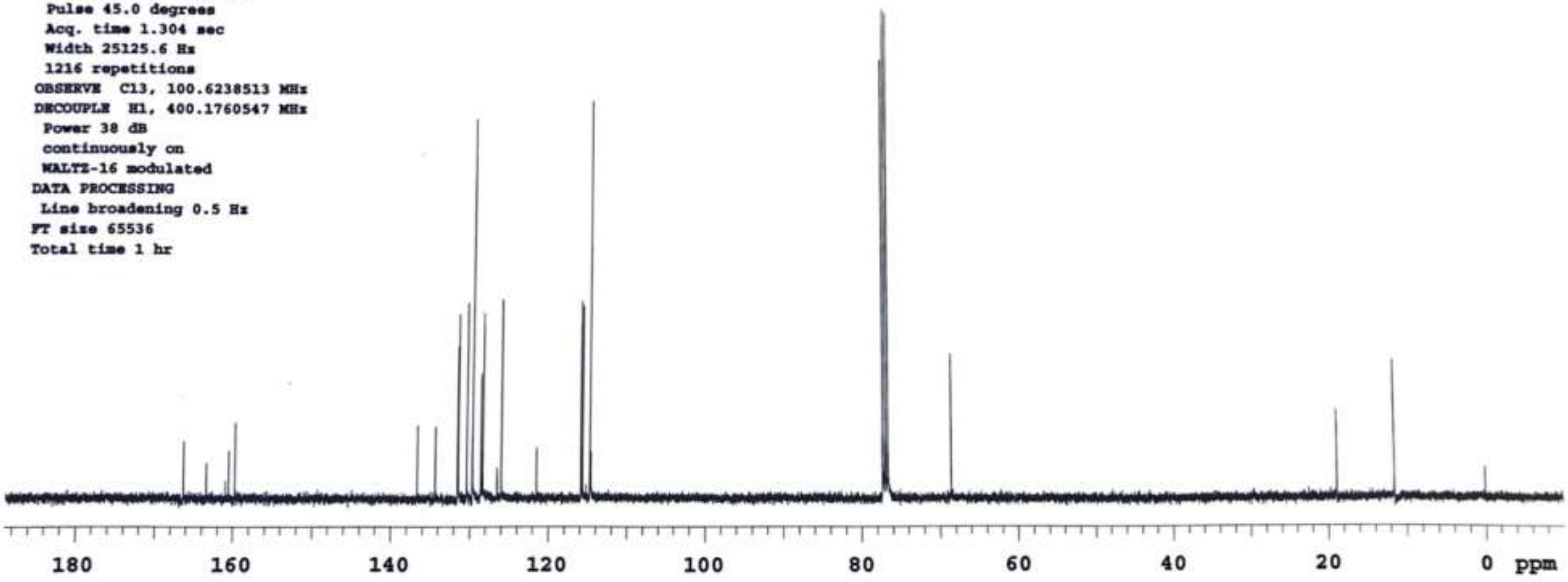

Figure S39. ^{1}H-NMR and ^{13}C-NMR spectrum of Compound **46**

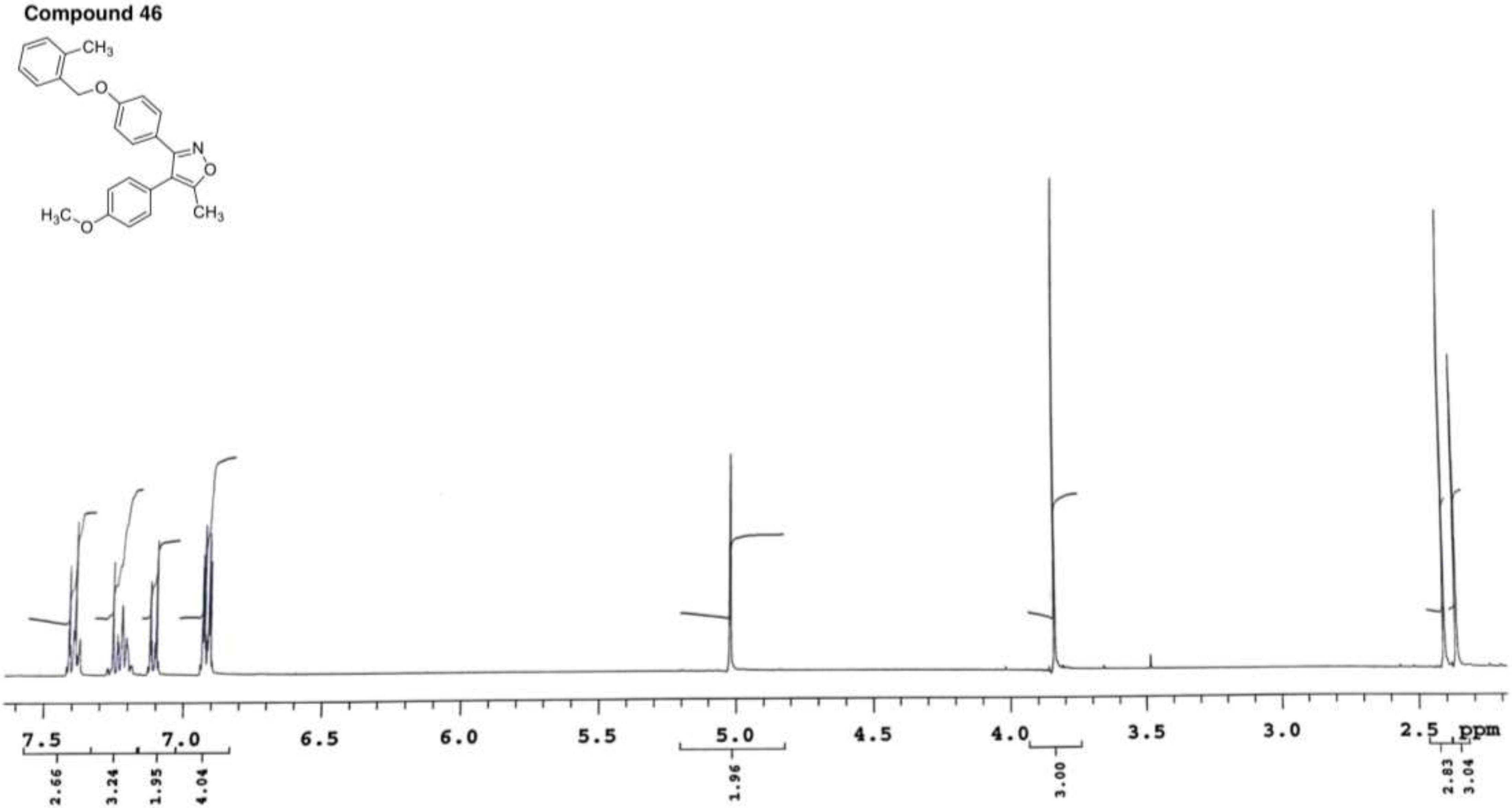

SMY136

Sample Name:
SMY136
Data Collected on:
mercury400-mercury400
Archive directory:
/home/vnmr1/vnmrsys/data
Sample directory:
SMY136_20160827_01
FidFile: current

Pulse Sequence: CARBON (s2pul)
Solvent: cdcl3
Data collected on: Aug 27 2016

Temp. 25.0 C / 298.1 K
Operator: vnmr1

Relax. delay 1.000 sec
Pulse 45.0 degrees
Acq. time 1.550 sec
Width 21141.6 Hz
256 repetitions
OBSERVE C13, 100.6234548 MHz
DECOUPLE H1, 400.1760547 MHz
Power 38 dB
continuously on
WALTZ-16 modulated
DATA PROCESSING
Line broadening 0.5 Hz
FT size 65536
Total time 1 hr, 28 min

Compound 46

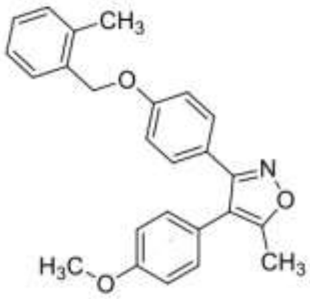

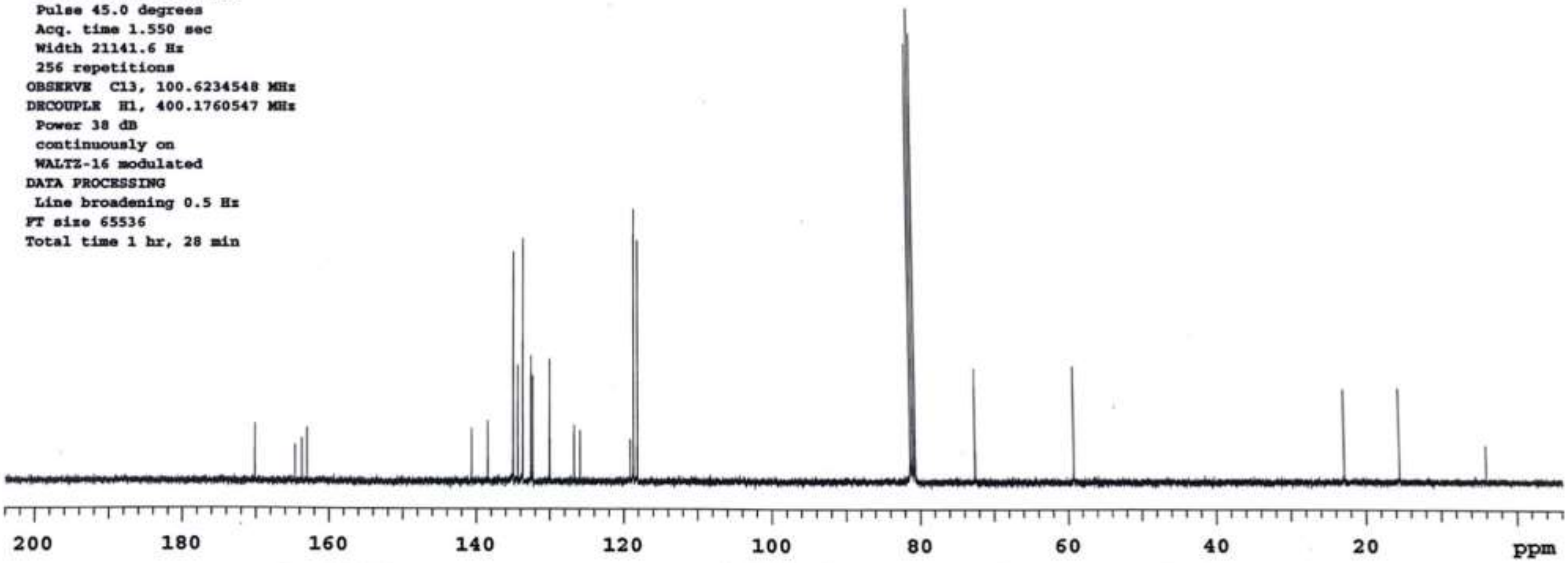

Figure S40. ^{1}H-NMR and ^{13}C-NMR spectrum of Compound **47**

Compound 47

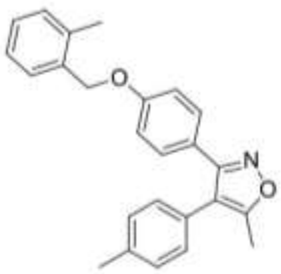

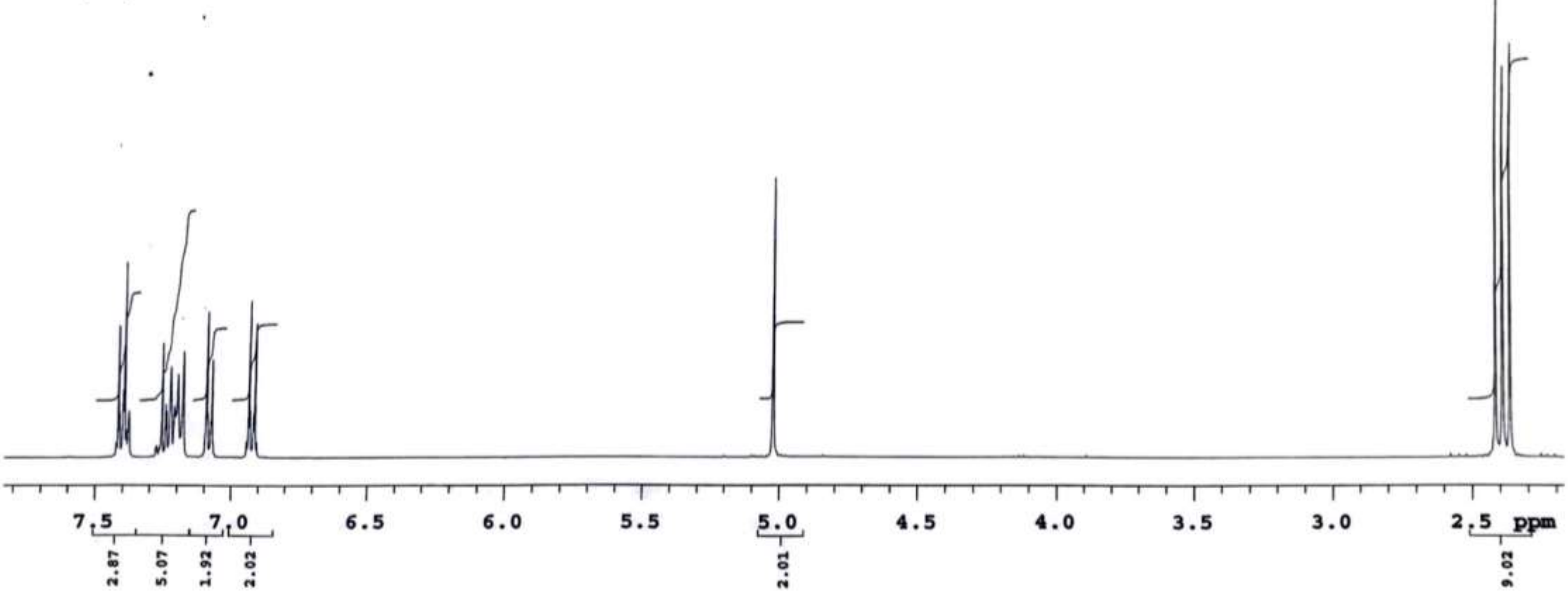

SMY-143

Sample Name:
SMY-143
Data Collected on:
mercury400-mercury400
Archive directory:
/home/vnmr1/vnmrsys/data
Sample directory:
SMY-143_20160930_01
FidFile: current

Pulse Sequence: CARBON (s2pul)
Solvent: cdcl3
Data collected on: Sep 30 2016

Temp. 25.0 C / 298.1 K
Operator: vnmr1

Relax. delay 1.000 sec
Pulse 45.0 degrees
Acq. time 1.304 sec
Width 25125.6 Hz
832 repetitions
OBSERVE C13, 100.6238513 MHz
DECOUPLE H1, 400.1760547 MHz
Power 38 dB
continuously on
WALTZ-16 modulated
DATA PROCESSING
Line broadening 0.5 Hz
FT size 65536
Total time 1 hr

Compound 47

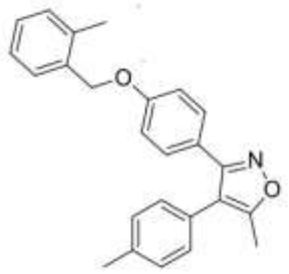

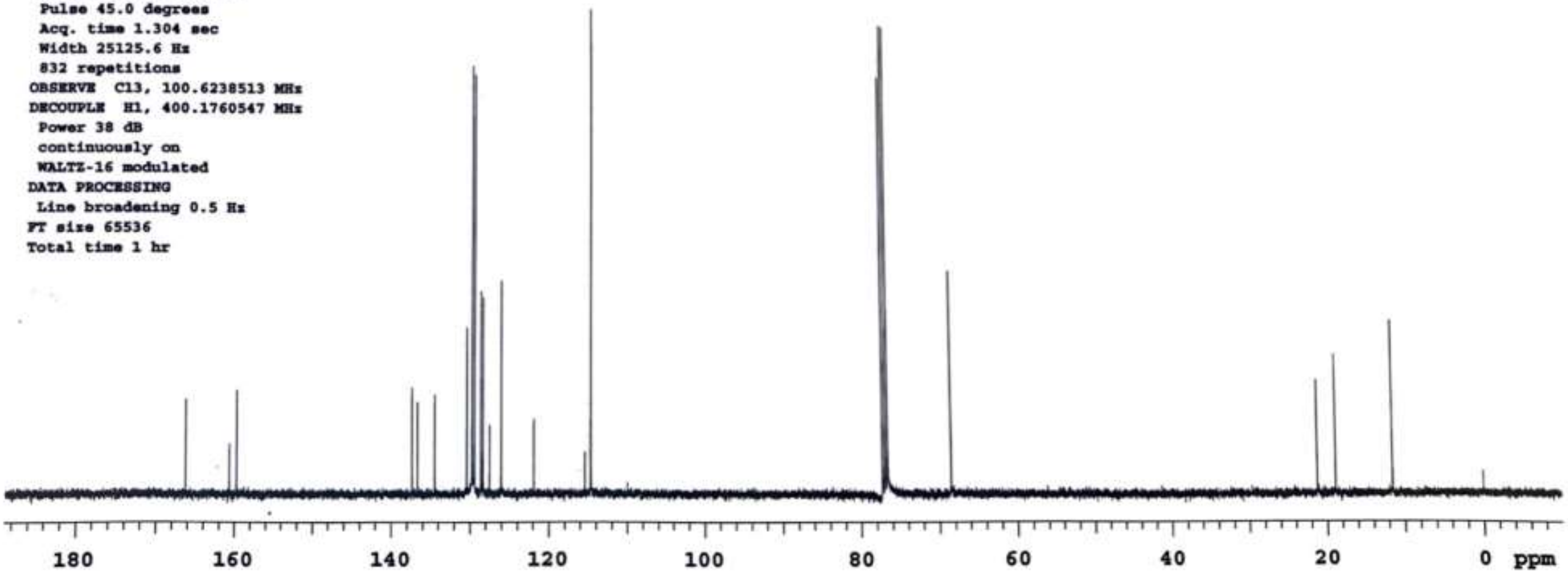

Figure S41. ^{1}H-NMR and ^{13}C-NMR spectrum of Compound **48**

Compound 48

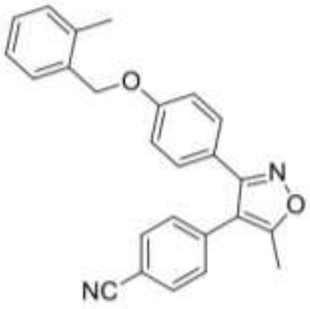

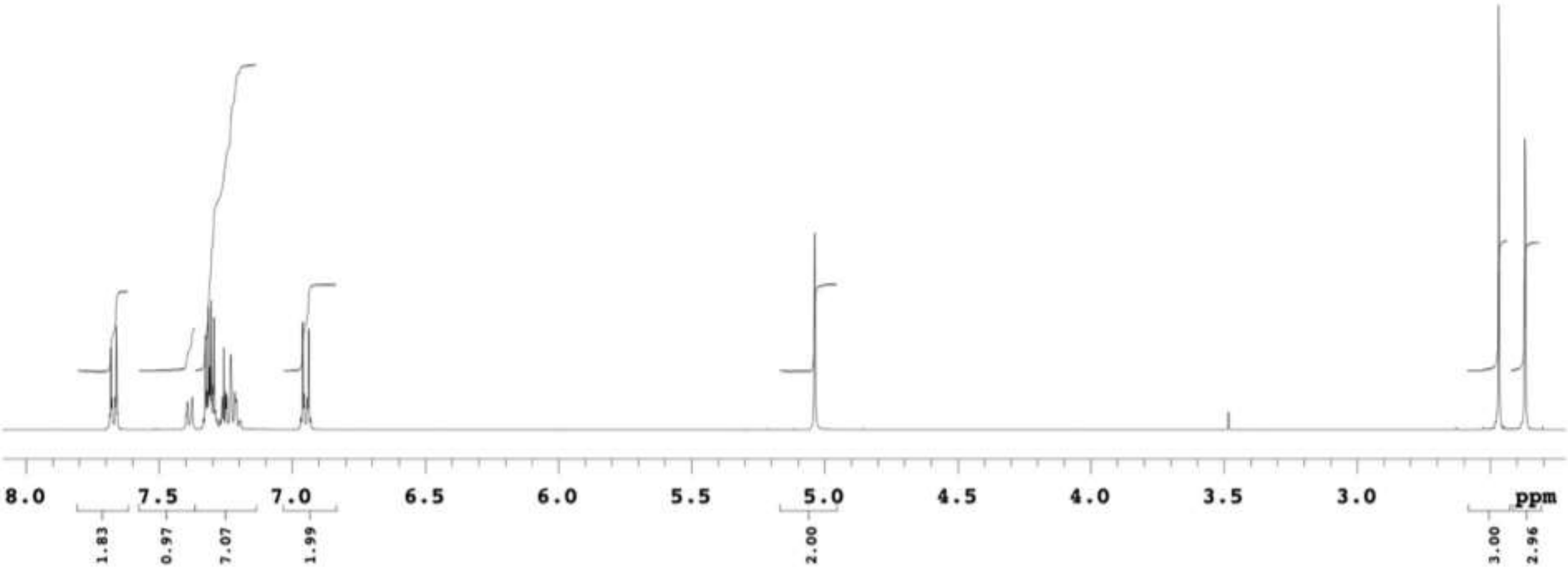

SMY145

Sample Name:
SMY145
Data Collected on:
mercury400-mercury400
Archive directory:
/home/vnmr1/vnmrsys/data
Sample directory:
SMY145_20161010_01
FidFile: current

Pulse Sequence: CARBON (s2pul)
Solvent: cdcl3
Data collected on: Oct 10 2016

Temp. 25.0 C / 298.1 K
Operator: vnmr1

Relax. delay 1.000 sec
Pulse 45.0 degrees
Acq. time 1.304 sec
Width 25125.6 Hz
640 repetitions
OBSERVE C13, 100.6238513 MHz
DECOUPLE H1, 400.1760547 MHz
Power 38 dB
continuously on
WALTZ-16 modulated
DATA PROCESSING
Line broadening 0.5 Hz
FT size 65536
Total time 1 hr

Compound 48

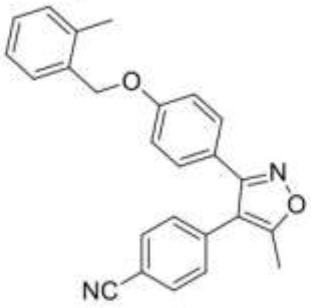

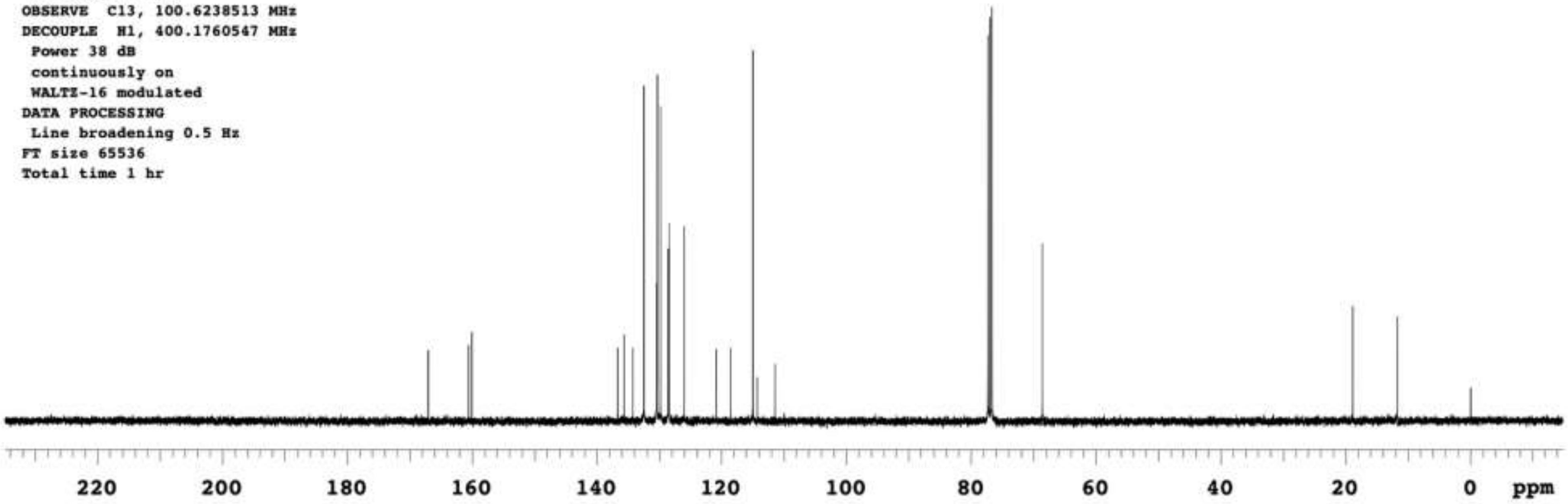

Figure S42. ^{1}H-NMR and ^{13}C-NMR spectrum of Compound **49**

Compound 49

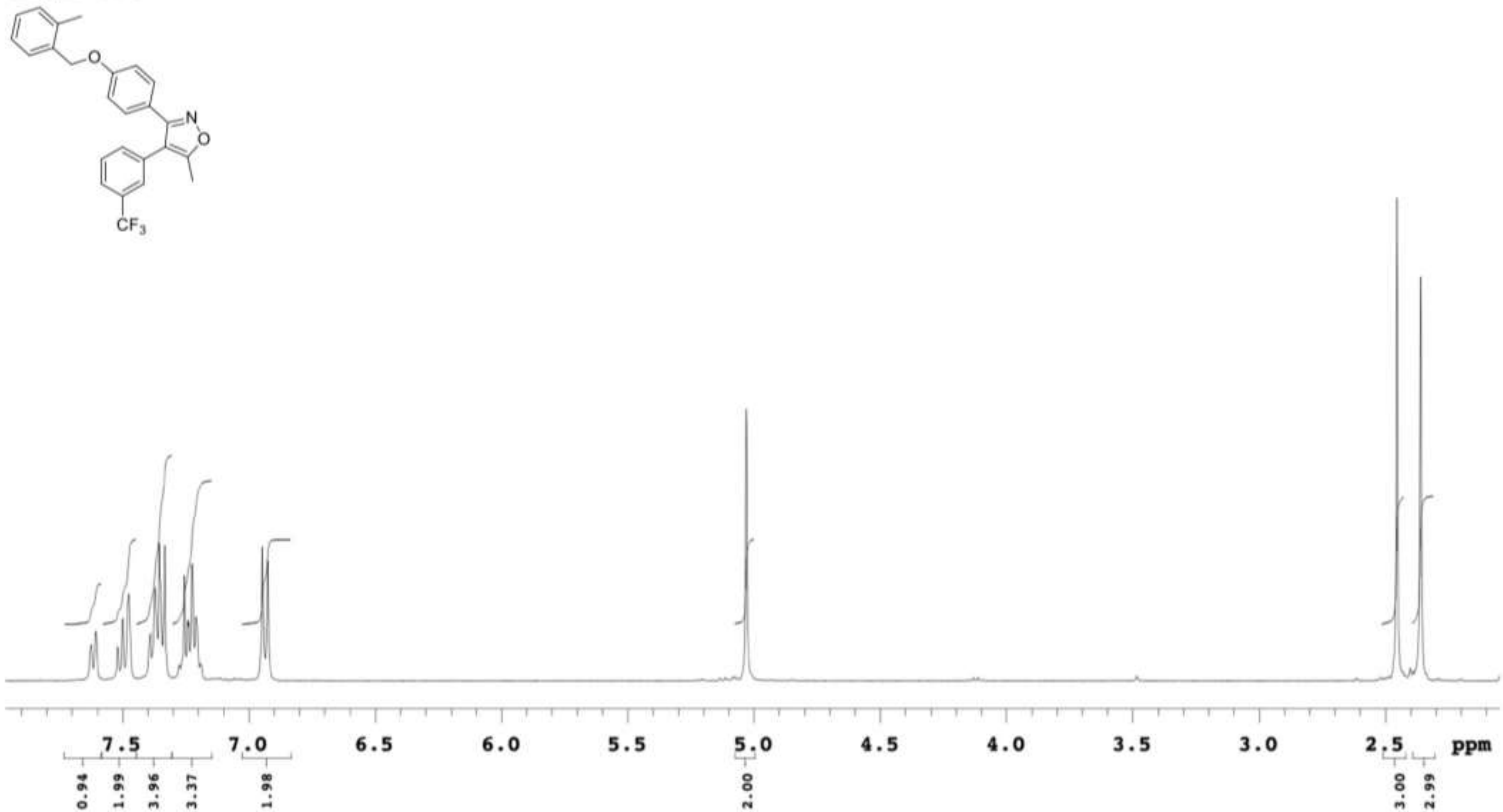

SMY151

Sample Name:
SMY151
Data Collected on:
mercury400-mercury400
Archive directory:
/home/vnmr1/vnmrsys/data
Sample directory:
SMY151_20161203_01
FidFile: CARBON_01

Pulse Sequence: CARBON (s2pul)
Solvent: cdcl3
Data collected on: Dec 3 2016

Temp. 25.0 C / 298.1 K
Operator: vnmr1

Relax. delay 1.000 sec
Pulse 45.0 degrees
Acq. time 1.550 sec
Width 21141.6 Hz
2512 repetitions
OBSERVE C13, 100.6238513 MHz
DECOUPLE H1, 400.1760547 MHz
Power 38 dB
continuously on
WALTZ-16 modulated
DATA PROCESSING
Line broadening 0.5 Hz
FT size 65536
Total time 1 hr, 50 min

Compound 49

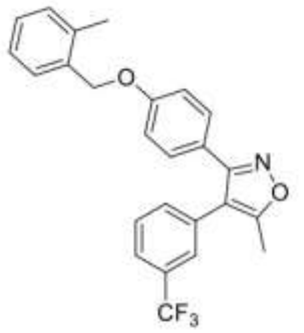

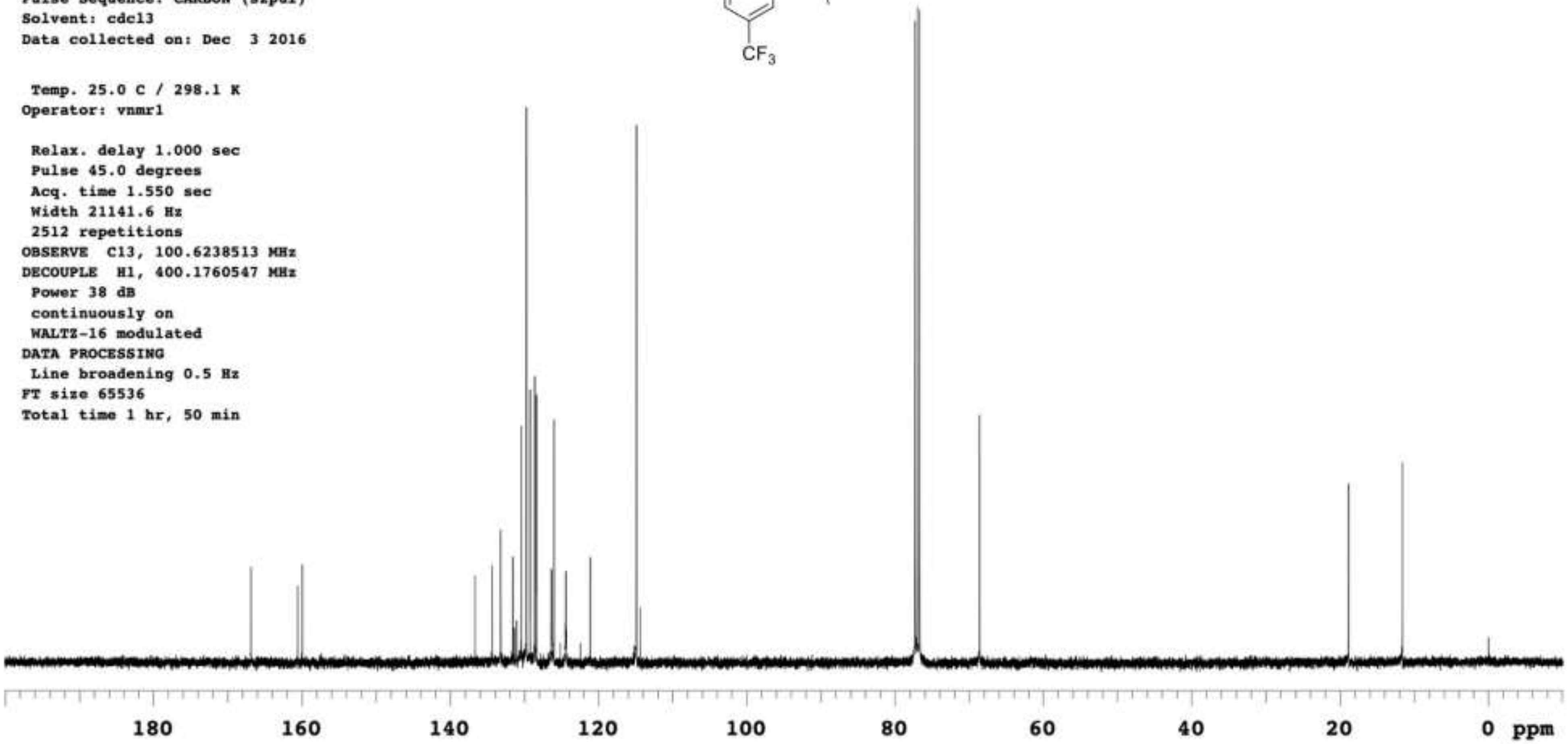

Figure S43. ^{1}H-NMR and ^{13}C-NMR spectrum of Compound **50**

Compound 50

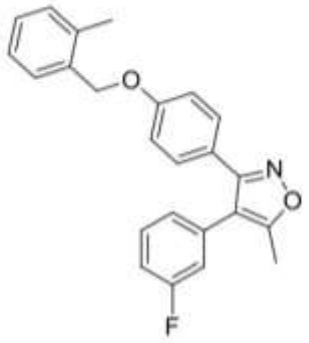

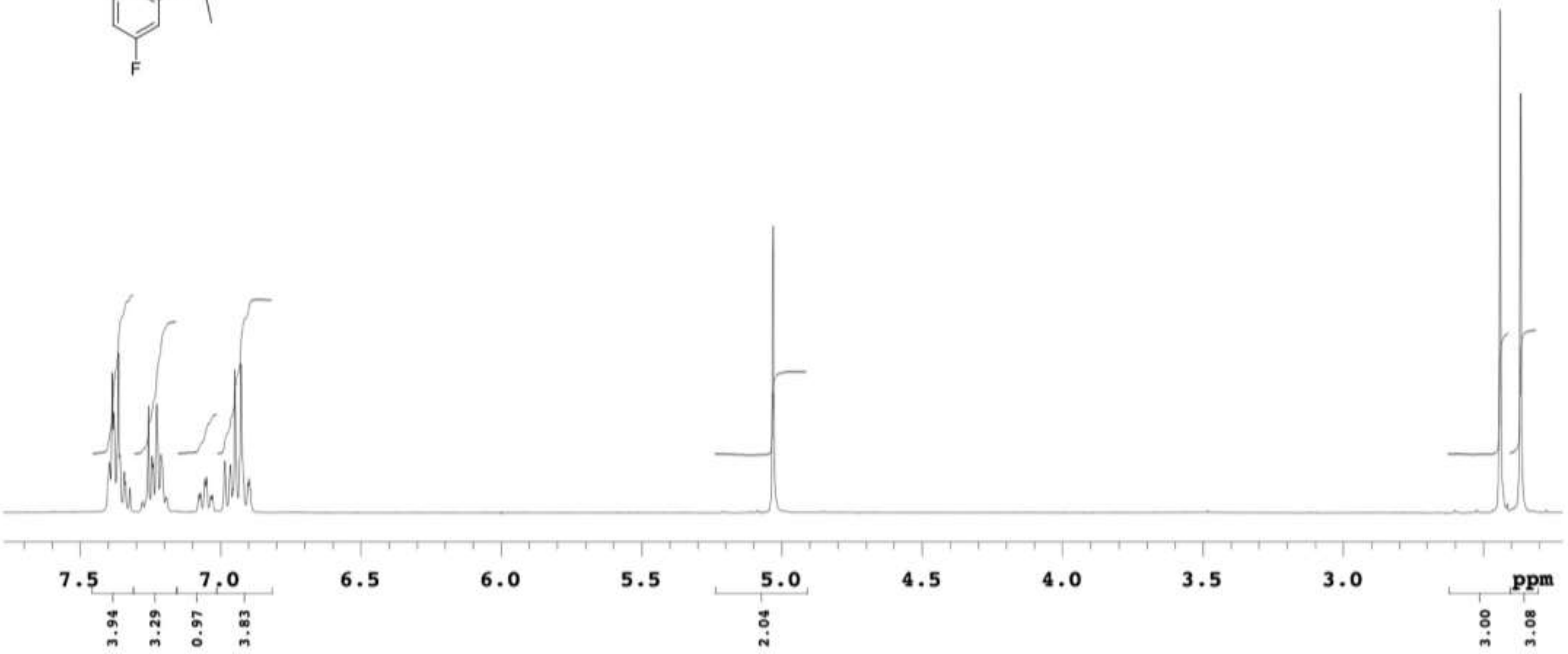

SMY153

Sample Name:
SMY153
Data Collected on:
mercury400-mercury400
Archive directory:
/home/vnmr1/vnmrsys/data
Sample directory:
SMY153_20161202_01
FidFile: CARBON_01

Pulse Sequence: CARBON (s2pul)
Solvent: cdcl3
Data collected on: Dec 2 2016

Temp. 25.0 C / 298.1 K
Operator: vnmr1

Relax. delay 1.000 sec
Pulse 45.0 degrees
Acq. time 1.304 sec
Width 25125.6 Hz
2512 repetitions
OBSERVE C13, 100.6238513 MHz
DECOUPLE H1, 400.1760547 MHz
Power 38 dB
continuously on
WALTZ-16 modulated
DATA PROCESSING
Line broadening 0.5 Hz
FT size 65536
Total time 1 hr, 40 min

Compound 50

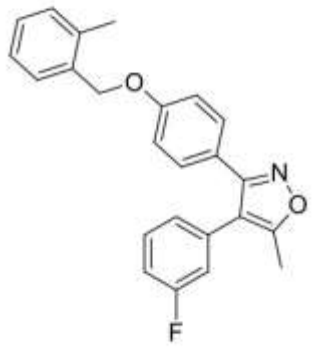

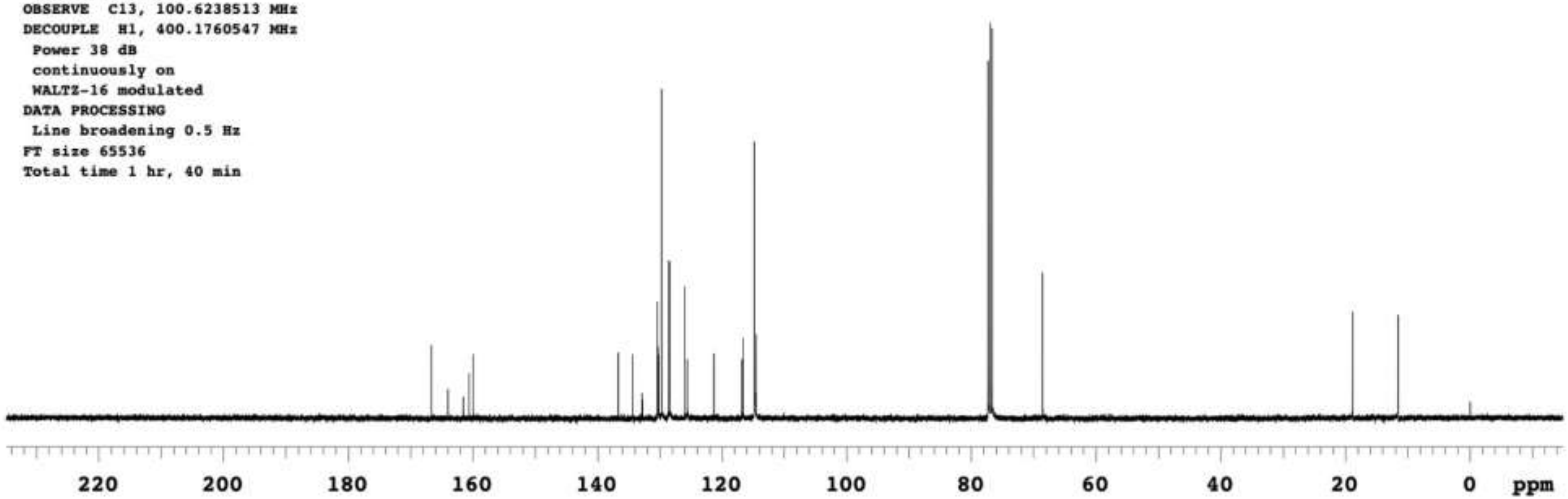

Figure S44. ^{1}H-NMR and ^{13}C-NMR spectrum of Compound **51**

Compound 51

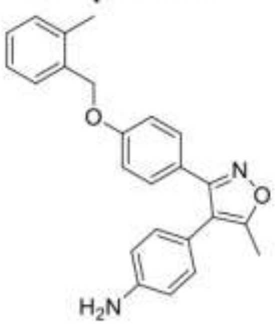

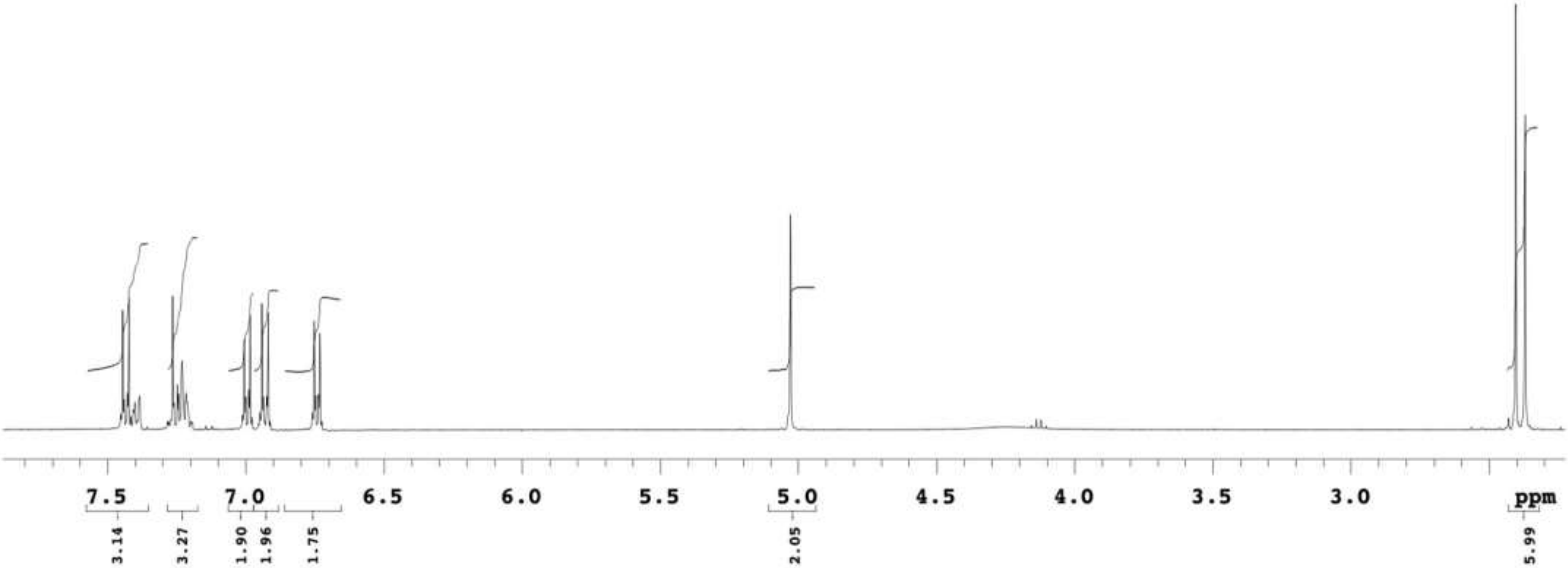

SMY-206

Sample Name:
SMY-206
Data Collected on:
mercury400-mercury400
Archive directory:
/home/vnmr1/vnmrsys/data
Sample directory:
SMY-206_20170303_01
FidFile: current

Pulse Sequence: CARBON (s2pul)
Solvent: cdcl3
Data collected on: Mar 3 2017

Temp. 26.0 C / 299.1 K
Operator: vnmr1

Relax. delay 1.000 sec
Pulse 45.0 degrees
Acq. time 1.304 sec
Width 25125.6 Hz
704 repetitions
OBSERVE C13, 100.6238513 MHz
DECOUPLE H1, 400.1760547 MHz
Power 38 dB
continuously on
WALTZ-16 modulated
DATA PROCESSING
Line broadening 0.5 Hz
FT size 65536
Total time 1 hr

Compound 51

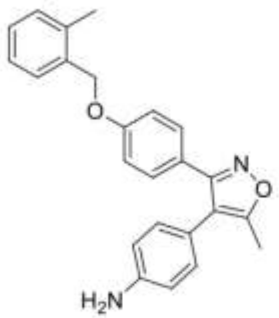

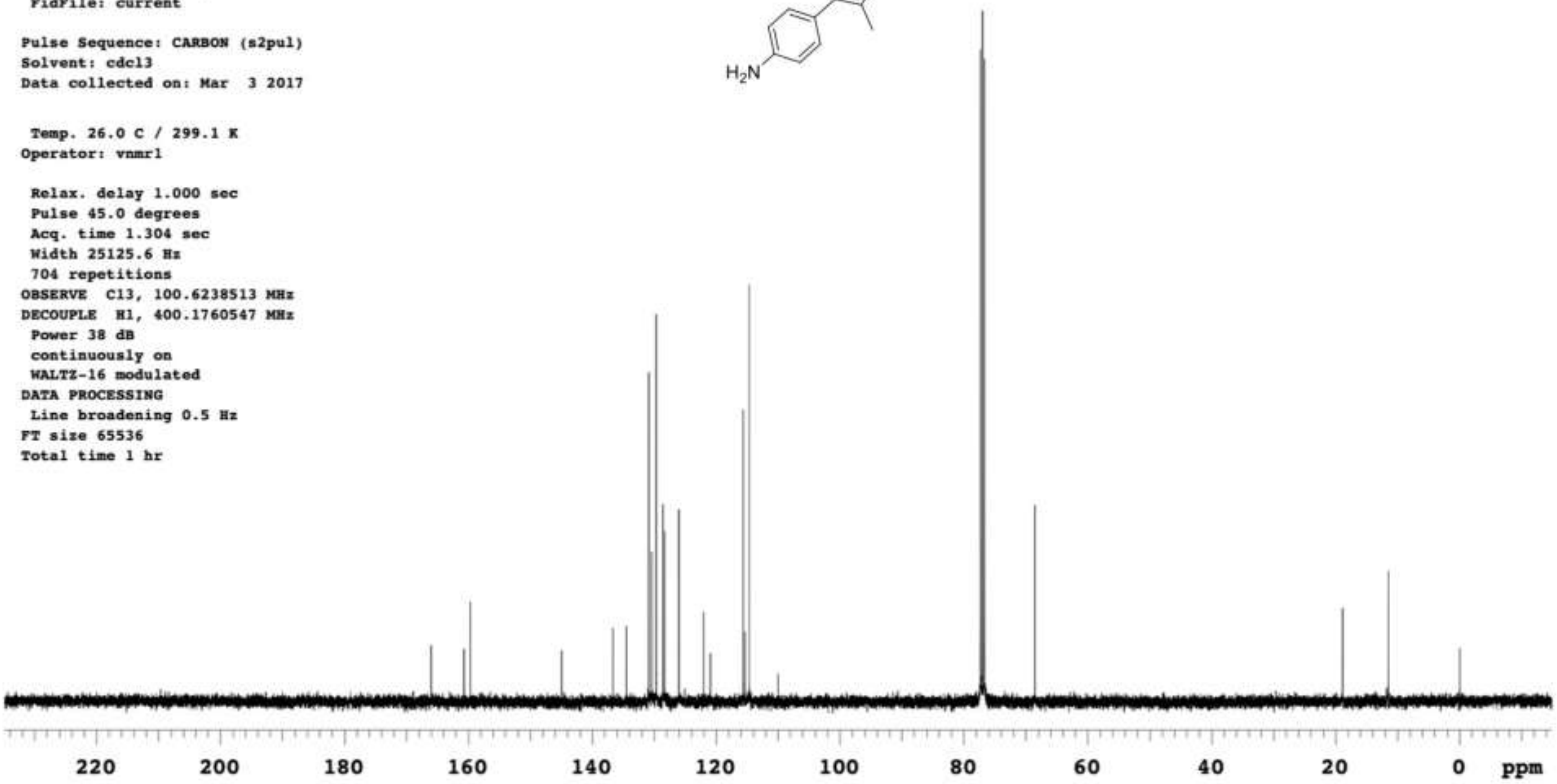

Figure S45. ^{1}H-NMR and ^{13}C-NMR spectrum of Compound **52**

Compound 52

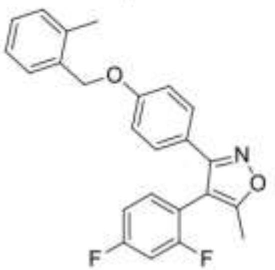

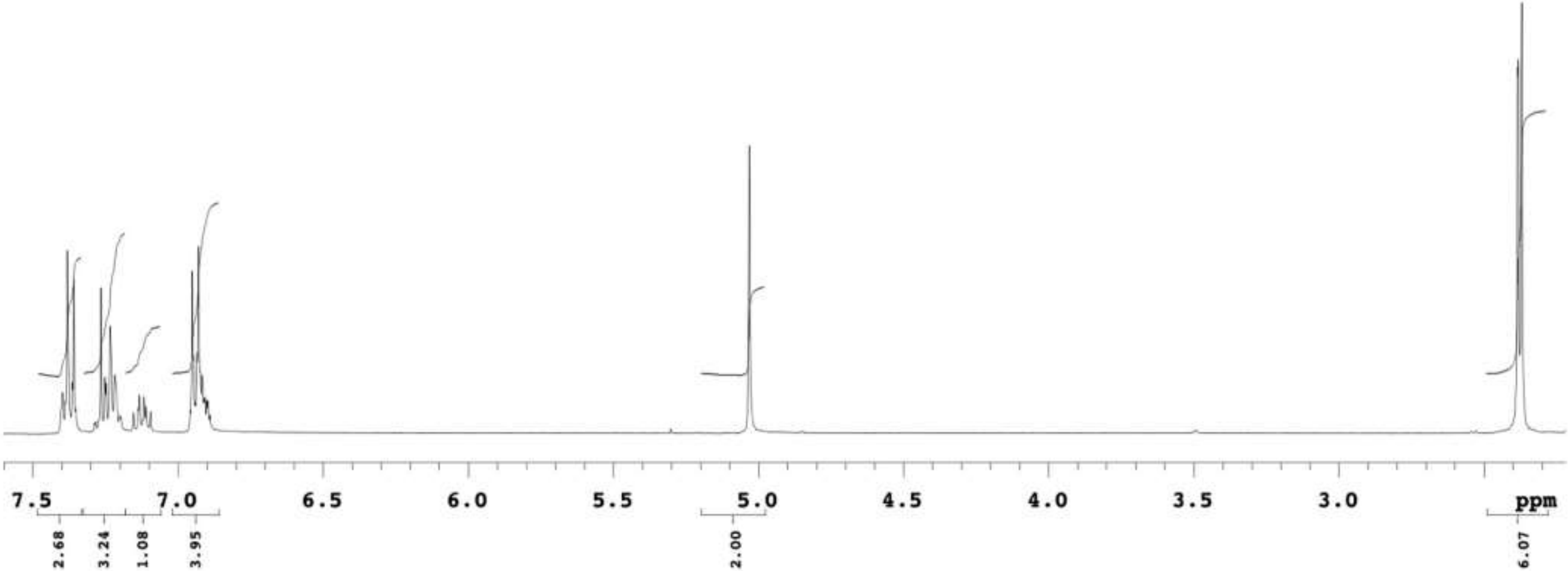

SMY155

Sample Name:
SMY155
Data Collected on:
mercury400-mercury400
Archive directory:
/home/vnmr1/vnmrsys/data
Sample directory:
SMY155_20161214_01
FidFile: current

Pulse Sequence: CARBON (s2pul)
Solvent: cdcl3
Data collected on: Dec 14 2016

Temp. 25.0 C / 298.1 K
Operator: vnmr1

Relax. delay 1.000 sec
Pulse 45.0 degrees
Acq. time 1.550 sec
Width 21141.6 Hz
64 repetitions
OBSERVE C13, 100.6238513 MHz
DECOUPLE H1, 400.1760547 MHz
Power 38 dB
continuously on
WALTZ-16 modulated
DATA PROCESSING
Line broadening 0.5 Hz
FT size 65536
Total time 3 hr, 40 min

Compound 52

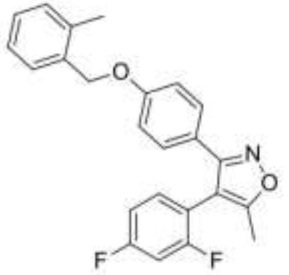

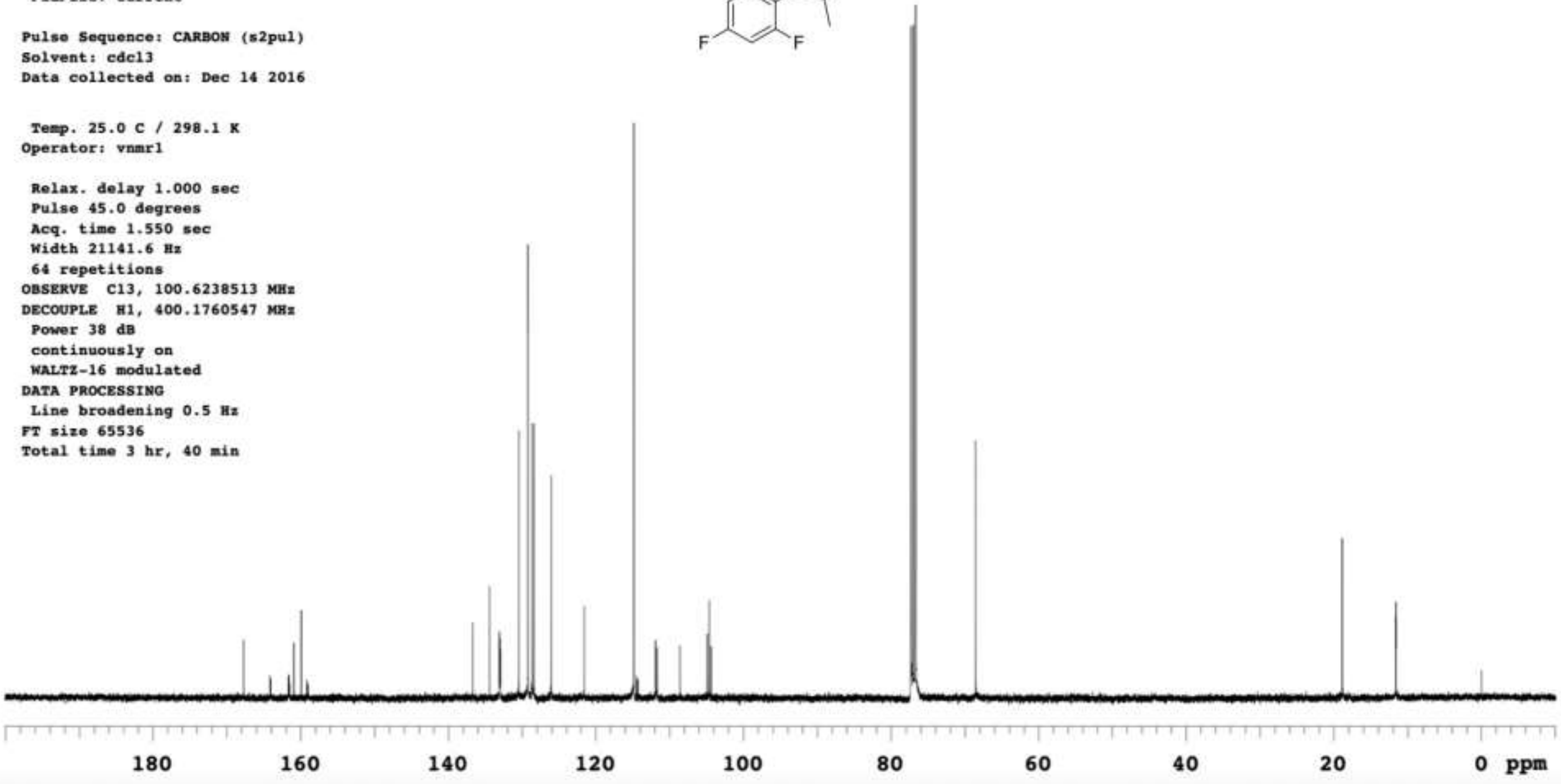

Figure S46. ^{1}H-NMR and ^{13}C-NMR spectrum of Compound **53**

Compound 53

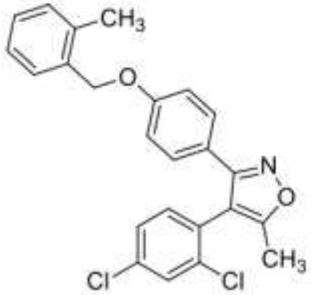

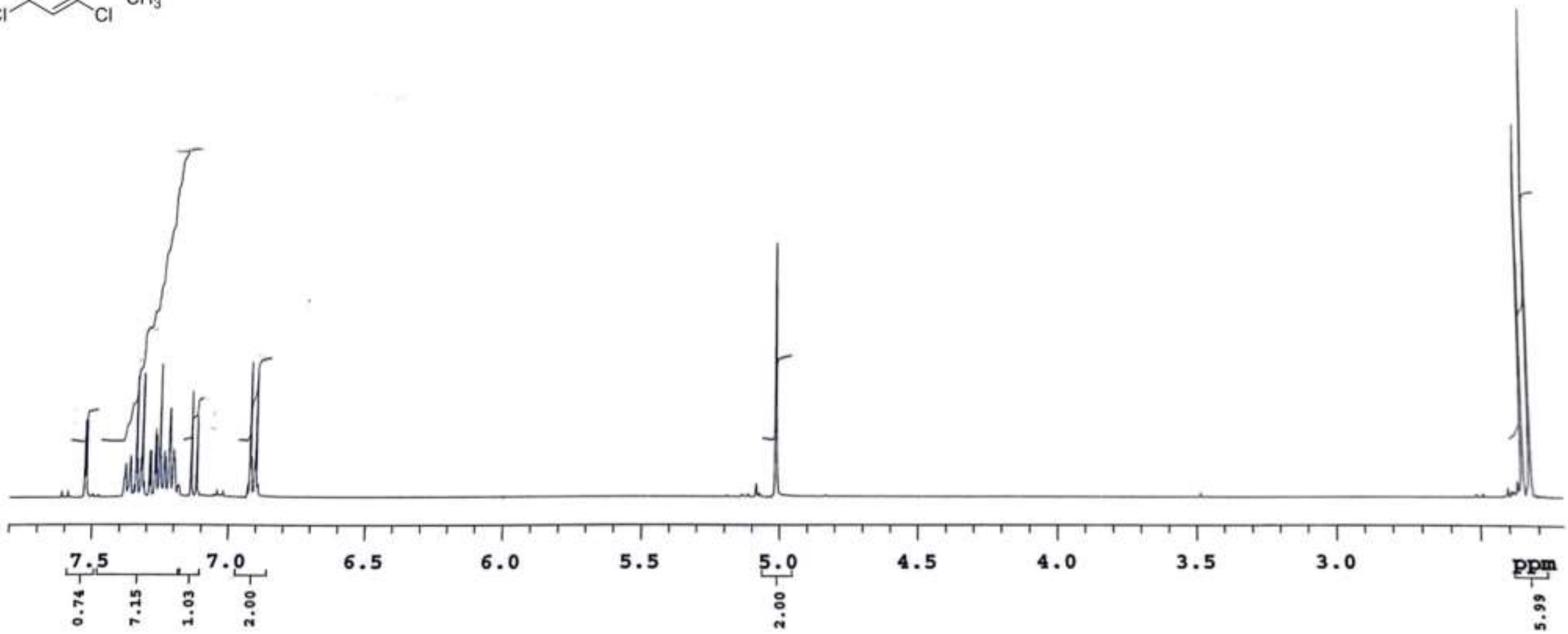

SMY134

Sample Name:
SMY134
Data Collected on:
mercury400-mercury400
Archive directory:
/home/vnmr1/vnmrsys/data
Sample directory:
SMY134_20160826_01
FidFile: current

Pulse Sequence: CARBON (s2pul)
Solvent: cdcl3
Data collected on: Aug 26 2016

Temp. 25.0 C / 298.1 K
Operator: vnmr1

Relax. delay 1.000 sec
Pulse 45.0 degrees
Acq. time 1.550 sec
Width 21141.6 Hz
256 repetitions
OBSERVE C13, 100.6238513 MHz
DECOUPLE H1, 400.1760547 MHz
Power 38 dB
continuously on
WALTZ-16 modulated
DATA PROCESSING
Line broadening 0.5 Hz
FT size 65536
Total time 55 min

Compound 53

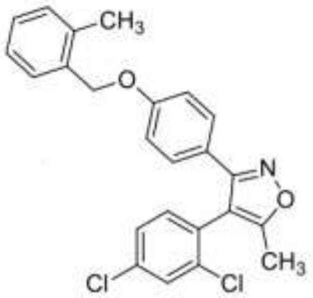

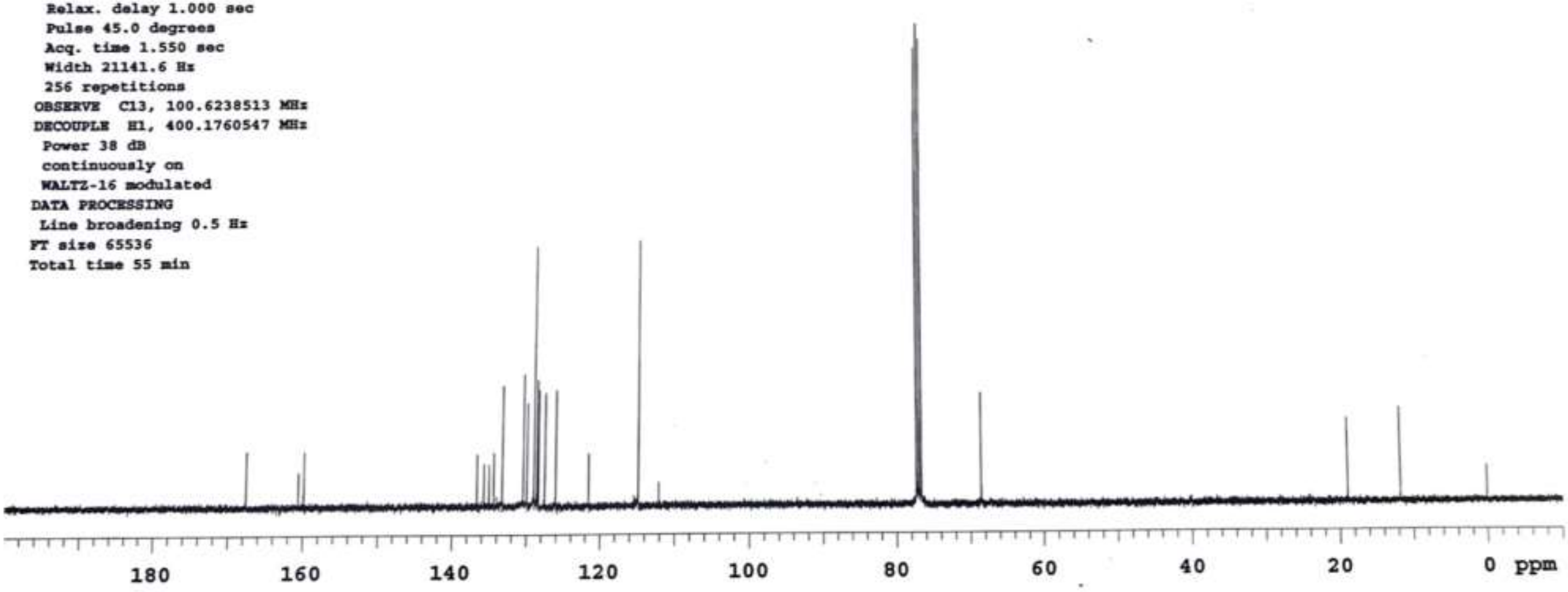

Figure S47. ^{1}H-NMR and ^{13}C-NMR spectrum of Compound **54**

Compound 54

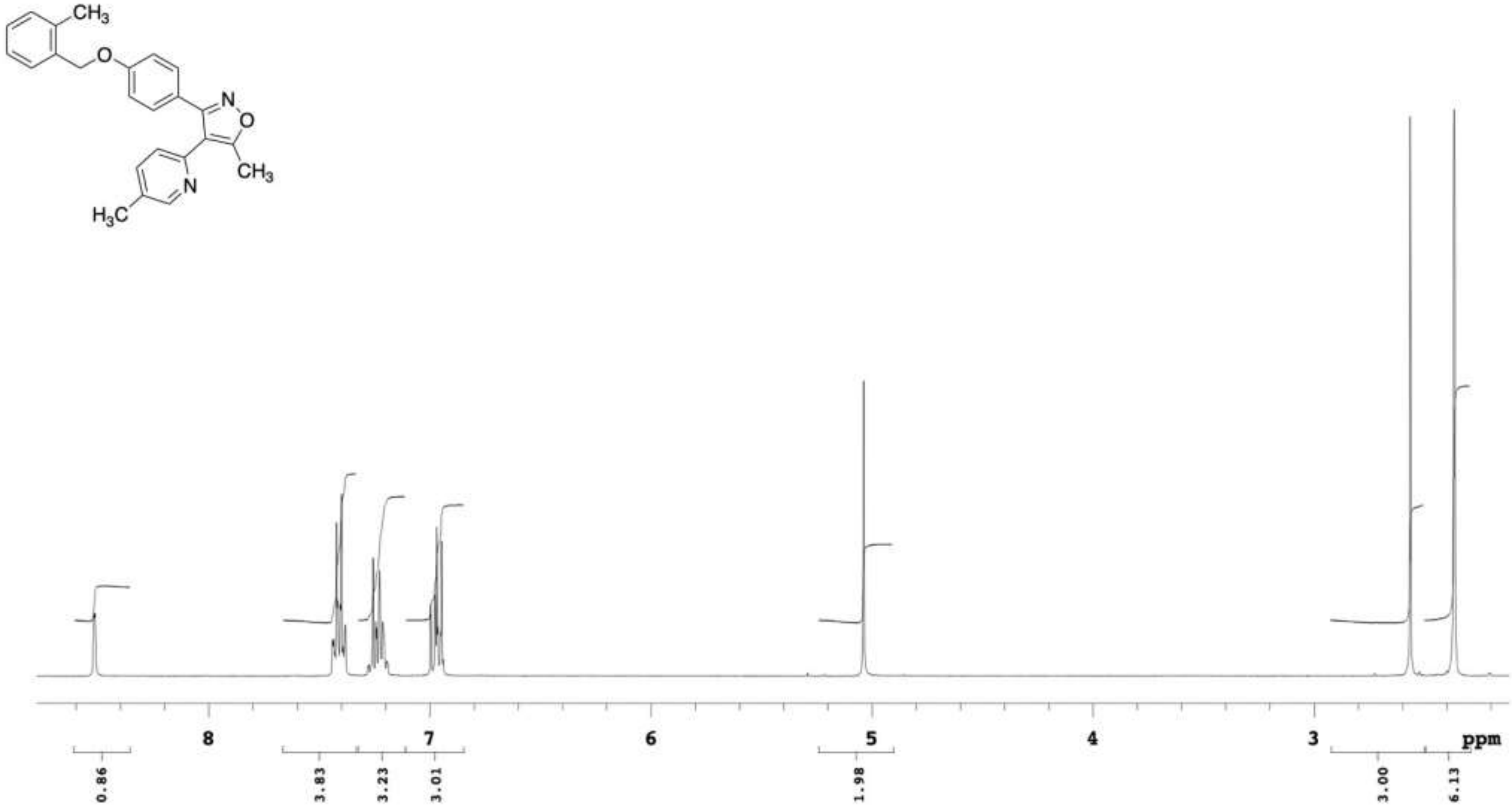

DNZ146

Sample Name:
DNZ146
Data Collected on:
mercury400-mercury400
Archive directory:
/home/vnmr1/vnmrsys/data
Sample directory:
DNZ146_20170601_01
FidFile: CARBON_01

Pulse Sequence: CARBON (s2pul)
Solvent: cdcl3
Data collected on: Jun 1 2017

Temp. 25.0 C / 298.1 K
Operator: vnmr1

Relax. delay 1.000 sec
Pulse 45.0 degrees
Acq. time 1.550 sec
Width 21141.6 Hz
1000 repetitions
OBSERVE C13, 100.6238513 MHz
DECOUPLE H1, 400.1760547 MHz
Power 38 dB
continuously on
WALTZ-16 modulated
DATA PROCESSING
Line broadening 0.5 Hz
FT size 65536
Total time 44 min

Compound 54

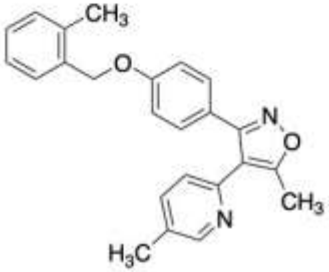

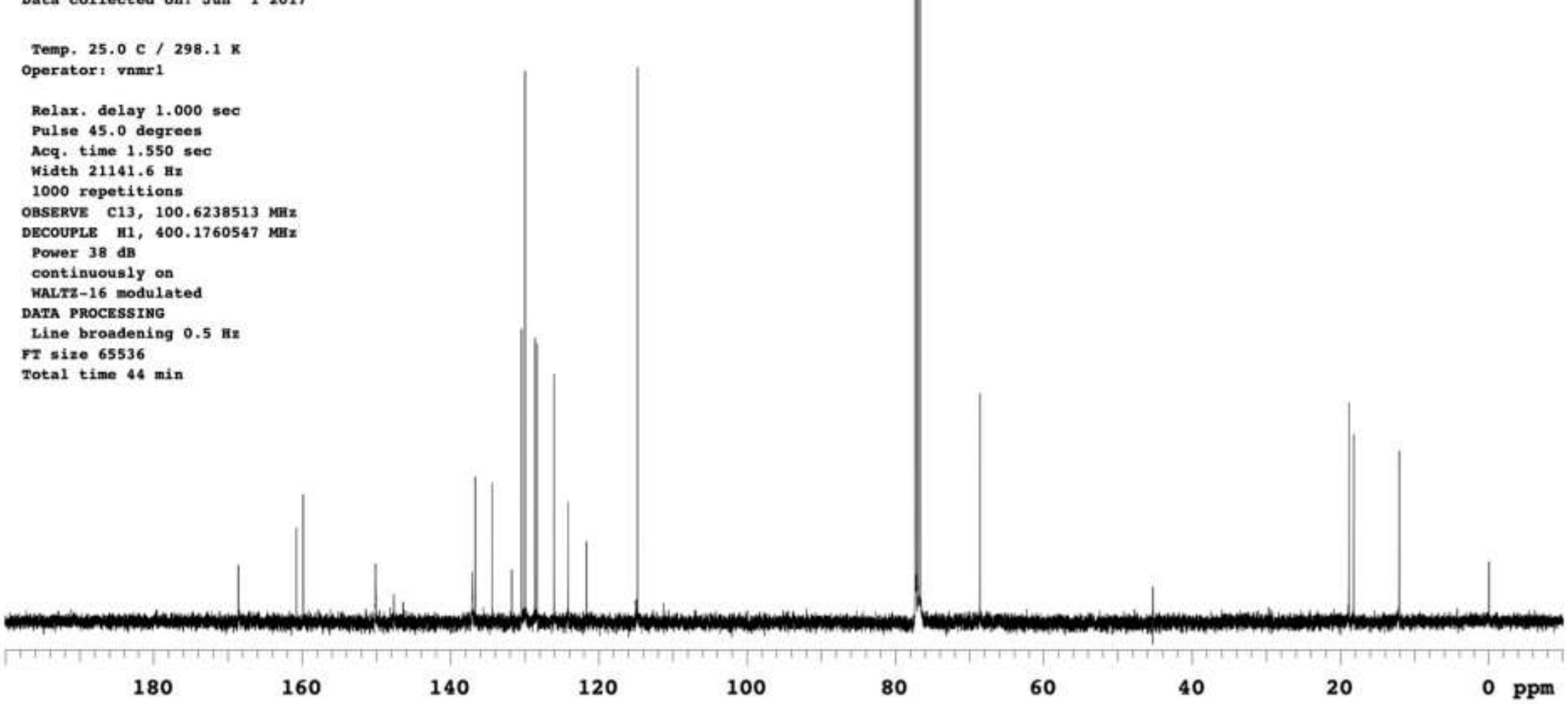

Figure S48. ^{1}H-NMR and ^{13}C-NMR spectrum of Compound **55**

Compound 55

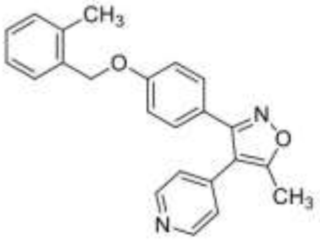

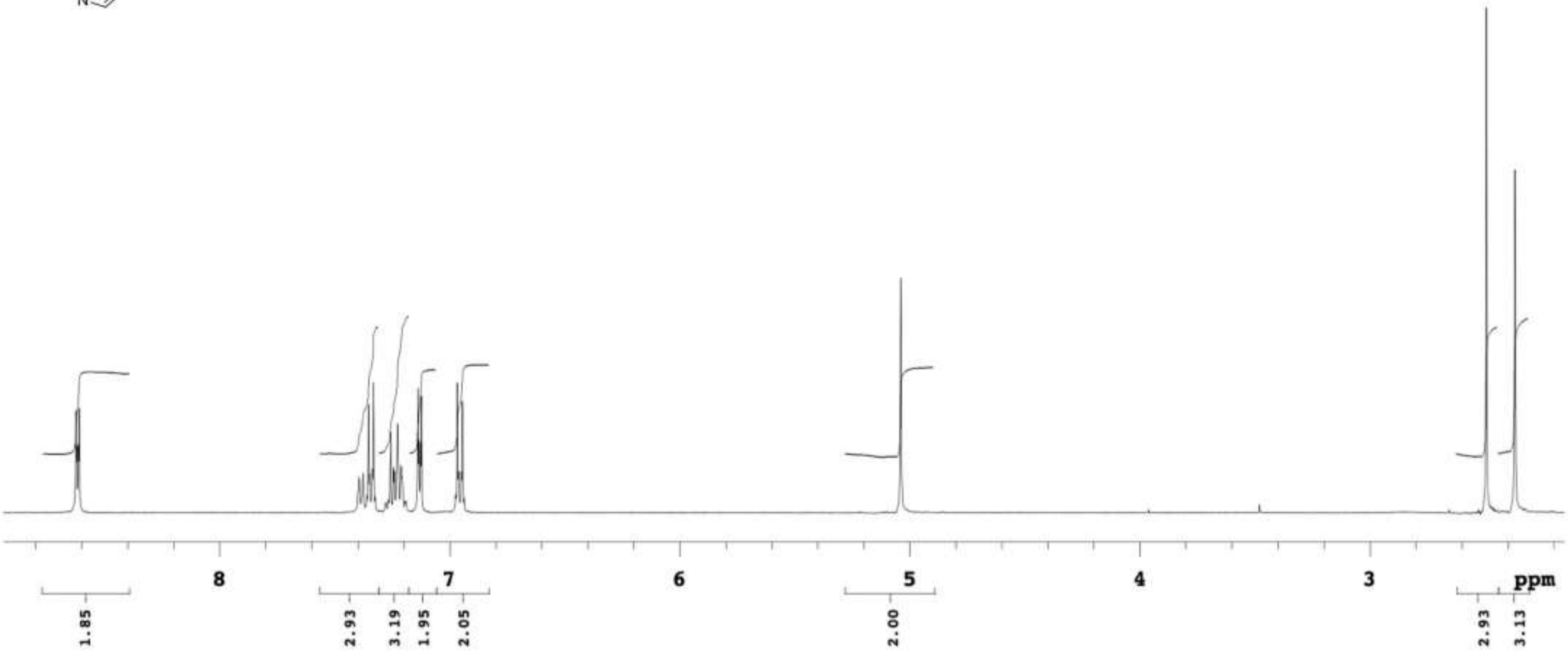

SMY-149

Sample Name:
SMY-149
Data Collected on:
mercury400-mercury400
Archive directory:
/home/vnmr1/vnmrsys/data
Sample directory:
SMY-149_20171214_01
FidFile: CARBON_01

Pulse Sequence: CARBON (s2pul)
Solvent: cdcl3
Data collected on: Dec 14 2017

Temp. 25.0 C / 298.1 K
Operator: vnmr1

Relax. delay 1.000 sec
Pulse 45.0 degrees
Acq. time 1.550 sec
Width 21141.6 Hz
1000 repetitions
OBSERVE C13, 100.6238513 MHz
DECOUPLE H1, 400.1760547 MHz
Power 38 dB
continuously on
WALTZ-16 modulated
DATA PROCESSING
Line broadening 0.5 Hz
FT size 65536
Total time 44 min

Compound 55

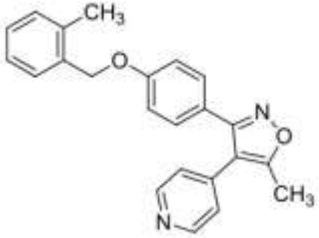

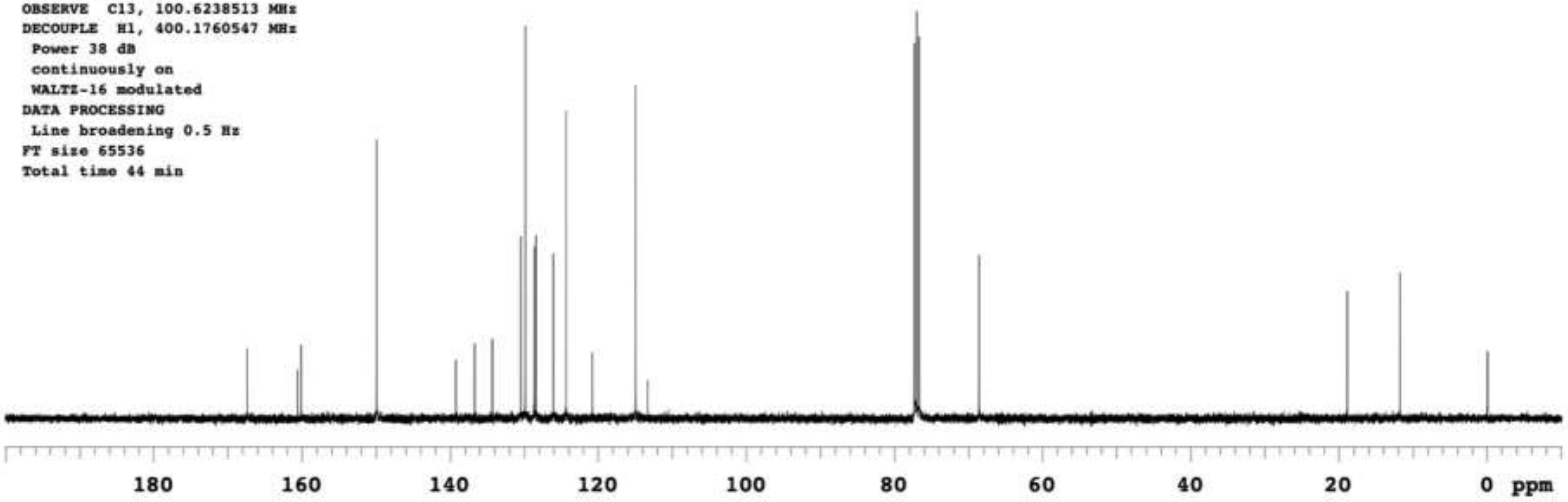

Figure S49. ^{1}H-NMR and ^{13}C-NMR spectrum of Compound **56**

Compound 56

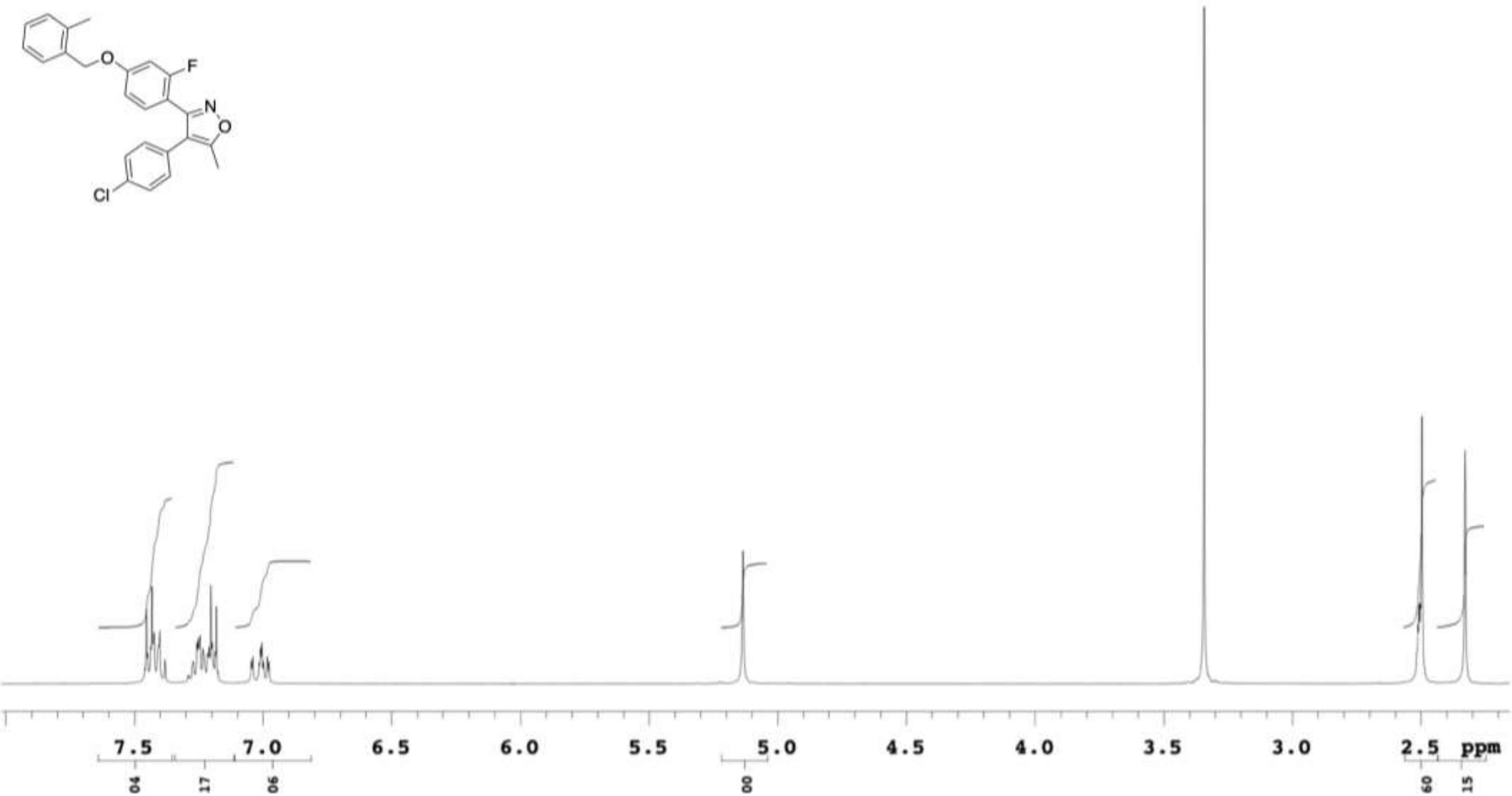

DNZ131

Sample Name:
DNZ131
Data Collected on:
mercury400-mercury400
Archive directory:
/home/vnmr1/vnmrsys/data
Sample directory:
DNZ131_20170322_01
FidFile: current

Pulse Sequence: CARBON (s2pul)
Solvent: dmso
Data collected on: Mar 22 2017

Temp. 25.0 C / 298.1 K
Operator: vnmr1

Relax. delay 1.000 sec
Pulse 45.0 degrees
Acq. time 1.304 sec
Width 25125.6 Hz
832 repetitions
OBSERVE C13, 100.6243774 MHz
DECOUPLE H1, 400.1779555 MHz
Power 38 dB
continuously on
WALTZ-16 modulated
DATA PROCESSING
Line broadening 0.5 Hz
FT size 65536
Total time 1 hr, 20 min

Compound 56

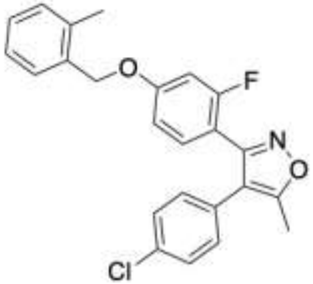

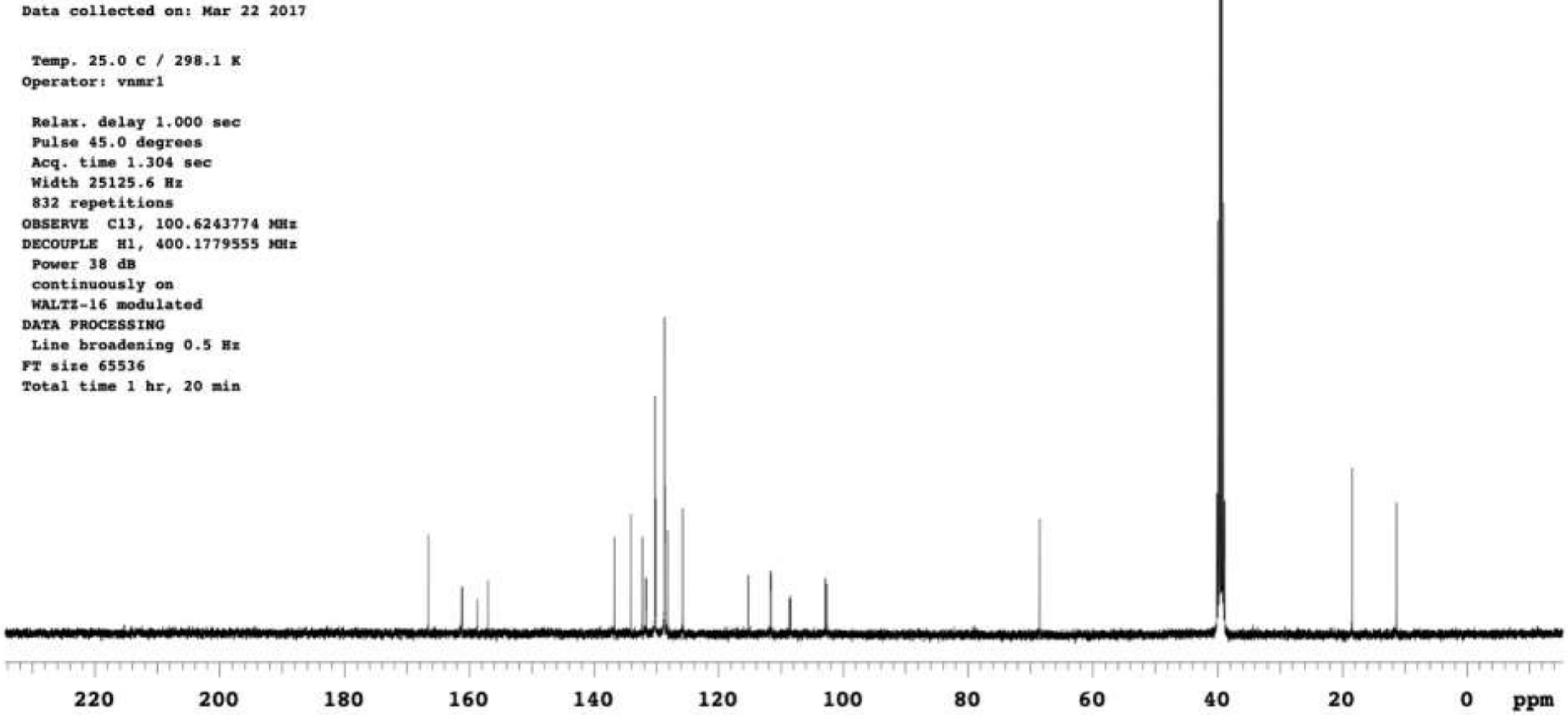

Figure S50. ^{1}H-NMR and ^{13}C-NMR spectrum of Compound **57**

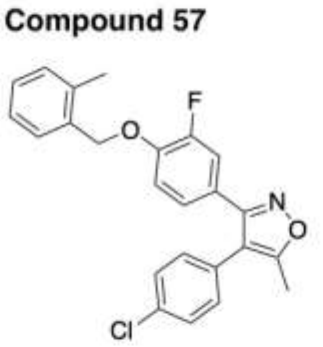

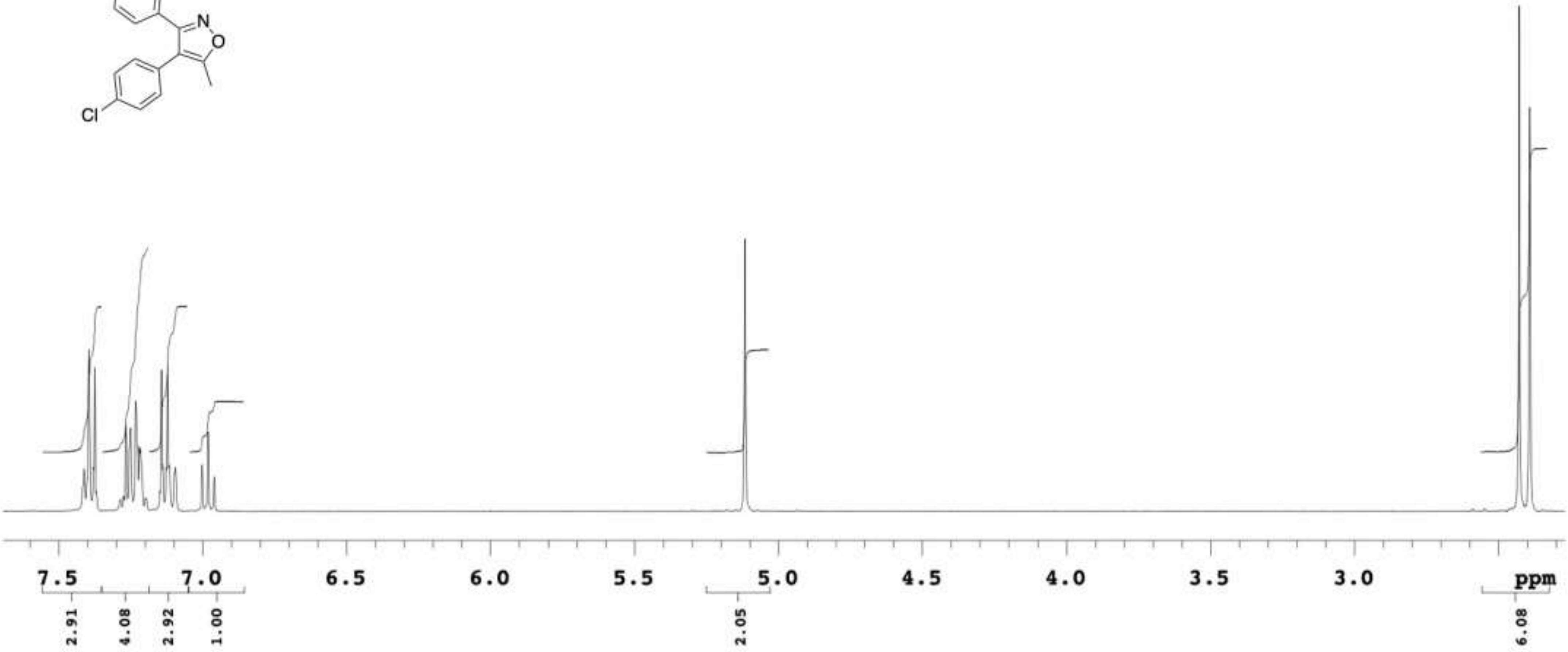

DNZ120

Sample Name:
DNZ120
Data Collected on:
mercury400-mercury400
Archive directory:
/home/vnmr1/vnmrsys/data
Sample directory:
DNZ120_20170303_01
FidFile: CARBON_01

Pulse Sequence: CARBON (s2pul)
Solvent: cdcl3
Data collected on: Mar 3 2017

Temp. 25.0 C / 298.1 K
Operator: vnmr1

Relax. delay 1.000 sec
Pulse 45.0 degrees
Acq. time 1.550 sec
Width 21141.6 Hz
5000 repetitions
OBSERVE C13, 100.6238513 MHz
DECOUPLE H1, 400.1760547 MHz
Power 38 dB
continuously on
WALTZ-16 modulated
DATA PROCESSING
Line broadening 0.5 Hz
FT size 65536
Total time 3 hr, 40 min

Compound 57

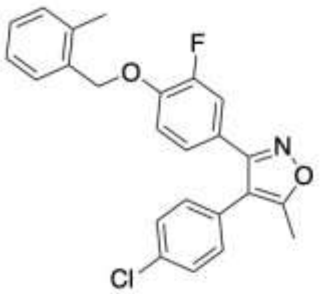

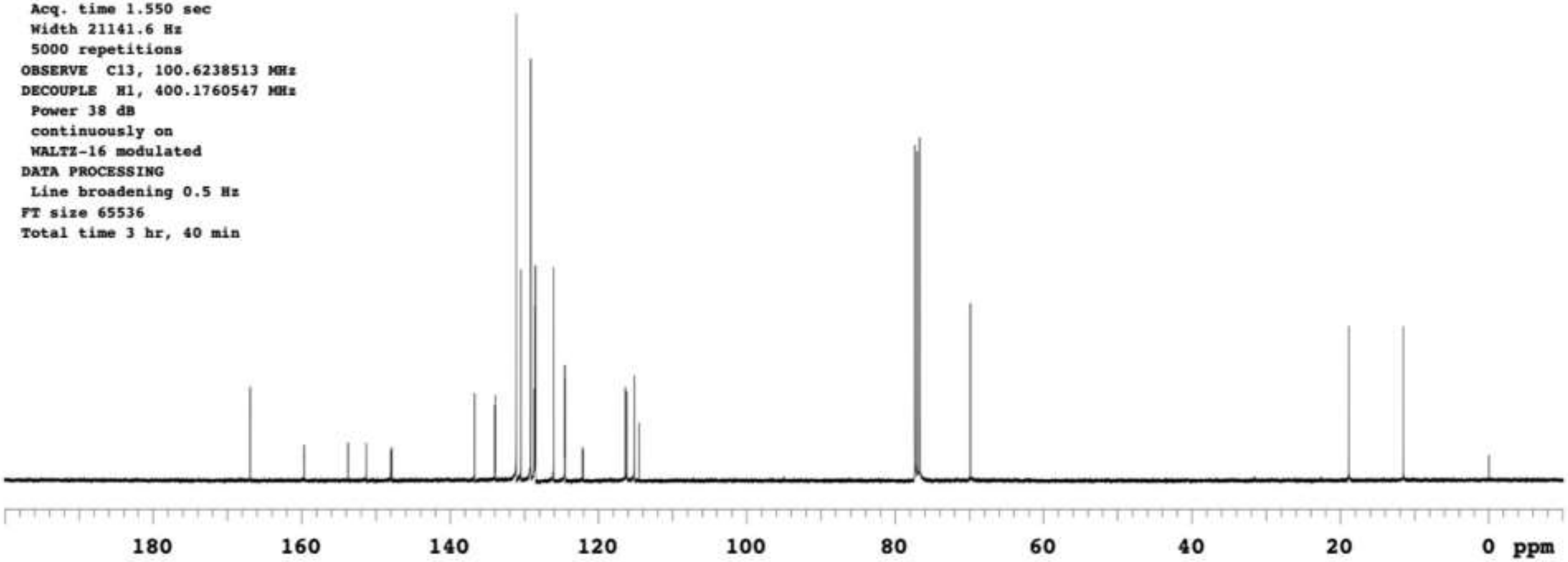

Figure S51. ^{1}H-NMR and ^{13}C-NMR spectrum of Compound **59**

Compound 59

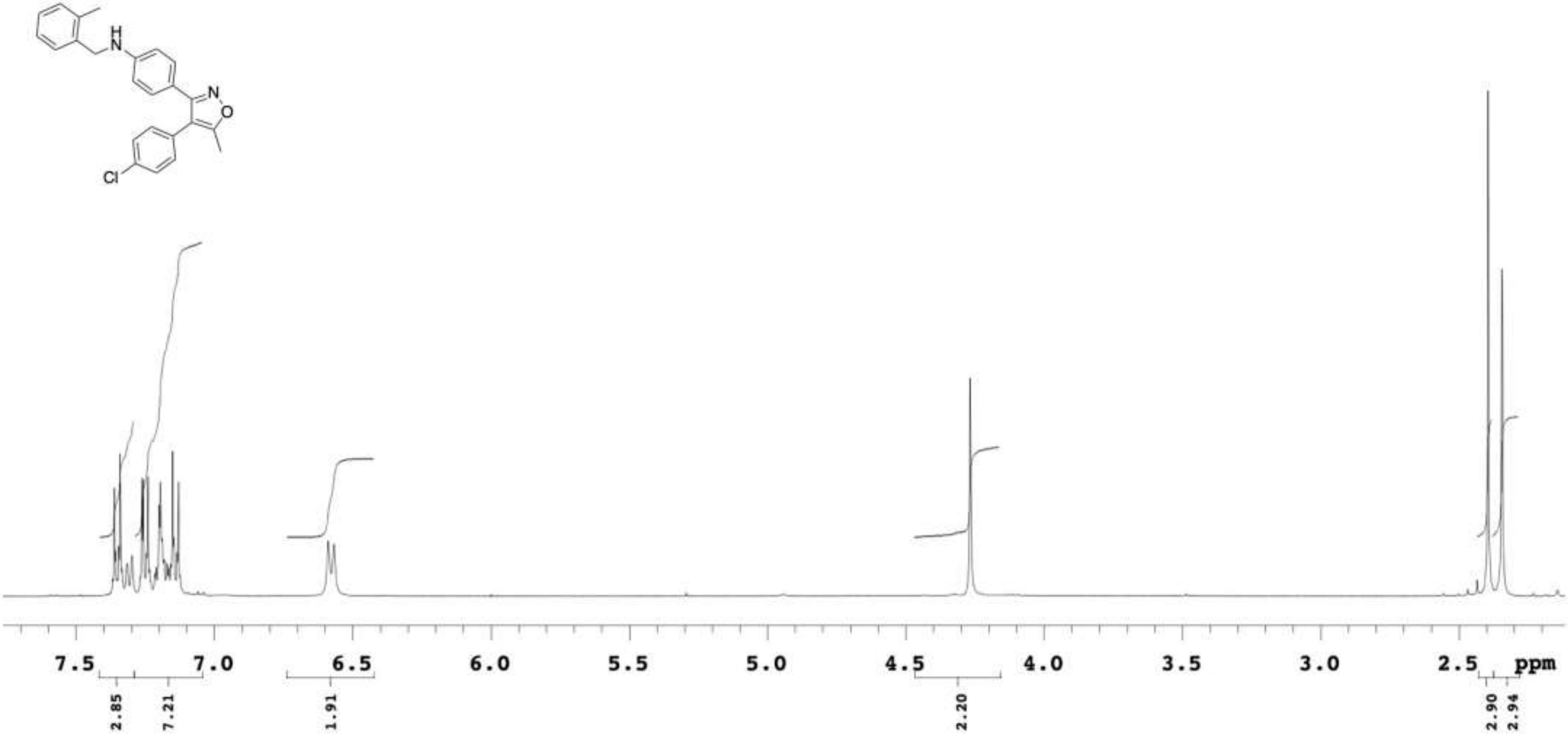

DNZ162

Sample Name:
DNZ162
Data Collected on:
mercury400-mercury400
Archive directory:
/home/vnmr1/vnmrsys/data
Sample directory:
DNZ162_20170601_01
FidFile: CARBON_02

Pulse Sequence: CARBON (s2pul)
Solvent: cdcl3
Data collected on: Jun 1 2017

Temp. 25.0 C / 298.1 K
Operator: vnmr1

Relax. delay 1.000 sec
Pulse 45.0 degrees
Acq. time 1.550 sec
Width 21141.6 Hz
2256 repetitions
OBSERVE C13, 100.6238513 MHz
DECOUPLE H1, 400.1760547 MHz
Power 38 dB
continuously on
WALTZ-16 modulated
DATA PROCESSING
Line broadening 0.5 Hz
FT size 65536
Total time 1 hr, 39 min

Compound 59

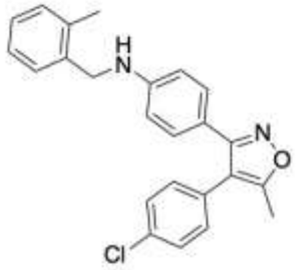

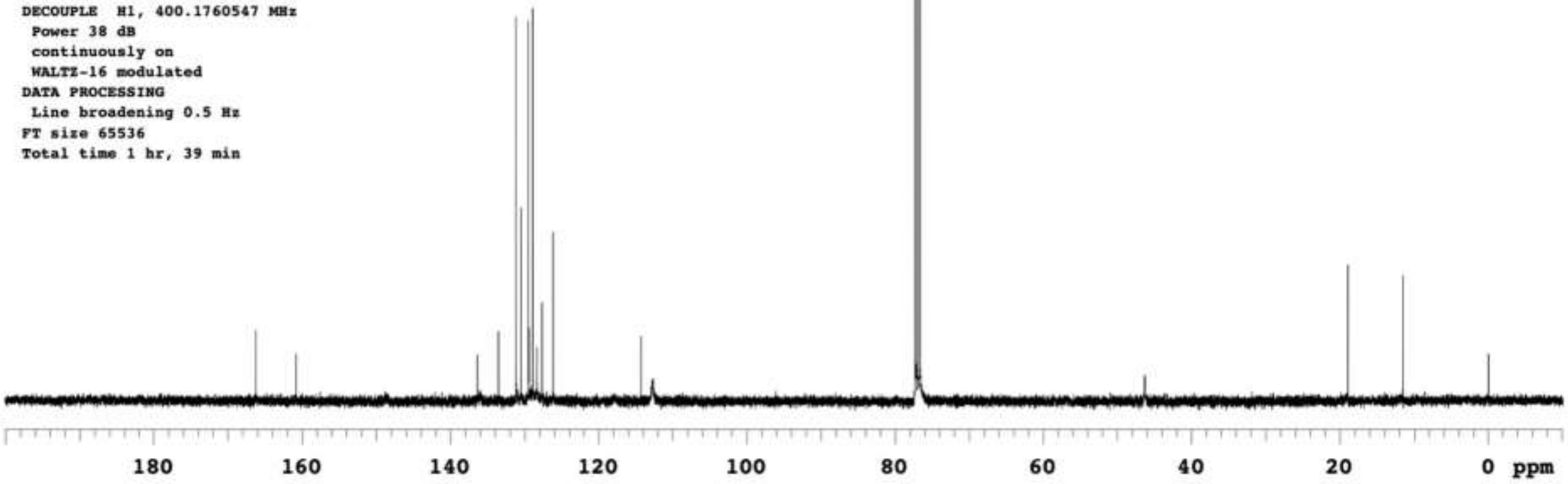

Figure S52. ^{1}H-NMR and ^{13}C-NMR spectrum of Compound **63**

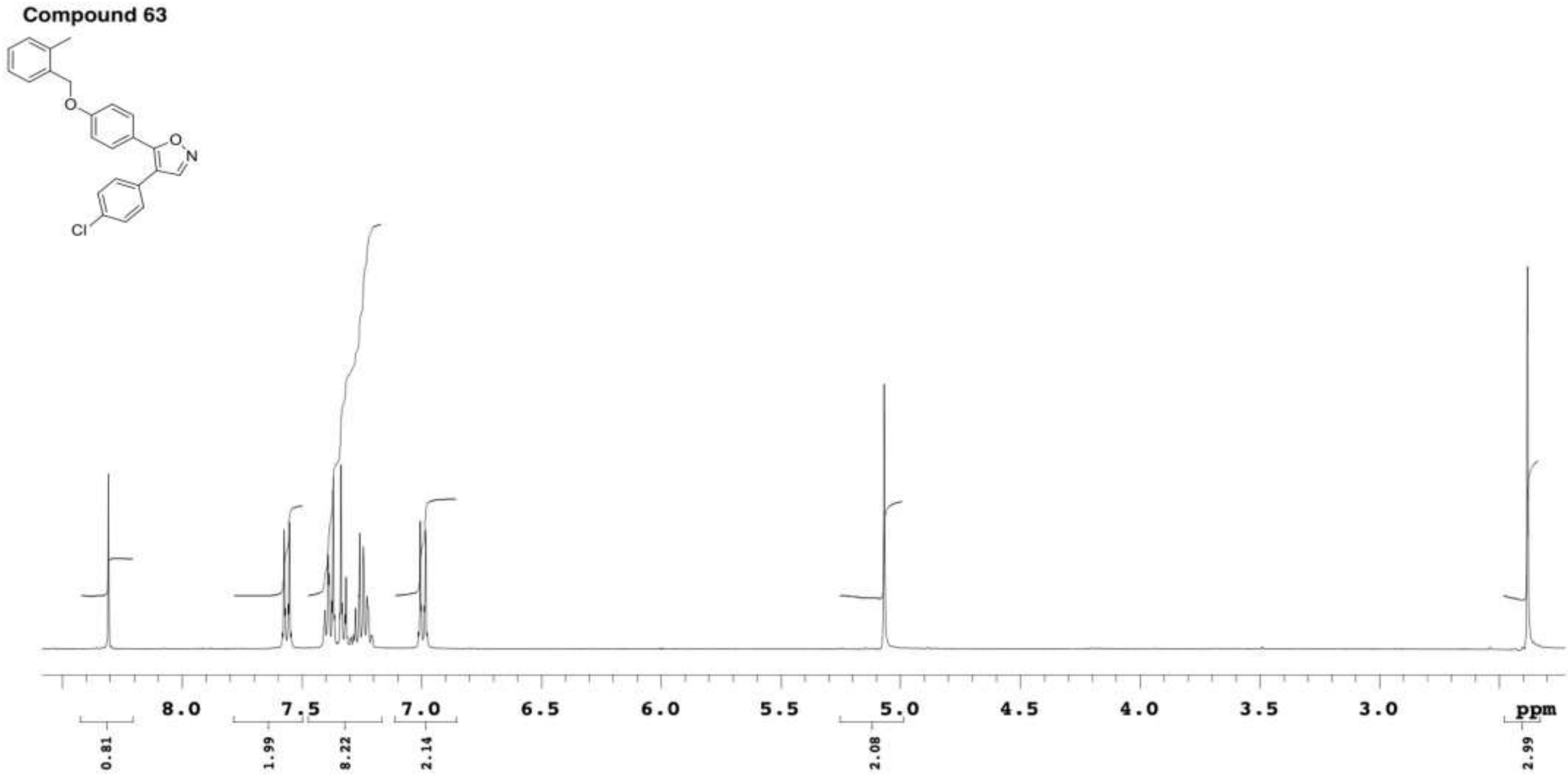

SMY180

Sample Name:
SMY180
Data Collected on:
mercury400-mercury400
Archive directory:
/home/vnmr1/vnmrsys/data
Sample directory:
SMY180_20170111_01
FidFile: current

Pulse Sequence: CARBON (s2pul)
Solvent: cdcl3
Data collected on: Jan 11 2017

Temp. 25.0 C / 298.1 K
Operator: vnmr1

Relax. delay 1.000 sec
Pulse 45.0 degrees
Acq. time 1.304 sec
Width 25125.6 Hz
1024 repetitions
OBSERVE C13, 100.6238513 MHz
DECOUPLE H1, 400.1760547 MHz
Power 38 dB
continuously on
WALTZ-16 modulated
DATA PROCESSING
Line broadening 0.5 Hz
FT size 65536
Total time 1 hr

Compound 63

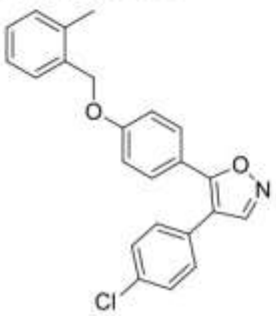

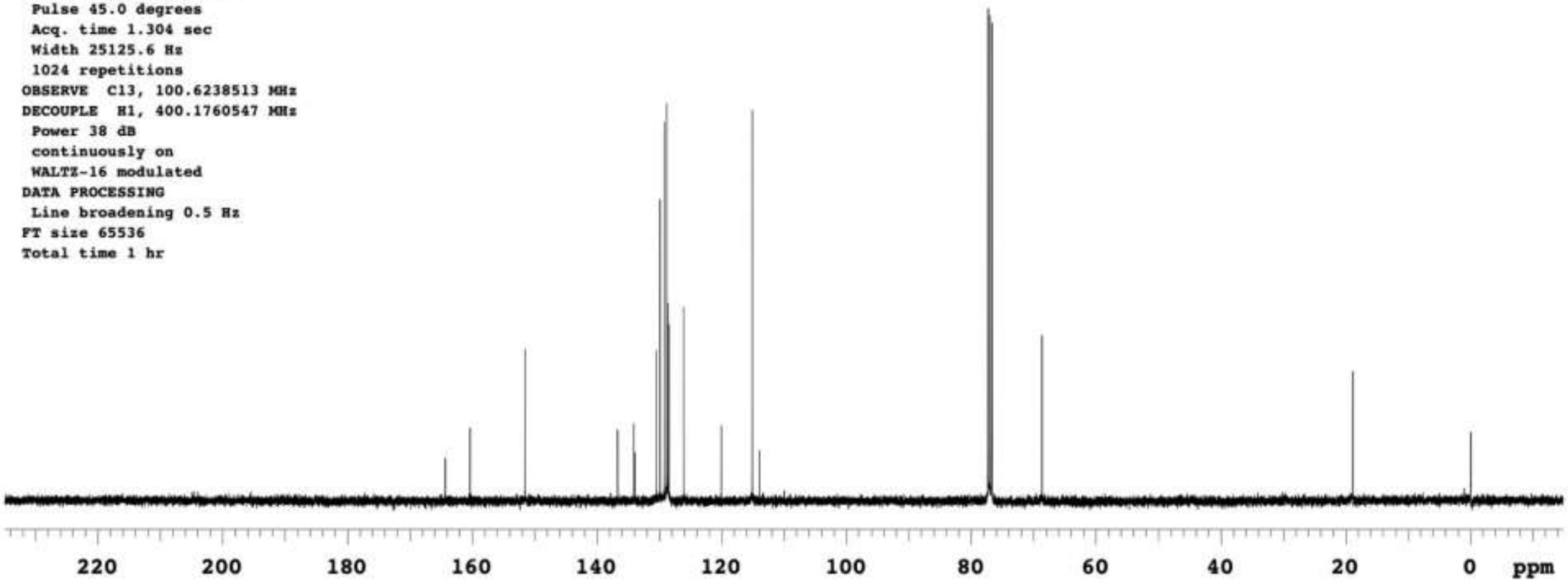

Figure S53. ^{1}H-NMR and ^{13}C-NMR spectrum of Compound **65**

Compound 65

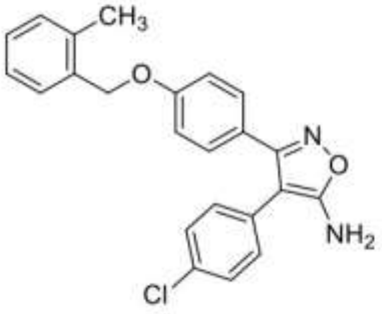

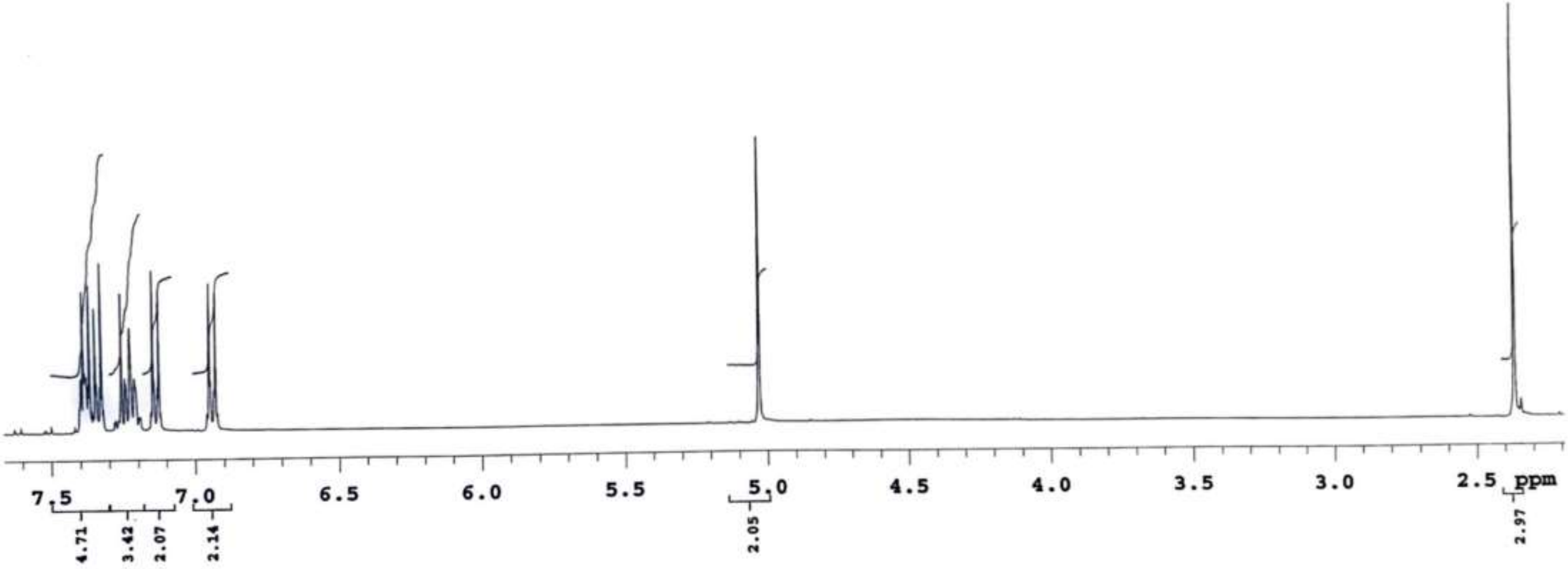

SMY-125

Sample Name:
SMY-125
Data Collected on:
mercury400-mercury400
Archive directory:
/home/vnmr1/vnmrsys/data
Sample directory:
SMY-125_20160930_01
FidFile: CARBON_01

Pulse Sequence: CARBON (s2pul)
Solvent: cdcl3
Data collected on: Sep 30 2016

Temp. 25.0 C / 298.1 K
Operator: vnmr1

Relax. delay 1.000 sec
Pulse 45.0 degrees
Acq. time 1.550 sec
Width 21141.6 Hz
2512 repetitions
OBSERVE C13, 100.6238513 MHz
DECOUPLE H1, 400.1760547 MHz
Power 38 dB
continuously on
WALTZ-16 modulated
DATA PROCESSING
Line broadening 0.5 Hz
FT size 65536
Total time 1 hr, 50 min

Compound 65

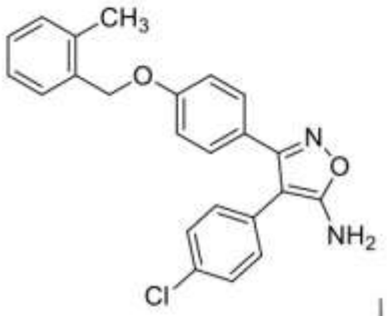

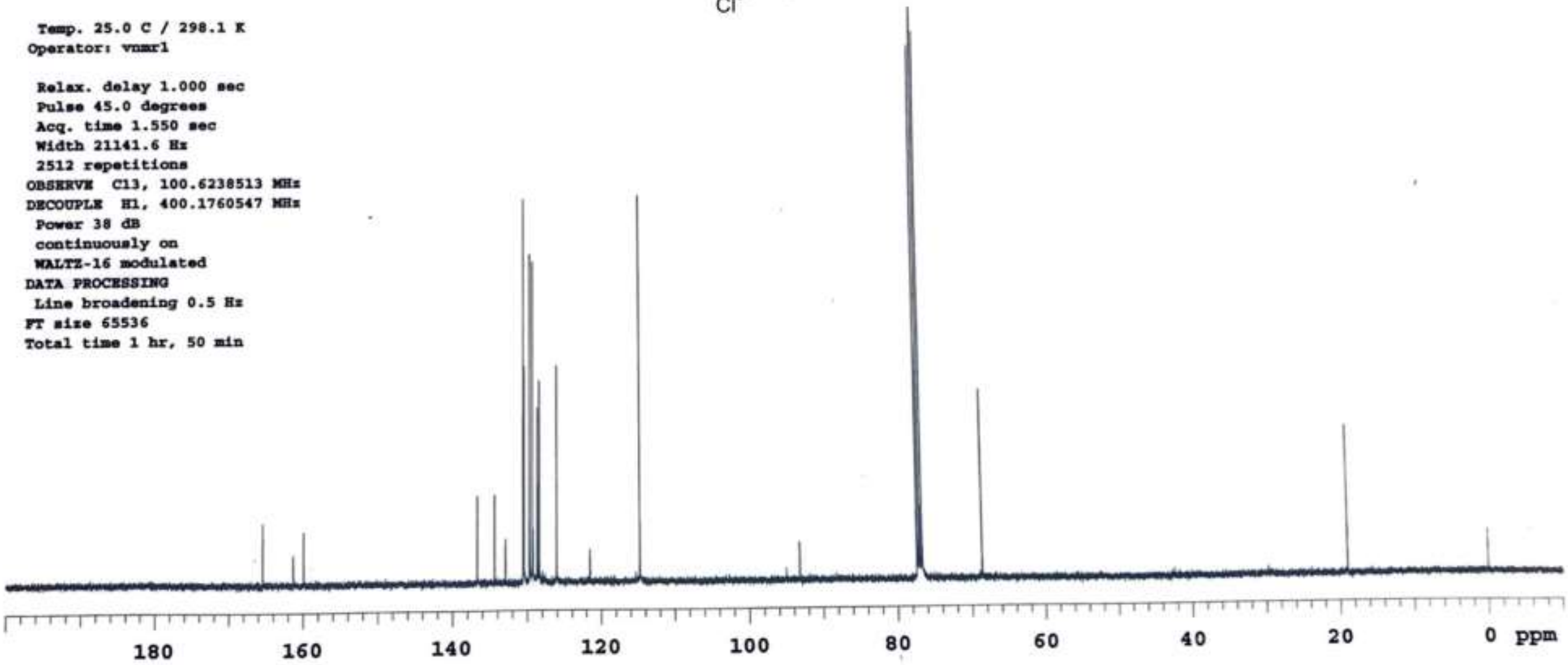

Figure S54. ^{1}H-NMR and ^{13}C-NMR spectrum of Compound **67**

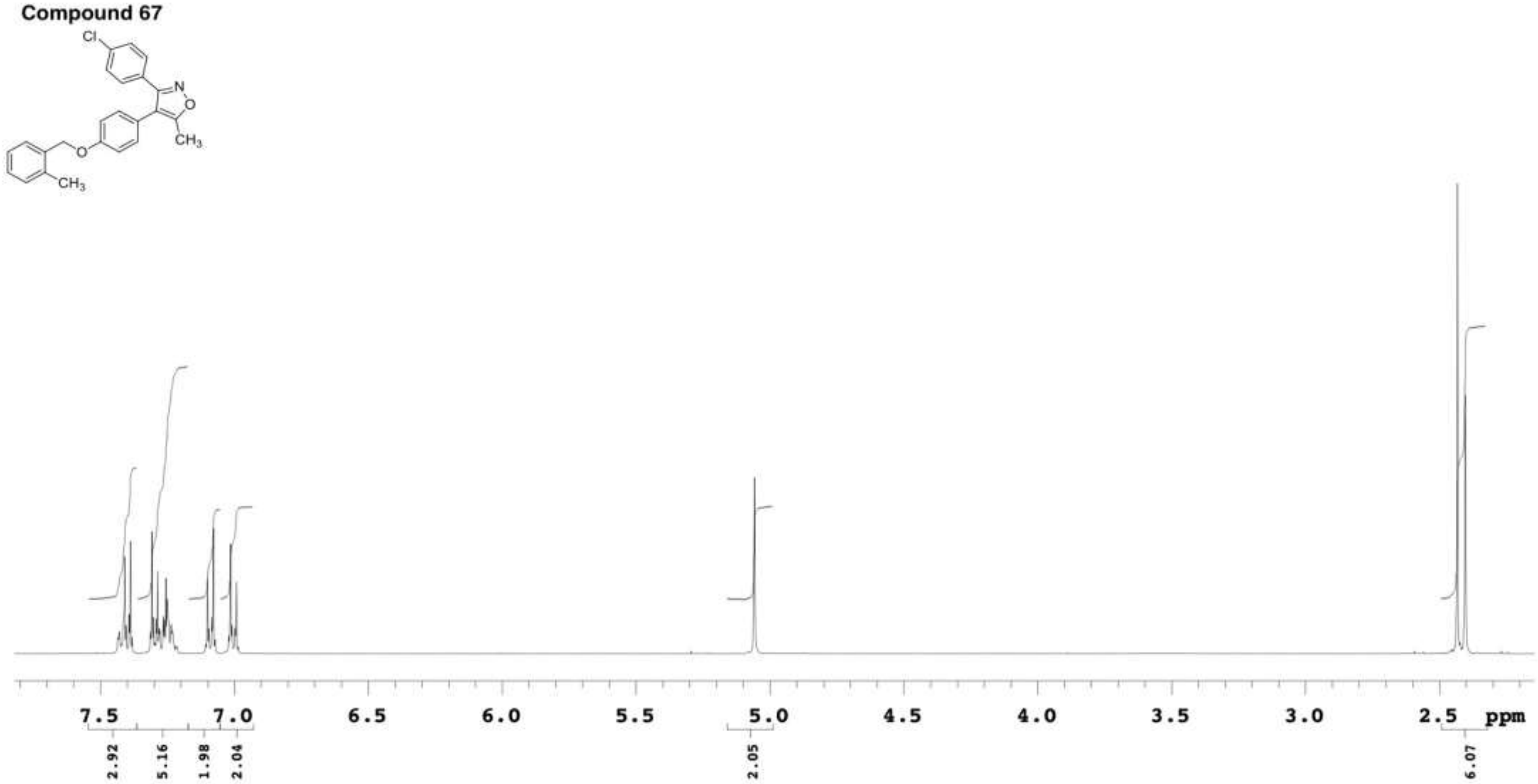

SMY235

Sample Name:
SMY235
Data Collected on:
mercury400-mercury400
Archive directory:
/home/vnmr1/vnmrsys/data
Sample directory:
SMY235_20170701_01
FidFile: current

Pulse Sequence: CARBON (s2pul)
Solvent: cdcl3
Data collected on: Jul 1 2017

Temp. 25.0 C / 298.1 K
Operator: vnmr1

Relax. delay 1.000 sec
Pulse 45.0 degrees
Acq. time 1.550 sec
Width 21141.6 Hz
1728 repetitions
OBSERVE C13, 100.6238513 MHz
DECOUPLE H1, 400.1760547 MHz
Power 38 dB
continuously on
WALTZ-16 modulated
DATA PROCESSING
Line broadening 0.5 Hz
FT size 65536
Total time 1 hr, 28 min

Compound 67

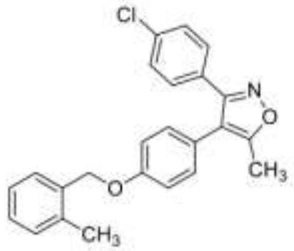

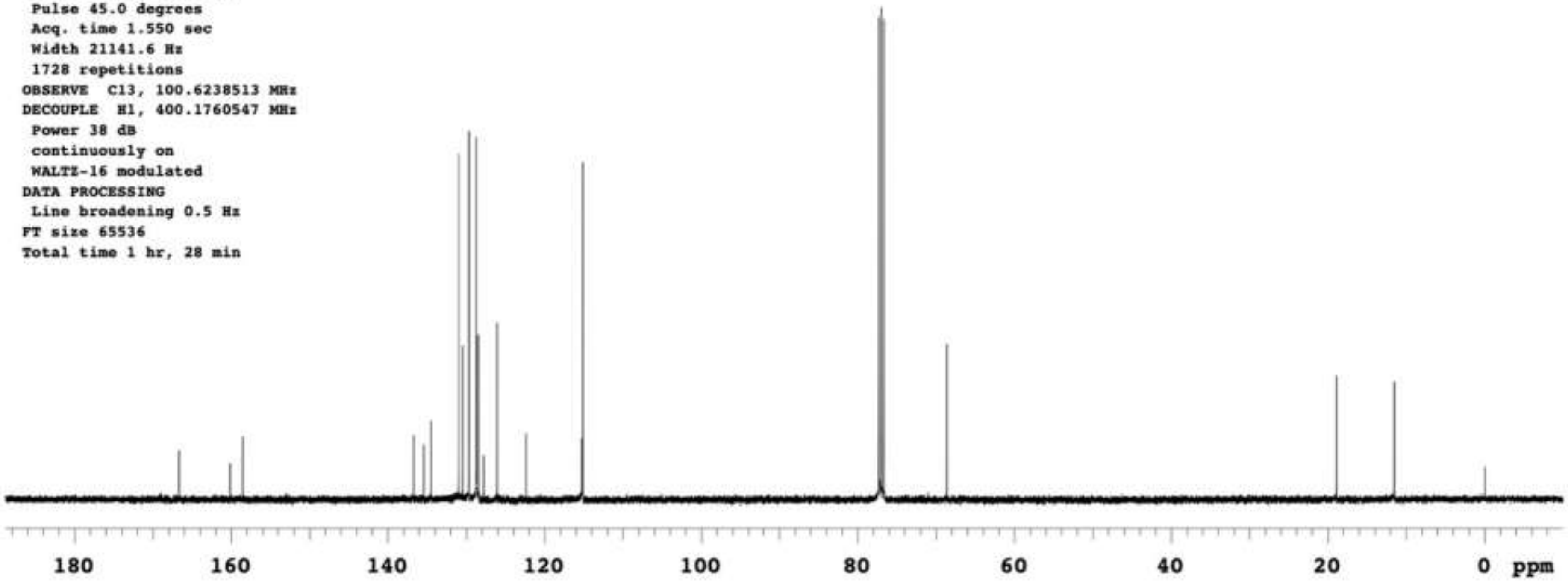

Figure S55. ^{1}H-NMR and ^{13}C-NMR spectrum of Compound **69**

Compound 69

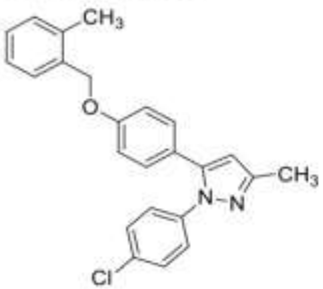

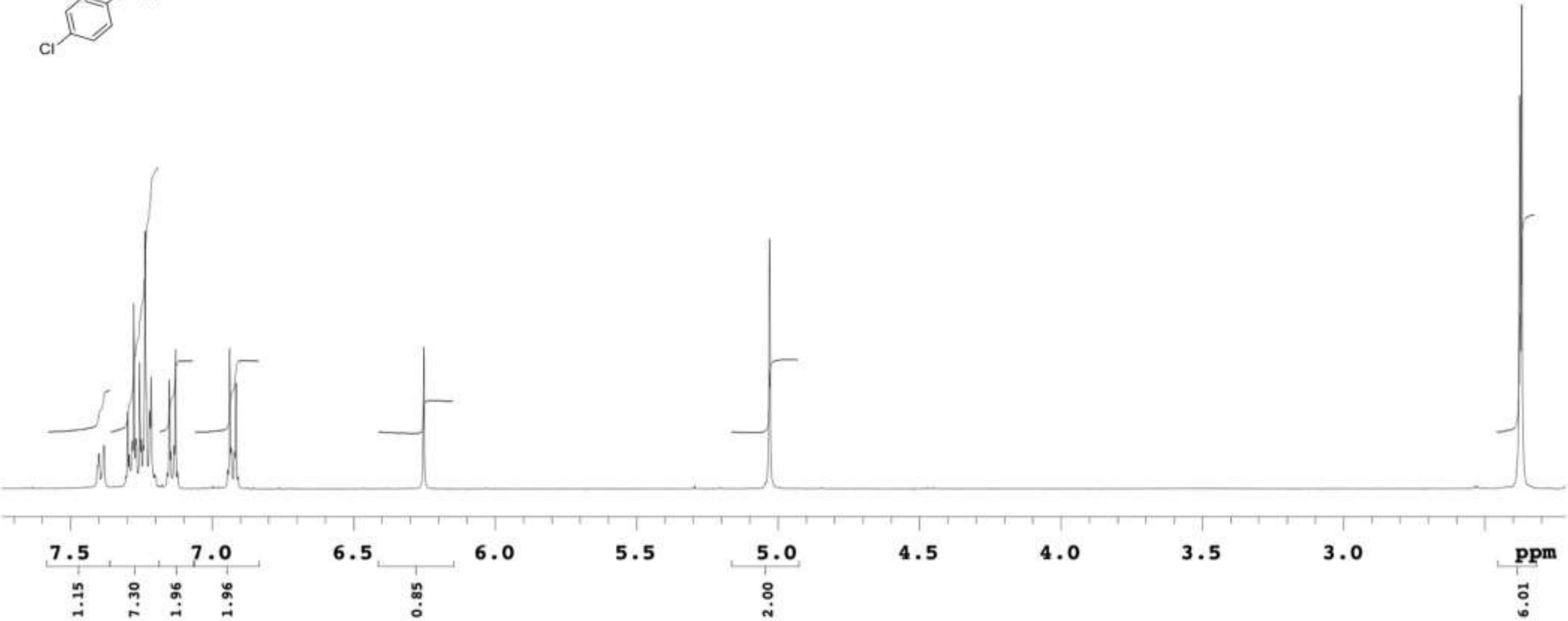

Compound 69

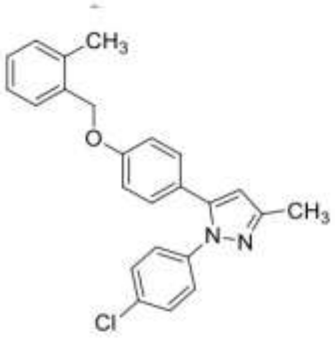
CH3
O
CH3
N
N
Cl

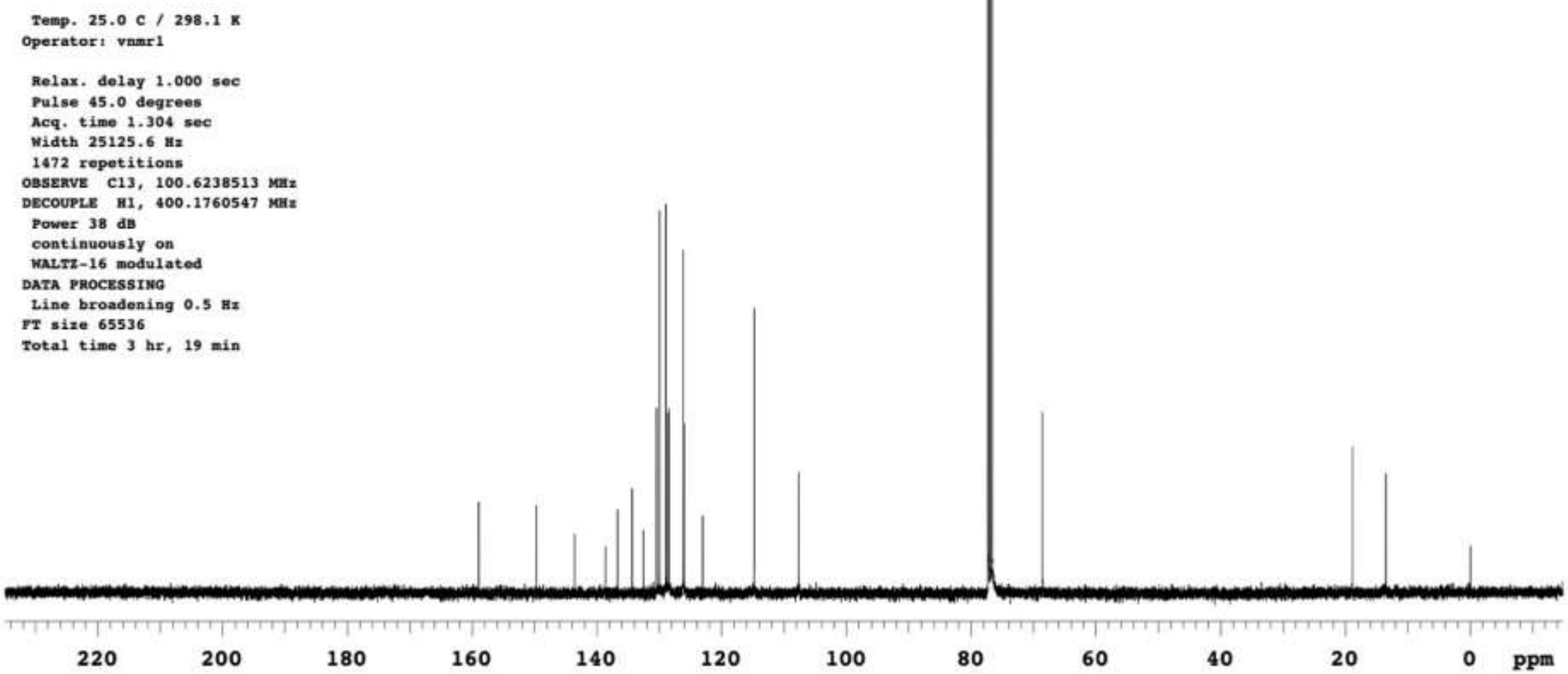
DNY942
Sample Name:
DNY942
Data Collected on:
mercury400-mercury400
Archive directory:
/home/vnmr1/vnmrsys/data
Sample directory:
DNY942_20170530_01
FidFile: current
Pulse Sequence: CARBON (s2pul)
Solvent: cdcl3
Data collected on: May 30 2017
Temp. 25.0 C / 298.1 K
Operator: vnmr1
Relax. delay 1.000 sec
Pulse 45.0 degrees
Acq. time 1.304 sec
Width 25125.6 Hz
1472 repetitions
OBSERVE C13, 100.6238513 MHz
DECOUPLE H1, 400.1760547 MHz
Power 38 dB
continuously on
WALTZ-16 modulated
DATA PROCESSING
Line broadening 0.5 Hz
FT size 65536
Total time 3 hr, 19 min
220
200
180
160
140
120
100
80
60
40
20
0
ppm

Figure S56. ^{1}H-NMR and ^{13}C-NMR spectrum of Compound **71**

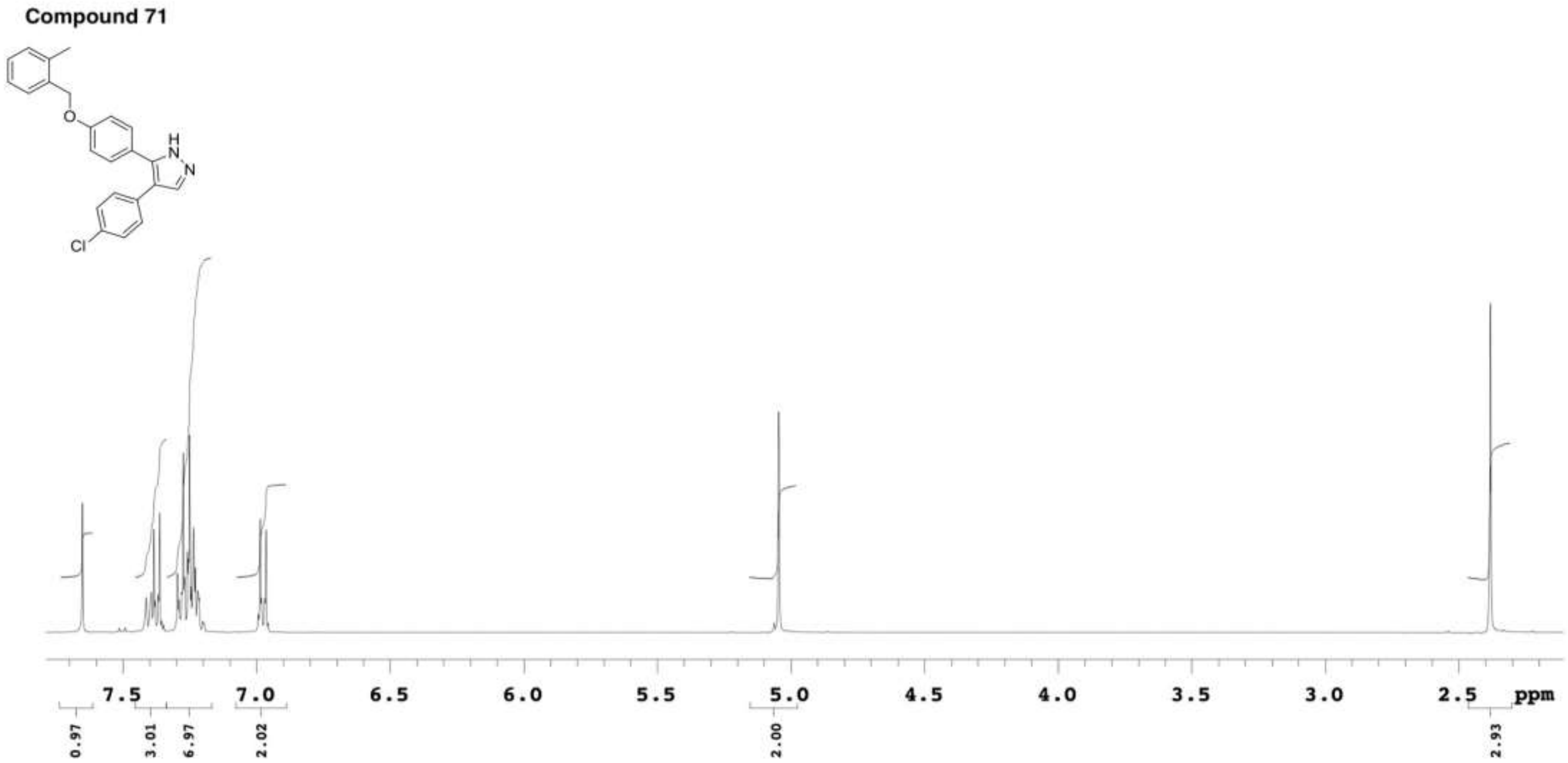

SMY-179

Sample Name:
SMY-179
Data Collected on:
mercury400-mercury400
Archive directory:
/home/vnmr1/vnmrsys/data
Sample directory:
SMY-179_20170111_01
FidFile: CARBON_01

Pulse Sequence: CARBON (s2pul)
Solvent: cdcl3
Data collected on: Jan 11 2017

Temp. 25.0 C / 298.1 K
Operator: vnmr1

Relax. delay 1.000 sec
Pulse 45.0 degrees
Acq. time 1.304 sec
Width 25125.6 Hz
1512 repetitions
OBSERVE C13, 100.6238513 MHz
DECOUPLE H1, 400.1760547 MHz
Power 38 dB
continuously on
WALTZ-16 modulated
DATA PROCESSING
Line broadening 0.5 Hz
FT size 65536
Total time 1 hr

Compound 71

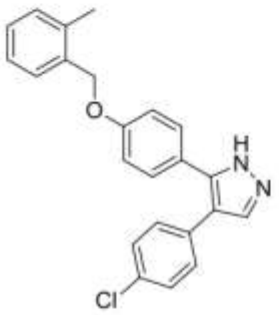

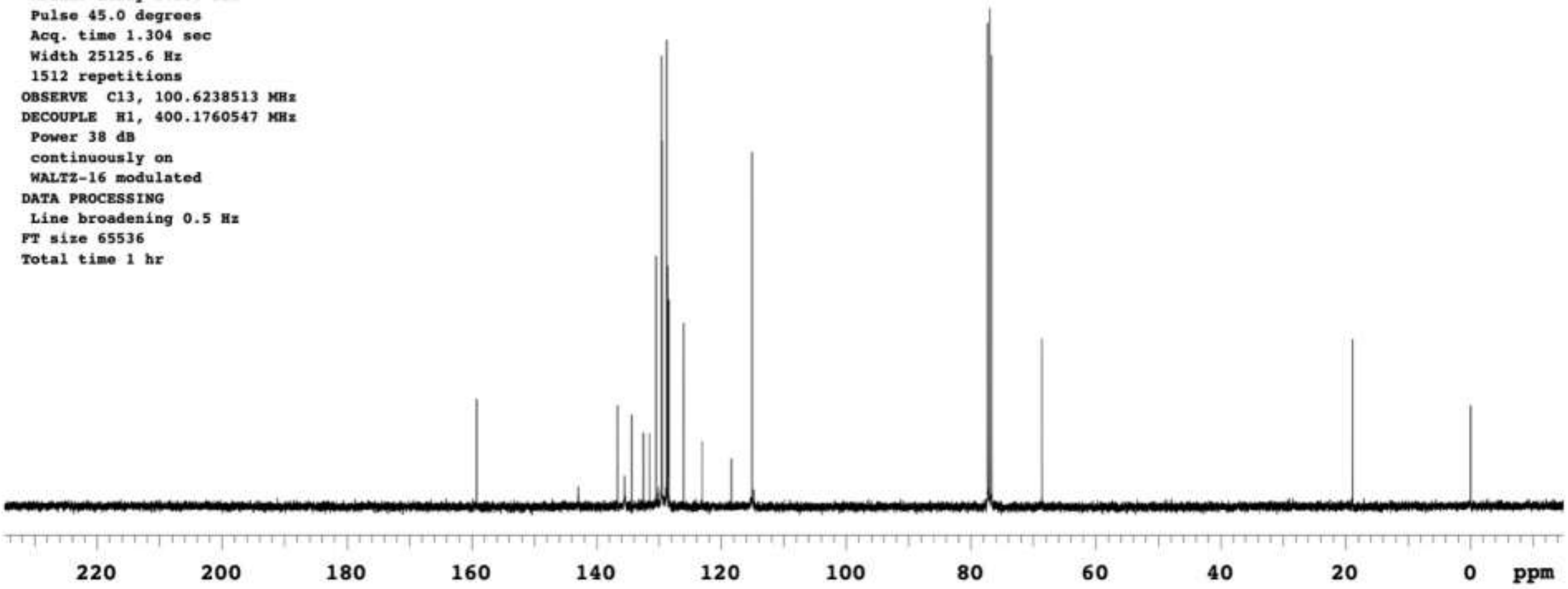

Figure S57. ^{1}H-NMR and ^{13}C-NMR spectrum of Compound **72**

Compound 72

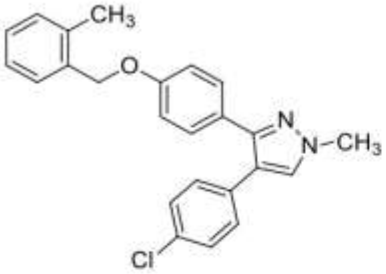

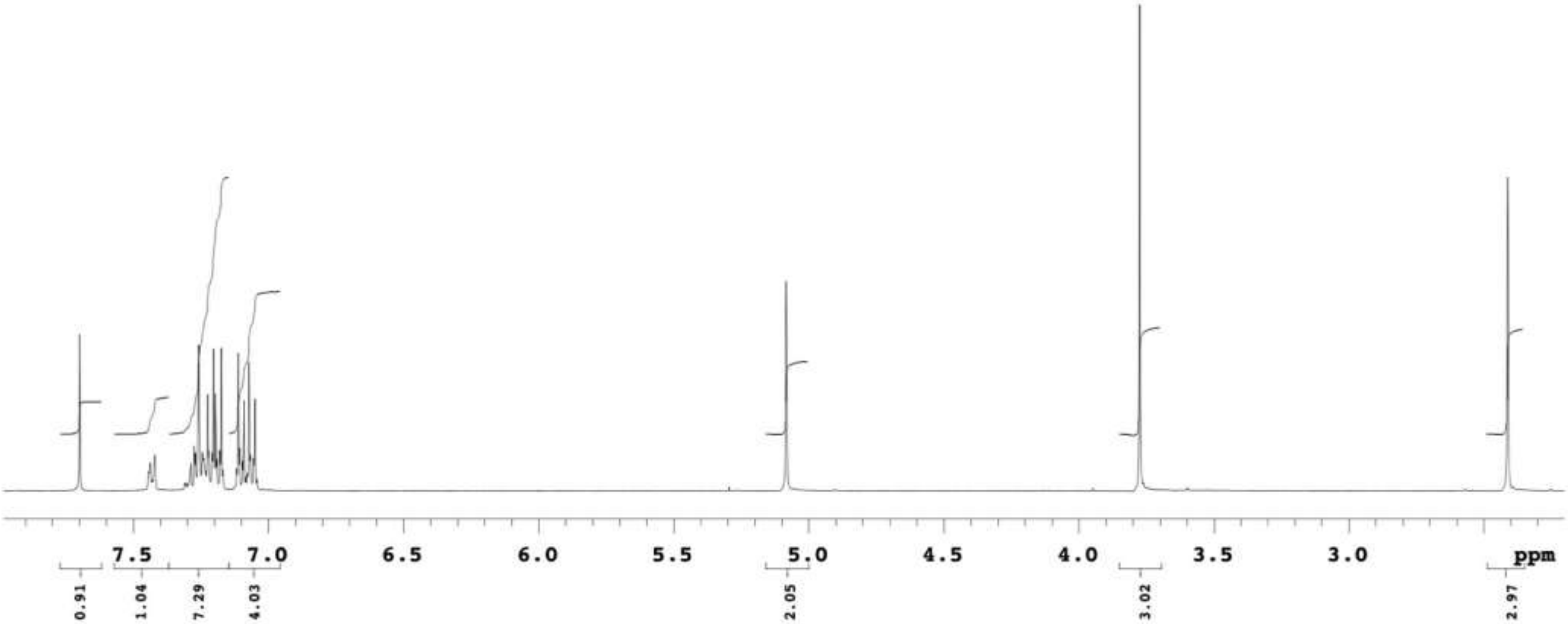

SMY217

Sample Name:
SMY217
Data Collected on:
mercury400-mercury400
Archive directory:
/home/vnmr1/vnmrsys/data
Sample directory:
SMY217_20170614_01
FidFile: current

Pulse Sequence: CARBON (s2pul)
Solvent: cdcl3
Data collected on: Jun 14 2017

Temp. 25.0 C / 298.1 K
Operator: vnmr1

Relax. delay 1.000 sec
Pulse 45.0 degrees
Acq. time 1.550 sec
Width 21141.6 Hz
448 repetitions
OBSERVE C13, 100.6238513 MHz
DECOUPLE H1, 400.1760547 MHz
Power 38 dB
continuously on
WALTZ-16 modulated
DATA PROCESSING
Line broadening 0.5 Hz
FT size 65536
Total time 1 hr, 28 min

Compound 72

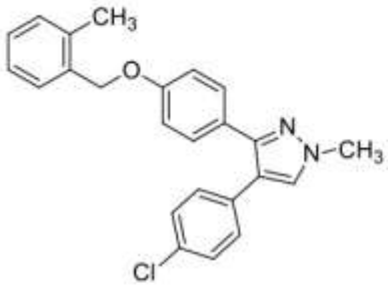

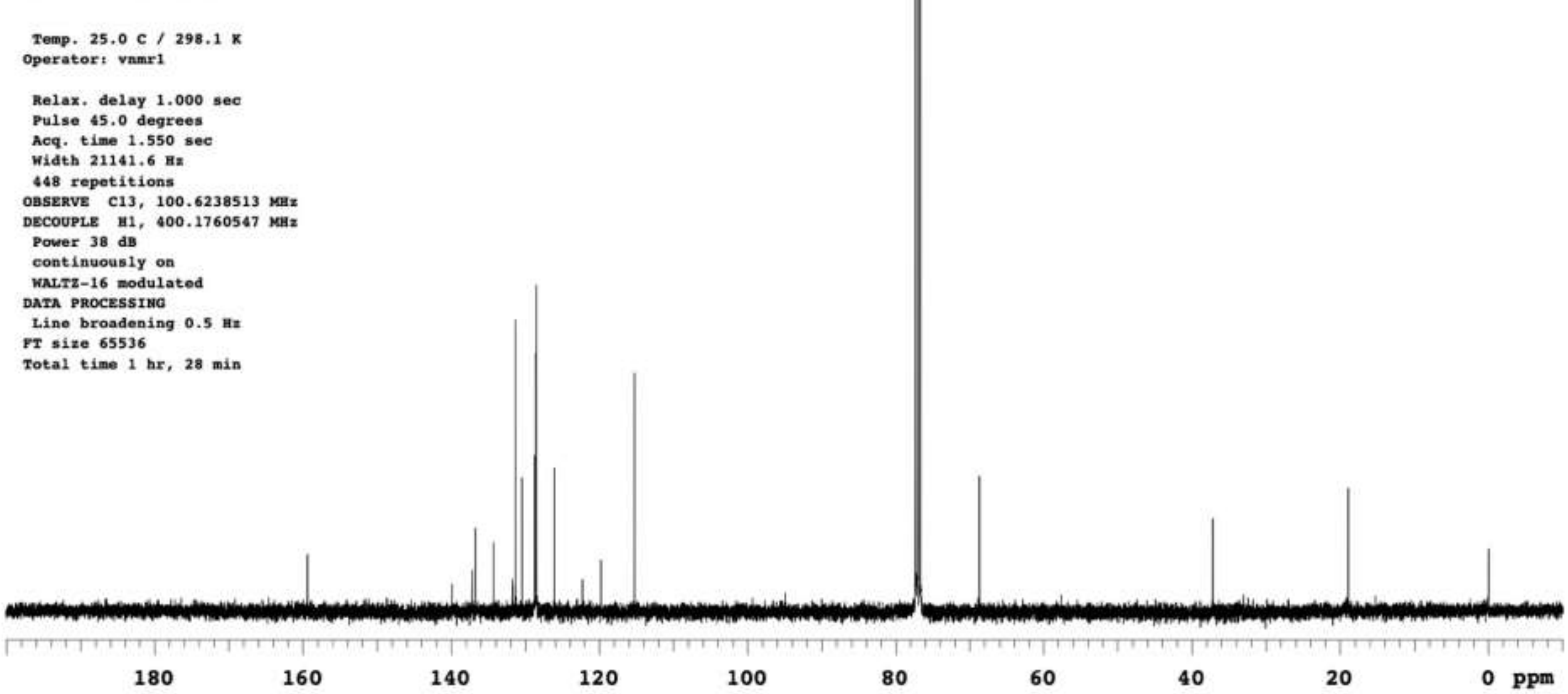

Figure S58. ^{1}H-NMR and ^{13}C-NMR spectrum of Compound **75**

Compound 75

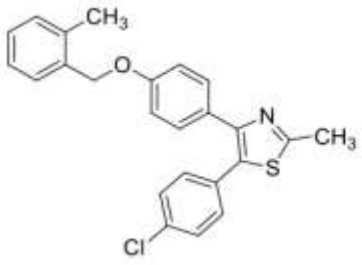

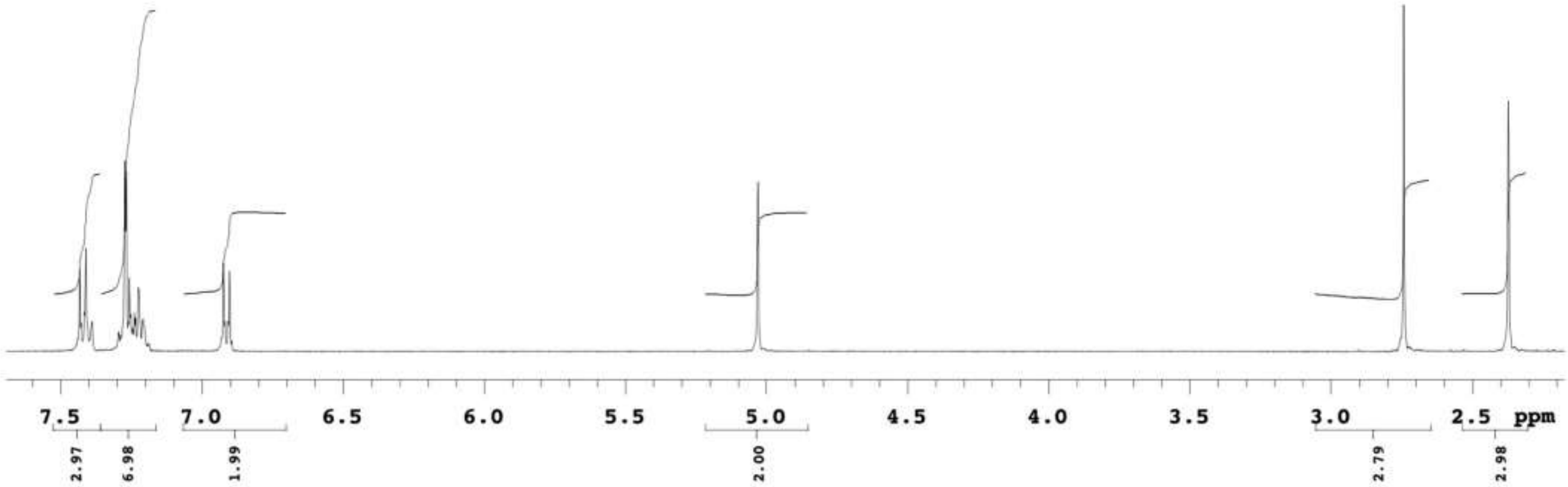

SMY-301

Sample Name:
SMY-301
Data Collected on:
mercury400-mercury400
Archive directory:
/home/vnmr1/vnmrsys/data
Sample directory:
SMY-301_20180309_01
FidFile: current

Pulse Sequence: CARBON (s2pul)
Solvent: cdcl3
Data collected on: Mar 9 2018

Temp. 25.0 C / 298.1 K
Operator: vnmr1

Relax. delay 1.000 sec
Pulse 45.0 degrees
Acq. time 1.550 sec
Width 21141.6 Hz
640 repetitions
OBSERVE C13, 100.6238513 MHz
DECOUPLE H1, 400.1760547 MHz
Power 38 dB
continuously on
WALTZ-16 modulated
DATA PROCESSING
Line broadening 0.5 Hz
FT size 65536
Total time 1 hr, 6 min

Compound 75

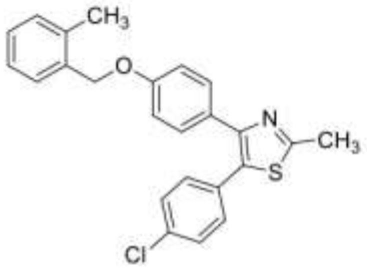

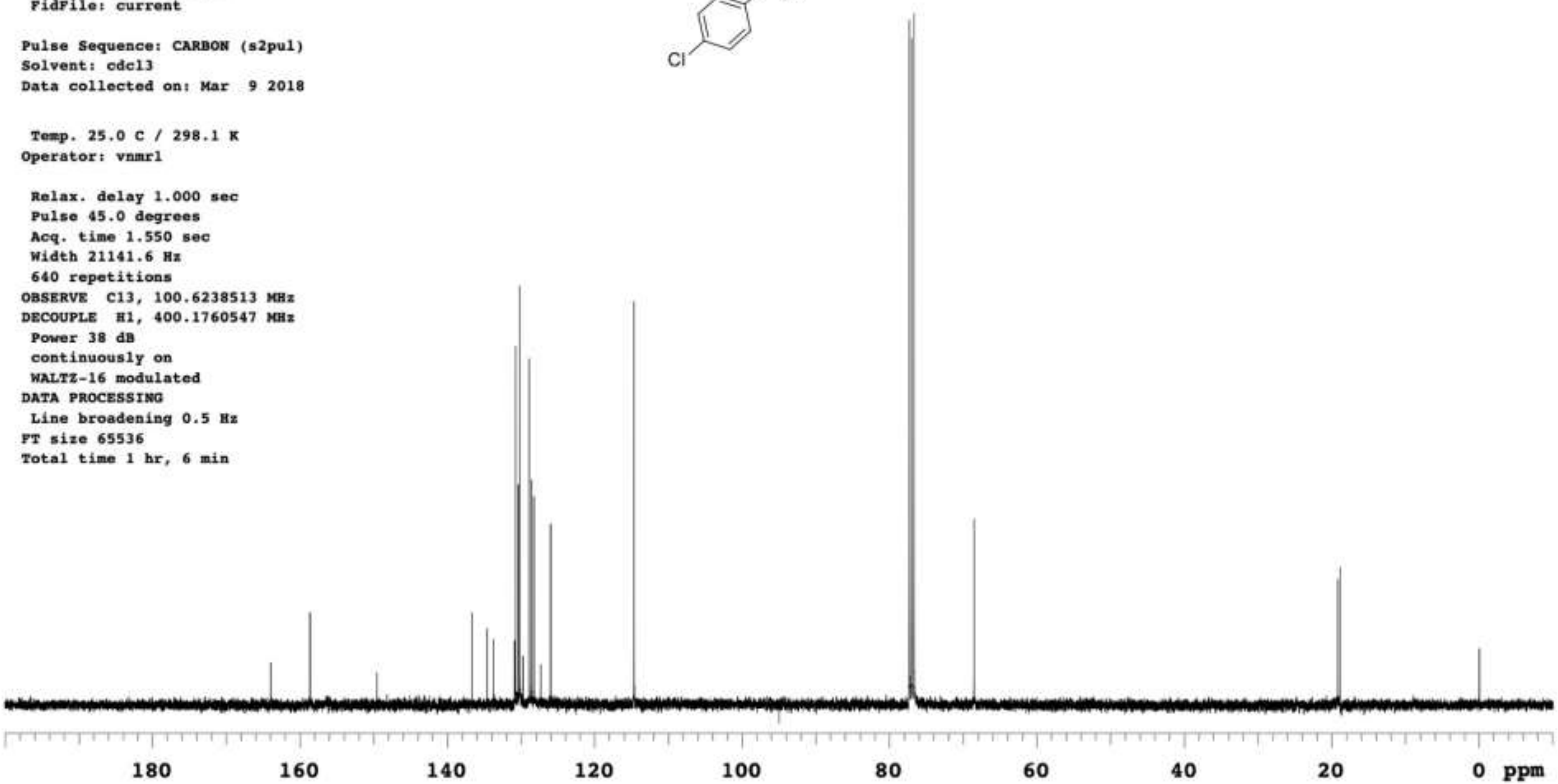

Figure S59. ^{1}H-NMR and ^{13}C-NMR spectrum of Compound **78**

Compound 78

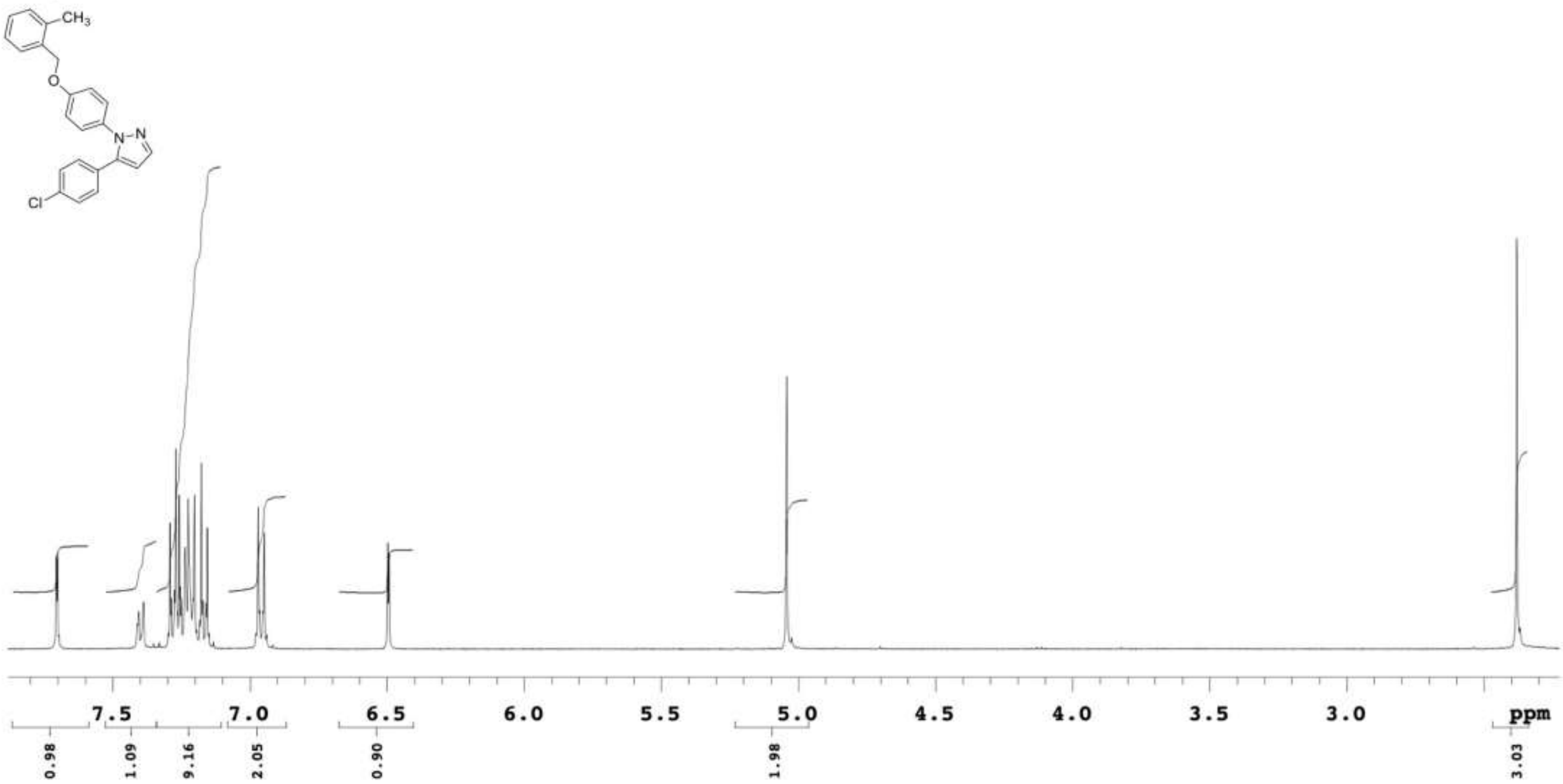

SMY-221

Sample Name:
SMY-221
Data Collected on:
mercury400-mercury400
Archive directory:
/home/vnmr1/vnmrsys/data
Sample directory:
SMY-221_20170420_01
FidFile: current

Pulse Sequence: CARBON (s2pul)
Solvent: cdcl3
Data collected on: Apr 20 2017

Temp. 25.0 C / 298.1 K
Operator: vnmr1

Relax. delay 1.000 sec
Pulse 45.0 degrees
Acq. time 1.304 sec
Width 25125.6 Hz
1600 repetitions
OBSERVE C13, 100.6238513 MHz
DECOUPLE H1, 400.1760547 MHz
Power 38 dB
continuously on
WALTZ-16 modulated
DATA PROCESSING
Line broadening 0.5 Hz
FT size 65536
Total time 1 hr, 20 min

Compound 78

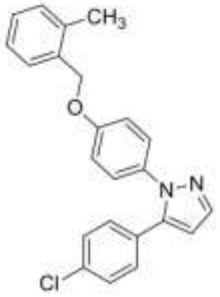

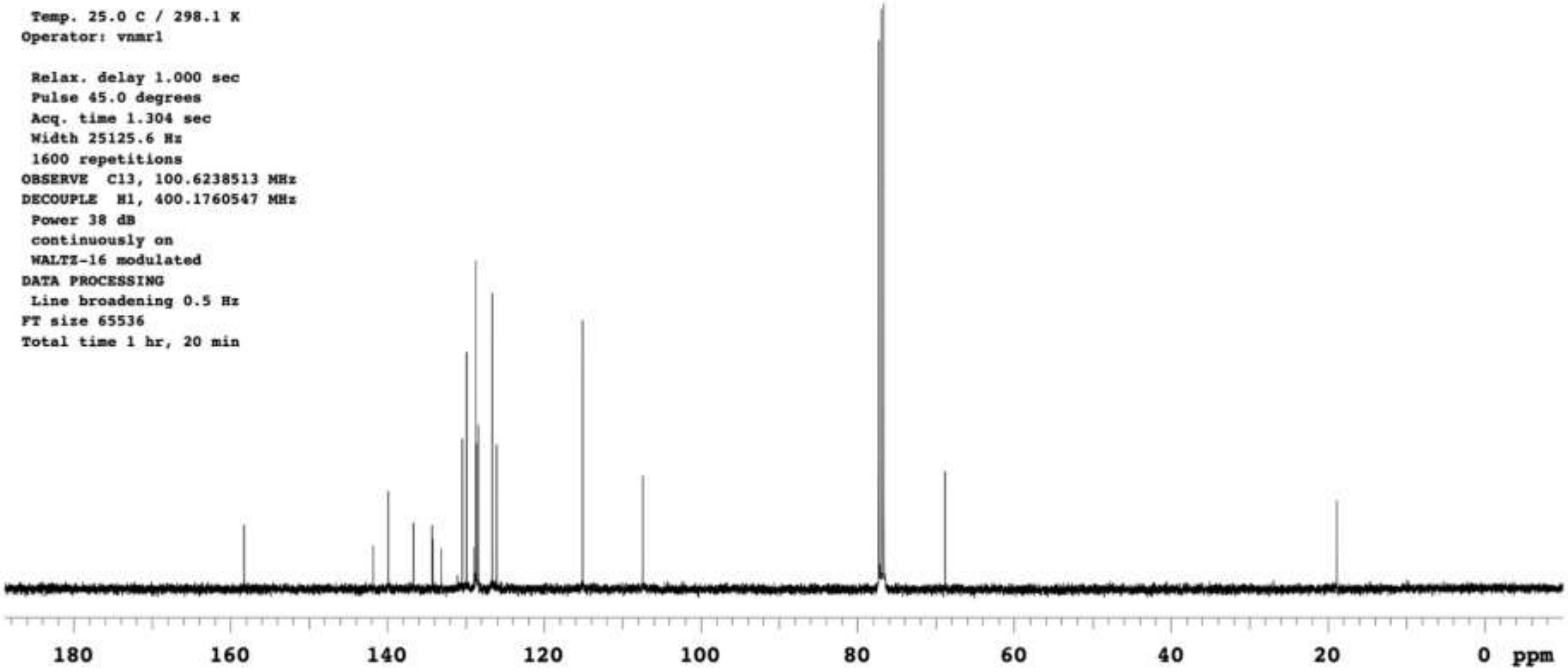

Figure S60. ^{1}H-NMR and ^{13}C-NMR spectrum of Compound **79**

Compound 79

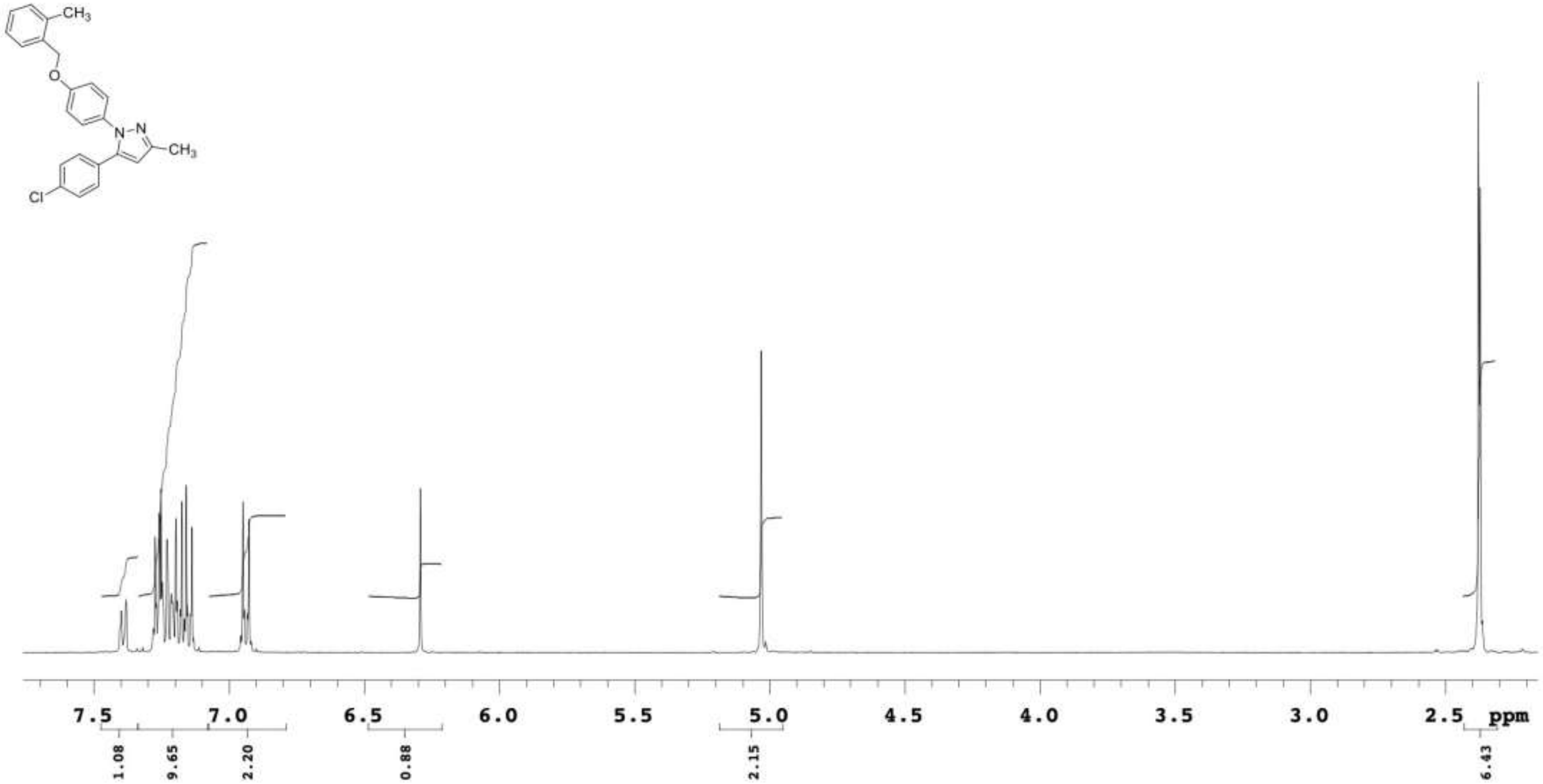

SMY-220

Sample Name:
SMY-220
Data Collected on:
mercury400-mercury400
Archive directory:
/home/vnmr1/vnmrsys/data
Sample directory:
SMY-220_20170420_01
FidFile: CARBON_01

Pulse Sequence: CARBON (s2pul)
Solvent: cdcl3
Data collected on: Apr 20 2017

Temp. 25.0 C / 298.1 K
Operator: vnmr1

Relax. delay 1.000 sec
Pulse 45.0 degrees
Acq. time 1.304 sec
Width 25125.6 Hz
2000 repetitions
OBSERVE C13, 100.6238513 MHz
DECOUPLE H1, 400.1760547 MHz
Power 38 dB
continuously on
WALTZ-16 modulated
DATA PROCESSING
Line broadening 0.5 Hz
FT size 65536
Total time 1 hr, 20 min

Compound 79

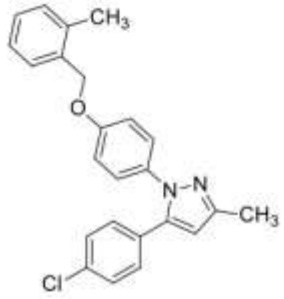

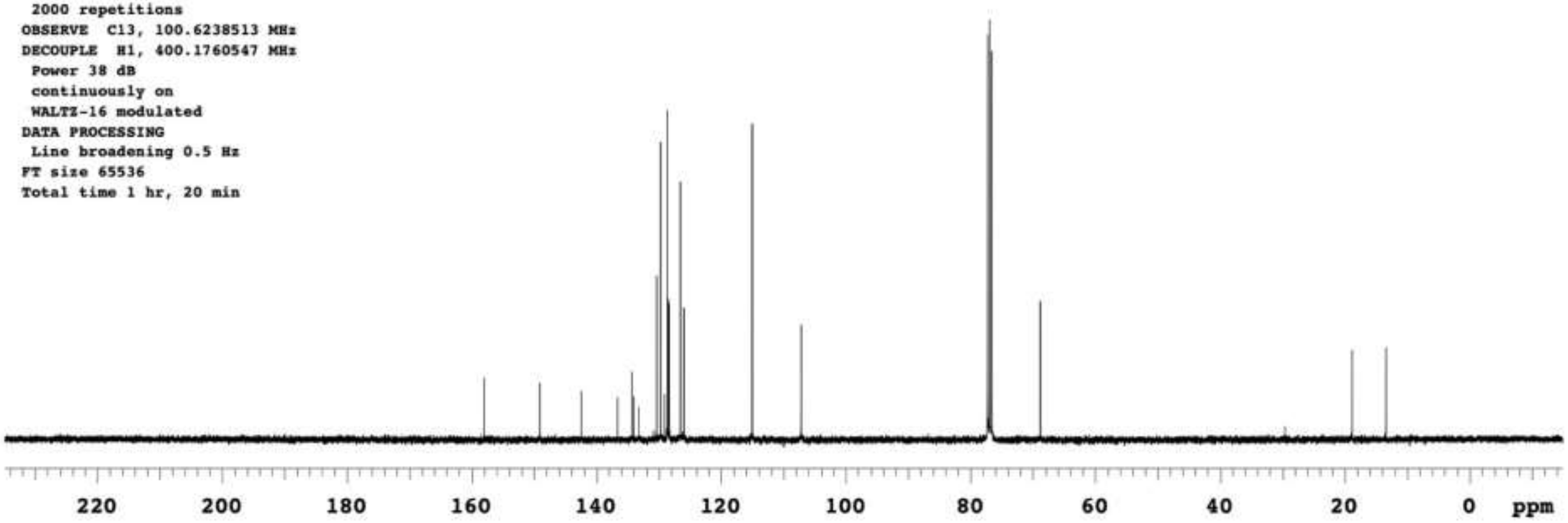

Figure S61. ^{1}H-NMR and ^{13}C-NMR spectrum of Compound **85**

Compound 85

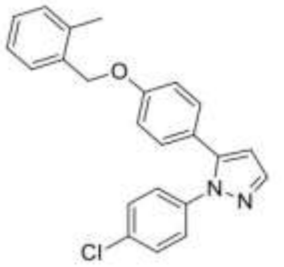

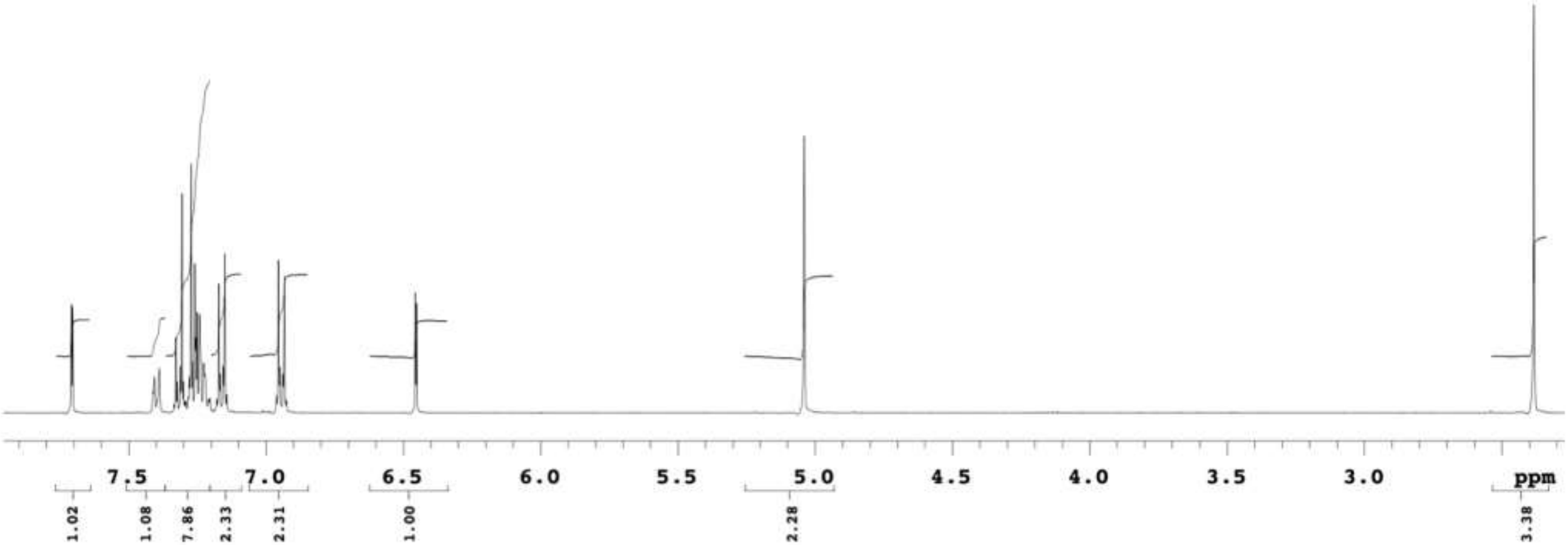

SMY163

Sample Name:
SMY163
Data Collected on:
mercury400-mercury400
Archive directory:
/home/vnmr1/vnmrsys/data
Sample directory:
SMY163_20161118_01
FidFile: CARBON_01

Pulse Sequence: CARBON (s2pul)
Solvent: cdcl3
Data collected on: Nov 18 2016

Temp. 25.0 C / 298.1 K
Operator: vnmr1

Relax. delay 1.000 sec
Pulse 45.0 degrees
Acq. time 1.550 sec
Width 21141.6 Hz
2000 repetitions
OBSERVE C13, 100.6238513 MHz
DECOUPLE H1, 400.1760547 MHz
Power 38 dB
continuously on
WALTZ-16 modulated
DATA PROCESSING
Line broadening 0.5 Hz
FT size 65536
Total time 1 hr, 28 min

Compound 85

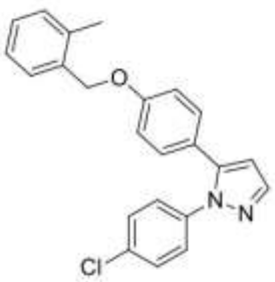

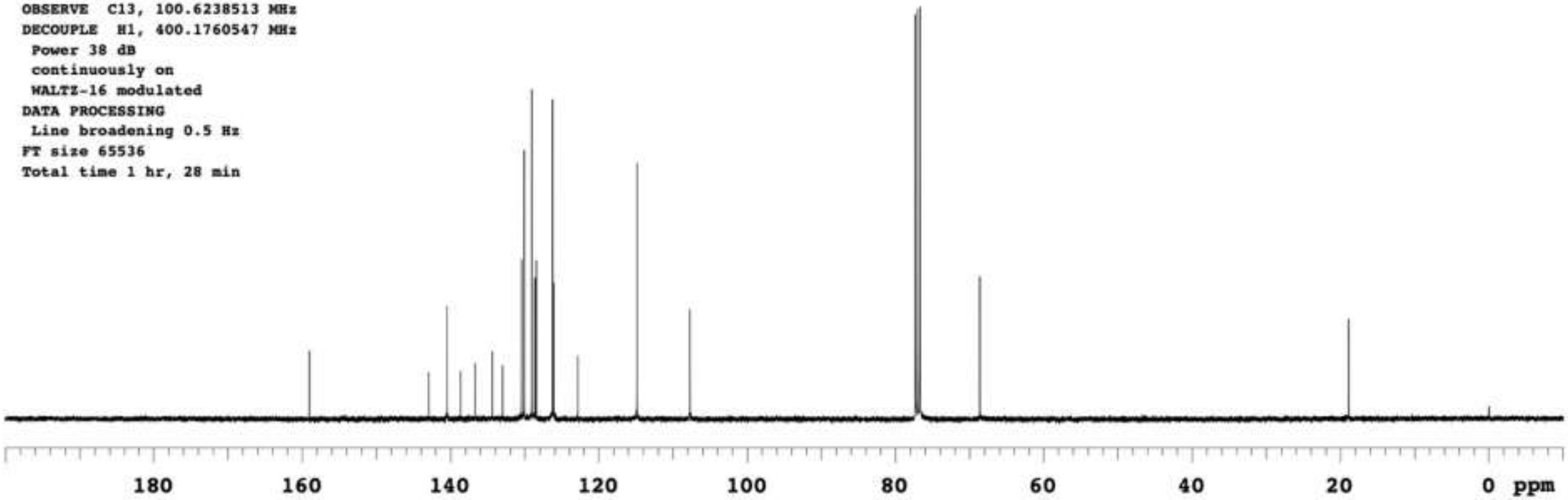

Figure S62. ^{1}H-NMR and ^{13}C-NMR spectrum of Compound **86**

Compound 86

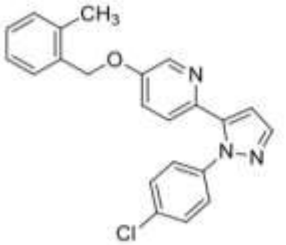

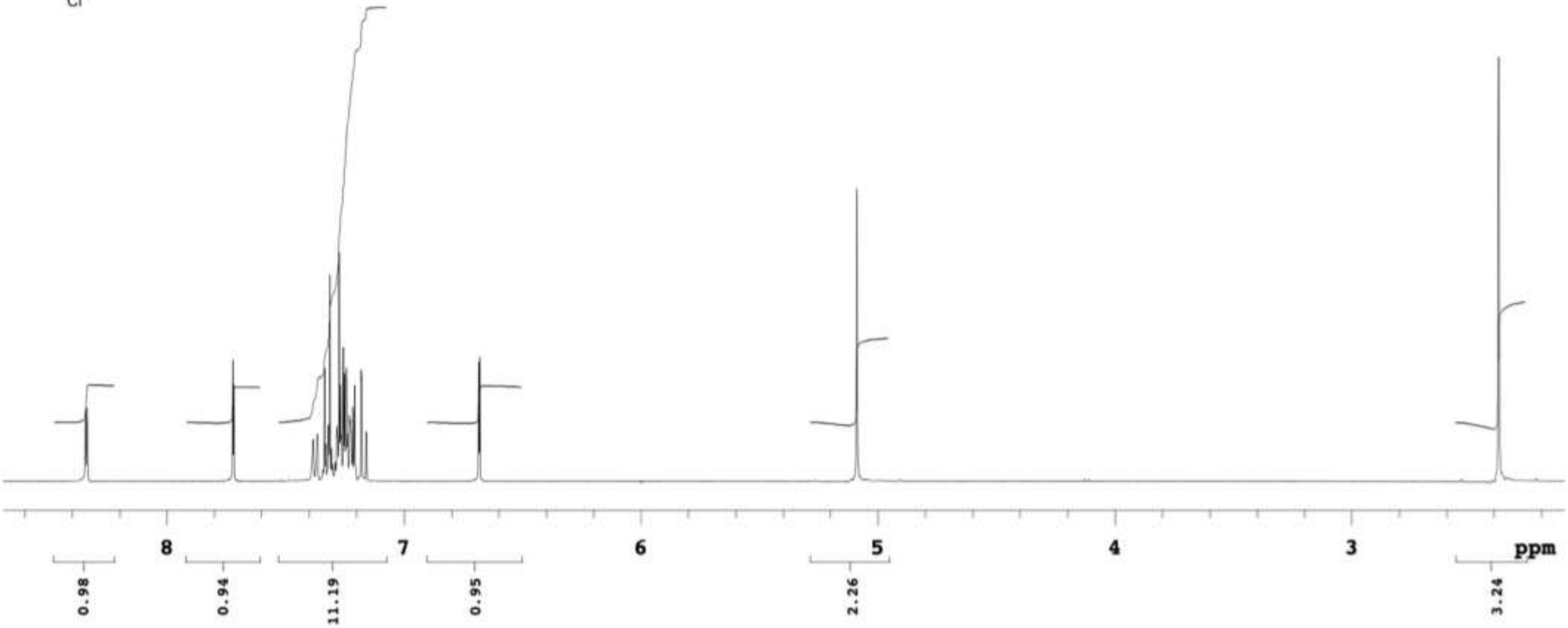

SMY270

Sample Name:
SMY270
Data Collected on:
mercury400-mercury400
Archive directory:
/home/vnmr1/vnmrsys/data
Sample directory:
SMY270_20171215_01
FidFile: current

Pulse Sequence: CARBON (s2pul)
Solvent: cdcl3
Data collected on: Dec 15 2017

Temp. 25.0 C / 298.1 K
Operator: vnmr1

Relax. delay 1.000 sec
Pulse 45.0 degrees
Acq. time 1.304 sec
Width 25125.6 Hz
1152 repetitions
OBSERVE C13, 100.6238513 MHz
DECOUPLE H1, 400.1760547 MHz
Power 38 dB
continuously on
WALTZ-16 modulated
DATA PROCESSING
Line broadening 0.5 Hz
FT size 65536
Total time 1 hr

Compound 86

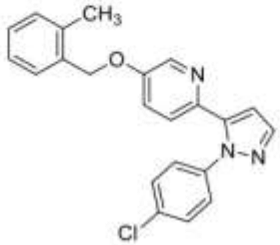

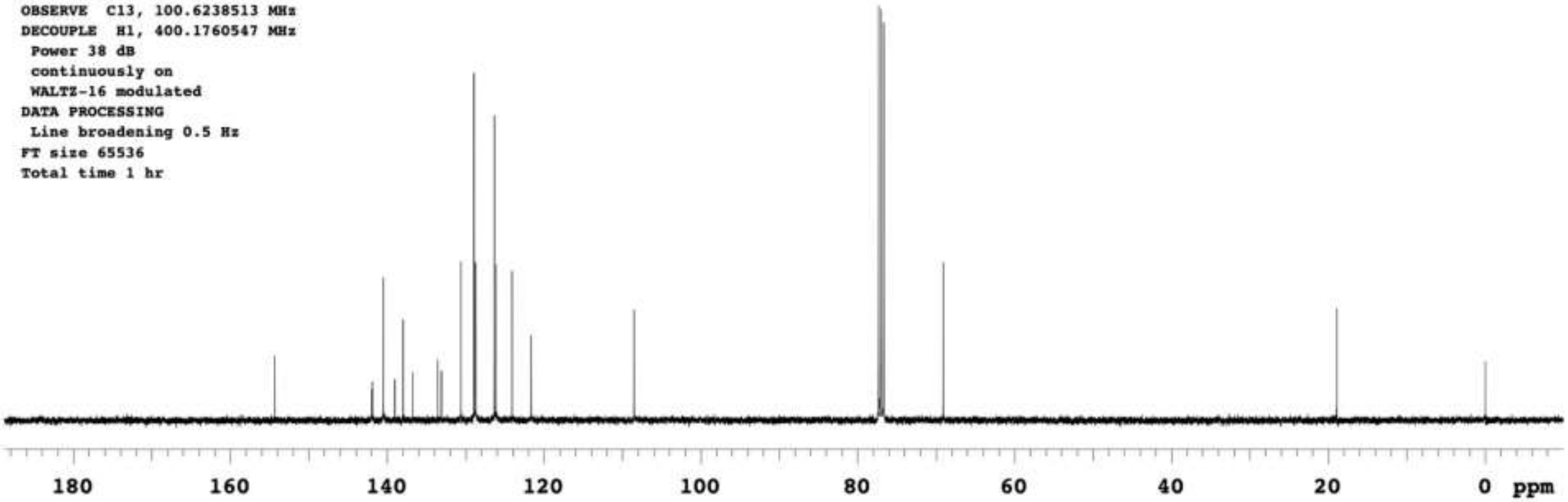

Figure S63. ^{1}H-NMR and ^{13}C-NMR spectrum of Compound **87**

Compound 87

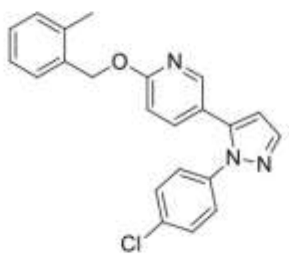

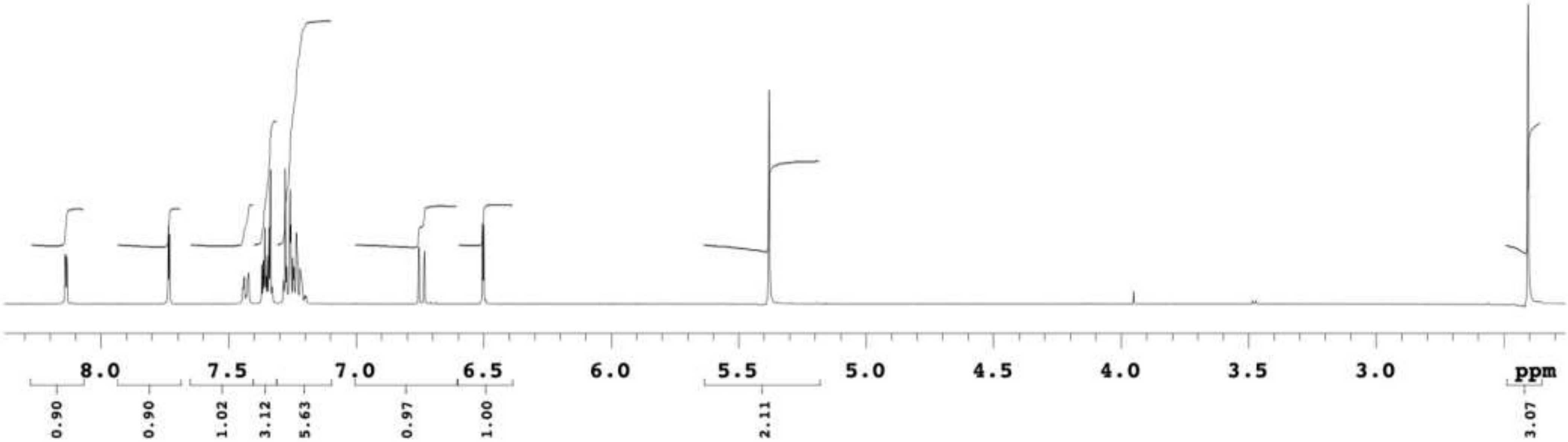

KUB368

Sample Name:
KUB368
Data Collected on:
mercury400-mercury400
Archive directory:
/home/vnmr1/vnmrsys/data
Sample directory:
KUB368_20181115_01
FidFile: CARBON_01

Pulse Sequence: CARBON (s2pul)
Solvent: cdcl3
Data collected on: Nov 15 2018

Temp. 25.0 C / 298.1 K
Operator: vnmr1

Relax. delay 1.000 sec
Pulse 45.0 degrees
Acq. time 1.550 sec
Width 21141.6 Hz
1000 repetitions
OBSERVE C13, 100.6238513 MHz
DECOUPLE H1, 400.1760547 MHz
Power 38 dB
continuously on
WALTZ-16 modulated
DATA PROCESSING
Line broadening 0.5 Hz
FT size 65536
Total time 44 min

Compound 87

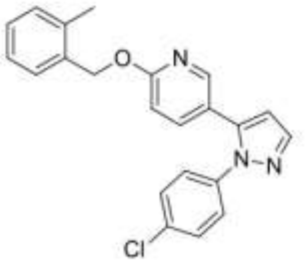

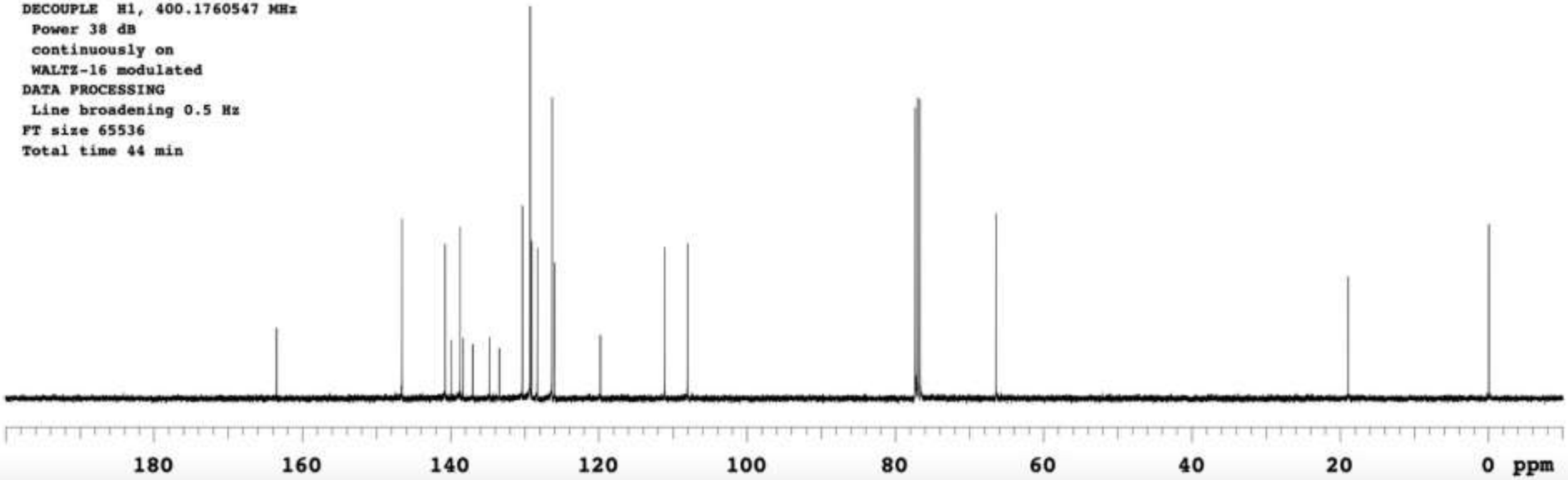

Figure S64. ^{1}H-NMR and ^{13}C-NMR spectrum of Compound **88**

Compound 88

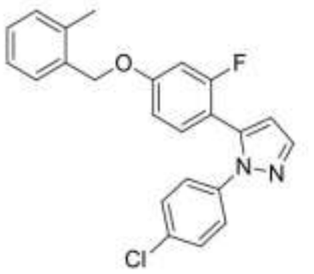

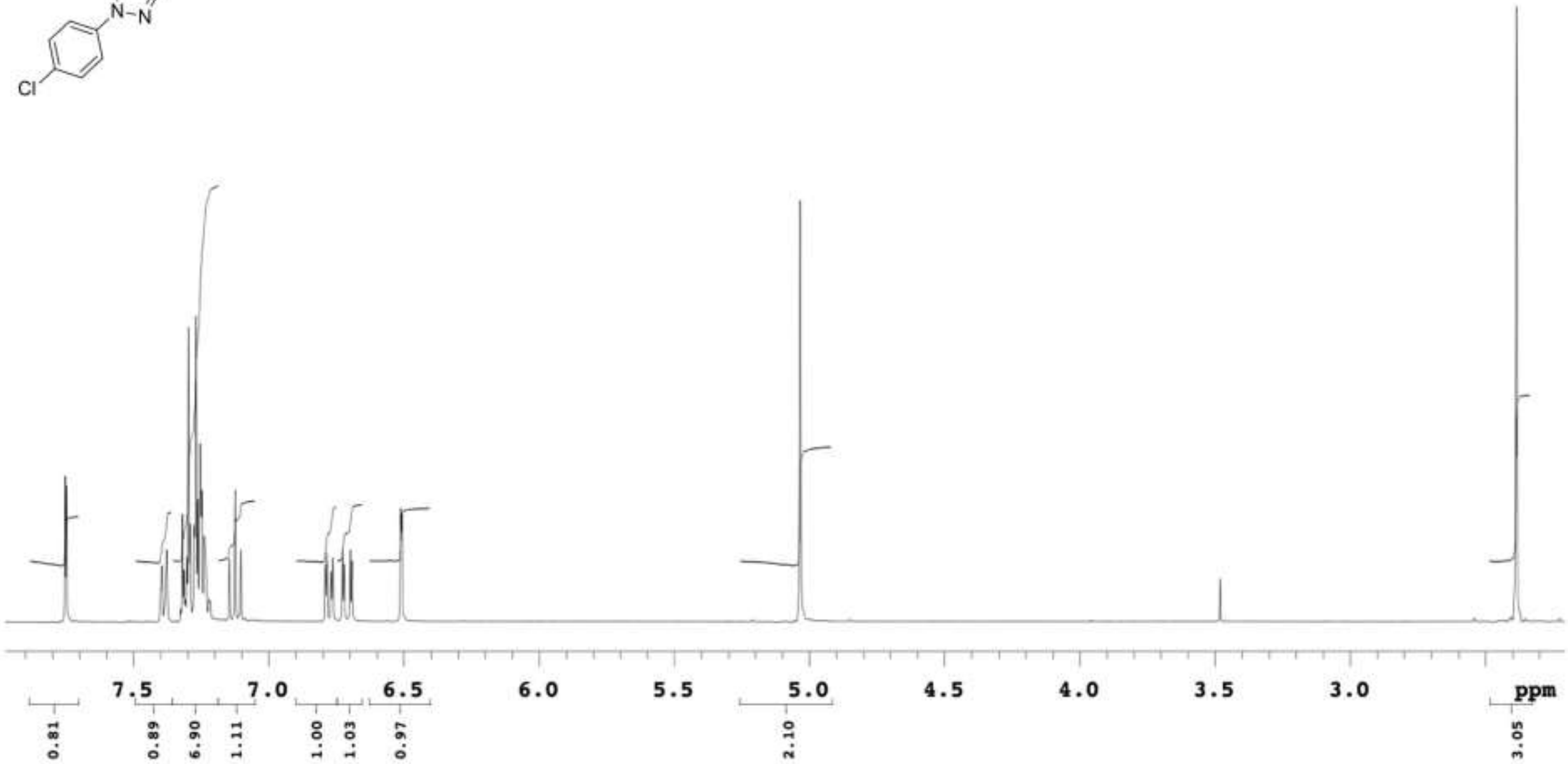

KUB325

Sample Name:
KUB325
Data Collected on:
mercury400-mercury400
Archive directory:
/home/vnmr1/vnmrsys/data
Sample directory:
KUB325_20180606_01
FidFile: current

Pulse Sequence: CARBON (s2pul)
Solvent: cdcl3
Data collected on: Jun 6 2018

Temp. 25.0 C / 298.1 K
Operator: vnmr1

Relax. delay 1.000 sec
Pulse 45.0 degrees
Acq. time 1.304 sec
Width 25125.6 Hz
448 repetitions
OBSERVE C13, 100.6238331 MHz
DECOUPLE H1, 400.1760547 MHz
Power 38 dB
continuously on
WALTZ-16 modulated
DATA PROCESSING
Line broadening 0.5 Hz
FT size 65536
Total time 1 hr, 20 min

Compound 88

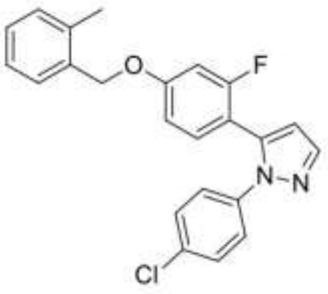

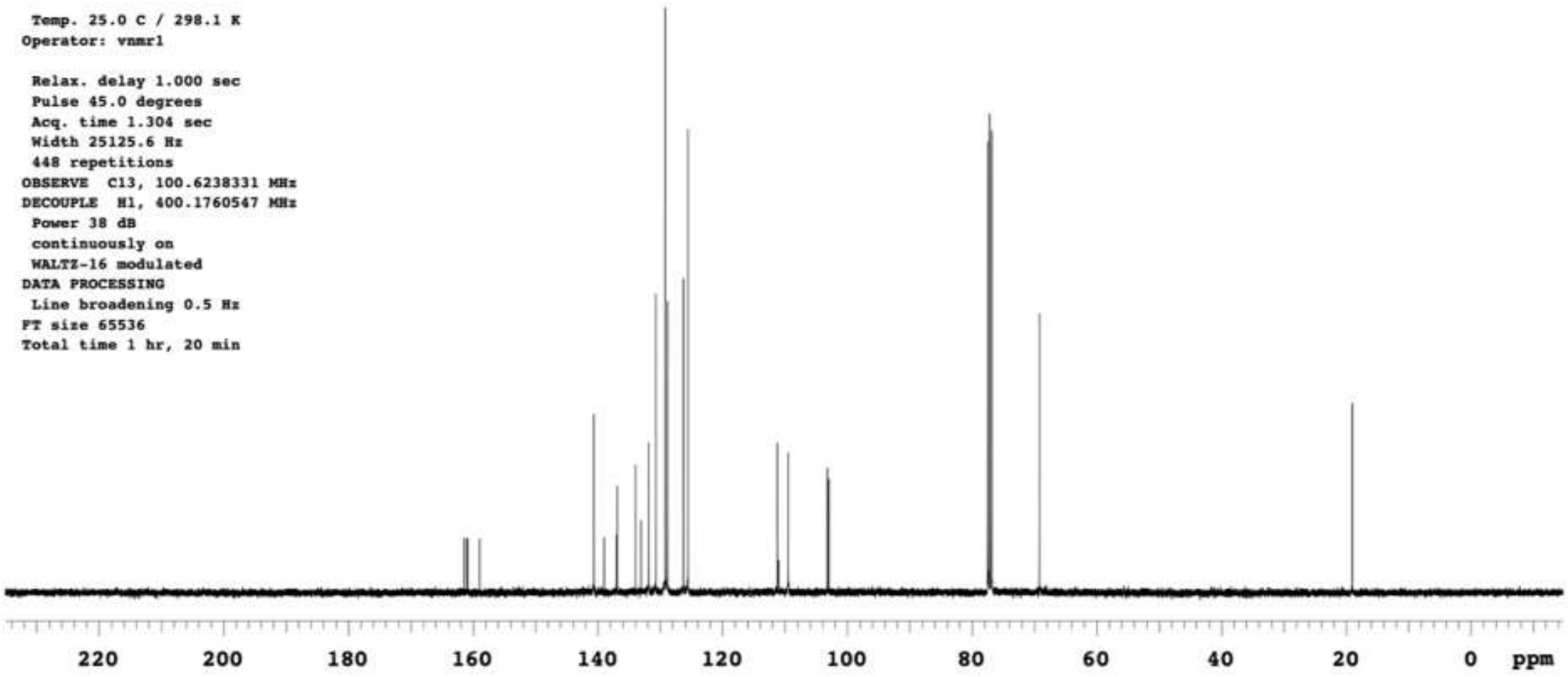

Figure S65. ^{1}H-NMR and ^{13}C-NMR spectrum of Compound **89**

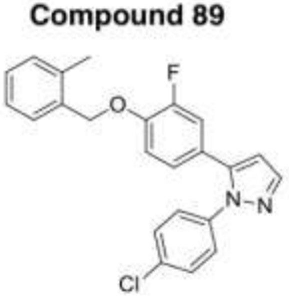

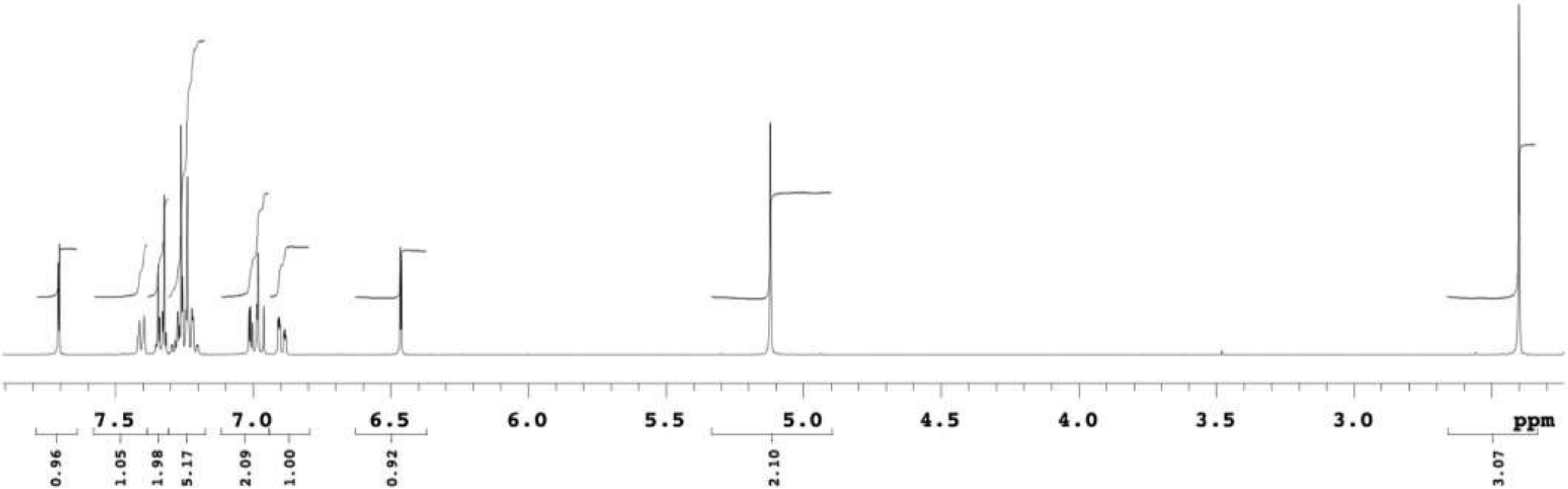

KUB329

Sample Name:
KUB329
Data Collected on:
mercury400-mercury400
Archive directory:
/home/vnmr1/vnmrsys/data
Sample directory:
KUB329_20180606_01
FidFile: current

Pulse Sequence: CARBON (s2pul)
Solvent: cdcl3
Data collected on: Jun 6 2018

Temp. 25.0 C / 298.1 K
Operator: vnmr1

Relax. delay 1.000 sec
Pulse 45.0 degrees
Acq. time 1.550 sec
Width 21141.6 Hz
192 repetitions
OBSERVE C13, 100.6238326 MHz
DECOUPLE H1, 400.1760547 MHz
Power 38 dB
continuously on
WALTZ-16 modulated
DATA PROCESSING
Line broadening 0.5 Hz
FT size 65536
Total time 1 hr, 28 min

Compound 89

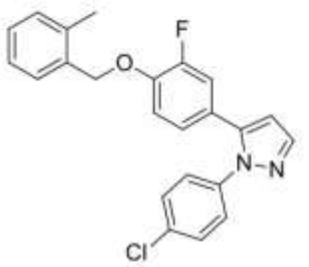

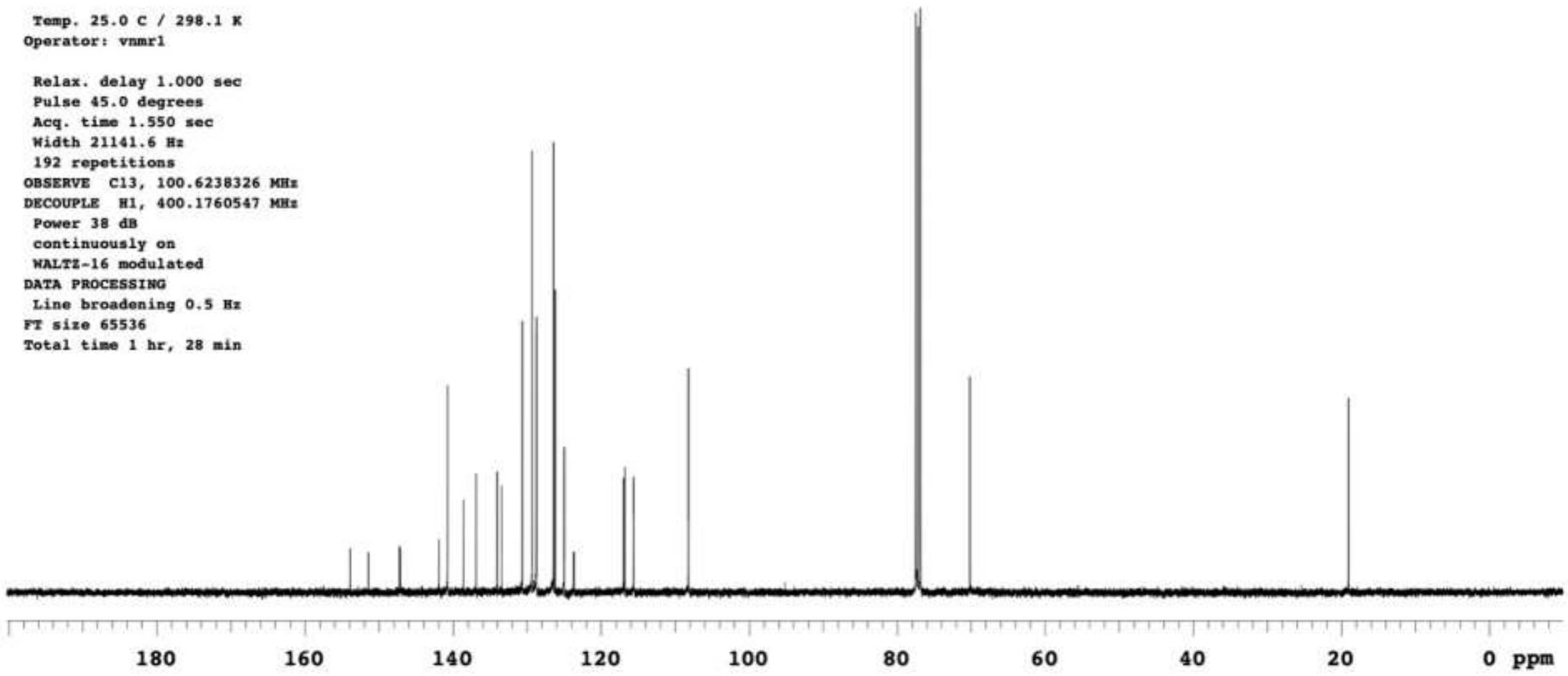

Figure S66. ^{1}H-NMR and ^{13}C-NMR spectrum of Compound **91**

Compound 91

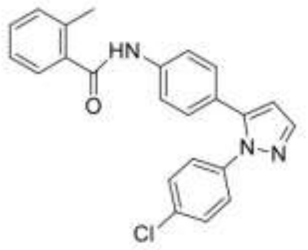

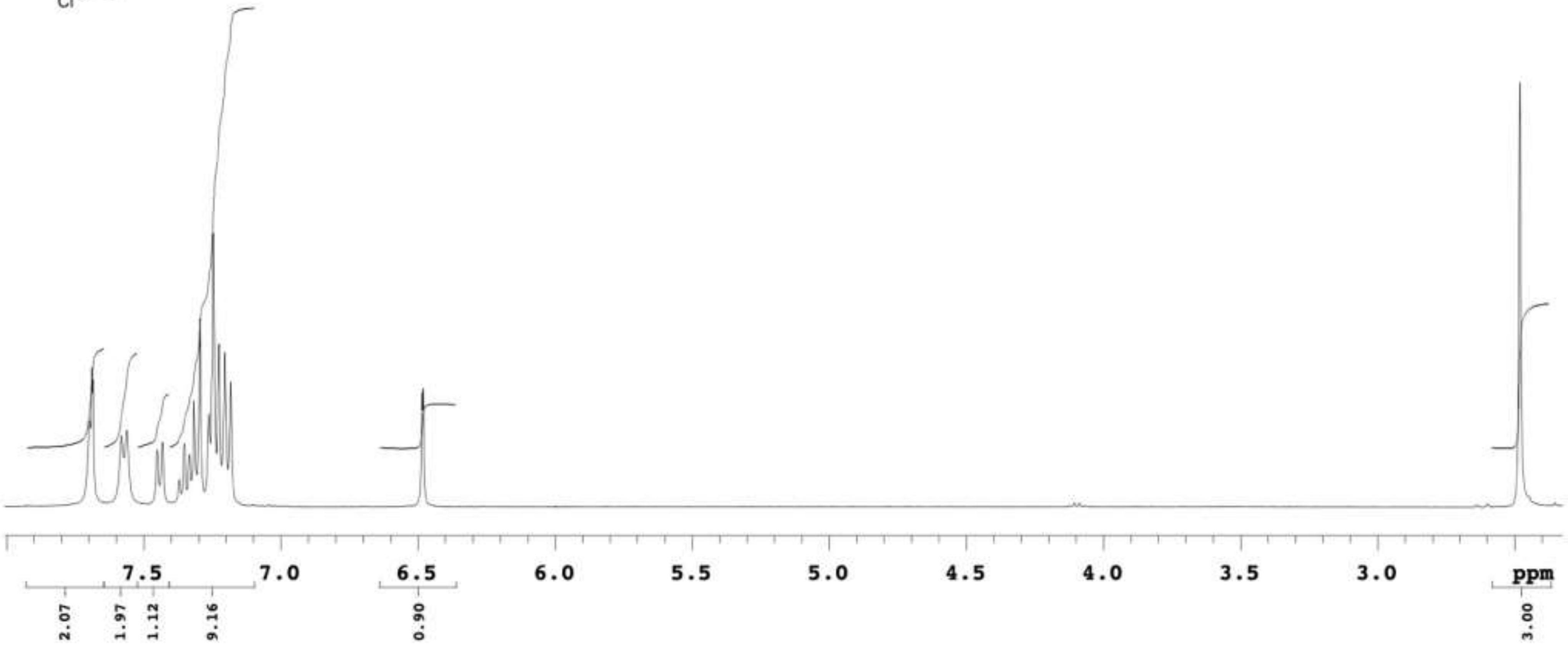

KUB341

Sample Name:
KUB341
Data Collected on:
mercury400-mercury400
Archive directory:
/home/vnmr1/vnmrsys/data
Sample directory:
KUB341_20180719_01
FidFile: current

Pulse Sequence: CARBON (s2pul)
Solvent: cdcl3
Data collected on: Jul 19 2018

Temp. 25.0 C / 298.1 K
Operator: vnmr1

Relax. delay 1.000 sec
Pulse 45.0 degrees
Acq. time 1.304 sec
Width 25125.6 Hz
1408 repetitions
OBSERVE C13, 100.6238513 MHz
DECOUPLE H1, 400.1760547 MHz
Power 38 dB
continuously on
WALTZ-16 modulated
DATA PROCESSING
Line broadening 0.5 Hz
FT size 65536
Total time 1 hr

Compound 91

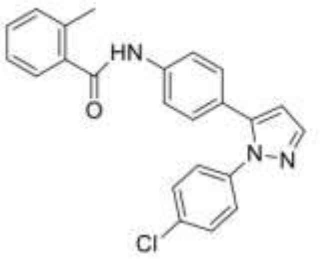

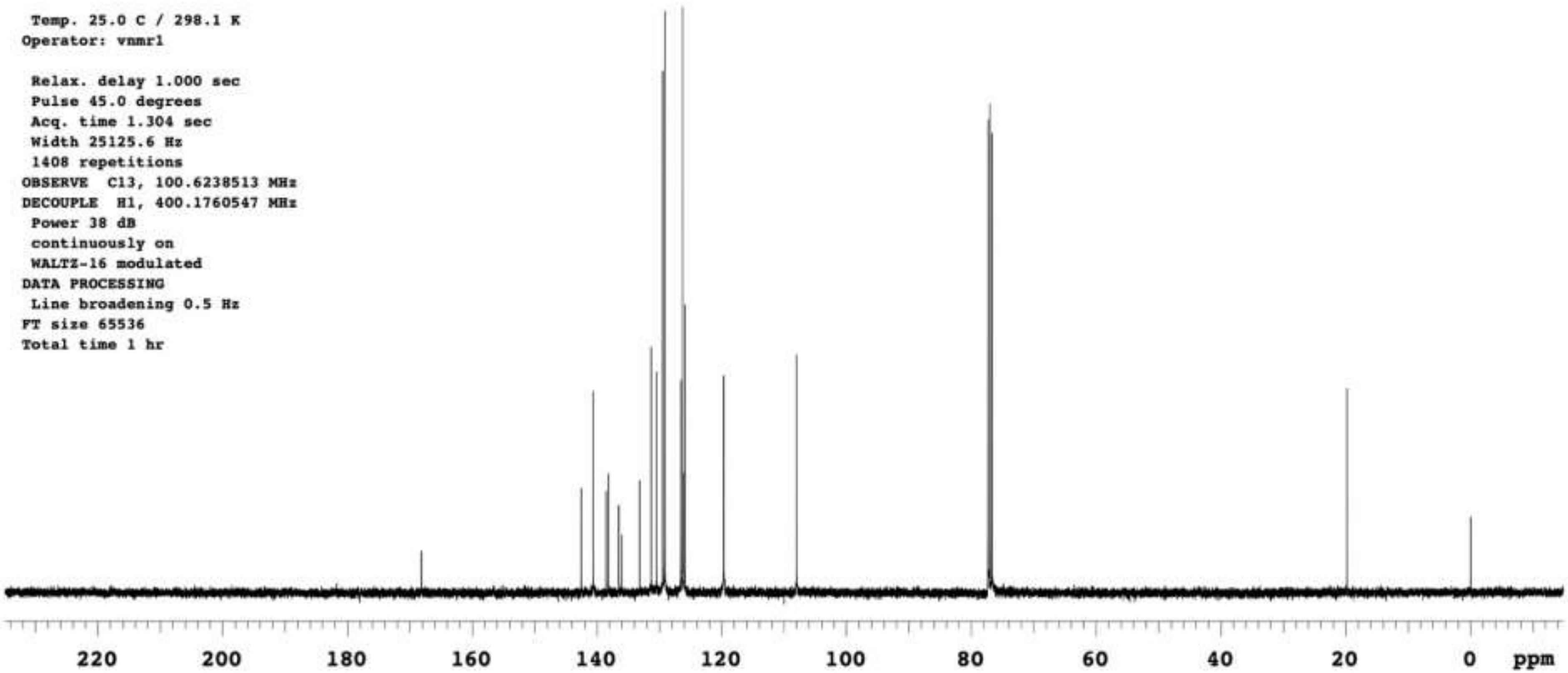

Figure S67. ^{1}H-NMR and ^{13}C-NMR spectrum of Compound **92**

Compound 92

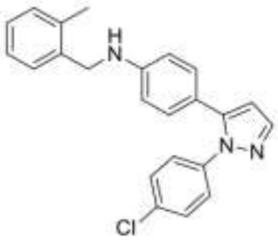

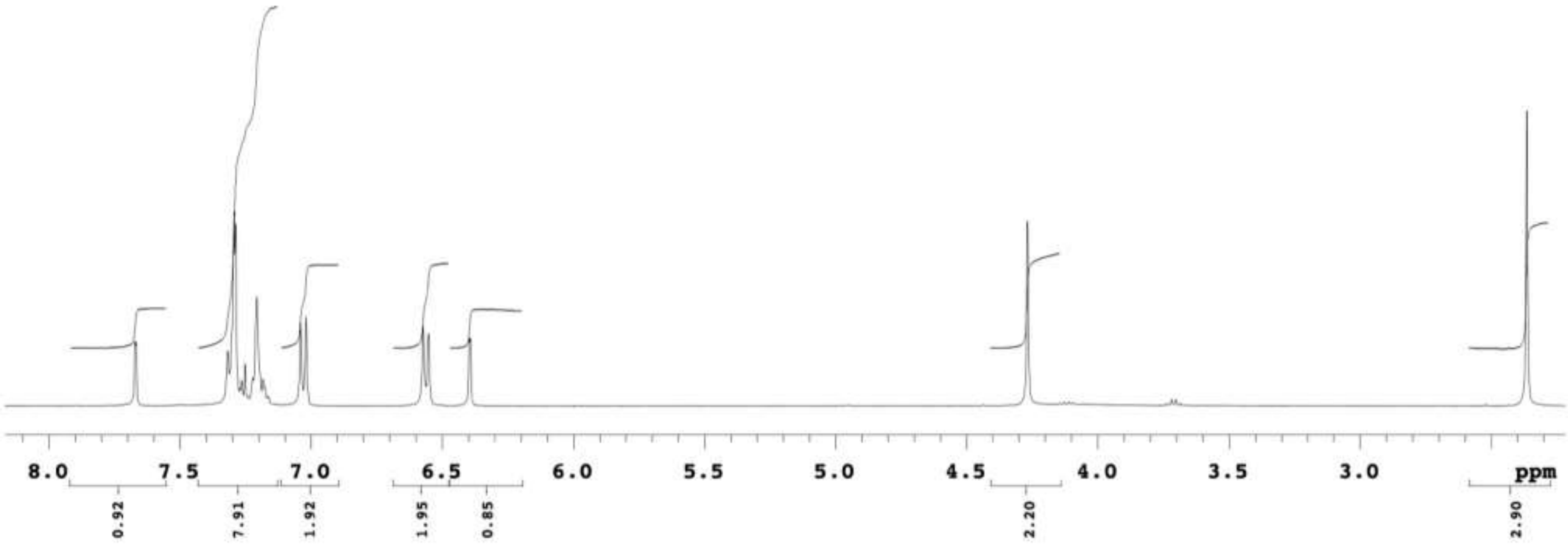

KUB342

Sample Name:
KUB342
Data Collected on:
mercury400-mercury400
Archive directory:
/home/vnmr1/vnmrsys/data
Sample directory:
KUB342_20180720_01
FidFile: CARBON_01

Pulse Sequence: CARBON (s2pul)
Solvent: cdcl3
Data collected on: Jul 20 2018

Temp. 25.0 C / 298.1 K
Operator: vnmr1

Relax. delay 1.000 sec
Pulse 45.0 degrees
Acq. time 1.550 sec
Width 21141.6 Hz
1256 repetitions
OBSERVE C13, 100.6238513 MHz
DECOUPLE H1, 400.1760547 MHz
Power 38 dB
continuously on
WALTZ-16 modulated
DATA PROCESSING
Line broadening 0.5 Hz
FT size 65536
Total time 55 min

Compound 92

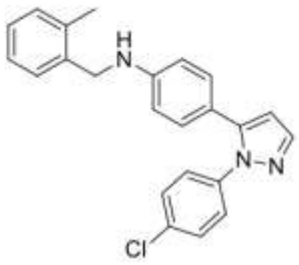

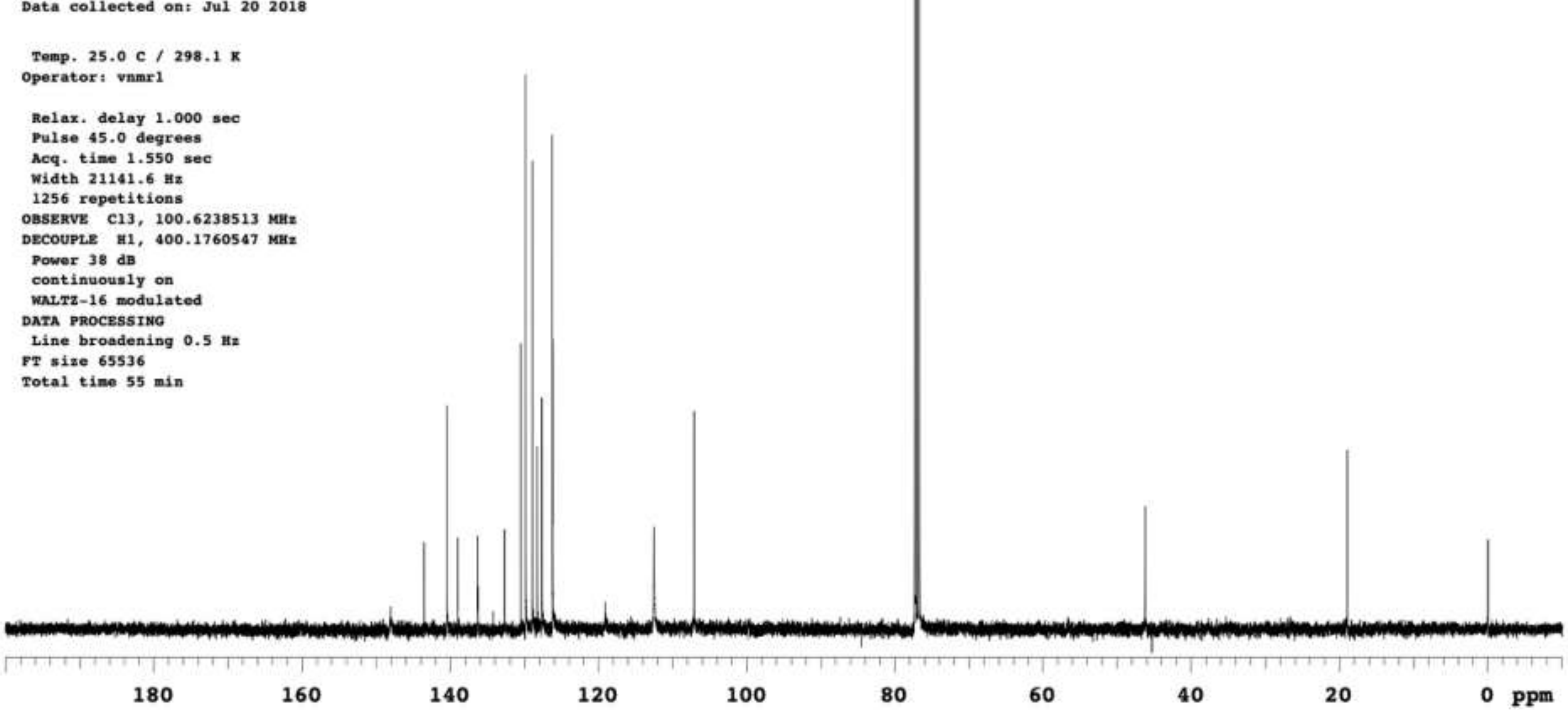

Supplement: Supplementary file 1 — ao2c03405_si_001.pdf [file ao2c03405_si_001.pdf]
